# Global, regional, and national trends in glaucoma burden among adults aged 45 years and older, 1990-2021

**DOI:** 10.1016/j.aopr.2026.06.004

**Published:** 2026-06-20

**Authors:** Chao Wu, Fan Cao, Baorui Chu, Xianyang Liu, Shuhao Zeng, Yakun Wang, Hui Feng, Xinyuan Chen, Ningli Wang, Shengping Hou

**Affiliations:** a Beijing Institute of Ophthalmology, Beijing Tongren Eye Center, Beijing Tongren Hospital, Capital Medical University, Beijing, China; b Department of Ophthalmology, Qilu Hospital, Shandong University, Jinan, China

**Keywords:** Annual percentage change, Average annual percentage change, Glaucoma, Global burden of disease study

## Abstract

**Purpose:**

Glaucoma is the leading cause of irreversible blindness worldwide. This study aimed to assess global, regional, and national trends in glaucoma burden among adults aged ≥45 years from 1990 to 2021.

**Methods:**

Data were obtained from the Global Burden of Disease 2021 study. Prevalence and disability-adjusted life-years (DALYs) for glaucoma were analyzed by age, sex, region, and Sociodemographic Index (SDI). Joinpoint regression identified trend changes, and frontier analysis benchmarked country-level performance.

**Results:**

Globally, age-standardized prevalence declined from 116.29 to 90.12 per 100 000 (average annual percentage change, AAPC = −0.82), and DALYs from 13.37 to 9.05 (AAPC = −1.24), while absolute cases increased by 86.33% due to population aging. Five significant trend changes were identified (1994, 2001, 2006, 2010, 2019). The largest prevalence reduction was in adults aged 90–94 years. East Asia experienced the most substantial declines. SDI showed a strong negative correlation with glaucoma burden. Sub-Saharan Africa had the highest burden, while high-SDI regions had the lowest.

**Conclusion*s*:**

Global age-standardized prevalence and DALYs for glaucoma declined between 1990 and 2021, with accelerated declines temporally coinciding with key therapeutic advances. The absolute number of affected individuals increased due to population aging, underscoring the need for targeted screening programs and public awareness campaigns emphasizing regular eye examinations for early detection.

## 1 Introduction

Glaucoma comprises a heterogenous group of conditions that lead to irreversible vision loss,^1^^,^^2^ characterized by progressive loss of retinal ganglion cells and optic nerve injury, often associated with elevated intraocular pressure.^1^^,^^2^ Glaucoma is the leading cause of irreversible blindness worldwide.^3^ Considering the gradual vision loss and asymmetry of disease between the eyes, patients are often in the advanced stage when they are aware of vision loss.^4^ Currently, though there is no treatment that can restore vision lost from glaucoma, treatment is effective at slowing the rate of vision loss from glaucoma, so case detection and timely therapeutic intervention are essential. Facing such a large group of glaucoma patients, addressing the challenge of conducting cost-effective and efficient glaucoma screenings has emerged as a critical public health concern across the globe. Worse still, as populations age, glaucoma prevalence will rise dramatically in the coming decades^5^ and a better understanding of the global burden of glaucoma to prevent unnecessary, preventable blindness becomes crucial.

The Global Burden of Disease (GBD) study offers a valuable platform for assessing the health impacts of diseases and injuries.^6^^,^^7^ Researchers can gain insights into the prevalence and disability-adjusted life-years (DALYs) of diseases or injuries, thereby obtaining a comprehensive understanding in global, regional, and national level.^8^^,^^9^

In this study, we aimed to examine the global trends of glaucoma prevalence and DALYs since 1990 and then to identify the years with the most substantial changes in trends of the above indicators; to shed light on the global trends by age groups, sex, and Sociodemographic Index (SDI); and to report trends at the regional and national level.

## 2 Methods

### 2.1 GBD data sources and case definitions

This study utilized data from the GBD 2021.^10^ Glaucoma was defined as increased intraocular pressure leading to optic nerve damage. The GBD cause list includes primary open-angle glaucoma (POAG), primary angle-closure glaucoma, and secondary glaucoma as a single aggregated entity. Vision loss severity was classified into three levels based on distance visual acuity: moderate (≥6/60 to <6/18), severe (≥3/60 to <6/60), and blindness (<3/60 or visual field <10°).

Input data for glaucoma burden estimation were derived from population-based surveys measuring visual acuity, including peer-reviewed literature, grey literature, unit record data, and contributions from the Vision Loss Expert Group (VLEG). For GBD 2021, cause-specific vision loss models (including glaucoma) utilized 500 sources from 113 countries.

The GBD study employs DisMod-MR 2.1, a Bayesian meta-regression tool that generates prevalence estimates by year, age, sex, and location. Total prevalence is first estimated for each severity level ("envelopes"), followed by cause-specific estimates. For glaucoma, two separate models are run: one for combined moderate and severe vision loss (as input data are typically aggregated), and one for blindness. Based on VLEG expert input, a minimum age of 45 years is applied to all glaucoma models.

### 2.2 Data collection

Two metrics—prevalence and DALYs—were extracted for analysis by age, sex, and location. SDI serves as a comprehensive predictor that characterizes the sociodemographic status of a country and exhibits a strong correlation with the health development outcomes of its population.^11^ It ranges from 0 to 1, serving as an indicator of the socioeconomic health and progress of a region or country.^6^^,^^12^ The SDI categorizes regions into quintiles: low, low-middle, middle, high-middle, and high SDI.

### 2.3 Data analysis

Counts and rates for prevalence and DALYs of glaucoma on global, regional, and national levels were calculated. For calculations, GBD estimates were retained at their full available precision and independently rounded to two decimal places for presentation. Accordingly, the sums of displayed subgroup estimates may differ slightly from the corresponding displayed aggregate estimates. To quantify glaucoma trends, we employed a statistical methodology based on the annual percentage changes (APC) and average annual percentage change (AAPC).^13^^,^^14^ APC serves to quantify year-to-year variations, while AAPC provides an average trend over a defined period.

Joinpoint regression^15^ analysis was employed to identify significant turning points in temporal trends, differentiating shifts from random variability. Joinpoint regression analysis was performed to examine temporal trends in glaucoma burden from 1990 to 2021. We used an R function based on Joinpoint methodology with a log-linear model (model = "ln"), assuming non-constant variance for more accurate estimation. The maximum number of joinpoints was set to 5, with model selection based on permutation tests at a significance level of *α* = 0.05. The APC was calculated as (e^∧^*β* – 1) × 100, where *β* is the slope coefficient, and 95% confidence intervals were derived from model standard errors. A trend change was considered statistically significant when the permutation test rejected the null hypothesis at *α* = 0.05. The AAPC was calculated as a weighted average of segment-specific APCs. Additionally, the strength and direction of the linear association between glaucoma indicators and SDI were characterized by Pearson correlation analysis.

Frontier analysis established benchmarks for glaucoma burden by constructing a frontier from the best-performing entities,^11^ thereby empirically deriving targets for under-performing regions based on observed best practices. Using bootstrap Data Envelopment Analysis with 1000 resamples, we estimated the theoretical minimum achievable age-standardized rate (the "frontier") for each SDI level. We calculated the effective difference (observed age-standardized rate (ASR) - frontier ASR) for each country. Positive values indicate excess burden and room for improvement. All differences were ≥0, so no countries were excluded from the final frontier estimation. The frontier curve was fitted using LOESS (locally estimated scatterplot smoothing) with span = 0.3 and polynomial degree = 2.

## 3 Results

### 3.1 Global trends

Globally, glaucoma cases rose by 86.33%, from nearly 4.07 million in 1990 to 7.59 million in 2021. Conversely, the age-standardized prevalence rate decreased substantially, with an AAPC of −0.82 (from 116.29 per 100000 population in 1990 to 90.12 per 100000 population in 2021) in this period. Global glaucoma DALYs increased by 62.51% in absolute numbers, while their ASRs decreased, with an AAPC of −1.24 (from 13.37 per 100000 population in 1990 to 9.05 per 100000 population in 2021) (Table 1).

**Table 1:** The prevalence and DALYs of glaucoma and their AAPCs from 1990 to 2021 at the global levels.

	Prevalence						DALYs					
	Cases (n), 1990	Prevalence (per 100000 population), 1990	Cases (n), 2021	Prevalence (per 100000 population), 2021	AAPC, 1990–2021	*P* value	Cases (n), 1990	DALYs (per 100000 population), 1990	Cases (n), 2021	DALYs (per 100000 population), 2021	AAPC, 1990–2021	*P* value
**Global (Age standardized)**												
	4072106.53 (3489888.71, 4752867.32)	116.29 (100.81, 136.30)	7587672.87 (6522905.98, 8917725.43)	90.12 (77.78, 105.49)	−0.82 (−0.85, −0.8)	<0.001	467600.40 (323490.51, 648641.64)	13.37 (9.36, 18.50)	759900.23 (530942.90, 1049127.17)	9.05 (6.29, 12.46)	−1.24 (−1.26, −1.21)	<0.001
**Sex (Age standardized)**												
Male	2083507.47 (1780536.35, 2426202.85)	140.69 (122.15, 165.38)	3831748.99 (3294975.62, 4493441.56)	103.13 (89.44, 120.25)	−1 (−1.03, −0.97)	<0.001	247392.20 (170492.48, 342971.34)	16.76 (11.73, 23.29)	397160.52 (276907.35, 547567.50)	10.78 (7.44, 14.86)	−1.39 (−1.44, −1.34)	<0.001
Female	1988599.06 (1703340.70, 2325425.06)	99.36 (86.02, 116.45)	3755923.88 (3231082.59, 4410200.11)	80.13 (68.86, 94.07)	−0.7 (−0.73, −0.67)	<0.001	220208.21 (152245.10, 306497.49)	11.02 (7.68, 15.22)	362739.71 (252122.30, 492781.15)	7.74 (5.37, 10.52)	−1.12 (−1.14, −1.1)	<0.001
**Age group, years**												
45 to 49	10068.54 (6498.83, 14752.64)	4.34 (2.80, 6.35)	16347.06 (10246.32, 23702.11)	3.45 (2.16, 5.01)	−0.75 (−0.83 to −0.67)	<0.001	1328.65 (641.33, 2457.23)	0.57 (0.28, 1.06)	1847.68 (944.63, 3322.38)	0.39 (0.20, 0.70)	−1.24 (−1.32 to −1.16)	<0.001
50 to 54	89700.53 (65677.42, 120645.35)	42.20 (30.90, 56.76)	146355.36 (106788.94, 199569.89)	32.89 (24.00, 44.85)	−0.8 (−0.92 to −0.68)	<0.001	10846.54 (6602.65, 17702.04)	5.10 (3.11, 8.33)	15249.62 (9344.21, 24415.59)	3.43 (2.10, 5.49)	−1.25 (−1.34 to −1.16)	<0.001
55 to 59	271402.40 (209655.39, 348930.27)	146.55 (113.20, 188.41)	453677.36 (342756.96, 588508.40)	114.64 (86.61, 148.72)	−0.79 (−0.86 to −0.73)	<0.001	31205.74 (20309.06, 46574.45)	16.85 (10.97, 25.15)	43932.68 (29107.00, 65726.86)	11.10 (7.36, 16.61)	−1.32 (−1.4 to −1.25)	<0.001
60 to 64	506607.14 (396018.67, 647850.93)	315.43 (246.57, 403.37)	826534.78 (650036.24, 1065071.16)	258.25 (203.11, 332.78)	−0.64 (−0.69 to −0.59)	<0.001	57223.86 (37936.83, 86073.02)	35.63 (23.62, 53.59)	78446.61 (52744.76, 116478.04)	24.51 (16.48, 36.39)	−1.18 (−1.26 to −1.1)	<0.001
65 to 69	664835.23 (524943.94, 845517.75)	537.85 (424.68, 684.02)	1176670.64 (923095.05, 1516851.54)	426.57 (334.65, 549.90)	−0.74 (−0.85 to −0.64)	<0.001	75015.80 (50113.53, 110703.77)	60.69 (40.54, 89.56)	111552.79 (75450.44, 161829.20)	40.44 (27.35, 58.67)	−1.29 (−1.37 to −1.22)	<0.001
70 to 74	708762.68 (551040.17, 898589.27)	837.18 (650.88, 1061.40)	1299913.46 (1015276.74, 1656860.54)	631.52 (493.24, 804.93)	−0.91 (−1.02 to −0.8)	<0.001	80576.59 (51638.80, 117198.43)	95.18 (60.99, 138.43)	126936.94 (81923.81, 180623.28)	61.67 (39.80, 87.75)	−1.38 (−1.42 to −1.33)	<0.001
75 to 79	726226.95 (580441.06, 893326.54)	1179.79 (942.96, 1451.25)	1222008.32 (977452.25, 1497441.25)	926.58 (741.14, 1135.42)	−0.78 (−0.84 to −0.73)	<0.001	84051.06 (55072.63, 122800.22)	136.55 (89.47, 199.50)	124086.89 (82951.63, 179297.65)	94.09 (62.90, 135.95)	−1.19 (−1.24 to −1.14)	<0.001
80 to 84	586861.51 (462674.26, 734593.17)	1658.93 (1307.88, 2076.53)	1113397.24 (875433.43, 1396801.36)	1271.25 (999.55, 1594.83)	−0.85 (−0.93 to −0.77)	<0.001	68480.70 (44667.42, 98476.12)	193.58 (126.27, 278.37)	116542.43 (76148.12, 165820.54)	133.07 (86.94, 189.33)	−1.18 (−1.23 to −1.13)	<0.001
85 to 89	344677.77 (277912.62, 426003.72)	2280.96 (1839.13, 2819.15)	786033.63 (635035.90, 965828.53)	1719.17 (1388.91, 2112.40)	−0.91 (−0.94 to −0.88)	<0.001	40086.69 (26579.72, 56432.65)	265.28 (175.90, 373.45)	83249.18 (55261.71, 116071.56)	182.08 (120.87, 253.86)	−1.2 (−1.24 to −1.17)	<0.001
90 to 94	124556.47 (99456.35, 153431.75)	2906.67 (2320.93, 3580.51)	387419.75 (307253.91, 472945.50)	2165.64 (1717.52, 2643.72)	−0.94 (−0.98 to −0.91)	<0.001	14368.06 (9764.30, 20263.05)	335.30 (227.86, 472.86)	41015.05 (27913.01, 58723.91)	229.27 (156.03, 328.26)	−1.21 (−1.25 to −1.16)	<0.001
95 plus	38407.31 (30445.03, 48288.18)	3772.50 (2990.42, 4743.03)	159315.27 (126464.86, 198673.48)	2923.05 (2320.32, 3645.18)	−0.82 (−0.86 to −0.78)	<0.001	4416.70 (2867.57, 6572.74)	433.82 (281.66, 645.60)	17040.35 (11327.45, 25075.68)	312.65 (207.83, 460.08)	−1.05 (−1.1 to −0.99)	<0.001

Data in parentheses are 95% uncertainty intervals for cases, prevalence, and DALYs, and 95% CIs for AAPCs. DALYs, disability-adjusted life-years; AAPC, average annual percentage change; CIs, confidence intervals.

Globally, the most pronounced reduction of glaucoma prevalence occurred between 2019 and 2021, with an APC of −2.32. The joinpoint regression analysis identified significant change in the rate of change in prevalence and DALYs of glaucoma in 1994, 2001, 2006, 2010, and 2019 (Fig. 1).

**Fig. 1:**
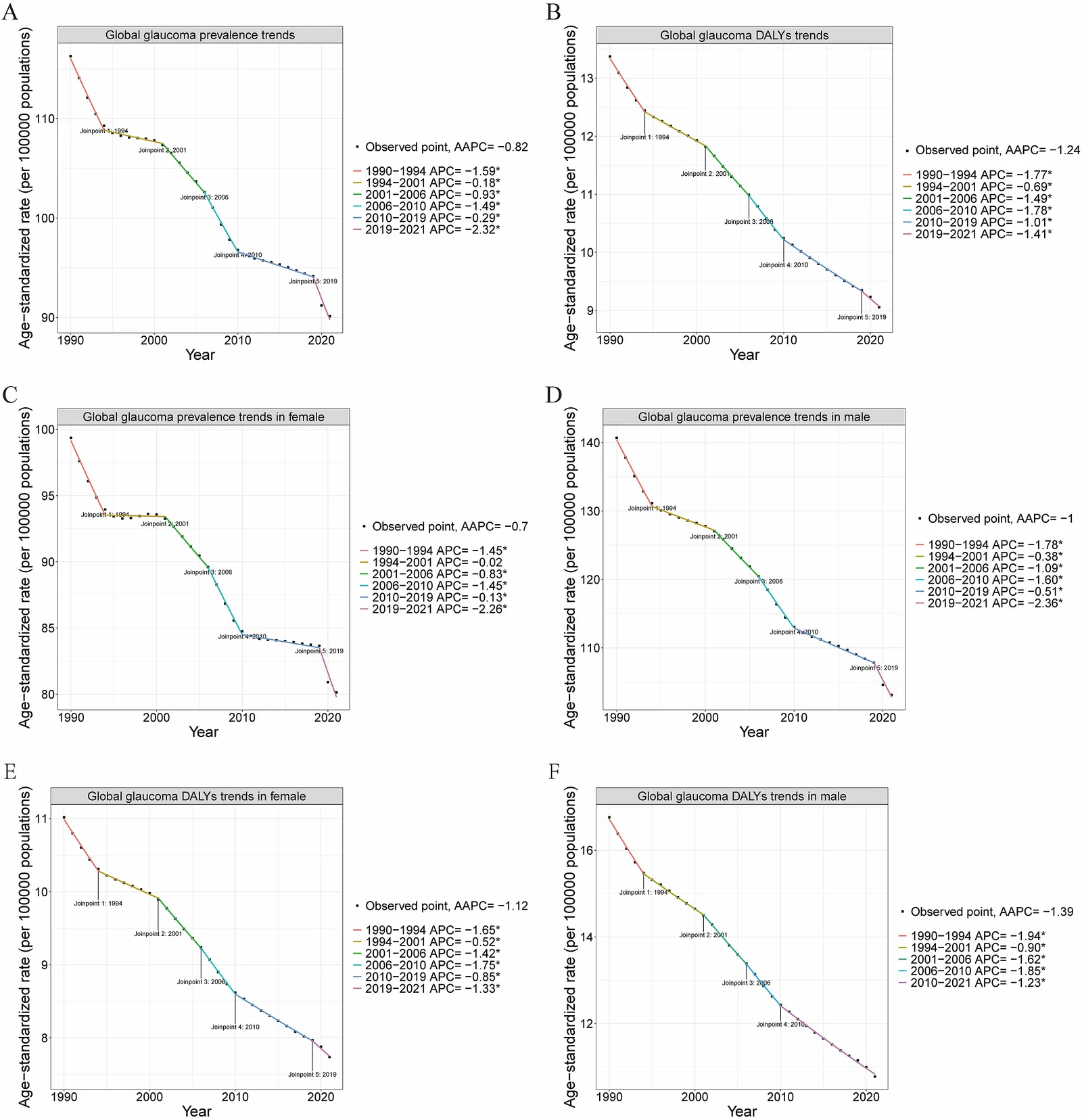
Joinpoint regression analysis of global glaucoma prevalence (A, C, D) and DALYs (B, E, F) from 1990 to 2021. DALYs, disability-adjusted life-years; APC, annual percentage change; AAPC, average annual percentage change.

### 3.2 Global trends by sex

There was global decline in prevalence of glaucoma from 1990 to 2021 in both males and females, with an AAPC of −1.0 (from 140.69 per 100000 population in 1990 to 103.13 per 100000 population in 2021) in males and an AAPC of −0.7 (from 99.36 per 100000 population in 1990 to 80.13 per 100000 population in 2021) in females (Table 1, Fig. 1). The trend in glaucoma prevalence exhibited a similar downward trajectory for both sexes, with the most rapid decline observed between 2019 and 2021 (males: APC −2.36; females: APC −2.26) (Supplementary Table 1).

There was global decline in DALYs of glaucoma from 1990 to 2021 in both males and females, with an AAPC of −1.39 (from 16.76 per 100000 population in 1990 to 10.78 per 100000 population in 2021) in males and an AAPC of −1.12 (from 11.02 per 100000 population in 1990 to 7.74 per 100000 population in 2021) in females (Table 1, Fig. 1). The period of slowest decline for both sexes was between 1994 and 2001, with APCs of −0.90 for males and −0.52 for females (Supplementary Table 1).

### 3.3 Global trends by age group

By age group, the largest decline in global glaucoma prevalence from 1990 to 2021 occurred among individuals aged 90–94 years, decreasing from 2906.67 per 100000 population in 1990 to 2165.64 per 100000 population in 2021 (AAPC −0.94). In contrast, the smallest decline was seen in those aged 60–64 years, falling from 315.43 per 100000 population in 1990 to 258.25 per 100000 population in 2021 (AAPC −0.64) (Table 1).

For DALYs, the greatest reduction occurred in the 70–74 age group, dropping from 95.18 per 100000 population in 1990 to 61.67 per 100000 population in 2021 (AAPC −1.38). The smallest decline in individuals aged 95 years and older, falling from 433.82 per 100000 population to 312.65 per 100000 population (AAPC −1.05) (Table 1).

### 3.4 Regional trends

From 1990 to 2021, the largest declines in glaucoma prevalence occurred in East Asia, North Africa and the Middle East, and South Asia, with AAPCs of −1.67, −1.14, and −1.12, respectively. Conversely, the smallest declines were seen in High-income North America, Central Sub-Saharan Africa, and Australasia, with AAPCs of −0.2, −0.27, and −0.49, respectively (Table 2).

**Table 2:** The prevalence and DALYs of glaucoma and their AAPCs from 1990 to 2021 at the regional levels.

	Prevalence						DALYs					
	Cases (n), 1990	Prevalence (per 100000 population), 1990	Cases (n), 2021	Prevalence (per 100000 population), 2021	AAPC, 1990–2021	*P* value	Cases (n), 1990	DALYs (per 100000 population), 1990	Cases (n), 2021	DALYs (per 100000 population), 2021	AAPC, 1990–2021	*P* value
**SociodemographicIndex (Age standardized)**												
High SDI	646582.09 (556759.58, 753570.30)	57.81 (49.95, 66.99)	1191263.22 (1031211.87, 1391418.48)	49.14 (42.60, 57.10)	−0.53 (−0.56, −0.51)	<0.001	73447.28 (50972.52, 102462.54)	6.56 (4.56, 9.14)	122280.63 (84500.29, 168350.62)	5.06 (3.48, 6.94)	−0.84 (−0.88, −0.8)	<0.001
High-middle SDI	889767.33 (762658.62, 1047424.07)	102.76 (88.55, 120.48)	1400629.46 (1205785.41, 1634878.89)	70.96 (61.19, 82.76)	−1.2 (−1.27, −1.13)	<0.001	107662.53 (74187.30, 150379.72)	12.44 (8.64, 17.25)	144364.35 (100345.58, 200371.70)	7.34 (5.11, 10.21)	−1.7 (−1.82, −1.58)	<0.001
Middle SDI	1213358.32 (1024437.86, 1414660.59)	149.20 (128.36, 176.34)	2611142.08 (2238156.28, 3074011.65)	105.50 (90.86, 123.59)	−1.1 (−1.22, −0.99)	<0.001	138628.18 (95850.58, 192075.86)	17.18 (11.94, 23.51)	253108.68 (175631.99, 349084.97)	10.32 (7.14, 14.15)	−1.6 (−1.67, −1.53)	<0.001
Low-middle SDI	897512.46 (755752.36, 1059114.53)	183.97 (156.47, 216.86)	1617523.21 (1366423.43, 1913844.26)	129.19 (109.78, 153.22)	−1.12 (−1.22, −1.03)	<0.001	97128.81 (66673.31, 134740.11)	20.20 (13.82, 27.74)	155452.05 (107278.97, 214804.76)	12.62 (8.65, 17.14)	−1.5 (−1.64, −1.36)	<0.001
Low SDI	421146.75 (355337.71, 495268.90)	244.96 (209.59, 287.82)	760939.17 (643085.64, 898413.67)	190.53 (162.56, 225.56)	−0.8 (−0.89, −0.7)	<0.001	50297.61 (34483.21, 70476.36)	29.57 (20.42, 40.72)	84046.63 (57426.56, 116740.24)	21.39 (14.63, 29.42)	−1.01 (−1.09, −0.94)	<0.001
**Region (Age standardized)**												
Andean Latin America	34609.95 (28878.69, 42124.08)	193.90 (161.81, 237.33)	77362.99 (64579.86, 93681.44)	137.33 (114.79, 165.99)	−1.11 (−1.16, −1.06)	<0.001	4100.79 (2719.24, 5804.22)	23.09 (15.32, 32.57)	7876.08 (5347.84, 10947.56)	14.02 (9.51, 19.52)	−1.59 (−1.66, −1.53)	<0.001
Australasia	13344.73 (11385.34, 15772.88)	58.36 (49.98, 68.43)	30385.94 (25548.36, 35983.78)	50.14 (42.14, 59.33)	−0.49 (−0.51, −0.46)	<0.001	1430.87 (983.44, 1984.61)	6.25 (4.29, 8.66)	2963.17 (1992.83, 4091.62)	4.92 (3.32, 6.82)	−0.77 (−0.81, −0.72)	<0.001
Caribbean	42425.91 (35190.26, 51048.99)	174.24 (145.97, 209.09)	70581.53 (59152.78, 84587.11)	130.01 (109.03, 156.08)	−0.94 (−0.95, −0.92)	<0.001	5168.66 (3372.84, 7356.77)	21.28 (13.97, 30.22)	7636.14 (5079.32, 10662.82)	14.05 (9.33, 19.59)	−1.33 (−1.35, −1.31)	<0.001
Central Asia	55141.11 (46280.08, 66828.91)	131.96 (111.09, 160.19)	72637.58 (60052.68, 88160.68)	105.87 (88.90, 126.07)	−0.71 (−0.73, −0.69)	<0.001	6543.67 (4420.20, 9406.30)	15.67 (10.51, 22.59)	7852.55 (5368.62, 11114.34)	11.50 (7.74, 16.32)	−0.99 (−1.01, −0.97)	<0.001
Central Europe	67897.43 (57304.12, 80428.59)	49.25 (41.87, 58.22)	92636.62 (78598.76, 110131.42)	38.62 (32.88, 45.72)	−0.78 (−0.8, −0.76)	<0.001	7042.88 (4787.94, 10004.57)	5.13 (3.51, 7.27)	8485.88 (5857.72, 11709.18)	3.54 (2.43, 4.87)	−1.19 (−1.23, −1.15)	<0.001
Central Latin America	118504.34 (100711.83, 139490.14)	168.53 (143.88, 199.97)	299310.49 (255750.40, 353855.95)	125.96 (107.35, 148.92)	−0.94 (−0.95, −0.92)	<0.001	12451.99 (8457.08, 17227.89)	17.82 (12.09, 24.46)	26760.20 (18736.52, 35588.11)	11.30 (7.90, 15.08)	−1.47 (−1.49, −1.44)	<0.001
Central Sub-Saharan Africa	25439.08 (21147.49, 30717.59)	174.84 (146.91, 209.44)	57793.07 (48292.57, 68716.25)	161.11 (135.57, 191.41)	−0.27 (−0.29, −0.25)	<0.001	2441.18 (1640.66, 3468.38)	17.19 (11.67, 23.98)	5088.05 (3475.42, 7136.47)	14.51 (10.01, 19.90)	−0.55 (−0.58, −0.52)	<0.001
East Asia	602900.47 (503678.83, 711879.04)	88.32 (75.07, 105.05)	1137135.42 (961986.83, 1349351.32)	53.75 (45.71, 63.40)	−1.67 (−1.92, −1.42)	<0.001	69522.83 (47858.85, 96449.63)	10.13 (6.94, 14.04)	100817.46 (68255.02, 140368.41)	4.77 (3.25, 6.65)	−2.44 (−2.79, −2.09)	<0.001
Eastern Europe	226898.02 (195997.23, 266370.90)	86.87 (75.74, 102.65)	252879.06 (218326.14, 297491.57)	69.27 (59.90, 81.40)	−0.73 (−0.76, −0.71)	<0.001	25309.81 (17401.29, 35024.52)	9.72 (6.72, 13.41)	24980.55 (17285.12, 33832.22)	6.85 (4.75, 9.28)	−1.11 (−1.14, −1.09)	<0.001
Eastern Sub-Saharan Africa	166898.40 (140868.85, 194357.12)	293.01 (250.92, 342.30)	308159.22 (260514.27, 363309.15)	236.71 (201.91, 279.55)	−0.69 (−0.72, −0.65)	<0.001	21277.35 (14704.98, 29910.37)	37.42 (26.25, 51.55)	36478.04 (25402.66, 50902.44)	28.32 (19.75, 38.97)	−0.89 (−0.93, −0.86)	<0.001
High-income Asia Pacific	124151.51 (108382.00, 143458.67)	67.18 (59.05, 77.30)	323089.91 (283086.39, 376459.47)	54.84 (47.83, 62.78)	−0.66 (−0.69, −0.62)	<0.001	15016.98 (10465.19, 21003.86)	8.01 (5.56, 11.03)	34518.38 (23784.17, 47294.21)	6.01 (4.13, 8.22)	−0.92 (−0.96, −0.88)	<0.001
High-income North America	172910.80 (150019.47, 201313.81)	46.47 (40.42, 53.87)	317228.12 (274946.98, 368049.72)	43.63 (37.66, 50.64)	−0.2 (−0.27, −0.14)	<0.001	18852.45 (13108.07, 25820.10)	5.07 (3.53, 6.95)	33183.02 (23298.73, 45594.52)	4.57 (3.18, 6.28)	−0.34 (−0.4, −0.27)	<0.001
North Africa and Middle East	402591.34 (339256.18, 477781.16)	299.08 (254.87, 357.81)	776896.79 (655149.18, 918337.99)	208.16 (176.62, 247.23)	−1.14 (−1.24, −1.05)	<0.001	51076.46 (35176.73, 71550.22)	38.01 (26.18, 52.96)	85940.38 (58782.00, 120136.80)	23.29 (15.89, 32.25)	−1.54 (−1.66, −1.43)	<0.001
Oceania	2371.47 (1901.08, 2901.88)	112.39 (92.40, 136.11)	5139.03 (4231.28, 6183.62)	92.25 (76.33, 110.00)	−0.64 (−0.7, −0.59)	<0.001	296.47 (194.00, 429.30)	13.93 (9.19, 19.57)	594.53 (393.79, 843.20)	10.66 (7.00, 15.11)	−0.85 (−0.98, −0.73)	<0.001
South Asia	803203.72 (669772.64, 948045.39)	175.54 (148.54, 207.57)	1621629.52 (1354035.57, 1917154.90)	123.19 (104.38, 145.59)	−1.12 (−1.28, −0.96)	<0.001	85478.27 (59379.94, 119547.58)	18.95 (13.19, 26.09)	152206.26 (106464.50, 210344.48)	11.76 (8.21, 16.13)	−1.52 (−1.77, −1.28)	<0.001
Southeast Asia	211833.93 (176766.21, 251299.42)	104.51 (87.71, 124.43)	422596.02 (354981.93, 499060.78)	74.55 (62.78, 87.99)	−1.08 (−1.11, −1.05)	<0.001	22716.83 (15370.82, 31616.85)	11.37 (7.75, 15.72)	38784.99 (26670.09, 53895.89)	6.95 (4.73, 9.48)	−1.58 (−1.63, −1.53)	<0.001
Southern Latin America	34689.31 (29150.00, 41989.68)	81.10 (68.46, 98.90)	58174.52 (48817.72, 68864.02)	63.51 (53.33, 75.01)	−0.78 (−0.81, −0.75)	<0.001	3675.65 (2438.26, 5162.60)	8.61 (5.76, 12.15)	5406.57 (3653.13, 7493.53)	5.90 (4.00, 8.17)	−1.21 (−1.25, −1.17)	<0.001
Southern Sub-Saharan Africa	53767.58 (45628.63, 64046.98)	236.34 (202.11, 282.79)	96003.88 (81545.88, 113008.80)	202.23 (171.94, 240.57)	−0.48 (−0.62, −0.35)	<0.001	6904.16 (4739.44, 9735.32)	30.19 (20.74, 42.07)	11337.86 (7812.50, 15779.34)	23.84 (16.51, 32.96)	−0.74 (−0.9, −0.58)	<0.001
Tropical Latin America	163390.75 (140039.43, 189585.86)	215.72 (185.11, 252.12)	420428.93 (360786.53, 492901.04)	169.78 (145.33, 199.28)	−0.82 (−1.08, −0.55)	<0.001	16693.77 (11585.55, 22896.57)	22.32 (15.45, 30.41)	38739.39 (26855.38, 52310.14)	15.72 (10.85, 21.14)	−1.11 (−1.16, −1.05)	<0.001
Western Europe	493873.23 (416915.36, 583523.67)	81.48 (69.16, 95.72)	695423.67 (592196.49, 812825.10)	60.97 (51.94, 70.83)	−0.94 (−0.97, −0.91)	<0.001	63124.27 (43340.04, 89173.45)	10.43 (7.20, 14.64)	81622.92 (56401.25, 114984.51)	7.25 (5.01, 10.15)	−1.18 (−1.2, −1.15)	<0.001
Western Sub-Saharan Africa	255263.44 (216161.31, 295046.22)	358.15 (306.95, 418.73)	452180.56 (383396.62, 527663.57)	294.35 (250.83, 345.34)	−0.63 (−0.67, −0.59)	<0.001	28475.06 (19862.43, 39633.92)	40.84 (28.39, 55.97)	48627.81 (33570.45, 67247.83)	32.48 (22.35, 44.53)	−0.73 (−0.82, −0.64)	<0.001

Data in parentheses are 95% uncertainty intervals for cases, prevalence, and DALYs, and 95% CIs for AAPCs. DALYs, disability-adjusted life-years; AAPC, average annual percentage change; SDI, Sociodemographic Index; CIs, confidence intervals.

For glaucoma DALYs, the most significant decreases were in East Asia, Andean Latin America, and Southeast Asia, with AAPCs of −2.44, −1.59, and −1.58, respectively. Meanwhile, the lowest declines were in High-income North America, Central Sub-Saharan Africa, Western Sub-Saharan Africa, and Australasia, with AAPCs of −0.34, −0.55, −0.73, and −0.77, respectively (Table 2).

### 3.5 National trends

From 1990 to 2021, Tunisia saw the largest decline in glaucoma prevalence (AAPC −1.91), dropping from 330.29 to 182.43 per 100000 population. Conversely, Côte d'Ivoire had the largest increase (AAPC 1.83), rising from 154.29 to 271.43 per 100000 population. In 2021, Niger had the highest prevalence at 345.65 per 100000 population (Supplementary Table 2).

For DALYs, the Maldives recorded the largest decrease (AAPC −2.61), falling from 15.70 to 6.93 per 100000 population, while Côte d'Ivoire saw the largest increase (AAPC 1.51), rising from 18.42 to 29.13 per 100000 population. Niger also had the highest DALYs in 2021 at 41.83 per 100000 population (Supplementary Table 3).

To investigate whether the sharp global decline in glaucoma prevalence during 2019–2021 represented a genuine burden reduction or a pandemic-related data artefact, we examined the 10 countries with the largest negative APC during this period based on GBD 2021 joinpoint regression results for 204 countries. These countries were: China (APC = −13.09), Qatar (APC = −8.54), Uganda (APC = −6.45), Sudan (APC = −5.89), Nepal (APC = −4.31), Fiji (APC = −4.17), Timor-Leste (APC = −4.04), Libya (APC = −3.13), Afghanistan (APC = −2.81), and Brazil (APC = −1.95). Using GBD 2023 data (1990–2023) for these 10 countries, we compared APC estimates for the pandemic period. Of the 10 countries, 7 (70%) showed either substantial deceleration or stabilization of the decline when data through 2023 were included (Supplementary Table 4, 5). This pattern supports the interpretation that the 2019–2021 decline was predominantly a pandemic-related data artefact rather than a genuine reduction in disease burden.

### 3.6 Global trends by SDI

From 1990 to 2021, the high-middle SDI quintile experienced the most significant declines in glaucoma prevalence and DALYs, with AAPCs of −1.2 and −1.7, respectively. In contrast, the high SDI quintile experienced the smallest reductions, with AAPCs of −0.53 for prevalence and −0.84 for DALYs. In 2021, the high SDI quintile had the lowest prevalence (49.14 per 100000 population), while the low SDI quintile had the highest (190.53 per 100000 population) (Table 2).

### 3.7 Correlations of ASR with SDI

Investigating the prevalence and DALYs of glaucoma globally and across 21 regions, a nuanced correlation with the SDI emerged. A strong negative correlation was observed between SDI and prevalence ASR (*r* = −0.59, *P* < 0.001). A similarly inverse association was noted for DALYs (*r* = −0.55, *P* < 0.001) (Supplementary Fig.1).

### 3.8 Frontier analysis

In the frontier analysis of glaucoma ASRs and SDI across 204 countries from 1990 to 2021, clear trends emerged. As SDI increased from 0.0 to 1.0, glaucoma prevalence and DALYs both decreased overall, indicating that higher development levels are associated with lower glaucoma burdens (Fig. 2A–C).

**Fig. 2:**
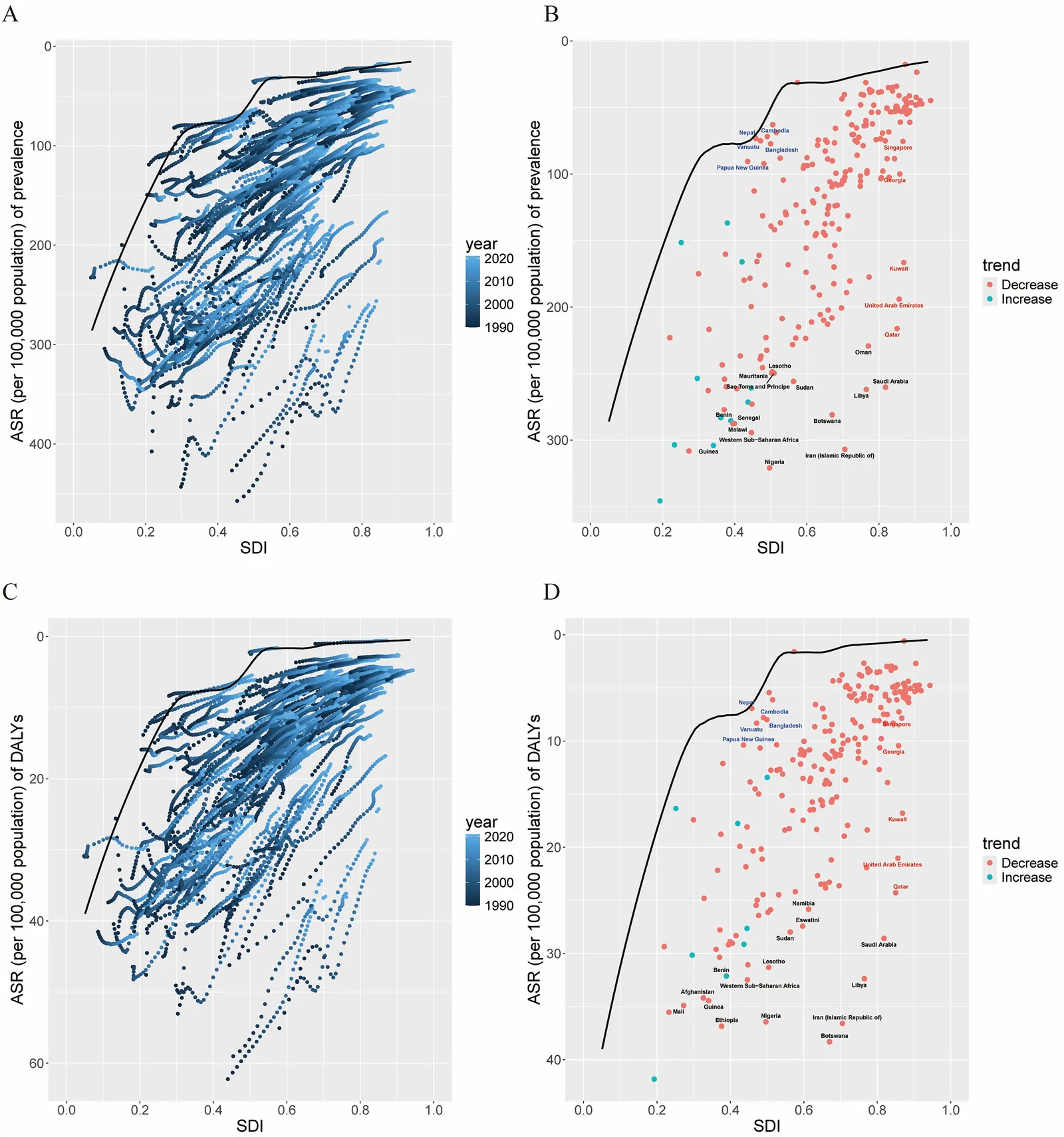
Frontier analysis, represented by the solid black lines, explores the relationship between SDI and ASR for prevalence (A, B) and DALYs (C, D) in the context of glaucoma. SDI, Sociodemographic Index; ASR, age-standardized rate; DALYs, disability-adjusted life-years.

Frontier analysis revealed substantial heterogeneity in country-level performance. Countries or regions such as the Democratic People's Republic of Korea and Taiwan (Province of China) had effective differences close to zero (0.03 and 0.22), indicating optimal outcomes relative to their SDI levels. In contrast, Iran (279.35), Nigeria (254.60), and Botswana (249.77) exhibited the largest effective differences for prevalence, while Botswana (36.67), Iran (35.53), and Nigeria (30.69) showed the largest differences for DALYs, suggesting substantial excess burden that could be addressed through targeted interventions. Notably, even within low-SDI countries, substantial heterogeneity existed; for instance, Nepal achieved near-frontier performance (effective difference = 0.40), while others faced moderate to high excess burden (Fig. 2B–D).

## 4 Discussion

This study provides a comprehensive analysis of global, regional, and national trends in glaucoma burden among adults aged 45 years and older from 1990 to 2021. Consistent with previous GBD-based studies, we found a paradoxical pattern: despite an 86.33% increase in absolute case numbers due to population aging and growth, age-standardized prevalence declined from 116.29 to 90.12 per 100000 population (AAPC = −0.82), and DALYs decreased from 13.37 to 9.05 per 100000 population (AAPC = −1.24). This pattern—declining age-standardized rates alongside rising absolute burden—reflects the dual impact of improved disease management and demographic aging. For a given age, glaucoma burden has decreased over time. Advances in screening, diagnosis, and treatment—including medications, laser therapy, and surgery—have contributed to better intraocular pressure control and delayed disease progression.^16^, ^17^, ^18^ However, because glaucoma risk increases exponentially with age, the absolute number of older adults has outpaced these per-age improvements, leading to rising total cases.

Our study extends previous GBD-based glaucoma analyses in several ways. Although several studies have reported global glaucoma trends using earlier GBD iterations,^16^^,^^19^^,^^20^ our analysis offers unique contributions by incorporating the most recent publicly available data (GBD 2021) supplemented with partial GBD 2023 estimates. First, we capture the sharp decline during 2019–2021 and, using GBD 2023 data for validation, interpret it as a pandemic-related data artefact. Second, unlike existing joinpoint analyses that focused on all-age populations,^21^ we provide detailed age-stratified (5-year age groups from 45–49 to 95+) and sex-stratified joinpoint analyses, revealing heterogeneity masked in aggregate analyses. Third, compared with a recent study on adults aged 45+ that did not apply joinpoint regression,^22^ our identification of five statistically significant inflection points (1994, 2001, 2006, 2010, and 2019) enables temporal alignment of trend changes with clinical developments. Finally, our frontier analysis provides country-level benchmarks with specific policy targets.

Joinpoint regression identified significant trend changes in 1994, 2001, 2006, 2010, and 2019. The accelerated decline around 2001 temporally coincides with the global expansion of prostaglandin analogues, particularly latanoprost. Approved by the FDA in 1996, latanoprost effectively lowers intraocular pressure by increasing uveoscleral outflow,^23^ and its once-daily dosing and minimal side effects^2^ established it as a first-line treatment for POAG.^24^ Considering the typical lag between the introduction of a new therapy and its widespread population-level impact, the widespread adoption of such effective treatments may have been a contributing factor to the accelerated decline observed around 2001. However, given the ecological nature of GBD data, we propose this as a plausible hypothesis rather than a causal conclusion.

The sharp decline between 2019 and 2021 requires cautious interpretation. Rather than representing a true reduction in disease burden, this decline most likely reflects under-ascertainment of cases due to pandemic-related healthcare disruptions, including reduced outpatient visits and deferred screenings.^25^^,^^26^ Fewer visits led to fewer newly diagnosed captured in GBD data sources, artificially lowering prevalence estimates and manifesting as a sharp negative APC. To validate this, we examined the 10 countries with the sharpest declines using GBD 2023 data; 7 of 10 (70%) showed substantial deceleration or stabilization when data through 2023 were included (Supplementary Table 4, 5). Once healthcare services resumed, previously undiagnosed cases began to be captured, explaining the observed deceleration. This suggests the pandemic-related decline was primarily a data artefact. More broadly, sharp declines in disease estimates during health emergencies should be interpreted with caution, as they may reflect diagnostic disruptions rather than genuine epidemiological improvements.

Age-specific analysis revealed that the highest glaucoma prevalence in 2021 was among individuals aged 95 years and older—more than 800-fold higher than those aged 45–49 years. This underscores the need for age-tailored screening strategies, with particular focus on the oldest-old population. The persistent sex disparity, with males consistently showing higher burden, likely reflects the predominance of open-angle glaucoma in men.

Regional analysis showed that sub-Saharan Africa bore a disproportionately high burden in 2021, while Central Europe had substantially lower rates, highlighting the influence of socioeconomic factors^27^ on disease epidemiology. East Asia's remarkable decline in both prevalence and DALYs (AAPC = −1.67 and −2.44) suggests that, alongside genetic susceptibility, healthcare access and environmental factors play important roles in shaping regional disparities.^16^^,^^27^^,^^28^

At the national level, there remain 11 countries where the prevalence of glaucoma is increasing, including Côte d'Ivoire, Burkina Faso, Benin, The Gambia, Niger, Chad, Togo, the Democratic Republic of the Congo, the Central African Republic, Guinea, and Guinea-Bissau. It seems that Low SDI countries experience disproportionate impact from glaucoma. It is imperative that future efforts to provide glaucoma prevention assistance and guidance to these nations be further reinforced.

Pearson correlation analysis revealed strong inverse associations between SDI and both prevalence (*r* = −0.59, *P* < 0.001) and DALYs (*r* = −0.55, *P* < 0.001), consistent with previous studies.^29^^,^^30^ Frontier analysis provided pivotal insights into country-level performance relative to development status. While SDI showed a strong inverse correlation with burden, substantial heterogeneity existed among countries at similar SDI levels. Some low-SDI countries achieved near-frontier performance. Conversely, several middle- and high-SDI countries exhibited excess burden relative to their development level. These findings confirm that glaucoma epidemiology is not monochromatic; while SDI exerts significant influence, it is modulated by a constellation of latent variables—from environmental and genetic susceptibilities to the architecture of local healthcare systems. For instance, Taiwan (Province of China) achieved near-frontier performance (effective difference = 0.22), likely reflecting its advanced universal health insurance system, high urbanization, and widespread access to ophthalmic care. Additionally, a study of the Amis aboriginal population in Taiwan (Province of China) reported an exceptionally low glaucoma prevalence (1.0%),^31^ suggesting that unique genetic protective factors may also contribute to the low observed burden in this population. Therefore, integrating SDI with additional data sources—such as per capita GDP, educational attainment, healthcare system efficiency, and genetic susceptibility—will help further elucidate the deep mechanisms underlying regional disparities in glaucoma burden.

This study has several limitations. Our data are sourced exclusively from the GBD study, which may not fully capture local variations. Data quality and consistency vary across regions, potentially introducing bias. Temporal changes in diagnostic criteria and medical technologies over the 32-year study period may affect trend comparability. Additionally, the minimum age restriction of 45 years in GBD glaucoma models means our findings do not apply to younger populations with congenital, juvenile, or early-onset glaucoma subtypes. Furthermore, the GBD study assigns all POAG, angle-closure glaucoma, and secondary glaucoma to a single entity. This aggregation may mask distinct epidemiological patterns, risk factor profiles, and temporal trends among different glaucoma subtypes. Although GBD 2023 estimates have been released, access to the global-level data is restricted by IHME policies for non-Collaborator researchers. Consequently, our findings do not capture trends after 2021. Should these data become publicly accessible for independent global research in the future, updated analyses will be warranted. Finally, this study did not quantify the independent effects of specific SDI components (e.g., per capita GDP, education, healthcare resources) or explore interactions among genetic, environmental, and lifestyle factors. Despite these limitations, this study provides valuable insights into global glaucoma epidemiology and identifies priority areas for targeted interventions.

## 5 Conclusions

From 1990 to 2021, global age-standardized prevalence and DALYs for glaucoma showed an overall declining trend, with periods of accelerated decline that temporally coincided with key therapeutic advances, including the widespread adoption of prostaglandin analogues. However, due to population aging and growth, the absolute number of individuals affected by glaucoma continued to increase, indicating that the global burden remains substantial. These findings underscore the need for targeted screening programs and public awareness campaigns emphasizing the importance of regular eye examinations for early detection, particularly among elderly populations who bear the highest risk.

## Study approval

Not Applicable.

## Author contributions

The authors confirm contribution to the paper as follows: Conceptualization, Methodology, Visualization, Writing – original draft: CW, FC; Data curation, Investigation: CW, FC, BC, XL, SZ, YW, XC, HF; Formal analysis: CW, FC, BC, XL, SZ, YW; Project administration, Supervision : NW, SH; Writing – review and editing: BC, XL, SZ, YW, XC, HF, NW, SH. All authors read and approved the final manuscript.

## Funding

This work was supported by the National Natural Science Foundation Project of China (82271078); the Project of Youth Beijing Scholar (No.076); Beijing Municipal Public Welfare Development and Reform Pilot Project for Medical Research Institutes, Research Project on the Regulation of the Immune Microenvironment in Major Blinding Eye Diseases; Beijing Institute of Ophthalmology Research Project 2024: Immunoregulation in Retinal and Optic Nerve Diseases (PWD&RPP-MRI, JYY2023-6).

## Declaration of competing interest

The authors declare that they have no known competing financial interests or personal relationships that could have appeared to influence the work reported in this paper.
