# Supplementary material for: Global, regional, and national trends in glaucoma burden among adults aged 45 years and older, 1990-2021
Source: Adv Ophthalmol Pract Res. 2026 Jun 20;6(4):363–72. doi: 10.1016/j.aopr.2026.06.004 (PMC13743481; doi:10.1016/j.aopr.2026.06.004)
Supplement: Supplementary file 1 [file mmc1.docx]

Supplementary Material

Content

[Supplementary Table 1	2](#_Toc20977)

[Supplementary Table 2	79](#_Toc5296)

[Supplementary Table 3	94](#_Toc24076)

[Supplementary Table 4	112](#_Toc17626)

[Supplementary Table 5	385](#_Toc8381)

[Supplementary Fig.1	387](#_Toc4450)

## Supplementary Table 1

| Supplementary Table1 Joinpoint regression analysis of prevalence and DALYs of glaucoma from 1990 to 2021 at the global and regional level, stratified by sex. | | | | | | | |
| --- | --- | --- | --- | --- | --- | --- | --- |
| measure | location | sex | cause | Segment.Start | Segment.End | P.Value | APC_95CI |
| DALYs | Global | Both | Glaucoma | 1990 | 1994 | 0 | -1.77 (-1.83, -1.70) |
| DALYs | Global | Both | Glaucoma | 1994 | 2001 | 0 | -0.69 (-0.72, -0.65) |
| DALYs | Global | Both | Glaucoma | 2001 | 2006 | 0 | -1.49 (-1.55, -1.43) |
| DALYs | Global | Both | Glaucoma | 2006 | 2010 | 0 | -1.78 (-1.88, -1.68) |
| DALYs | Global | Both | Glaucoma | 2010 | 2019 | 0 | -1.01 (-1.03, -0.98) |
| DALYs | Global | Both | Glaucoma | 2019 | 2021 | 0 | -1.41 (-1.61, -1.21) |
| DALYs | Global | Female | Glaucoma | 1990 | 1994 | 0 | -1.65 (-1.71, -1.59) |
| DALYs | Global | Female | Glaucoma | 1994 | 2001 | 0 | -0.52 (-0.56, -0.49) |
| DALYs | Global | Female | Glaucoma | 2001 | 2006 | 0 | -1.42 (-1.48, -1.36) |
| DALYs | Global | Female | Glaucoma | 2006 | 2010 | 0 | -1.75 (-1.85, -1.66) |
| DALYs | Global | Female | Glaucoma | 2010 | 2019 | 0 | -0.85 (-0.88, -0.83) |
| DALYs | Global | Female | Glaucoma | 2019 | 2021 |  | -1.33 (-1.52, -1.14) |
| DALYs | Global | Male | Glaucoma | 1990 | 1994 | 0 | -1.94 (-2.11, -1.77) |
| DALYs | Global | Male | Glaucoma | 1994 | 2001 | 0 | -0.90 (-0.99, -0.81) |
| DALYs | Global | Male | Glaucoma | 2001 | 2006 | 0 | -1.62 (-1.79, -1.45) |
| DALYs | Global | Male | Glaucoma | 2006 | 2010 | 0 | -1.85 (-2.12, -1.59) |
| DALYs | Global | Male | Glaucoma | 2010 | 2021 | 0 | -1.23 (-1.27, -1.19) |
| DALYs | Andean Latin America | Both | Glaucoma | 1990 | 1995 | 0 | -1.36  (-1.48, -1.24) |
| DALYs | Andean Latin America | Both | Glaucoma | 1995 | 2000 | 0 | -2.17  (-2.35, -2.05) |
| DALYs | Andean Latin America | Both | Glaucoma | 2000 | 2018 | 0 | -1.62  (-1.64, -1.6) |
| DALYs | Andean Latin America | Both | Glaucoma | 2018 | 2021 | 0 | -0.82  (-1.05, -0.43) |
| DALYs | Andean Latin America | Female | Glaucoma | 1990 | 1995 | 0 | -1.24  (-1.37, -1.08) |
| DALYs | Andean Latin America | Female | Glaucoma | 1995 | 2000 | 0 | -2.21  (-2.47, -2.07) |
| DALYs | Andean Latin America | Female | Glaucoma | 2000 | 2017 | 0 | -1.69  (-1.72, -1.67) |
| DALYs | Andean Latin America | Female | Glaucoma | 2017 | 2021 | 0 | -1.07  (-1.25, -0.8) |
| DALYs | Andean Latin America | Male | Glaucoma | 1990 | 1995 | 0 | -1.46  (-1.53, -1.39) |
| DALYs | Andean Latin America | Male | Glaucoma | 1995 | 2000 | 0 | -2.16  (-2.26, -2.1) |
| DALYs | Andean Latin America | Male | Glaucoma | 2000 | 2005 | 0 | -1.49  (-1.55, -1.35) |
| DALYs | Andean Latin America | Male | Glaucoma | 2005 | 2009 | 0 | -1.75  (-1.87, -1.66) |
| DALYs | Andean Latin America | Male | Glaucoma | 2009 | 2019 | 0 | -1.47  (-1.5, -1.43) |
| DALYs | Andean Latin America | Male | Glaucoma | 2019 | 2021 | 0 | -0.42  (-0.61, -0.28) |
| DALYs | Australasia | Both | Glaucoma | 1990 | 1997 | 0 | -1.46  (-1.57, -1.42) |
| DALYs | Australasia | Both | Glaucoma | 1997 | 2000 | 0.01119776 | -1.24  (-1.36, -1.14) |
| DALYs | Australasia | Both | Glaucoma | 2000 | 2006 | 0.074785043 | -0.05  (-0.13, 0.01) |
| DALYs | Australasia | Both | Glaucoma | 2006 | 2011 | 0.00959808 | -0.59  (-0.65, -0.26) |
| DALYs | Australasia | Both | Glaucoma | 2011 | 2018 | 0 | -0.74  (-0.89, -0.7) |
| DALYs | Australasia | Both | Glaucoma | 2018 | 2021 | 0 | -0.44  (-0.59, -0.24) |
| DALYs | Australasia | Female | Glaucoma | 1990 | 2000 | 0 | -1.4  (-1.44, -1.36) |
| DALYs | Australasia | Female | Glaucoma | 2000 | 2005 | 0.087982404 | 0.1  (-0.02, 0.18) |
| DALYs | Australasia | Female | Glaucoma | 2005 | 2021 | 0 | -0.65  (-0.68, -0.63) |
| DALYs | Australasia | Male | Glaucoma | 1990 | 1997 | 0 | -1.53  (-1.72, -1.46) |
| DALYs | Australasia | Male | Glaucoma | 1997 | 2000 | 0.019196161 | -1.28  (-1.42, -0.33) |
| DALYs | Australasia | Male | Glaucoma | 2000 | 2006 | 0.092381524 | -0.12  (-0.21, 0.07) |
| DALYs | Australasia | Male | Glaucoma | 2006 | 2010 | 0 | -0.46  (-0.71, -0.29) |
| DALYs | Australasia | Male | Glaucoma | 2010 | 2017 | 0 | -0.9  (-1.08, -0.83) |
| DALYs | Australasia | Male | Glaucoma | 2017 | 2021 | 0.025594881 | -0.38  (-0.53, -0.13) |
| DALYs | Caribbean | Both | Glaucoma | 1990 | 1993 | 0 | -1.72  (-1.87, -1.62) |
| DALYs | Caribbean | Both | Glaucoma | 1993 | 2004 | 0 | -1.5  (-1.52, -1.48) |
| DALYs | Caribbean | Both | Glaucoma | 2004 | 2009 | 0 | -1.27  (-1.35, -1.19) |
| DALYs | Caribbean | Both | Glaucoma | 2009 | 2015 | 0 | -1.04  (-1.08, -0.95) |
| DALYs | Caribbean | Both | Glaucoma | 2015 | 2021 | 0 | -1.15  (-1.22, -1.12) |
| DALYs | Caribbean | Female | Glaucoma | 1990 | 1994 | 0 | -1.65  (-1.8, -1.58) |
| DALYs | Caribbean | Female | Glaucoma | 1994 | 2005 | 0 | -1.45  (-1.47, -1.42) |
| DALYs | Caribbean | Female | Glaucoma | 2005 | 2010 | 0 | -1.3  (-1.38, -1.18) |
| DALYs | Caribbean | Female | Glaucoma | 2010 | 2015 | 0 | -0.94  (-0.98, -0.85) |
| DALYs | Caribbean | Female | Glaucoma | 2015 | 2018 | 0 | -1.36  (-1.43, -1.25) |
| DALYs | Caribbean | Female | Glaucoma | 2018 | 2021 | 0 | -0.75  (-0.84, -0.64) |
| DALYs | Caribbean | Male | Glaucoma | 1990 | 1992 | 0 | -1.77  (-1.85, -1.64) |
| DALYs | Caribbean | Male | Glaucoma | 1992 | 2003 | 0 | -1.5  (-1.52, -1.49) |
| DALYs | Caribbean | Male | Glaucoma | 2003 | 2007 | 0 | -1.29  (-1.38, -1.2) |
| DALYs | Caribbean | Male | Glaucoma | 2007 | 2019 | 0 | -1.08  (-1.09, -1.07) |
| DALYs | Caribbean | Male | Glaucoma | 2019 | 2021 | 0 | -1.43  (-1.52, -1.3) |
| DALYs | Central Asia | Both | Glaucoma | 1990 | 1994 | 0 | -0.66  (-0.83, -0.57) |
| DALYs | Central Asia | Both | Glaucoma | 1994 | 2001 | 0.00079984 | -0.16  (-0.2, -0.11) |
| DALYs | Central Asia | Both | Glaucoma | 2001 | 2006 | 0.00079984 | -1.4  (-1.45, -1.32) |
| DALYs | Central Asia | Both | Glaucoma | 2006 | 2014 | 0 | -1.58  (-1.68, -1.55) |
| DALYs | Central Asia | Both | Glaucoma | 2014 | 2018 | 0 | -1.24  (-1.42, -1.1) |
| DALYs | Central Asia | Both | Glaucoma | 2018 | 2021 | 0 | -0.78  (-0.92, -0.56) |
| DALYs | Central Asia | Female | Glaucoma | 1990 | 1994 | 0 | -0.62  (-0.83, -0.5) |
| DALYs | Central Asia | Female | Glaucoma | 1994 | 2001 | 0.00039992 | -0.19  (-0.24, -0.13) |
| DALYs | Central Asia | Female | Glaucoma | 2001 | 2015 | 0 | -1.52  (-1.54, -1.5) |
| DALYs | Central Asia | Female | Glaucoma | 2015 | 2021 | 0 | -1  (-1.06, -0.9) |
| DALYs | Central Asia | Male | Glaucoma | 1990 | 1994 | 0 | -0.65  (-0.83, -0.54) |
| DALYs | Central Asia | Male | Glaucoma | 1994 | 2001 | 0 | -0.23  (-0.28, -0.17) |
| DALYs | Central Asia | Male | Glaucoma | 2001 | 2006 | 0 | -1.51  (-1.57, -1.43) |
| DALYs | Central Asia | Male | Glaucoma | 2006 | 2012 | 0 | -1.83  (-1.98, -1.77) |
| DALYs | Central Asia | Male | Glaucoma | 2012 | 2018 | 0 | -1.54  (-1.6, -1.44) |
| DALYs | Central Asia | Male | Glaucoma | 2018 | 2021 | 0 | -0.62  (-0.75, -0.49) |
| DALYs | Central Europe | Both | Glaucoma | 1990 | 1994 | 0 | -1.57  (-1.68, -1.49) |
| DALYs | Central Europe | Both | Glaucoma | 1994 | 2001 | 0 | -1.19  (-1.22, -1.15) |
| DALYs | Central Europe | Both | Glaucoma | 2001 | 2004 | 0 | -1.71  (-1.77, -1.58) |
| DALYs | Central Europe | Both | Glaucoma | 2004 | 2010 | 0 | -1.42  (-1.45, -1.36) |
| DALYs | Central Europe | Both | Glaucoma | 2010 | 2018 | 0 | -0.95  (-0.97, -0.92) |
| DALYs | Central Europe | Both | Glaucoma | 2018 | 2021 | 0.00239952 | -0.33  (-0.41, -0.26) |
| DALYs | Central Europe | Female | Glaucoma | 1990 | 1994 | 0 | -1.88  (-2.1, -1.74) |
| DALYs | Central Europe | Female | Glaucoma | 1994 | 2001 | 0 | -1.07  (-1.14, -0.98) |
| DALYs | Central Europe | Female | Glaucoma | 2001 | 2010 | 0 | -1.57  (-1.64, -1.52) |
| DALYs | Central Europe | Female | Glaucoma | 2010 | 2019 | 0 | -1.18  (-1.23, -1.13) |
| DALYs | Central Europe | Female | Glaucoma | 2019 | 2021 | 0.01159768 | 0.35  (0.1, 0.55) |
| DALYs | Central Europe | Male | Glaucoma | 1990 | 1996 | 0 | -1.2  (-1.26, -1.08) |
| DALYs | Central Europe | Male | Glaucoma | 1996 | 2001 | 0 | -1.38  (-1.64, -1.3) |
| DALYs | Central Europe | Male | Glaucoma | 2001 | 2004 | 0 | -1.77  (-1.86, -1.37) |
| DALYs | Central Europe | Male | Glaucoma | 2004 | 2009 | 0 | -1.34  (-1.45, -0.98) |
| DALYs | Central Europe | Male | Glaucoma | 2009 | 2014 | 0 | -0.91  (-1.05, -0.69) |
| DALYs | Central Europe | Male | Glaucoma | 2014 | 2021 | 0 | -0.61  (-0.66, -0.52) |
| DALYs | Central Latin America | Both | Glaucoma | 1990 | 1996 | 0 | -1.92  (-1.97, -1.85) |
| DALYs | Central Latin America | Both | Glaucoma | 1996 | 2000 | 0 | -2.16  (-2.26, -2.09) |
| DALYs | Central Latin America | Both | Glaucoma | 2000 | 2009 | 0 | -1.44  (-1.48, -1.41) |
| DALYs | Central Latin America | Both | Glaucoma | 2009 | 2019 | 0 | -1.1  (-1.13, -1.07) |
| DALYs | Central Latin America | Both | Glaucoma | 2019 | 2021 | 0 | -0.61  (-0.82, -0.48) |
| DALYs | Central Latin America | Female | Glaucoma | 1990 | 1995 | 0 | -1.68  (-1.76, -1.6) |
| DALYs | Central Latin America | Female | Glaucoma | 1995 | 2000 | 0 | -2.14  (-2.27, -2.07) |
| DALYs | Central Latin America | Female | Glaucoma | 2000 | 2010 | 0 | -1.49  (-1.52, -1.47) |
| DALYs | Central Latin America | Female | Glaucoma | 2010 | 2015 | 0 | -0.94  (-1, -0.78) |
| DALYs | Central Latin America | Female | Glaucoma | 2015 | 2019 | 0 | -1.27  (-1.41, -1.19) |
| DALYs | Central Latin America | Female | Glaucoma | 2019 | 2021 | 0.00319936 | -0.27  (-0.5, -0.1) |
| DALYs | Central Latin America | Male | Glaucoma | 1990 | 2000 | 0 | -2.04  (-2.06, -2.02) |
| DALYs | Central Latin America | Male | Glaucoma | 2000 | 2010 | 0 | -1.33  (-1.36, -1.31) |
| DALYs | Central Latin America | Male | Glaucoma | 2010 | 2019 | 0 | -1.05  (-1.09, -1.02) |
| DALYs | Central Latin America | Male | Glaucoma | 2019 | 2021 | 0 | -0.62  (-0.84, -0.5) |
| DALYs | Central Sub-Saharan Africa | Both | Glaucoma | 1990 | 1995 | 0 | -0.31  (-0.42, -0.23) |
| DALYs | Central Sub-Saharan Africa | Both | Glaucoma | 1995 | 2005 | 0 | 0.15  (0.12, 0.19) |
| DALYs | Central Sub-Saharan Africa | Both | Glaucoma | 2005 | 2011 | 0 | -0.29  (-0.34, -0.23) |
| DALYs | Central Sub-Saharan Africa | Both | Glaucoma | 2011 | 2015 | 0 | -1.8  (-1.93, -1.72) |
| DALYs | Central Sub-Saharan Africa | Both | Glaucoma | 2015 | 2021 | 0 | -1.36  (-1.41, -1.28) |
| DALYs | Central Sub-Saharan Africa | Female | Glaucoma | 1990 | 1995 | 0 | -0.19  (-0.31, -0.11) |
| DALYs | Central Sub-Saharan Africa | Female | Glaucoma | 1995 | 1999 | 0.021195761 | 0.22  (0.04, 0.4) |
| DALYs | Central Sub-Saharan Africa | Female | Glaucoma | 1999 | 2005 | 0.00679864 | 0.6  (0.55, 0.68) |
| DALYs | Central Sub-Saharan Africa | Female | Glaucoma | 2005 | 2011 | 0 | -0.29  (-0.33, -0.24) |
| DALYs | Central Sub-Saharan Africa | Female | Glaucoma | 2011 | 2015 | 0 | -1.5  (-1.61, -1.42) |
| DALYs | Central Sub-Saharan Africa | Female | Glaucoma | 2015 | 2021 | 0 | -1.18  (-1.23, -1.09) |
| DALYs | Central Sub-Saharan Africa | Male | Glaucoma | 1990 | 1995 | 0.00079984 | -0.17  (-0.35, -0.07) |
| DALYs | Central Sub-Saharan Africa | Male | Glaucoma | 1995 | 2000 | 0 | 0.27  (0.16, 0.48) |
| DALYs | Central Sub-Saharan Africa | Male | Glaucoma | 2000 | 2011 | 0 | -0.24  (-0.27, -0.21) |
| DALYs | Central Sub-Saharan Africa | Male | Glaucoma | 2011 | 2015 | 0 | -2.16  (-2.33, -2.06) |
| DALYs | Central Sub-Saharan Africa | Male | Glaucoma | 2015 | 2021 | 0 | -1.57  (-1.65, -1.47) |
| DALYs | East Asia | Both | Glaucoma | 1990 | 1992 | 0 | -11.01  (-12.74, -8.12) |
| DALYs | East Asia | Both | Glaucoma | 1992 | 1995 | 0.100779844 | -5.76  (-6.75, 4.68) |
| DALYs | East Asia | Both | Glaucoma | 1995 | 2000 | 0.058788242 | 5.19  (-2.79, 6.08) |
| DALYs | East Asia | Both | Glaucoma | 2000 | 2007 | 0.009998 | -3.97  (-4.72, -3.2) |
| DALYs | East Asia | Both | Glaucoma | 2007 | 2019 | 0.00359928 | -1.05  (-1.27, -0.69) |
| DALYs | East Asia | Both | Glaucoma | 2019 | 2021 | 0 | -9.41  (-11.36, -6.72) |
| DALYs | East Asia | Female | Glaucoma | 1990 | 1992 | 0 | -9.84  (-11.89, -6.25) |
| DALYs | East Asia | Female | Glaucoma | 1992 | 1995 | 0.25354929 | -4.75  (-5.67, 6.09) |
| DALYs | East Asia | Female | Glaucoma | 1995 | 2000 | 0.156368726 | 5.4  (-3.82, 7.36) |
| DALYs | East Asia | Female | Glaucoma | 2000 | 2008 | 0.031993601 | -3.51  (-4.58, -1.32) |
| DALYs | East Asia | Female | Glaucoma | 2008 | 2019 | 0.031193761 | -0.94  (-1.26, -0.19) |
| DALYs | East Asia | Female | Glaucoma | 2019 | 2021 | 0 | -8.64  (-11.06, -6.05) |
| DALYs | East Asia | Male | Glaucoma | 1990 | 1992 | 0 | -12.19  (-13.86, -10.21) |
| DALYs | East Asia | Male | Glaucoma | 1992 | 1995 | 0.041591682 | -6.82  (-7.81, -2.56) |
| DALYs | East Asia | Male | Glaucoma | 1995 | 2000 | 0.022395521 | 4.97  (4.29, 5.71) |
| DALYs | East Asia | Male | Glaucoma | 2000 | 2006 | 0.00319936 | -4.69  (-5.51, -4.11) |
| DALYs | East Asia | Male | Glaucoma | 2006 | 2019 | 0.00079984 | -1.16  (-1.35, -0.9) |
| DALYs | East Asia | Male | Glaucoma | 2019 | 2021 | 0 | -10.21  (-11.93, -7.12) |
| DALYs | Eastern Europe | Both | Glaucoma | 1990 | 1994 | 0 | -0.87  (-1.12, -0.73) |
| DALYs | Eastern Europe | Both | Glaucoma | 1994 | 2001 | 0.109578084 | -0.05  (-0.1, 0.03) |
| DALYs | Eastern Europe | Both | Glaucoma | 2001 | 2009 | 0 | -2.09  (-2.15, -2.05) |
| DALYs | Eastern Europe | Both | Glaucoma | 2009 | 2019 | 0 | -1.37  (-1.41, -1.32) |
| DALYs | Eastern Europe | Both | Glaucoma | 2019 | 2021 | 0.213957209 | -0.13  (-0.46, 0.07) |
| DALYs | Eastern Europe | Female | Glaucoma | 1990 | 1994 | 0 | -0.86  (-1.24, -0.64) |
| DALYs | Eastern Europe | Female | Glaucoma | 1994 | 2001 | 0.0079984 | -0.23  (-0.31, -0.11) |
| DALYs | Eastern Europe | Female | Glaucoma | 2001 | 2008 | 0 | -2.3  (-2.42, -2.23) |
| DALYs | Eastern Europe | Female | Glaucoma | 2008 | 2018 | 0 | -1.5  (-1.56, -1.44) |
| DALYs | Eastern Europe | Female | Glaucoma | 2018 | 2021 | 0.058788242 | -0.46  (-0.76, 0.04) |
| DALYs | Eastern Europe | Male | Glaucoma | 1990 | 1994 | 0 | -0.93  (-1.08, -0.8) |
| DALYs | Eastern Europe | Male | Glaucoma | 1994 | 2001 | 0.01039792 | 0.11  (0.05, 0.18) |
| DALYs | Eastern Europe | Male | Glaucoma | 2001 | 2005 | 0.013197361 | -1.72  (-1.82, -1.55) |
| DALYs | Eastern Europe | Male | Glaucoma | 2005 | 2010 | 0 | -2.05  (-2.28, -1.95) |
| DALYs | Eastern Europe | Male | Glaucoma | 2010 | 2019 | 0 | -1.35  (-1.41, -1.3) |
| DALYs | Eastern Europe | Male | Glaucoma | 2019 | 2021 | 0.00039992 | -0.36  (-0.71, -0.16) |
| DALYs | Eastern Sub-Saharan Africa | Both | Glaucoma | 1990 | 1995 | 0 | -0.6  (-0.73, -0.47) |
| DALYs | Eastern Sub-Saharan Africa | Both | Glaucoma | 1995 | 2000 | 0 | -2.45  (-2.55, -2.33) |
| DALYs | Eastern Sub-Saharan Africa | Both | Glaucoma | 2000 | 2005 | 0.400319936 | 0.04  (-0.13, 0.16) |
| DALYs | Eastern Sub-Saharan Africa | Both | Glaucoma | 2005 | 2015 | 0 | -1.13  (-1.19, -1.08) |
| DALYs | Eastern Sub-Saharan Africa | Both | Glaucoma | 2015 | 2019 | 0 | 0.71  (0.56, 0.87) |
| DALYs | Eastern Sub-Saharan Africa | Both | Glaucoma | 2019 | 2021 | 0 | -2.03  (-2.46, -1.65) |
| DALYs | Eastern Sub-Saharan Africa | Female | Glaucoma | 1990 | 1995 | 0 | -0.69  (-0.85, -0.53) |
| DALYs | Eastern Sub-Saharan Africa | Female | Glaucoma | 1995 | 2000 | 0 | -2.58  (-2.71, -2.44) |
| DALYs | Eastern Sub-Saharan Africa | Female | Glaucoma | 2000 | 2005 | 0.057188562 | 0.18  (-0.02, 0.31) |
| DALYs | Eastern Sub-Saharan Africa | Female | Glaucoma | 2005 | 2015 | 0 | -1.26  (-1.32, -1.2) |
| DALYs | Eastern Sub-Saharan Africa | Female | Glaucoma | 2015 | 2019 | 0 | 1.14  (0.97, 1.31) |
| DALYs | Eastern Sub-Saharan Africa | Female | Glaucoma | 2019 | 2021 | 0 | -2.05  (-2.52, -1.62) |
| DALYs | Eastern Sub-Saharan Africa | Male | Glaucoma | 1990 | 1996 | 0 | -0.66  (-0.75, -0.55) |
| DALYs | Eastern Sub-Saharan Africa | Male | Glaucoma | 1996 | 1999 | 0 | -3.03  (-3.2, -2.87) |
| DALYs | Eastern Sub-Saharan Africa | Male | Glaucoma | 1999 | 2006 | 0 | -0.26  (-0.36, -0.16) |
| DALYs | Eastern Sub-Saharan Africa | Male | Glaucoma | 2006 | 2015 | 0 | -1.01  (-1.09, -0.94) |
| DALYs | Eastern Sub-Saharan Africa | Male | Glaucoma | 2015 | 2019 | 0.00079984 | 0.36  (0.18, 0.64) |
| DALYs | Eastern Sub-Saharan Africa | Male | Glaucoma | 2019 | 2021 | 0 | -1.91  (-2.32, -1.49) |
| DALYs | High SDI | Both | Glaucoma | 1990 | 1994 | 0 | -1.77  (-1.85, -1.68) |
| DALYs | High SDI | Both | Glaucoma | 1994 | 2010 | 0 | -0.98  (-1, -0.96) |
| DALYs | High SDI | Both | Glaucoma | 2010 | 2014 | 0 | -0.46  (-0.67, -0.26) |
| DALYs | High SDI | Both | Glaucoma | 2014 | 2019 | 0.851029794 | -0.02  (-0.09, 0.15) |
| DALYs | High SDI | Both | Glaucoma | 2019 | 2021 | 0 | -0.64  (-0.86, -0.38) |
| DALYs | High SDI | Female | Glaucoma | 1990 | 1994 | 0 | -1.69  (-1.83, -1.6) |
| DALYs | High SDI | Female | Glaucoma | 1994 | 2006 | 0 | -1.13  (-1.15, -1.1) |
| DALYs | High SDI | Female | Glaucoma | 2006 | 2009 | 0 | -1.39  (-1.47, -1.24) |
| DALYs | High SDI | Female | Glaucoma | 2009 | 2013 | 0 | -0.77  (-0.9, -0.62) |
| DALYs | High SDI | Female | Glaucoma | 2013 | 2019 | 0.00039992 | -0.23  (-0.27, -0.12) |
| DALYs | High SDI | Female | Glaucoma | 2019 | 2021 | 0 | -0.65  (-0.82, -0.43) |
| DALYs | High SDI | Male | Glaucoma | 1990 | 1994 | 0 | -1.87  (-2.05, -1.78) |
| DALYs | High SDI | Male | Glaucoma | 1994 | 1997 | 0 | -1.05  (-1.54, -0.91) |
| DALYs | High SDI | Male | Glaucoma | 1997 | 2010 | 0 | -0.89  (-0.91, -0.79) |
| DALYs | High SDI | Male | Glaucoma | 2010 | 2015 | 0 | -0.42  (-0.53, -0.28) |
| DALYs | High SDI | Male | Glaucoma | 2015 | 2019 | 0.01119776 | 0.12  (0.04, 0.28) |
| DALYs | High SDI | Male | Glaucoma | 2019 | 2021 | 0 | -0.74  (-0.95, -0.46) |
| DALYs | High-income Asia Pacific | Both | Glaucoma | 1990 | 1994 | 0 | -2.17  (-2.3, -2.1) |
| DALYs | High-income Asia Pacific | Both | Glaucoma | 1994 | 1999 | 0 | -1.7  (-1.81, -1.62) |
| DALYs | High-income Asia Pacific | Both | Glaucoma | 1999 | 2002 | 0 | -1.23  (-1.55, -1.04) |
| DALYs | High-income Asia Pacific | Both | Glaucoma | 2002 | 2008 | 0 | -1.01  (-1.06, -0.49) |
| DALYs | High-income Asia Pacific | Both | Glaucoma | 2008 | 2011 | 0.023595281 | -0.46  (-0.74, -0.05) |
| DALYs | High-income Asia Pacific | Both | Glaucoma | 2011 | 2021 | 0.108778244 | -0.02  (-0.05, 0.01) |
| DALYs | High-income Asia Pacific | Female | Glaucoma | 1990 | 1997 | 0 | -2.05  (-2.18, -2) |
| DALYs | High-income Asia Pacific | Female | Glaucoma | 1997 | 2000 | 0 | -1.68  (-1.98, -1.12) |
| DALYs | High-income Asia Pacific | Female | Glaucoma | 2000 | 2009 | 0 | -0.99  (-1.14, -0.92) |
| DALYs | High-income Asia Pacific | Female | Glaucoma | 2009 | 2012 | 0.014397121 | -0.44  (-0.99, -0.13) |
| DALYs | High-income Asia Pacific | Female | Glaucoma | 2012 | 2019 | 0.49510098 | 0.02  (-0.26, 0.09) |
| DALYs | High-income Asia Pacific | Female | Glaucoma | 2019 | 2021 | 0 | 0.46  (0.14, 0.61) |
| DALYs | High-income Asia Pacific | Male | Glaucoma | 1990 | 1995 | 0 | -2.11  (-2.27, -2.02) |
| DALYs | High-income Asia Pacific | Male | Glaucoma | 1995 | 2006 | 0 | -1.37  (-1.41, -1.33) |
| DALYs | High-income Asia Pacific | Male | Glaucoma | 2006 | 2010 | 0 | -0.75  (-1.22, -0.52) |
| DALYs | High-income Asia Pacific | Male | Glaucoma | 2010 | 2018 | 0.025594881 | -0.2  (-0.26, -0.05) |
| DALYs | High-income Asia Pacific | Male | Glaucoma | 2018 | 2021 | 0 | -0.52  (-0.81, -0.31) |
| DALYs | High-income North America | Both | Glaucoma | 1990 | 1999 | 0 | -1.18  (-1.23, -1.13) |
| DALYs | High-income North America | Both | Glaucoma | 1999 | 2005 | 0 | -0.49  (-0.58, -0.32) |
| DALYs | High-income North America | Both | Glaucoma | 2005 | 2010 | 0 | -0.91  (-1.05, -0.81) |
| DALYs | High-income North America | Both | Glaucoma | 2010 | 2015 | 0 | 0.59  (0.46, 0.68) |
| DALYs | High-income North America | Both | Glaucoma | 2015 | 2018 | 0 | 1.6  (1.38, 1.75) |
| DALYs | High-income North America | Both | Glaucoma | 2018 | 2021 | 0.903819236 | 0.01  (-0.19, 0.19) |
| DALYs | High-income North America | Female | Glaucoma | 1990 | 2000 | 0 | -1.16  (-1.23, -1.11) |
| DALYs | High-income North America | Female | Glaucoma | 2000 | 2005 | 0 | -0.75  (-0.87, -0.51) |
| DALYs | High-income North America | Female | Glaucoma | 2005 | 2010 | 0 | -1.16  (-1.32, -1.05) |
| DALYs | High-income North America | Female | Glaucoma | 2010 | 2015 | 0.013997201 | 0.58  (0.36, 0.66) |
| DALYs | High-income North America | Female | Glaucoma | 2015 | 2018 | 0 | 1.17  (0.9, 1.32) |
| DALYs | High-income North America | Female | Glaucoma | 2018 | 2021 | 0.020795841 | -0.25  (-0.5, -0.05) |
| DALYs | High-income North America | Male | Glaucoma | 1990 | 1998 | 0 | -1.28  (-1.37, -1.21) |
| DALYs | High-income North America | Male | Glaucoma | 1998 | 2006 | 0.00319936 | -0.43  (-0.5, -0.28) |
| DALYs | High-income North America | Male | Glaucoma | 2006 | 2010 | 0 | -0.91  (-1.15, -0.71) |
| DALYs | High-income North America | Male | Glaucoma | 2010 | 2015 | 0 | 0.41  (0.23, 0.55) |
| DALYs | High-income North America | Male | Glaucoma | 2015 | 2018 | 0 | 1.91  (1.58, 2.11) |
| DALYs | High-income North America | Male | Glaucoma | 2018 | 2021 | 0.171965607 | 0.18  (-0.13, 0.41) |
| DALYs | High-middle SDI | Both | Glaucoma | 1990 | 1992 | 0 | -3.94  (-4.4, -3.41) |
| DALYs | High-middle SDI | Both | Glaucoma | 1992 | 1995 | 0.021195761 | -2.53  (-2.78, -1.58) |
| DALYs | High-middle SDI | Both | Glaucoma | 1995 | 2000 | 0.026794641 | 0.25  (0.1, 0.41) |
| DALYs | High-middle SDI | Both | Glaucoma | 2000 | 2009 | 0 | -2.16  (-2.25, -2.08) |
| DALYs | High-middle SDI | Both | Glaucoma | 2009 | 2019 | 0 | -1.14  (-1.21, -1.05) |
| DALYs | High-middle SDI | Both | Glaucoma | 2019 | 2021 | 0 | -3.74  (-4.16, -3.27) |
| DALYs | High-middle SDI | Female | Glaucoma | 1990 | 1992 | 0 | -3.3  (-3.72, -2.59) |
| DALYs | High-middle SDI | Female | Glaucoma | 1992 | 1995 | 0.089582084 | -2.2  (-2.42, 0.26) |
| DALYs | High-middle SDI | Female | Glaucoma | 1995 | 2000 | 0.097580484 | 0.18  (-2.2, 0.34) |
| DALYs | High-middle SDI | Female | Glaucoma | 2000 | 2009 | 0 | -2.21  (-2.3, -2.01) |
| DALYs | High-middle SDI | Female | Glaucoma | 2009 | 2019 | 0 | -1.17  (-1.24, -1.08) |
| DALYs | High-middle SDI | Female | Glaucoma | 2019 | 2021 | 0 | -3.47  (-3.86, -3) |
| DALYs | High-middle SDI | Male | Glaucoma | 1990 | 1992 | 0 | -4.68  (-5.34, -3.55) |
| DALYs | High-middle SDI | Male | Glaucoma | 1992 | 1995 | 0.131173765 | -3.04  (-3.37, 0.41) |
| DALYs | High-middle SDI | Male | Glaucoma | 1995 | 2000 | 0.196360728 | 0.22  (-2.34, 0.6) |
| DALYs | High-middle SDI | Male | Glaucoma | 2000 | 2008 | 0 | -2.27  (-2.48, -1.49) |
| DALYs | High-middle SDI | Male | Glaucoma | 2008 | 2019 | 0 | -1.26  (-1.34, -1.11) |
| DALYs | High-middle SDI | Male | Glaucoma | 2019 | 2021 | 0 | -3.8  (-4.5, -2.84) |
| DALYs | Low SDI | Both | Glaucoma | 1990 | 1995 | 0.388722256 | -0.05  (-0.19, 0.1) |
| DALYs | Low SDI | Both | Glaucoma | 1995 | 2000 | 0.025194961 | -1.9  (-2.06, -0.78) |
| DALYs | Low SDI | Both | Glaucoma | 2000 | 2005 | 0.00479904 | -0.59  (-1.85, -0.36) |
| DALYs | Low SDI | Both | Glaucoma | 2005 | 2014 | 0 | -1.52  (-1.62, -0.59) |
| DALYs | Low SDI | Both | Glaucoma | 2014 | 2019 | 0 | -0.62  (-1.57, -0.3) |
| DALYs | Low SDI | Both | Glaucoma | 2019 | 2021 | 0 | -1.27  (-1.64, -0.74) |
| DALYs | Low SDI | Female | Glaucoma | 1990 | 1996 | 0.00039992 | -0.47  (-0.57, -0.35) |
| DALYs | Low SDI | Female | Glaucoma | 1996 | 2000 | 0 | -2.04  (-2.31, -1.82) |
| DALYs | Low SDI | Female | Glaucoma | 2000 | 2005 | 0.017996401 | -0.46  (-0.65, -0.25) |
| DALYs | Low SDI | Female | Glaucoma | 2005 | 2014 | 0 | -1.53  (-1.6, -1.46) |
| DALYs | Low SDI | Female | Glaucoma | 2014 | 2019 | 0.063187363 | -0.22  (-0.34, 0.05) |
| DALYs | Low SDI | Female | Glaucoma | 2019 | 2021 | 0 | -1.28  (-1.7, -0.82) |
| DALYs | Low SDI | Male | Glaucoma | 1990 | 1995 | 0.220755849 | 0.07  (-0.05, 0.19) |
| DALYs | Low SDI | Male | Glaucoma | 1995 | 2000 | 0 | -1.89  (-2.03, -1.77) |
| DALYs | Low SDI | Male | Glaucoma | 2000 | 2005 | 0.00119976 | -0.61  (-0.77, -0.38) |
| DALYs | Low SDI | Male | Glaucoma | 2005 | 2014 | 0 | -1.46  (-1.55, -1.4) |
| DALYs | Low SDI | Male | Glaucoma | 2014 | 2021 | 0 | -0.93  (-1.02, -0.81) |
| DALYs | Low-middle SDI | Both | Glaucoma | 1990 | 1996 | 0 | -0.45  (-0.57, -0.33) |
| DALYs | Low-middle SDI | Both | Glaucoma | 1996 | 1999 | 0 | -2.52  (-2.7, -2.2) |
| DALYs | Low-middle SDI | Both | Glaucoma | 1999 | 2006 | 0 | -1.58  (-1.66, -1.39) |
| DALYs | Low-middle SDI | Both | Glaucoma | 2006 | 2010 | 0 | -2.54  (-2.84, -2.28) |
| DALYs | Low-middle SDI | Both | Glaucoma | 2010 | 2018 | 0 | -1.75  (-1.83, -1.65) |
| DALYs | Low-middle SDI | Both | Glaucoma | 2018 | 2021 | 0.064387123 | -0.32  (-0.67, 0.03) |
| DALYs | Low-middle SDI | Female | Glaucoma | 1990 | 1996 | 0 | -0.58  (-0.68, -0.47) |
| DALYs | Low-middle SDI | Female | Glaucoma | 1996 | 1999 | 0 | -2.26  (-2.41, -1.95) |
| DALYs | Low-middle SDI | Female | Glaucoma | 1999 | 2006 | 0 | -1.53  (-1.61, -1.37) |
| DALYs | Low-middle SDI | Female | Glaucoma | 2006 | 2010 | 0 | -2.59  (-2.88, -2.36) |
| DALYs | Low-middle SDI | Female | Glaucoma | 2010 | 2018 | 0 | -1.46  (-1.54, -1.39) |
| DALYs | Low-middle SDI | Female | Glaucoma | 2018 | 2021 | 0.088382324 | -0.23  (-0.52, 0.14) |
| DALYs | Low-middle SDI | Male | Glaucoma | 1990 | 1996 | 0.015196961 | -0.33  (-0.47, -0.17) |
| DALYs | Low-middle SDI | Male | Glaucoma | 1996 | 1999 | 0.00319936 | -2.63  (-2.85, -2.18) |
| DALYs | Low-middle SDI | Male | Glaucoma | 1999 | 2006 | 0 | -1.51  (-1.6, -1.24) |
| DALYs | Low-middle SDI | Male | Glaucoma | 2006 | 2009 | 0 | -2.52  (-2.72, -2.11) |
| DALYs | Low-middle SDI | Male | Glaucoma | 2009 | 2018 | 0 | -1.93  (-2.01, -1.76) |
| DALYs | Low-middle SDI | Male | Glaucoma | 2018 | 2021 | 0.110377924 | -0.33  (-0.71, 0.24) |
| DALYs | Middle SDI | Both | Glaucoma | 1990 | 1994 | 0 | -3.04  (-3.24, -2.82) |
| DALYs | Middle SDI | Both | Glaucoma | 1994 | 2001 | 0.00639872 | -0.37  (-0.57, -0.24) |
| DALYs | Middle SDI | Both | Glaucoma | 2001 | 2010 | 0.00479904 | -1.97  (-2.11, -0.31) |
| DALYs | Middle SDI | Both | Glaucoma | 2010 | 2019 | 0 | -1.46  (-2.08, -1.13) |
| DALYs | Middle SDI | Both | Glaucoma | 2019 | 2021 | 0 | -2.14  (-2.61, -1.52) |
| DALYs | Middle SDI | Female | Glaucoma | 1990 | 1994 | 0 | -2.73  (-2.9, -2.57) |
| DALYs | Middle SDI | Female | Glaucoma | 1994 | 2000 | 0.095980804 | -0.1  (-0.2, 0.03) |
| DALYs | Middle SDI | Female | Glaucoma | 2000 | 2005 | 0.00359928 | -1.54  (-1.69, -1.23) |
| DALYs | Middle SDI | Female | Glaucoma | 2005 | 2010 | 0 | -2.18  (-2.51, -2) |
| DALYs | Middle SDI | Female | Glaucoma | 2010 | 2015 | 0 | -1.18  (-1.35, -0.86) |
| DALYs | Middle SDI | Female | Glaucoma | 2015 | 2021 | 0 | -1.63  (-1.88, -1.52) |
| DALYs | Middle SDI | Male | Glaucoma | 1990 | 1994 | 0 | -3.37  (-3.52, -3.22) |
| DALYs | Middle SDI | Male | Glaucoma | 1994 | 2001 | 0 | -0.55  (-0.65, -0.45) |
| DALYs | Middle SDI | Male | Glaucoma | 2001 | 2006 | 0.00039992 | -1.84  (-1.98, -0.66) |
| DALYs | Middle SDI | Male | Glaucoma | 2006 | 2009 | 0 | -2.3  (-2.47, -1.68) |
| DALYs | Middle SDI | Male | Glaucoma | 2009 | 2019 | 0 | -1.53  (-1.58, -1.3) |
| DALYs | Middle SDI | Male | Glaucoma | 2019 | 2021 | 0 | -2.16  (-2.51, -1.69) |
| DALYs | North Africa and Middle East | Both | Glaucoma | 1990 | 2001 | 0 | -1.24  (-1.3, -1.18) |
| DALYs | North Africa and Middle East | Both | Glaucoma | 2001 | 2010 | 0 | -2  (-2.12, -1.93) |
| DALYs | North Africa and Middle East | Both | Glaucoma | 2010 | 2015 | 0 | -1.32  (-1.47, -0.95) |
| DALYs | North Africa and Middle East | Both | Glaucoma | 2015 | 2018 | 0 | -2.19  (-2.41, -1.87) |
| DALYs | North Africa and Middle East | Both | Glaucoma | 2018 | 2021 | 0.00279944 | -0.99  (-1.25, -0.46) |
| DALYs | North Africa and Middle East | Female | Glaucoma | 1990 | 1996 | 0 | -0.99  (-1.12, -0.63) |
| DALYs | North Africa and Middle East | Female | Glaucoma | 1996 | 2001 | 0 | -1.41  (-1.85, -1.21) |
| DALYs | North Africa and Middle East | Female | Glaucoma | 2001 | 2010 | 0 | -2  (-2.17, -1.93) |
| DALYs | North Africa and Middle East | Female | Glaucoma | 2010 | 2015 | 0 | -1.13  (-1.28, -0.79) |
| DALYs | North Africa and Middle East | Female | Glaucoma | 2015 | 2018 | 0 | -2.21  (-2.42, -1.87) |
| DALYs | North Africa and Middle East | Female | Glaucoma | 2018 | 2021 | 0.00439912 | -0.85  (-1.11, -0.32) |
| DALYs | North Africa and Middle East | Male | Glaucoma | 1990 | 1994 | 0 | -1.58  (-2.01, -1.37) |
| DALYs | North Africa and Middle East | Male | Glaucoma | 1994 | 2001 | 0 | -1.14  (-1.24, -0.83) |
| DALYs | North Africa and Middle East | Male | Glaucoma | 2001 | 2010 | 0 | -2.01  (-2.16, -1.93) |
| DALYs | North Africa and Middle East | Male | Glaucoma | 2010 | 2015 | 0 | -1.51  (-1.67, -1.14) |
| DALYs | North Africa and Middle East | Male | Glaucoma | 2015 | 2018 | 0 | -2.26  (-2.47, -1.93) |
| DALYs | North Africa and Middle East | Male | Glaucoma | 2018 | 2021 | 0.00159968 | -1.04  (-1.3, -0.51) |
| DALYs | Oceania | Both | Glaucoma | 1990 | 1996 | 0.00279944 | -1.43  (-2.12, -1.14) |
| DALYs | Oceania | Both | Glaucoma | 1996 | 2010 | 0.020795841 | -0.61  (-1.02, -0.12) |
| DALYs | Oceania | Both | Glaucoma | 2010 | 2015 | 0.00879824 | -1.3  (-1.97, -0.37) |
| DALYs | Oceania | Both | Glaucoma | 2015 | 2018 | 0.615076985 | -0.03  (-1.52, 0.41) |
| DALYs | Oceania | Both | Glaucoma | 2018 | 2021 | 0.00119976 | -1.17  (-2.24, -0.52) |
| DALYs | Oceania | Female | Glaucoma | 1990 | 1995 | 0.0019996 | -1.4  (-3.07, -0.67) |
| DALYs | Oceania | Female | Glaucoma | 1995 | 2010 | 0.174765047 | -0.41  (-0.53, 0.72) |
| DALYs | Oceania | Female | Glaucoma | 2010 | 2014 | 0.022395521 | -1.96  (-3.08, -0.86) |
| DALYs | Oceania | Female | Glaucoma | 2014 | 2021 | 0.035192961 | 0.57  (0.07, 1.77) |
| DALYs | Oceania | Male | Glaucoma | 1990 | 1993 | 0 | -1.72  (-1.89, -1.62) |
| DALYs | Oceania | Male | Glaucoma | 1993 | 1996 | 0 | -1.37  (-1.52, -0.98) |
| DALYs | Oceania | Male | Glaucoma | 1996 | 2001 | 0 | -0.9  (-1, -0.79) |
| DALYs | Oceania | Male | Glaucoma | 2001 | 2012 | 0 | -0.78  (-0.81, -0.73) |
| DALYs | Oceania | Male | Glaucoma | 2012 | 2018 | 0 | -1.15  (-1.2, -1.1) |
| DALYs | Oceania | Male | Glaucoma | 2018 | 2021 | 0 | -1.89  (-1.98, -1.8) |
| DALYs | South Asia | Both | Glaucoma | 1990 | 1996 | 0.072785443 | 0.21  (-0.02, 0.53) |
| DALYs | South Asia | Both | Glaucoma | 1996 | 1999 | 0.0019996 | -3.01  (-3.39, -2.25) |
| DALYs | South Asia | Both | Glaucoma | 1999 | 2005 | 0 | -1.55  (-1.8, -0.85) |
| DALYs | South Asia | Both | Glaucoma | 2005 | 2018 | 0 | -2.59  (-2.69, -2.5) |
| DALYs | South Asia | Both | Glaucoma | 2018 | 2021 | 0.00439912 | 1.24  (0.67, 1.75) |
| DALYs | South Asia | Female | Glaucoma | 1990 | 1995 | 0.189162168 | 0.21  (-0.15, 0.65) |
| DALYs | South Asia | Female | Glaucoma | 1995 | 2006 | 0.00239952 | -1.68  (-1.78, -1.54) |
| DALYs | South Asia | Female | Glaucoma | 2006 | 2009 | 0 | -3.3  (-3.58, -2.61) |
| DALYs | South Asia | Female | Glaucoma | 2009 | 2018 | 0 | -2.2  (-2.33, -1.91) |
| DALYs | South Asia | Female | Glaucoma | 2018 | 2021 | 0.01079784 | 0.87  (0.27, 2.02) |
| DALYs | South Asia | Male | Glaucoma | 1990 | 1996 | 0.061987602 | 0.25  (-0.02, 0.6) |
| DALYs | South Asia | Male | Glaucoma | 1996 | 2000 | 0.00039992 | -2.87  (-3.59, -2.28) |
| DALYs | South Asia | Male | Glaucoma | 2000 | 2005 | 0.00239952 | -1.19  (-1.57, -0.36) |
| DALYs | South Asia | Male | Glaucoma | 2005 | 2018 | 0 | -2.59  (-2.7, -2.49) |
| DALYs | South Asia | Male | Glaucoma | 2018 | 2021 | 0.00679864 | 1.23  (0.44, 1.96) |
| DALYs | Southeast Asia | Both | Glaucoma | 1990 | 1998 | 0 | -0.98  (-1.02, -0.92) |
| DALYs | Southeast Asia | Both | Glaucoma | 1998 | 2001 | 0 | -1.33  (-1.65, -1.09) |
| DALYs | Southeast Asia | Both | Glaucoma | 2001 | 2008 | 0 | -1.77  (-1.86, -1.71) |
| DALYs | Southeast Asia | Both | Glaucoma | 2008 | 2018 | 0 | -2.04  (-2.08, -2.01) |
| DALYs | Southeast Asia | Both | Glaucoma | 2018 | 2021 | 0 | -1.43  (-1.59, -1.17) |
| DALYs | Southeast Asia | Female | Glaucoma | 1990 | 1996 | 0 | -0.68  (-0.75, -0.6) |
| DALYs | Southeast Asia | Female | Glaucoma | 1996 | 2000 | 0 | -1.28  (-1.44, -1.09) |
| DALYs | Southeast Asia | Female | Glaucoma | 2000 | 2010 | 0 | -1.76  (-1.8, -1.72) |
| DALYs | Southeast Asia | Female | Glaucoma | 2010 | 2019 | 0 | -2.12  (-2.17, -2.08) |
| DALYs | Southeast Asia | Female | Glaucoma | 2019 | 2021 | 0 | -1.09  (-1.41, -0.91) |
| DALYs | Southeast Asia | Male | Glaucoma | 1990 | 1993 | 0 | -1.42  (-1.62, -1.28) |
| DALYs | Southeast Asia | Male | Glaucoma | 1993 | 2001 | 0 | -1.07  (-1.1, -1.01) |
| DALYs | Southeast Asia | Male | Glaucoma | 2001 | 2006 | 0 | -1.67  (-1.73, -1.42) |
| DALYs | Southeast Asia | Male | Glaucoma | 2006 | 2014 | 0 | -2.06  (-2.15, -1.72) |
| DALYs | Southeast Asia | Male | Glaucoma | 2014 | 2018 | 0 | -1.82  (-2.04, -1.72) |
| DALYs | Southeast Asia | Male | Glaucoma | 2018 | 2021 | 0 | -1.51  (-1.65, -1.35) |
| DALYs | Southern Latin America | Both | Glaucoma | 1990 | 1995 | 0 | -1.72  (-1.86, -1.64) |
| DALYs | Southern Latin America | Both | Glaucoma | 1995 | 2002 | 0 | -1.2  (-1.32, -1.13) |
| DALYs | Southern Latin America | Both | Glaucoma | 2002 | 2011 | 0 | -0.87  (-1.1, -0.81) |
| DALYs | Southern Latin America | Both | Glaucoma | 2011 | 2015 | 0 | -1.32  (-1.38, -0.87) |
| DALYs | Southern Latin America | Both | Glaucoma | 2015 | 2018 | 0 | -1.62  (-1.73, -1.46) |
| DALYs | Southern Latin America | Both | Glaucoma | 2018 | 2021 | 0 | -0.84  (-0.97, -0.62) |
| DALYs | Southern Latin America | Female | Glaucoma | 1990 | 1993 | 0 | -1.59  (-1.8, -1.46) |
| DALYs | Southern Latin America | Female | Glaucoma | 1993 | 2003 | 0 | -1.22  (-1.25, -1.18) |
| DALYs | Southern Latin America | Female | Glaucoma | 2003 | 2007 | 0 | -0.87  (-1.13, -0.75) |
| DALYs | Southern Latin America | Female | Glaucoma | 2007 | 2011 | 0 | -1.07  (-1.45, -0.99) |
| DALYs | Southern Latin America | Female | Glaucoma | 2011 | 2019 | 0 | -1.58  (-1.61, -1.54) |
| DALYs | Southern Latin America | Female | Glaucoma | 2019 | 2021 | 0 | -0.52  (-0.68, -0.4) |
| DALYs | Southern Latin America | Male | Glaucoma | 1990 | 1996 | 0 | -1.96  (-2.01, -1.91) |
| DALYs | Southern Latin America | Male | Glaucoma | 1996 | 2003 | 0 | -1.1  (-1.17, -1.05) |
| DALYs | Southern Latin America | Male | Glaucoma | 2003 | 2011 | 0 | -0.72  (-0.78, -0.62) |
| DALYs | Southern Latin America | Male | Glaucoma | 2011 | 2015 | 0 | -1.02  (-1.14, -0.78) |
| DALYs | Southern Latin America | Male | Glaucoma | 2015 | 2018 | 0 | -1.52  (-1.62, -1.34) |
| DALYs | Southern Latin America | Male | Glaucoma | 2018 | 2021 | 0 | -0.91  (-1.03, -0.69) |
| DALYs | Southern Sub-Saharan Africa | Both | Glaucoma | 1990 | 2000 | 0.00159968 | -0.66  (-1.22, -0.45) |
| DALYs | Southern Sub-Saharan Africa | Both | Glaucoma | 2000 | 2006 | 0.170765847 | 0.29  (-0.17, 1.25) |
| DALYs | Southern Sub-Saharan Africa | Both | Glaucoma | 2006 | 2010 | 0.00119976 | -5.16  (-6.33, -4.26) |
| DALYs | Southern Sub-Saharan Africa | Both | Glaucoma | 2010 | 2019 | 0 | -1.29  (-1.54, -1.03) |
| DALYs | Southern Sub-Saharan Africa | Both | Glaucoma | 2019 | 2021 | 0 | 7.63  (5.73, 9.07) |
| DALYs | Southern Sub-Saharan Africa | Female | Glaucoma | 1990 | 2001 | 0.00039992 | -0.7  (-1.1, -0.5) |
| DALYs | Southern Sub-Saharan Africa | Female | Glaucoma | 2001 | 2005 | 0.039992002 | 1.1  (0.05, 2.26) |
| DALYs | Southern Sub-Saharan Africa | Female | Glaucoma | 2005 | 2011 | 0.0019996 | -4.08  (-4.95, -3.55) |
| DALYs | Southern Sub-Saharan Africa | Female | Glaucoma | 2011 | 2019 | 0 | -1.21  (-1.57, -0.81) |
| DALYs | Southern Sub-Saharan Africa | Female | Glaucoma | 2019 | 2021 | 0 | 8.25  (5.54, 9.99) |
| DALYs | Southern Sub-Saharan Africa | Male | Glaucoma | 1990 | 2000 | 0 | -0.36  (-0.69, -0.22) |
| DALYs | Southern Sub-Saharan Africa | Male | Glaucoma | 2000 | 2006 | 0.027994401 | 0.33  (0.04, 0.85) |
| DALYs | Southern Sub-Saharan Africa | Male | Glaucoma | 2006 | 2010 | 0 | -5.1  (-5.44, -4.72) |
| DALYs | Southern Sub-Saharan Africa | Male | Glaucoma | 2010 | 2019 | 0 | -1.31  (-1.46, -1.15) |
| DALYs | Southern Sub-Saharan Africa | Male | Glaucoma | 2019 | 2021 | 0 | 6.72  (5.76, 7.73) |
| DALYs | Tropical Latin America | Both | Glaucoma | 1990 | 1994 | 0 | -5.21  (-5.44, -4.98) |
| DALYs | Tropical Latin America | Both | Glaucoma | 1994 | 1997 | 0 | -1.72  (-1.97, -1.32) |
| DALYs | Tropical Latin America | Both | Glaucoma | 1997 | 2005 | 0.101979604 | -0.1  (-0.2, 0.04) |
| DALYs | Tropical Latin America | Both | Glaucoma | 2005 | 2010 | 0 | -1.64  (-1.82, -1.46) |
| DALYs | Tropical Latin America | Both | Glaucoma | 2010 | 2015 | 0 | 1.46  (1.32, 1.61) |
| DALYs | Tropical Latin America | Both | Glaucoma | 2015 | 2021 | 0 | -1.01  (-1.14, -0.88) |
| DALYs | Tropical Latin America | Female | Glaucoma | 1990 | 1994 | 0 | -5.92  (-6.23, -5.59) |
| DALYs | Tropical Latin America | Female | Glaucoma | 1994 | 1998 | 0 | -1.35  (-1.94, -0.99) |
| DALYs | Tropical Latin America | Female | Glaucoma | 1998 | 2005 | 0 | 0.42  (0.23, 0.7) |
| DALYs | Tropical Latin America | Female | Glaucoma | 2005 | 2010 | 0 | -1.62  (-1.88, -1.34) |
| DALYs | Tropical Latin America | Female | Glaucoma | 2010 | 2015 | 0 | 1.88  (1.65, 2.13) |
| DALYs | Tropical Latin America | Female | Glaucoma | 2015 | 2021 | 0 | -1.16  (-1.38, -0.98) |
| DALYs | Tropical Latin America | Male | Glaucoma | 1990 | 1993 | 0 | -4.95  (-5.33, -4.64) |
| DALYs | Tropical Latin America | Male | Glaucoma | 1993 | 1996 | 0 | -2.67  (-2.94, -2.37) |
| DALYs | Tropical Latin America | Male | Glaucoma | 1996 | 2005 | 0 | -0.47  (-0.55, -0.39) |
| DALYs | Tropical Latin America | Male | Glaucoma | 2005 | 2010 | 0 | -1.67  (-1.84, -1.48) |
| DALYs | Tropical Latin America | Male | Glaucoma | 2010 | 2015 | 0 | 1.05  (0.91, 1.2) |
| DALYs | Tropical Latin America | Male | Glaucoma | 2015 | 2021 | 0 | -0.84  (-0.97, -0.72) |
| DALYs | Western Europe | Both | Glaucoma | 1990 | 1996 | 0 | -1.71  (-1.76, -1.65) |
| DALYs | Western Europe | Both | Glaucoma | 1996 | 2000 | 0 | -2.05  (-2.14, -1.98) |
| DALYs | Western Europe | Both | Glaucoma | 2000 | 2009 | 0 | -1.13  (-1.16, -1.11) |
| DALYs | Western Europe | Both | Glaucoma | 2009 | 2015 | 0 | -0.54  (-0.59, -0.49) |
| DALYs | Western Europe | Both | Glaucoma | 2015 | 2019 | 0 | -0.89  (-1.02, -0.81) |
| DALYs | Western Europe | Both | Glaucoma | 2019 | 2021 | 0 | -0.45  (-0.65, -0.29) |
| DALYs | Western Europe | Female | Glaucoma | 1990 | 1996 | 0 | -1.7  (-1.75, -1.63) |
| DALYs | Western Europe | Female | Glaucoma | 1996 | 2000 | 0 | -2.04  (-2.16, -1.95) |
| DALYs | Western Europe | Female | Glaucoma | 2000 | 2009 | 0 | -1.3  (-1.33, -1.27) |
| DALYs | Western Europe | Female | Glaucoma | 2009 | 2015 | 0 | -0.49  (-0.54, -0.45) |
| DALYs | Western Europe | Female | Glaucoma | 2015 | 2019 | 0 | -1.16  (-1.31, -1.05) |
| DALYs | Western Europe | Female | Glaucoma | 2019 | 2021 | 0 | -0.47  (-0.71, -0.3) |
| DALYs | Western Europe | Male | Glaucoma | 1990 | 1996 | 0 | -1.8  (-1.85, -1.75) |
| DALYs | Western Europe | Male | Glaucoma | 1996 | 2000 | 0 | -2.11  (-2.19, -2.02) |
| DALYs | Western Europe | Male | Glaucoma | 2000 | 2005 | 0 | -1.23  (-1.36, -1.18) |
| DALYs | Western Europe | Male | Glaucoma | 2005 | 2009 | 0 | -1  (-1.15, -0.84) |
| DALYs | Western Europe | Male | Glaucoma | 2009 | 2018 | 0 | -0.76  (-0.81, -0.72) |
| DALYs | Western Europe | Male | Glaucoma | 2018 | 2021 | 0 | -0.55  (-0.67, -0.38) |
| DALYs | Western Sub-Saharan Africa | Both | Glaucoma | 1990 | 1993 | 0 | 1.17  (0.94, 1.54) |
| DALYs | Western Sub-Saharan Africa | Both | Glaucoma | 1993 | 1996 | 0.024395121 | -0.35  (-0.58, -0.04) |
| DALYs | Western Sub-Saharan Africa | Both | Glaucoma | 1996 | 1999 | 0 | -3.45  (-3.64, -3.26) |
| DALYs | Western Sub-Saharan Africa | Both | Glaucoma | 1999 | 2009 | 0 | -0.53  (-0.59, -0.46) |
| DALYs | Western Sub-Saharan Africa | Both | Glaucoma | 2009 | 2015 | 0 | -1.1  (-1.4, -0.96) |
| DALYs | Western Sub-Saharan Africa | Both | Glaucoma | 2015 | 2021 | 0.00439912 | -0.45  (-0.58, -0.24) |
| DALYs | Western Sub-Saharan Africa | Female | Glaucoma | 1990 | 1995 | 0.049590082 | 0.15  (0, 0.3) |
| DALYs | Western Sub-Saharan Africa | Female | Glaucoma | 1995 | 2000 | 0.031193761 | -2.58  (-2.69, -2.41) |
| DALYs | Western Sub-Saharan Africa | Female | Glaucoma | 2000 | 2008 | 0.00119976 | -0.32  (-0.65, -0.23) |
| DALYs | Western Sub-Saharan Africa | Female | Glaucoma | 2008 | 2015 | 0.00519896 | -1.07  (-1.25, -0.27) |
| DALYs | Western Sub-Saharan Africa | Female | Glaucoma | 2015 | 2019 | 0.160767846 | 0.38  (-1.12, 0.75) |
| DALYs | Western Sub-Saharan Africa | Female | Glaucoma | 2019 | 2021 | 0.158768246 | -0.48  (-0.95, 0.15) |
| DALYs | Western Sub-Saharan Africa | Male | Glaucoma | 1990 | 1993 | 0 | 1.59  (1.34, 1.91) |
| DALYs | Western Sub-Saharan Africa | Male | Glaucoma | 1993 | 1996 | 0.044791042 | -0.3  (-0.54, -0.01) |
| DALYs | Western Sub-Saharan Africa | Male | Glaucoma | 1996 | 1999 | 0 | -3.58  (-3.78, -3.37) |
| DALYs | Western Sub-Saharan Africa | Male | Glaucoma | 1999 | 2008 | 0 | -0.64  (-0.7, -0.53) |
| DALYs | Western Sub-Saharan Africa | Male | Glaucoma | 2008 | 2021 | 0 | -0.91  (-0.97, -0.86) |
| Prevalence | Global | Both | Glaucoma | 1990 | 1994 | 0 | -1.59 (-1.67, -1.51) |
| Prevalence | Global | Both | Glaucoma | 1994 | 2001 | 0 | -0.18 (-0.22, -0.13) |
| Prevalence | Global | Both | Glaucoma | 2001 | 2006 | 0 | -0.93 (-1.01, -0.86) |
| Prevalence | Global | Both | Glaucoma | 2006 | 2010 | 0 | -1.49 (-1.61, -1.38) |
| Prevalence | Global | Both | Glaucoma | 2010 | 2019 | 0 | -0.29 (-0.32, -0.27) |
| Prevalence | Global | Both | Glaucoma | 2019 | 2021 | 0 | -2.32 (-2.57, -2.07) |
| Prevalence | Global | Female | Glaucoma | 1990 | 1994 | 0 | -1.45 (-1.54, -1.36) |
| Prevalence | Global | Female | Glaucoma | 1994 | 2001 | 0 | -0.02 (-0.06, 0.03) |
| Prevalence | Global | Female | Glaucoma | 2001 | 2006 | 0 | -0.83 (-0.92, -0.75) |
| Prevalence | Global | Female | Glaucoma | 2006 | 2010 | 0 | -1.45 (-1.59, -1.32) |
| Prevalence | Global | Female | Glaucoma | 2010 | 2019 | 0 | -0.13 (-0.16, -0.10) |
| Prevalence | Global | Female | Glaucoma | 2019 | 2021 | 0 | -2.26 (-2.54, -1.98) |
| Prevalence | Global | Male | Glaucoma | 1990 | 1994 | 0 | -1.78 (-1.86, -1.70) |
| Prevalence | Global | Male | Glaucoma | 1994 | 2001 | 0 | -0.38 (-0.43, -0.34) |
| Prevalence | Global | Male | Glaucoma | 2001 | 2006 | 0 | -1.09 (-1.16, -1.01) |
| Prevalence | Global | Male | Glaucoma | 2006 | 2010 | 0 | -1.60 (-1.72, -1.48) |
| Prevalence | Global | Male | Glaucoma | 2010 | 2019 | 0 | -0.51 (-0.54, -0.48) |
| Prevalence | Global | Male | Glaucoma | 2019 | 2021 | 0 | -2.36 (-2.61, -2.11) |
| Prevalence | Andean Latin America | Both | Glaucoma | 1990 | 1995 | 0 | -0.76  (-0.86, -0.67) |
| Prevalence | Andean Latin America | Both | Glaucoma | 1995 | 2000 | 0 | -1.59  (-1.72, -1.5) |
| Prevalence | Andean Latin America | Both | Glaucoma | 2000 | 2005 | 0 | -0.9  (-0.99, -0.72) |
| Prevalence | Andean Latin America | Both | Glaucoma | 2005 | 2011 | 0 | -1.17  (-1.27, -1.09) |
| Prevalence | Andean Latin America | Both | Glaucoma | 2011 | 2015 | 0 | -1.59  (-1.76, -1.46) |
| Prevalence | Andean Latin America | Both | Glaucoma | 2015 | 2021 | 0 | -0.82  (-0.89, -0.73) |
| Prevalence | Andean Latin America | Female | Glaucoma | 1990 | 1995 | 0 | -0.64  (-0.74, -0.54) |
| Prevalence | Andean Latin America | Female | Glaucoma | 1995 | 2000 | 0 | -1.64  (-1.79, -1.54) |
| Prevalence | Andean Latin America | Female | Glaucoma | 2000 | 2010 | 0 | -1.1  (-1.14, -1.05) |
| Prevalence | Andean Latin America | Female | Glaucoma | 2010 | 2015 | 0 | -1.55  (-1.73, -1.44) |
| Prevalence | Andean Latin America | Female | Glaucoma | 2015 | 2021 | 0 | -0.88  (-0.97, -0.78) |
| Prevalence | Andean Latin America | Male | Glaucoma | 1990 | 1995 | 0 | -0.87  (-0.99, -0.73) |
| Prevalence | Andean Latin America | Male | Glaucoma | 1995 | 2000 | 0 | -1.54  (-1.76, -1.42) |
| Prevalence | Andean Latin America | Male | Glaucoma | 2000 | 2006 | 0 | -0.76  (-0.86, -0.61) |
| Prevalence | Andean Latin America | Male | Glaucoma | 2006 | 2017 | 0 | -1.31  (-1.36, -1.27) |
| Prevalence | Andean Latin America | Male | Glaucoma | 2017 | 2021 | 0.0019996 | -0.52  (-0.69, -0.25) |
| Prevalence | Australasia | Both | Glaucoma | 1990 | 1998 | 0 | -0.99  (-1.05, -0.95) |
| Prevalence | Australasia | Both | Glaucoma | 1998 | 2005 | 0 | -0.54  (-0.93, -0.49) |
| Prevalence | Australasia | Both | Glaucoma | 2005 | 2010 | 0.123175365 | 0.37  (-0.54, 0.42) |
| Prevalence | Australasia | Both | Glaucoma | 2010 | 2019 | 0.132773445 | -0.5  (-0.58, 0.36) |
| Prevalence | Australasia | Both | Glaucoma | 2019 | 2021 | 0.00079984 | -0.24  (-0.49, -0.12) |
| Prevalence | Australasia | Female | Glaucoma | 1990 | 1998 | 0 | -0.99  (-1.03, -0.96) |
| Prevalence | Australasia | Female | Glaucoma | 1998 | 2005 | 0 | -0.45  (-0.5, -0.41) |
| Prevalence | Australasia | Female | Glaucoma | 2005 | 2010 | 0.0119976 | 0.18  (0.11, 0.3) |
| Prevalence | Australasia | Female | Glaucoma | 2010 | 2015 | 0.0119976 | -0.28  (-0.35, -0.16) |
| Prevalence | Australasia | Female | Glaucoma | 2015 | 2018 | 0 | -0.74  (-0.84, -0.58) |
| Prevalence | Australasia | Female | Glaucoma | 2018 | 2021 | 0.049990002 | -0.23  (-0.36, 0) |
| Prevalence | Australasia | Male | Glaucoma | 1990 | 1997 | 0 | -1.06  (-1.13, -1.01) |
| Prevalence | Australasia | Male | Glaucoma | 1997 | 2005 | 0 | -0.71  (-0.75, -0.66) |
| Prevalence | Australasia | Male | Glaucoma | 2005 | 2010 | 0 | 0.55  (0.49, 0.6) |
| Prevalence | Australasia | Male | Glaucoma | 2010 | 2015 | 0 | -0.75  (-0.86, -0.68) |
| Prevalence | Australasia | Male | Glaucoma | 2015 | 2021 | 0.00039992 | -0.41  (-0.47, -0.3) |
| Prevalence | Caribbean | Both | Glaucoma | 1990 | 2003 | 0 | -1.06  (-1.07, -1.05) |
| Prevalence | Caribbean | Both | Glaucoma | 2003 | 2008 | 0 | -0.91  (-0.97, -0.86) |
| Prevalence | Caribbean | Both | Glaucoma | 2008 | 2015 | 0 | -0.78  (-0.8, -0.72) |
| Prevalence | Caribbean | Both | Glaucoma | 2015 | 2021 | 0 | -0.88  (-0.92, -0.85) |
| Prevalence | Caribbean | Female | Glaucoma | 1990 | 2000 | 0 | -1.02  (-1.03, -1) |
| Prevalence | Caribbean | Female | Glaucoma | 2000 | 2005 | 0 | -0.87  (-0.9, -0.81) |
| Prevalence | Caribbean | Female | Glaucoma | 2005 | 2010 | 0 | -0.99  (-1.04, -0.97) |
| Prevalence | Caribbean | Female | Glaucoma | 2010 | 2015 | 0 | -0.66  (-0.69, -0.63) |
| Prevalence | Caribbean | Female | Glaucoma | 2015 | 2019 | 0 | -0.96  (-1.01, -0.92) |
| Prevalence | Caribbean | Female | Glaucoma | 2019 | 2021 | 0 | -0.23  (-0.31, -0.15) |
| Prevalence | Caribbean | Male | Glaucoma | 1990 | 2005 | 0 | -1.08  (-1.1, -1.07) |
| Prevalence | Caribbean | Male | Glaucoma | 2005 | 2010 | 0 | -0.73  (-1.06, -0.62) |
| Prevalence | Caribbean | Male | Glaucoma | 2010 | 2019 | 0 | -0.83  (-0.89, -0.8) |
| Prevalence | Caribbean | Male | Glaucoma | 2019 | 2021 | 0 | -1.3  (-1.43, -1.1) |
| Prevalence | Central Asia | Both | Glaucoma | 1990 | 1994 | 0 | -0.51  (-0.65, -0.42) |
| Prevalence | Central Asia | Both | Glaucoma | 1994 | 2001 | 0.00439912 | -0.11  (-0.14, -0.06) |
| Prevalence | Central Asia | Both | Glaucoma | 2001 | 2010 | 0.00159968 | -1.07  (-1.09, -1.02) |
| Prevalence | Central Asia | Both | Glaucoma | 2010 | 2015 | 0 | -1.22  (-1.35, -1.1) |
| Prevalence | Central Asia | Both | Glaucoma | 2015 | 2019 | 0 | -0.7  (-1.17, -0.63) |
| Prevalence | Central Asia | Both | Glaucoma | 2019 | 2021 | 0 | -0.32  (-0.53, -0.19) |
| Prevalence | Central Asia | Female | Glaucoma | 1990 | 1995 | 0.00319936 | -0.42  (-0.74, -0.29) |
| Prevalence | Central Asia | Female | Glaucoma | 1995 | 2000 | 0.970605879 | 0  (-0.15, 0.21) |
| Prevalence | Central Asia | Female | Glaucoma | 2000 | 2010 | 0.00759848 | -0.96  (-0.99, -0.86) |
| Prevalence | Central Asia | Female | Glaucoma | 2010 | 2015 | 0 | -1.16  (-1.42, -1.04) |
| Prevalence | Central Asia | Female | Glaucoma | 2015 | 2021 | 0 | -0.57  (-0.67, -0.41) |
| Prevalence | Central Asia | Male | Glaucoma | 1990 | 1994 | 0 | -0.52  (-0.66, -0.44) |
| Prevalence | Central Asia | Male | Glaucoma | 1994 | 2001 | 0 | -0.16  (-0.19, -0.12) |
| Prevalence | Central Asia | Male | Glaucoma | 2001 | 2006 | 0 | -1.22  (-1.27, -1.15) |
| Prevalence | Central Asia | Male | Glaucoma | 2006 | 2014 | 0 | -1.42  (-1.48, -1.39) |
| Prevalence | Central Asia | Male | Glaucoma | 2014 | 2018 | 0 | -1.13  (-1.2, -1.01) |
| Prevalence | Central Asia | Male | Glaucoma | 2018 | 2021 | 0.00759848 | -0.24  (-0.35, -0.15) |
| Prevalence | Central Europe | Both | Glaucoma | 1990 | 1994 | 0 | -1.01  (-1.1, -0.96) |
| Prevalence | Central Europe | Both | Glaucoma | 1994 | 2001 | 0 | -0.77  (-0.79, -0.74) |
| Prevalence | Central Europe | Both | Glaucoma | 2001 | 2004 | 0 | -1.25  (-1.3, -1.17) |
| Prevalence | Central Europe | Both | Glaucoma | 2004 | 2009 | 0 | -0.94  (-0.99, -0.88) |
| Prevalence | Central Europe | Both | Glaucoma | 2009 | 2017 | 0 | -0.68  (-0.7, -0.66) |
| Prevalence | Central Europe | Both | Glaucoma | 2017 | 2021 | 0 | -0.2  (-0.25, -0.16) |
| Prevalence | Central Europe | Female | Glaucoma | 1990 | 1994 | 0 | -1.26  (-1.31, -1.2) |
| Prevalence | Central Europe | Female | Glaucoma | 1994 | 2001 | 0 | -0.6  (-0.63, -0.57) |
| Prevalence | Central Europe | Female | Glaucoma | 2001 | 2004 | 0 | -1.2  (-1.26, -1.03) |
| Prevalence | Central Europe | Female | Glaucoma | 2004 | 2009 | 0 | -0.99  (-1.07, -0.85) |
| Prevalence | Central Europe | Female | Glaucoma | 2009 | 2018 | 0 | -0.8  (-0.83, -0.76) |
| Prevalence | Central Europe | Female | Glaucoma | 2018 | 2021 | 0.946610678 | 0  (-0.09, 0.07) |
| Prevalence | Central Europe | Male | Glaucoma | 1990 | 1996 | 0 | -0.76  (-0.81, -0.71) |
| Prevalence | Central Europe | Male | Glaucoma | 1996 | 2001 | 0 | -1.04  (-1.1, -0.91) |
| Prevalence | Central Europe | Male | Glaucoma | 2001 | 2004 | 0 | -1.26  (-1.33, -1.13) |
| Prevalence | Central Europe | Male | Glaucoma | 2004 | 2009 | 0 | -0.89  (-0.99, -0.79) |
| Prevalence | Central Europe | Male | Glaucoma | 2009 | 2014 | 0 | -0.68  (-0.75, -0.47) |
| Prevalence | Central Europe | Male | Glaucoma | 2014 | 2021 | 0 | -0.35  (-0.39, -0.3) |
| Prevalence | Central Latin America | Both | Glaucoma | 1990 | 1994 | 0 | -1.51  (-1.61, -1.45) |
| Prevalence | Central Latin America | Both | Glaucoma | 1994 | 2000 | 0 | -1.25  (-1.28, -1.21) |
| Prevalence | Central Latin America | Both | Glaucoma | 2000 | 2010 | 0 | -0.84  (-0.86, -0.82) |
| Prevalence | Central Latin America | Both | Glaucoma | 2010 | 2015 | 0 | -0.66  (-0.7, -0.56) |
| Prevalence | Central Latin America | Both | Glaucoma | 2015 | 2019 | 0 | -0.84  (-0.92, -0.8) |
| Prevalence | Central Latin America | Both | Glaucoma | 2019 | 2021 | 0.00079984 | -0.2  (-0.33, -0.1) |
| Prevalence | Central Latin America | Female | Glaucoma | 1990 | 1995 | 0 | -1.22  (-1.27, -1.19) |
| Prevalence | Central Latin America | Female | Glaucoma | 1995 | 2003 | 0 | -1.07  (-1.08, -1.04) |
| Prevalence | Central Latin America | Female | Glaucoma | 2003 | 2009 | 0 | -0.86  (-0.89, -0.82) |
| Prevalence | Central Latin America | Female | Glaucoma | 2009 | 2015 | 0 | -0.62  (-0.65, -0.59) |
| Prevalence | Central Latin America | Female | Glaucoma | 2015 | 2019 | 0 | -0.96  (-0.99, -0.92) |
| Prevalence | Central Latin America | Female | Glaucoma | 2019 | 2021 | 0.459108178 | 0.03  (-0.06, 0.1) |
| Prevalence | Central Latin America | Male | Glaucoma | 1990 | 1994 | 0 | -1.71  (-1.84, -1.63) |
| Prevalence | Central Latin America | Male | Glaucoma | 1994 | 2000 | 0 | -1.33  (-1.37, -1.28) |
| Prevalence | Central Latin America | Male | Glaucoma | 2000 | 2006 | 0 | -0.68  (-0.72, -0.62) |
| Prevalence | Central Latin America | Male | Glaucoma | 2006 | 2009 | 0 | -0.9  (-0.95, -0.79) |
| Prevalence | Central Latin America | Male | Glaucoma | 2009 | 2019 | 0 | -0.7  (-0.73, -0.67) |
| Prevalence | Central Latin America | Male | Glaucoma | 2019 | 2021 | 0 | -0.33  (-0.52, -0.23) |
| Prevalence | Central Sub-Saharan Africa | Both | Glaucoma | 1990 | 1994 | 0 | -0.3  (-0.48, -0.2) |
| Prevalence | Central Sub-Saharan Africa | Both | Glaucoma | 1994 | 2010 | 0 | 0.11  (0.1, 0.13) |
| Prevalence | Central Sub-Saharan Africa | Both | Glaucoma | 2010 | 2015 | 0 | -1.01  (-1.1, -0.95) |
| Prevalence | Central Sub-Saharan Africa | Both | Glaucoma | 2015 | 2021 | 0 | -0.63  (-0.69, -0.55) |
| Prevalence | Central Sub-Saharan Africa | Female | Glaucoma | 1990 | 1995 | 0.00039992 | -0.14  (-0.25, -0.07) |
| Prevalence | Central Sub-Saharan Africa | Female | Glaucoma | 1995 | 2000 | 0.014797041 | 0.23  (0.07, 0.33) |
| Prevalence | Central Sub-Saharan Africa | Female | Glaucoma | 2000 | 2004 | 0 | 0.49  (0.38, 0.6) |
| Prevalence | Central Sub-Saharan Africa | Female | Glaucoma | 2004 | 2010 | 0.021195761 | 0.17  (0.11, 0.21) |
| Prevalence | Central Sub-Saharan Africa | Female | Glaucoma | 2010 | 2015 | 0 | -0.87  (-0.94, -0.82) |
| Prevalence | Central Sub-Saharan Africa | Female | Glaucoma | 2015 | 2021 | 0 | -0.39  (-0.44, -0.33) |
| Prevalence | Central Sub-Saharan Africa | Male | Glaucoma | 1990 | 1995 | 0.093981204 | -0.1  (-0.44, 0.02) |
| Prevalence | Central Sub-Saharan Africa | Male | Glaucoma | 1995 | 2000 | 0.01159768 | 0.34  (0.21, 0.6) |
| Prevalence | Central Sub-Saharan Africa | Male | Glaucoma | 2000 | 2006 | 0.027594481 | -0.13  (-0.36, -0.02) |
| Prevalence | Central Sub-Saharan Africa | Male | Glaucoma | 2006 | 2010 | 0.141971606 | 0.14  (-0.98, 0.31) |
| Prevalence | Central Sub-Saharan Africa | Male | Glaucoma | 2010 | 2019 | 0.00519896 | -1.11  (-1.28, -1.07) |
| Prevalence | Central Sub-Saharan Africa | Male | Glaucoma | 2019 | 2021 | 0.00119976 | -0.47  (-0.92, -0.24) |
| Prevalence | East Asia | Both | Glaucoma | 1990 | 1994 | 0 | -7.67  (-10.21, -6.28) |
| Prevalence | East Asia | Both | Glaucoma | 1994 | 2000 | 0 | 3.46  (2.51, 4.86) |
| Prevalence | East Asia | Both | Glaucoma | 2000 | 2010 | 0 | -1.85  (-2.75, -1.39) |
| Prevalence | East Asia | Both | Glaucoma | 2010 | 2019 | 0.019996001 | 0.64  (0.13, 1.61) |
| Prevalence | East Asia | Both | Glaucoma | 2019 | 2021 | 0 | -12.93  (-15.77, -8.35) |
| Prevalence | East Asia | Female | Glaucoma | 1990 | 1994 | 0 | -6.65  (-9.39, -5.19) |
| Prevalence | East Asia | Female | Glaucoma | 1994 | 2000 | 0 | 3.73  (2.77, 5.19) |
| Prevalence | East Asia | Female | Glaucoma | 2000 | 2011 | 0 | -1.63  (-2.28, -1.23) |
| Prevalence | East Asia | Female | Glaucoma | 2011 | 2019 | 0.00639872 | 0.99  (0.39, 2.22) |
| Prevalence | East Asia | Female | Glaucoma | 2019 | 2021 | 0 | -12.92  (-15.82, -8.34) |
| Prevalence | East Asia | Male | Glaucoma | 1990 | 1994 | 0 | -8.79  (-10.42, -7.41) |
| Prevalence | East Asia | Male | Glaucoma | 1994 | 2000 | 0 | 3.15  (2.18, 4.49) |
| Prevalence | East Asia | Male | Glaucoma | 2000 | 2009 | 0 | -2.18  (-3.38, -1.63) |
| Prevalence | East Asia | Male | Glaucoma | 2009 | 2019 | 0.104379124 | 0.35  (-0.09, 1.15) |
| Prevalence | East Asia | Male | Glaucoma | 2019 | 2021 | 0 | -13.06  (-15.88, -8.42) |
| Prevalence | Eastern Europe | Both | Glaucoma | 1990 | 1994 | 0 | -0.56  (-0.87, -0.37) |
| Prevalence | Eastern Europe | Both | Glaucoma | 1994 | 2001 | 0.085982803 | 0.06  (-0.01, 0.17) |
| Prevalence | Eastern Europe | Both | Glaucoma | 2001 | 2008 | 0.00159968 | -1.5  (-1.6, -1.43) |
| Prevalence | Eastern Europe | Both | Glaucoma | 2008 | 2019 | 0 | -0.91  (-0.98, -0.86) |
| Prevalence | Eastern Europe | Both | Glaucoma | 2019 | 2021 | 0.113977205 | -0.19  (-0.67, 0.05) |
| Prevalence | Eastern Europe | Female | Glaucoma | 1990 | 1994 | 0 | -0.57  (-0.81, -0.43) |
| Prevalence | Eastern Europe | Female | Glaucoma | 1994 | 2001 | 0.111577684 | -0.07  (-0.13, 0.03) |
| Prevalence | Eastern Europe | Female | Glaucoma | 2001 | 2005 | 0.01159768 | -1.71  (-1.87, -0.35) |
| Prevalence | Eastern Europe | Female | Glaucoma | 2005 | 2009 | 0 | -1.26  (-1.56, -1) |
| Prevalence | Eastern Europe | Female | Glaucoma | 2009 | 2019 | 0 | -0.89  (-0.97, -0.84) |
| Prevalence | Eastern Europe | Female | Glaucoma | 2019 | 2021 | 0.00039992 | -0.37  (-0.73, -0.19) |
| Prevalence | Eastern Europe | Male | Glaucoma | 1990 | 1994 | 0 | -0.58  (-0.93, -0.38) |
| Prevalence | Eastern Europe | Male | Glaucoma | 1994 | 2001 | 0.00039992 | 0.28  (0.21, 0.4) |
| Prevalence | Eastern Europe | Male | Glaucoma | 2001 | 2010 | 0.0019996 | -1.43  (-1.52, -1.38) |
| Prevalence | Eastern Europe | Male | Glaucoma | 2010 | 2019 | 0 | -0.99  (-1.1, -0.9) |
| Prevalence | Eastern Europe | Male | Glaucoma | 2019 | 2021 | 0.623075385 | -0.05  (-0.61, 0.2) |
| Prevalence | Eastern Sub-Saharan Africa | Both | Glaucoma | 1990 | 1995 | 0 | -0.62  (-0.74, -0.5) |
| Prevalence | Eastern Sub-Saharan Africa | Both | Glaucoma | 1995 | 2000 | 0 | -1.84  (-1.94, -1.73) |
| Prevalence | Eastern Sub-Saharan Africa | Both | Glaucoma | 2000 | 2005 | 0.091581684 | 0.09  (-0.05, 0.17) |
| Prevalence | Eastern Sub-Saharan Africa | Both | Glaucoma | 2005 | 2015 | 0 | -0.91  (-0.95, -0.87) |
| Prevalence | Eastern Sub-Saharan Africa | Both | Glaucoma | 2015 | 2019 | 0 | 0.65  (0.53, 0.78) |
| Prevalence | Eastern Sub-Saharan Africa | Both | Glaucoma | 2019 | 2021 | 0 | -1.42  (-1.76, -1.08) |
| Prevalence | Eastern Sub-Saharan Africa | Female | Glaucoma | 1990 | 1996 | 0 | -0.81  (-0.89, -0.71) |
| Prevalence | Eastern Sub-Saharan Africa | Female | Glaucoma | 1996 | 1999 | 0 | -2.45  (-2.6, -2.28) |
| Prevalence | Eastern Sub-Saharan Africa | Female | Glaucoma | 1999 | 2006 | 0.113977205 | -0.05  (-0.13, 0.02) |
| Prevalence | Eastern Sub-Saharan Africa | Female | Glaucoma | 2006 | 2015 | 0 | -1.06  (-1.11, -1.01) |
| Prevalence | Eastern Sub-Saharan Africa | Female | Glaucoma | 2015 | 2019 | 0 | 0.99  (0.87, 1.12) |
| Prevalence | Eastern Sub-Saharan Africa | Female | Glaucoma | 2019 | 2021 | 0 | -1.37  (-1.72, -1.05) |
| Prevalence | Eastern Sub-Saharan Africa | Male | Glaucoma | 1990 | 1995 | 0 | -0.51  (-0.6, -0.42) |
| Prevalence | Eastern Sub-Saharan Africa | Male | Glaucoma | 1995 | 2000 | 0 | -1.73  (-1.81, -1.65) |
| Prevalence | Eastern Sub-Saharan Africa | Male | Glaucoma | 2000 | 2006 | 0.029994001 | -0.09  (-0.19, -0.02) |
| Prevalence | Eastern Sub-Saharan Africa | Male | Glaucoma | 2006 | 2015 | 0 | -0.82  (-0.87, -0.77) |
| Prevalence | Eastern Sub-Saharan Africa | Male | Glaucoma | 2015 | 2019 | 0 | 0.41  (0.29, 0.53) |
| Prevalence | Eastern Sub-Saharan Africa | Male | Glaucoma | 2019 | 2021 | 0 | -1.38  (-1.68, -1.08) |
| Prevalence | Global | Both | Glaucoma | 1990 | 1994 | 0 | -1.59  (-1.96, -1.42) |
| Prevalence | Global | Both | Glaucoma | 1994 | 2001 | 0.020795841 | -0.18  (-0.26, -0.06) |
| Prevalence | Global | Both | Glaucoma | 2001 | 2006 | 0.00279944 | -0.93  (-1.06, -0.72) |
| Prevalence | Global | Both | Glaucoma | 2006 | 2010 | 0.00159968 | -1.49  (-1.78, -1.32) |
| Prevalence | Global | Both | Glaucoma | 2010 | 2019 | 0.00159968 | -0.29  (-0.35, -0.21) |
| Prevalence | Global | Both | Glaucoma | 2019 | 2021 | 0 | -2.32  (-2.72, -1.62) |
| Prevalence | Global | Female | Glaucoma | 1990 | 1994 | 0 | -1.45  (-1.87, -1.25) |
| Prevalence | Global | Female | Glaucoma | 1994 | 2001 | 0.662667467 | -0.02  (-0.11, 0.14) |
| Prevalence | Global | Female | Glaucoma | 2001 | 2006 | 0.00639872 | -0.83  (-0.98, -0.59) |
| Prevalence | Global | Female | Glaucoma | 2006 | 2010 | 0.00479904 | -1.45  (-1.82, -1.25) |
| Prevalence | Global | Female | Glaucoma | 2010 | 2019 | 0.022395521 | -0.13  (-0.19, -0.03) |
| Prevalence | Global | Female | Glaucoma | 2019 | 2021 | 0 | -2.26  (-2.72, -1.5) |
| Prevalence | Global | Male | Glaucoma | 1990 | 1994 | 0 | -1.78  (-1.92, -1.64) |
| Prevalence | Global | Male | Glaucoma | 1994 | 2001 | 0.00079984 | -0.38  (-0.46, -0.3) |
| Prevalence | Global | Male | Glaucoma | 2001 | 2006 | 0 | -1.09  (-1.2, -0.9) |
| Prevalence | Global | Male | Glaucoma | 2006 | 2010 | 0 | -1.6  (-1.82, -1.46) |
| Prevalence | Global | Male | Glaucoma | 2010 | 2019 | 0 | -0.51  (-0.56, -0.45) |
| Prevalence | Global | Male | Glaucoma | 2019 | 2021 | 0 | -2.36  (-2.7, -1.75) |
| Prevalence | High SDI | Both | Glaucoma | 1990 | 1994 | 0 | -1.38  (-1.73, -1.22) |
| Prevalence | High SDI | Both | Glaucoma | 1994 | 2010 | 0 | -0.67  (-0.81, -0.49) |
| Prevalence | High SDI | Both | Glaucoma | 2010 | 2013 | 0.794641072 | -0.01  (-0.85, 0.13) |
| Prevalence | High SDI | Both | Glaucoma | 2013 | 2019 | 0 | 0.27  (0.18, 0.48) |
| Prevalence | High SDI | Both | Glaucoma | 2019 | 2021 | 0 | -0.93  (-1.31, -0.48) |
| Prevalence | High SDI | Female | Glaucoma | 1990 | 1994 | 0 | -1.29  (-1.64, -1.1) |
| Prevalence | High SDI | Female | Glaucoma | 1994 | 2011 | 0 | -0.72  (-0.74, -0.7) |
| Prevalence | High SDI | Female | Glaucoma | 2011 | 2019 | 0 | 0.18  (0.13, 0.24) |
| Prevalence | High SDI | Female | Glaucoma | 2019 | 2021 | 0 | -0.99  (-1.35, -0.54) |
| Prevalence | High SDI | Male | Glaucoma | 1990 | 1994 | 0 | -1.54  (-1.86, -1.39) |
| Prevalence | High SDI | Male | Glaucoma | 1994 | 2009 | 0 | -0.68  (-0.72, -0.65) |
| Prevalence | High SDI | Male | Glaucoma | 2009 | 2013 | 0.127574485 | -0.17  (-0.62, 0.08) |
| Prevalence | High SDI | Male | Glaucoma | 2013 | 2019 | 0 | 0.29  (0.21, 0.51) |
| Prevalence | High SDI | Male | Glaucoma | 2019 | 2021 | 0 | -0.91  (-1.28, -0.47) |
| Prevalence | High-income Asia Pacific | Both | Glaucoma | 1990 | 1994 | 0 | -1.71  (-1.85, -1.63) |
| Prevalence | High-income Asia Pacific | Both | Glaucoma | 1994 | 1999 | 0 | -1.22  (-1.33, -1.13) |
| Prevalence | High-income Asia Pacific | Both | Glaucoma | 1999 | 2007 | 0 | -0.78  (-0.86, -0.75) |
| Prevalence | High-income Asia Pacific | Both | Glaucoma | 2007 | 2010 | 0.043591282 | -0.53  (-0.66, -0.13) |
| Prevalence | High-income Asia Pacific | Both | Glaucoma | 2010 | 2015 | 0.00839832 | 0.19  (0.13, 0.32) |
| Prevalence | High-income Asia Pacific | Both | Glaucoma | 2015 | 2021 | 0.00519896 | -0.08  (-0.15, -0.04) |
| Prevalence | High-income Asia Pacific | Female | Glaucoma | 1990 | 1999 | 0 | -1.47  (-1.54, -1.43) |
| Prevalence | High-income Asia Pacific | Female | Glaucoma | 1999 | 2006 | 0 | -0.56  (-1.4, -0.46) |
| Prevalence | High-income Asia Pacific | Female | Glaucoma | 2006 | 2010 | 0.00359928 | -0.76  (-0.92, -0.62) |
| Prevalence | High-income Asia Pacific | Female | Glaucoma | 2010 | 2015 | 0.026794641 | 0.33  (0.22, 0.48) |
| Prevalence | High-income Asia Pacific | Female | Glaucoma | 2015 | 2019 | 0.030393921 | -0.17  (-0.37, -0.04) |
| Prevalence | High-income Asia Pacific | Female | Glaucoma | 2019 | 2021 | 0.015196961 | 0.51  (0.17, 0.73) |
| Prevalence | High-income Asia Pacific | Male | Glaucoma | 1990 | 1994 | 0 | -1.91  (-2.01, -1.82) |
| Prevalence | High-income Asia Pacific | Male | Glaucoma | 1994 | 2001 | 0 | -0.99  (-1.03, -0.9) |
| Prevalence | High-income Asia Pacific | Male | Glaucoma | 2001 | 2005 | 0 | -1.24  (-1.4, -1.13) |
| Prevalence | High-income Asia Pacific | Male | Glaucoma | 2005 | 2009 | 0.00039992 | -0.68  (-0.87, -0.45) |
| Prevalence | High-income Asia Pacific | Male | Glaucoma | 2009 | 2018 | 0.023195361 | -0.13  (-0.16, -0.05) |
| Prevalence | High-income Asia Pacific | Male | Glaucoma | 2018 | 2021 | 0 | -0.46  (-0.72, -0.3) |
| Prevalence | High-income North America | Both | Glaucoma | 1990 | 1995 | 0 | -1.17  (-1.35, -1.05) |
| Prevalence | High-income North America | Both | Glaucoma | 1995 | 2010 | 0 | -0.67  (-0.7, -0.65) |
| Prevalence | High-income North America | Both | Glaucoma | 2010 | 2015 | 0.035592881 | 0.98  (0.73, 1.05) |
| Prevalence | High-income North America | Both | Glaucoma | 2015 | 2018 | 0 | 1.51  (1.23, 1.65) |
| Prevalence | High-income North America | Both | Glaucoma | 2018 | 2021 | 0.158768246 | 0.14  (-0.11, 0.32) |
| Prevalence | High-income North America | Female | Glaucoma | 1990 | 1995 | 0 | -1.02  (-1.26, -0.91) |
| Prevalence | High-income North America | Female | Glaucoma | 1995 | 2010 | 0 | -0.77  (-0.8, -0.7) |
| Prevalence | High-income North America | Female | Glaucoma | 2010 | 2013 | 0.174765047 | 0.81  (-0.93, 0.94) |
| Prevalence | High-income North America | Female | Glaucoma | 2013 | 2018 | 0 | 1.14  (1.01, 1.33) |
| Prevalence | High-income North America | Female | Glaucoma | 2018 | 2021 | 0.732253549 | 0.03  (-0.19, 0.27) |
| Prevalence | High-income North America | Male | Glaucoma | 1990 | 1995 | 0 | -1.38  (-1.57, -1.24) |
| Prevalence | High-income North America | Male | Glaucoma | 1995 | 2010 | 0 | -0.62  (-0.64, -0.59) |
| Prevalence | High-income North America | Male | Glaucoma | 2010 | 2015 | 0.0019996 | 0.87  (0.71, 0.95) |
| Prevalence | High-income North America | Male | Glaucoma | 2015 | 2018 | 0 | 1.8  (1.53, 1.96) |
| Prevalence | High-income North America | Male | Glaucoma | 2018 | 2021 | 0.076384723 | 0.2  (-0.05, 0.39) |
| Prevalence | High-middle SDI | Both | Glaucoma | 1990 | 1995 | 0 | -2.45  (-3.22, -2.04) |
| Prevalence | High-middle SDI | Both | Glaucoma | 1995 | 2000 | 0.009998 | 0.64  (0.2, 1.7) |
| Prevalence | High-middle SDI | Both | Glaucoma | 2000 | 2010 | 0 | -1.45  (-1.74, -1.28) |
| Prevalence | High-middle SDI | Both | Glaucoma | 2010 | 2019 | 0.043991202 | -0.34  (-0.51, -0.02) |
| Prevalence | High-middle SDI | Both | Glaucoma | 2019 | 2021 | 0 | -5.12  (-6.3, -3.39) |
| Prevalence | High-middle SDI | Female | Glaucoma | 1990 | 1995 | 0 | -2.07  (-2.89, -1.64) |
| Prevalence | High-middle SDI | Female | Glaucoma | 1995 | 2000 | 0.023195361 | 0.56  (0.1, 1.64) |
| Prevalence | High-middle SDI | Female | Glaucoma | 2000 | 2010 | 0 | -1.45  (-1.74, -1.29) |
| Prevalence | High-middle SDI | Female | Glaucoma | 2010 | 2019 | 0.051189762 | -0.31  (-0.48, 0) |
| Prevalence | High-middle SDI | Female | Glaucoma | 2019 | 2021 | 0 | -4.96  (-6.12, -3.26) |
| Prevalence | High-middle SDI | Male | Glaucoma | 1990 | 1995 | 0 | -2.98  (-3.73, -2.56) |
| Prevalence | High-middle SDI | Male | Glaucoma | 1995 | 2000 | 0.0119976 | 0.6  (0.16, 1.58) |
| Prevalence | High-middle SDI | Male | Glaucoma | 2000 | 2010 | 0 | -1.52  (-1.85, -1.34) |
| Prevalence | High-middle SDI | Male | Glaucoma | 2010 | 2019 | 0.025994801 | -0.45  (-0.63, -0.1) |
| Prevalence | High-middle SDI | Male | Glaucoma | 2019 | 2021 | 0 | -5.24  (-6.44, -3.51) |
| Prevalence | Low SDI | Both | Glaucoma | 1990 | 1996 | 0.00559888 | -0.29  (-0.38, -0.2) |
| Prevalence | Low SDI | Both | Glaucoma | 1996 | 1999 | 0 | -1.67  (-1.8, -1.28) |
| Prevalence | Low SDI | Both | Glaucoma | 1999 | 2006 | 0 | -0.58  (-0.67, -0.48) |
| Prevalence | Low SDI | Both | Glaucoma | 2006 | 2009 | 0 | -1.7  (-1.84, -0.7) |
| Prevalence | Low SDI | Both | Glaucoma | 2009 | 2013 | 0 | -1.17  (-1.43, -0.65) |
| Prevalence | Low SDI | Both | Glaucoma | 2013 | 2021 | 0.00079984 | -0.51  (-0.58, -0.4) |
| Prevalence | Low SDI | Female | Glaucoma | 1990 | 1996 | 0.00279944 | -0.46  (-0.57, -0.32) |
| Prevalence | Low SDI | Female | Glaucoma | 1996 | 2000 | 0.00039992 | -1.3  (-1.56, -1.05) |
| Prevalence | Low SDI | Female | Glaucoma | 2000 | 2005 | 0.014397121 | -0.37  (-0.57, -0.17) |
| Prevalence | Low SDI | Female | Glaucoma | 2005 | 2013 | 0 | -1.41  (-1.48, -1.21) |
| Prevalence | Low SDI | Female | Glaucoma | 2013 | 2019 | 0.121175765 | -0.11  (-1.37, 0.07) |
| Prevalence | Low SDI | Female | Glaucoma | 2019 | 2021 | 0 | -0.69  (-1, -0.22) |
| Prevalence | Low SDI | Male | Glaucoma | 1990 | 1995 | 0.095980804 | -0.08  (-0.17, 0.02) |
| Prevalence | Low SDI | Male | Glaucoma | 1995 | 2000 | 0 | -1.36  (-1.5, -1.27) |
| Prevalence | Low SDI | Male | Glaucoma | 2000 | 2005 | 0.00039992 | -0.48  (-0.61, -0.33) |
| Prevalence | Low SDI | Male | Glaucoma | 2005 | 2012 | 0 | -1.35  (-1.45, -1.28) |
| Prevalence | Low SDI | Male | Glaucoma | 2012 | 2021 | 0 | -0.69  (-0.74, -0.64) |
| Prevalence | Low-middle SDI | Both | Glaucoma | 1990 | 1996 | 0 | -0.65  (-0.76, -0.51) |
| Prevalence | Low-middle SDI | Both | Glaucoma | 1996 | 2006 | 0 | -1.19  (-1.25, -1.1) |
| Prevalence | Low-middle SDI | Both | Glaucoma | 2006 | 2010 | 0 | -2.57  (-2.69, -1.21) |
| Prevalence | Low-middle SDI | Both | Glaucoma | 2010 | 2015 | 0 | -0.82  (-2.5, -0.63) |
| Prevalence | Low-middle SDI | Both | Glaucoma | 2015 | 2018 | 0 | -1.26  (-1.42, -0.96) |
| Prevalence | Low-middle SDI | Both | Glaucoma | 2018 | 2021 | 0.115976805 | -0.28  (-0.51, 0.14) |
| Prevalence | Low-middle SDI | Female | Glaucoma | 1990 | 1997 | 0.00079984 | -0.77  (-0.87, -0.64) |
| Prevalence | Low-middle SDI | Female | Glaucoma | 1997 | 2006 | 0 | -1.14  (-1.22, -1.07) |
| Prevalence | Low-middle SDI | Female | Glaucoma | 2006 | 2010 | 0 | -2.59  (-2.71, -2.27) |
| Prevalence | Low-middle SDI | Female | Glaucoma | 2010 | 2019 | 0 | -0.7  (-1.02, -0.65) |
| Prevalence | Low-middle SDI | Female | Glaucoma | 2019 | 2021 | 0.937812438 | 0.03  (-0.58, 0.29) |
| Prevalence | Low-middle SDI | Male | Glaucoma | 1990 | 1995 | 0.00359928 | -0.46  (-0.63, -0.23) |
| Prevalence | Low-middle SDI | Male | Glaucoma | 1995 | 2006 | 0 | -1.14  (-1.2, -1.08) |
| Prevalence | Low-middle SDI | Male | Glaucoma | 2006 | 2010 | 0 | -2.48  (-2.71, -2.23) |
| Prevalence | Low-middle SDI | Male | Glaucoma | 2010 | 2015 | 0 | -0.87  (-1, -0.59) |
| Prevalence | Low-middle SDI | Male | Glaucoma | 2015 | 2018 | 0 | -1.59  (-1.79, -1.27) |
| Prevalence | Low-middle SDI | Male | Glaucoma | 2018 | 2021 | 0.111977604 | -0.34  (-0.61, 0.16) |
| Prevalence | Middle SDI | Both | Glaucoma | 1990 | 1994 | 0 | -2.64  (-2.91, -2.39) |
| Prevalence | Middle SDI | Both | Glaucoma | 1994 | 2001 | 0.107578484 | 0.11  (-0.04, 0.33) |
| Prevalence | Middle SDI | Both | Glaucoma | 2001 | 2006 | 0.027194561 | -1.13  (-1.43, -0.28) |
| Prevalence | Middle SDI | Both | Glaucoma | 2006 | 2010 | 0.00679864 | -1.78  (-2.18, -0.79) |
| Prevalence | Middle SDI | Both | Glaucoma | 2010 | 2018 | 0.00639872 | -0.43  (-0.56, -0.26) |
| Prevalence | Middle SDI | Both | Glaucoma | 2018 | 2021 | 0 | -2.66  (-3.18, -2.19) |
| Prevalence | Middle SDI | Female | Glaucoma | 1990 | 1994 | 0 | -2.36  (-3.07, -2.02) |
| Prevalence | Middle SDI | Female | Glaucoma | 1994 | 2000 | 0.014797041 | 0.36  (0.15, 0.77) |
| Prevalence | Middle SDI | Female | Glaucoma | 2000 | 2005 | 0.016796641 | -0.83  (-1.16, -0.4) |
| Prevalence | Middle SDI | Female | Glaucoma | 2005 | 2010 | 0.00919816 | -1.69  (-2.31, -1.35) |
| Prevalence | Middle SDI | Female | Glaucoma | 2010 | 2019 | 0.00519896 | -0.43  (-0.55, -0.25) |
| Prevalence | Middle SDI | Female | Glaucoma | 2019 | 2021 | 0 | -3.74  (-4.51, -2.56) |
| Prevalence | Middle SDI | Male | Glaucoma | 1990 | 1994 | 0 | -2.99  (-3.25, -2.74) |
| Prevalence | Middle SDI | Male | Glaucoma | 1994 | 2001 | 0.639072186 | -0.04  (-0.2, 0.18) |
| Prevalence | Middle SDI | Male | Glaucoma | 2001 | 2005 | 0.027194561 | -1.05  (-1.52, -0.25) |
| Prevalence | Middle SDI | Male | Glaucoma | 2005 | 2010 | 0.00479904 | -1.7  (-2.13, -0.73) |
| Prevalence | Middle SDI | Male | Glaucoma | 2010 | 2018 | 0.00159968 | -0.56  (-0.7, -0.37) |
| Prevalence | Middle SDI | Male | Glaucoma | 2018 | 2021 | 0 | -2.61  (-3.11, -2.13) |
| Prevalence | North Africa and Middle East | Both | Glaucoma | 1990 | 2001 | 0 | -0.87  (-0.91, -0.82) |
| Prevalence | North Africa and Middle East | Both | Glaucoma | 2001 | 2010 | 0 | -1.54  (-1.63, -1.48) |
| Prevalence | North Africa and Middle East | Both | Glaucoma | 2010 | 2015 | 0 | -0.94  (-1.06, -0.63) |
| Prevalence | North Africa and Middle East | Both | Glaucoma | 2015 | 2018 | 0 | -1.77  (-1.95, -1.5) |
| Prevalence | North Africa and Middle East | Both | Glaucoma | 2018 | 2021 | 0.00359928 | -0.69  (-0.91, -0.27) |
| Prevalence | North Africa and Middle East | Female | Glaucoma | 1990 | 2000 | 0 | -0.72  (-0.77, -0.67) |
| Prevalence | North Africa and Middle East | Female | Glaucoma | 2000 | 2009 | 0 | -1.52  (-1.59, -1.46) |
| Prevalence | North Africa and Middle East | Female | Glaucoma | 2009 | 2015 | 0 | -0.86  (-0.95, -0.63) |
| Prevalence | North Africa and Middle East | Female | Glaucoma | 2015 | 2018 | 0 | -1.69  (-1.86, -1.4) |
| Prevalence | North Africa and Middle East | Female | Glaucoma | 2018 | 2021 | 0.019196161 | -0.54  (-0.77, -0.09) |
| Prevalence | North Africa and Middle East | Male | Glaucoma | 1990 | 1994 | 0 | -1.21  (-1.58, -1.03) |
| Prevalence | North Africa and Middle East | Male | Glaucoma | 1994 | 2001 | 0 | -0.81  (-0.88, -0.59) |
| Prevalence | North Africa and Middle East | Male | Glaucoma | 2001 | 2010 | 0 | -1.59  (-1.68, -1.53) |
| Prevalence | North Africa and Middle East | Male | Glaucoma | 2010 | 2015 | 0 | -1.12  (-1.24, -0.81) |
| Prevalence | North Africa and Middle East | Male | Glaucoma | 2015 | 2018 | 0 | -1.86  (-2.04, -1.59) |
| Prevalence | North Africa and Middle East | Male | Glaucoma | 2018 | 2021 | 0.00279944 | -0.77  (-0.99, -0.33) |
| Prevalence | Oceania | Both | Glaucoma | 1990 | 1996 | 0.00119976 | -1.03  (-1.62, -0.79) |
| Prevalence | Oceania | Both | Glaucoma | 1996 | 2008 | 0.080383923 | -0.45  (-0.53, 0.08) |
| Prevalence | Oceania | Both | Glaucoma | 2008 | 2021 | 0 | -0.65  (-0.82, -0.59) |
| Prevalence | Oceania | Female | Glaucoma | 1990 | 1995 | 0.00679864 | -0.99  (-2.19, -0.45) |
| Prevalence | Oceania | Female | Glaucoma | 1995 | 2010 | 0.229554089 | -0.29  (-0.39, 0.63) |
| Prevalence | Oceania | Female | Glaucoma | 2010 | 2014 | 0.027994401 | -1.26  (-2.06, -0.53) |
| Prevalence | Oceania | Female | Glaucoma | 2014 | 2021 | 0.092781444 | 0.28  (-0.09, 1.36) |
| Prevalence | Oceania | Male | Glaucoma | 1990 | 1994 | 0 | -1.18  (-1.44, -1.05) |
| Prevalence | Oceania | Male | Glaucoma | 1994 | 1999 | 0 | -0.86  (-0.99, -0.64) |
| Prevalence | Oceania | Male | Glaucoma | 1999 | 2014 | 0 | -0.59  (-0.6, -0.53) |
| Prevalence | Oceania | Male | Glaucoma | 2014 | 2017 | 0 | -0.95  (-1.19, -0.66) |
| Prevalence | Oceania | Male | Glaucoma | 2017 | 2021 | 0 | -1.43  (-1.58, -1.33) |
| Prevalence | South Asia | Both | Glaucoma | 1990 | 1995 | 0.23755249 | -0.19  (-0.45, 0.2) |
| Prevalence | South Asia | Both | Glaucoma | 1995 | 2006 | 0 | -1.1  (-1.2, -1.01) |
| Prevalence | South Asia | Both | Glaucoma | 2006 | 2009 | 0 | -4.17  (-4.43, -3.52) |
| Prevalence | South Asia | Both | Glaucoma | 2009 | 2018 | 0 | -1.23  (-1.38, -1.11) |
| Prevalence | South Asia | Both | Glaucoma | 2018 | 2021 | 0.027594481 | 0.68  (0.08, 1.71) |
| Prevalence | South Asia | Female | Glaucoma | 1990 | 1996 | 0.00879824 | -0.39  (-0.54, -0.19) |
| Prevalence | South Asia | Female | Glaucoma | 1996 | 2006 | 0.00279944 | -1.03  (-1.12, -0.96) |
| Prevalence | South Asia | Female | Glaucoma | 2006 | 2009 | 0 | -4.47  (-4.69, -4.13) |
| Prevalence | South Asia | Female | Glaucoma | 2009 | 2012 | 0 | -1.5  (-1.86, -1.2) |
| Prevalence | South Asia | Female | Glaucoma | 2012 | 2018 | 0.00039992 | -0.84  (-1.02, -0.54) |
| Prevalence | South Asia | Female | Glaucoma | 2018 | 2021 | 0 | 0.73  (0.37, 1.36) |
| Prevalence | South Asia | Male | Glaucoma | 1990 | 1995 | 0.49270146 | -0.13  (-0.44, 0.46) |
| Prevalence | South Asia | Male | Glaucoma | 1995 | 2006 | 0 | -1.03  (-1.15, -0.92) |
| Prevalence | South Asia | Male | Glaucoma | 2006 | 2009 | 0 | -3.72  (-4.01, -2.92) |
| Prevalence | South Asia | Male | Glaucoma | 2009 | 2019 | 0 | -1.24  (-1.39, -1.09) |
| Prevalence | South Asia | Male | Glaucoma | 2019 | 2021 | 0.031193761 | 1.42  (0.18, 2.09) |
| Prevalence | Southeast Asia | Both | Glaucoma | 1990 | 2000 | 0 | -0.75  (-0.78, -0.72) |
| Prevalence | Southeast Asia | Both | Glaucoma | 2000 | 2010 | 0 | -1.33  (-1.4, -1.31) |
| Prevalence | Southeast Asia | Both | Glaucoma | 2010 | 2019 | 0 | -1.25  (-1.29, -1.19) |
| Prevalence | Southeast Asia | Both | Glaucoma | 2019 | 2021 | 0 | -0.78  (-1.02, -0.63) |
| Prevalence | Southeast Asia | Female | Glaucoma | 1990 | 1996 | 0 | -0.5  (-0.55, -0.45) |
| Prevalence | Southeast Asia | Female | Glaucoma | 1996 | 2000 | 0 | -0.96  (-1.06, -0.84) |
| Prevalence | Southeast Asia | Female | Glaucoma | 2000 | 2009 | 0 | -1.32  (-1.39, -1.29) |
| Prevalence | Southeast Asia | Female | Glaucoma | 2009 | 2015 | 0 | -1.19  (-1.24, -1.08) |
| Prevalence | Southeast Asia | Female | Glaucoma | 2015 | 2019 | 0 | -1.49  (-1.6, -1.41) |
| Prevalence | Southeast Asia | Female | Glaucoma | 2019 | 2021 | 0 | -0.64  (-0.83, -0.49) |
| Prevalence | Southeast Asia | Male | Glaucoma | 1990 | 1994 | 0 | -1.04  (-1.17, -0.96) |
| Prevalence | Southeast Asia | Male | Glaucoma | 1994 | 2000 | 0 | -0.69  (-0.73, -0.63) |
| Prevalence | Southeast Asia | Male | Glaucoma | 2000 | 2006 | 0 | -1.22  (-1.26, -1.17) |
| Prevalence | Southeast Asia | Male | Glaucoma | 2006 | 2010 | 0 | -1.51  (-1.61, -1.43) |
| Prevalence | Southeast Asia | Male | Glaucoma | 2010 | 2019 | 0 | -1.17  (-1.2, -1.14) |
| Prevalence | Southeast Asia | Male | Glaucoma | 2019 | 2021 | 0 | -0.76  (-0.96, -0.66) |
| Prevalence | Southern Latin America | Both | Glaucoma | 1990 | 1993 | 0 | -1.43  (-1.6, -1.34) |
| Prevalence | Southern Latin America | Both | Glaucoma | 1993 | 1996 | 0 | -1.03  (-1.23, -0.82) |
| Prevalence | Southern Latin America | Both | Glaucoma | 1996 | 2004 | 0 | -0.74  (-0.77, -0.69) |
| Prevalence | Southern Latin America | Both | Glaucoma | 2004 | 2012 | 0 | -0.53  (-0.55, -0.5) |
| Prevalence | Southern Latin America | Both | Glaucoma | 2012 | 2019 | 0 | -0.89  (-0.92, -0.86) |
| Prevalence | Southern Latin America | Both | Glaucoma | 2019 | 2021 | 0 | -0.25  (-0.39, -0.16) |
| Prevalence | Southern Latin America | Female | Glaucoma | 1990 | 1994 | 0 | -1.21  (-1.34, -1.14) |
| Prevalence | Southern Latin America | Female | Glaucoma | 1994 | 2005 | 0 | -0.76  (-0.78, -0.74) |
| Prevalence | Southern Latin America | Female | Glaucoma | 2005 | 2011 | 0 | -0.55  (-0.6, -0.46) |
| Prevalence | Southern Latin America | Female | Glaucoma | 2011 | 2019 | 0 | -0.87  (-0.9, -0.84) |
| Prevalence | Southern Latin America | Female | Glaucoma | 2019 | 2021 | 0.9930014 | 0  (-0.15, 0.11) |
| Prevalence | Southern Latin America | Male | Glaucoma | 1990 | 1995 | 0 | -1.5  (-1.64, -1.39) |
| Prevalence | Southern Latin America | Male | Glaucoma | 1995 | 2003 | 0 | -0.77  (-0.84, -0.71) |
| Prevalence | Southern Latin America | Male | Glaucoma | 2003 | 2012 | 0 | -0.47  (-0.52, -0.4) |
| Prevalence | Southern Latin America | Male | Glaucoma | 2012 | 2021 | 0 | -0.86  (-0.91, -0.81) |
| Prevalence | Southern Sub-Saharan Africa | Both | Glaucoma | 1990 | 2001 | 0 | -0.5  (-0.75, -0.36) |
| Prevalence | Southern Sub-Saharan Africa | Both | Glaucoma | 2001 | 2005 | 0.00679864 | 1.03  (0.3, 1.82) |
| Prevalence | Southern Sub-Saharan Africa | Both | Glaucoma | 2005 | 2011 | 0.00039992 | -3.14  (-3.69, -2.79) |
| Prevalence | Southern Sub-Saharan Africa | Both | Glaucoma | 2011 | 2019 | 0 | -0.79  (-1.03, -0.52) |
| Prevalence | Southern Sub-Saharan Africa | Both | Glaucoma | 2019 | 2021 | 0 | 6.12  (4.5, 7.29) |
| Prevalence | Southern Sub-Saharan Africa | Female | Glaucoma | 1990 | 2001 | 0.00039992 | -0.52  (-0.82, -0.36) |
| Prevalence | Southern Sub-Saharan Africa | Female | Glaucoma | 2001 | 2005 | 0.021195761 | 0.98  (0.13, 1.93) |
| Prevalence | Southern Sub-Saharan Africa | Female | Glaucoma | 2005 | 2011 | 0.00239952 | -3.04  (-3.76, -2.63) |
| Prevalence | Southern Sub-Saharan Africa | Female | Glaucoma | 2011 | 2019 | 0 | -0.75  (-1.03, -0.45) |
| Prevalence | Southern Sub-Saharan Africa | Female | Glaucoma | 2019 | 2021 | 0 | 6.71  (4.55, 8.12) |
| Prevalence | Southern Sub-Saharan Africa | Male | Glaucoma | 1990 | 2000 | 0 | -0.28  (-0.46, -0.16) |
| Prevalence | Southern Sub-Saharan Africa | Male | Glaucoma | 2000 | 2006 | 0 | 0.52  (0.29, 0.9) |
| Prevalence | Southern Sub-Saharan Africa | Male | Glaucoma | 2006 | 2010 | 0 | -4.09  (-4.37, -3.8) |
| Prevalence | Southern Sub-Saharan Africa | Male | Glaucoma | 2010 | 2019 | 0 | -0.99  (-1.11, -0.86) |
| Prevalence | Southern Sub-Saharan Africa | Male | Glaucoma | 2019 | 2021 | 0 | 5.62  (4.88, 6.4) |
| Prevalence | Tropical Latin America | Both | Glaucoma | 1990 | 1992 | 0 | -5.5  (-6.45, -3.88) |
| Prevalence | Tropical Latin America | Both | Glaucoma | 1992 | 1995 | 0.23875225 | -3.27  (-3.68, 1.25) |
| Prevalence | Tropical Latin America | Both | Glaucoma | 1995 | 2001 | 0.181563687 | 0.96  (-1.09, 1.72) |
| Prevalence | Tropical Latin America | Both | Glaucoma | 2001 | 2010 | 0.099180164 | -0.87  (-1.19, 1.94) |
| Prevalence | Tropical Latin America | Both | Glaucoma | 2010 | 2015 | 0.065586883 | 1.94  (-1.48, 2.6) |
| Prevalence | Tropical Latin America | Both | Glaucoma | 2015 | 2021 | 0 | -1.92  (-2.29, -1.53) |
| Prevalence | Tropical Latin America | Female | Glaucoma | 1990 | 1994 | 0 | -5.3  (-5.71, -4.87) |
| Prevalence | Tropical Latin America | Female | Glaucoma | 1994 | 2003 | 0.01159768 | 0.64  (0.41, 0.88) |
| Prevalence | Tropical Latin America | Female | Glaucoma | 2003 | 2010 | 0.069986003 | -0.94  (-1.38, 0.41) |
| Prevalence | Tropical Latin America | Female | Glaucoma | 2010 | 2015 | 0.116776645 | 2.56  (-0.98, 3.03) |
| Prevalence | Tropical Latin America | Female | Glaucoma | 2015 | 2019 | 0.131573685 | -2.75  (-3.48, 2.52) |
| Prevalence | Tropical Latin America | Female | Glaucoma | 2019 | 2021 | 0.225554889 | -0.58  (-2.27, 0.44) |
| Prevalence | Tropical Latin America | Male | Glaucoma | 1990 | 1992 | 0 | -4.91  (-5.61, -3.75) |
| Prevalence | Tropical Latin America | Male | Glaucoma | 1992 | 1995 | 0.097980404 | -3.09  (-3.45, 1.2) |
| Prevalence | Tropical Latin America | Male | Glaucoma | 1995 | 2000 | 0.082783443 | 1.05  (-0.89, 1.45) |
| Prevalence | Tropical Latin America | Male | Glaucoma | 2000 | 2010 | 0.039192162 | -0.9  (-1.03, -0.67) |
| Prevalence | Tropical Latin America | Male | Glaucoma | 2010 | 2015 | 0.029194161 | 1.51  (1.11, 1.81) |
| Prevalence | Tropical Latin America | Male | Glaucoma | 2015 | 2021 | 0 | -1.61  (-1.84, -1.4) |
| Prevalence | Western Europe | Both | Glaucoma | 1990 | 1996 | 0 | -1.4  (-1.44, -1.31) |
| Prevalence | Western Europe | Both | Glaucoma | 1996 | 2000 | 0 | -1.62  (-1.72, -1.52) |
| Prevalence | Western Europe | Both | Glaucoma | 2000 | 2009 | 0 | -0.91  (-0.95, -0.88) |
| Prevalence | Western Europe | Both | Glaucoma | 2009 | 2015 | 0 | -0.38  (-0.82, -0.3) |
| Prevalence | Western Europe | Both | Glaucoma | 2015 | 2019 | 0 | -0.76  (-0.9, -0.4) |
| Prevalence | Western Europe | Both | Glaucoma | 2019 | 2021 | 0 | -0.36  (-0.64, -0.2) |
| Prevalence | Western Europe | Female | Glaucoma | 1990 | 1996 | 0 | -1.37  (-1.41, -1.29) |
| Prevalence | Western Europe | Female | Glaucoma | 1996 | 2000 | 0 | -1.57  (-1.69, -1.49) |
| Prevalence | Western Europe | Female | Glaucoma | 2000 | 2009 | 0 | -0.98  (-1.01, -0.95) |
| Prevalence | Western Europe | Female | Glaucoma | 2009 | 2015 | 0 | -0.34  (-0.38, -0.3) |
| Prevalence | Western Europe | Female | Glaucoma | 2015 | 2019 | 0 | -0.92  (-1.05, -0.81) |
| Prevalence | Western Europe | Female | Glaucoma | 2019 | 2021 | 0.00039992 | -0.36  (-0.58, -0.21) |
| Prevalence | Western Europe | Male | Glaucoma | 1990 | 1996 | 0 | -1.5  (-1.62, -1.32) |
| Prevalence | Western Europe | Male | Glaucoma | 1996 | 2000 | 0 | -1.7  (-1.86, -1.14) |
| Prevalence | Western Europe | Male | Glaucoma | 2000 | 2005 | 0 | -1.06  (-1.71, -0.82) |
| Prevalence | Western Europe | Male | Glaucoma | 2005 | 2009 | 0 | -0.81  (-1.02, -0.48) |
| Prevalence | Western Europe | Male | Glaucoma | 2009 | 2021 | 0 | -0.58  (-0.68, -0.49) |
| Prevalence | Western Sub-Saharan Africa | Both | Glaucoma | 1990 | 1993 | 0 | 0.3  (0.16, 0.59) |
| Prevalence | Western Sub-Saharan Africa | Both | Glaucoma | 1993 | 1996 | 0 | -0.54  (-0.68, -0.37) |
| Prevalence | Western Sub-Saharan Africa | Both | Glaucoma | 1996 | 1999 | 0 | -2.72  (-2.83, -2.6) |
| Prevalence | Western Sub-Saharan Africa | Both | Glaucoma | 1999 | 2009 | 0 | -0.34  (-0.37, -0.29) |
| Prevalence | Western Sub-Saharan Africa | Both | Glaucoma | 2009 | 2015 | 0 | -0.86  (-1.01, -0.77) |
| Prevalence | Western Sub-Saharan Africa | Both | Glaucoma | 2015 | 2021 | 0 | -0.36  (-0.44, -0.25) |
| Prevalence | Western Sub-Saharan Africa | Female | Glaucoma | 1990 | 1996 | 0 | -0.55  (-0.63, -0.46) |
| Prevalence | Western Sub-Saharan Africa | Female | Glaucoma | 1996 | 1999 | 0 | -2.58  (-2.7, -2.45) |
| Prevalence | Western Sub-Saharan Africa | Female | Glaucoma | 1999 | 2010 | 0 | -0.28  (-0.32, -0.23) |
| Prevalence | Western Sub-Saharan Africa | Female | Glaucoma | 2010 | 2015 | 0 | -0.85  (-1.07, -0.72) |
| Prevalence | Western Sub-Saharan Africa | Female | Glaucoma | 2015 | 2021 | 0.211957608 | 0.05  (-0.04, 0.19) |
| Prevalence | Western Sub-Saharan Africa | Male | Glaucoma | 1990 | 1993 | 0 | 0.74  (0.58, 0.94) |
| Prevalence | Western Sub-Saharan Africa | Male | Glaucoma | 1993 | 1996 | 0 | -0.53  (-0.68, -0.36) |
| Prevalence | Western Sub-Saharan Africa | Male | Glaucoma | 1996 | 1999 | 0 | -2.93  (-3.05, -2.8) |
| Prevalence | Western Sub-Saharan Africa | Male | Glaucoma | 1999 | 2009 | 0 | -0.45  (-0.49, -0.4) |
| Prevalence | Western Sub-Saharan Africa | Male | Glaucoma | 2009 | 2015 | 0 | -0.86  (-1.07, -0.77) |
| Prevalence | Western Sub-Saharan Africa | Male | Glaucoma | 2015 | 2021 | 0 | -0.55  (-0.64, -0.4) |
| Abbreviations: DALYs, Disability-Adjusted Life Years; APC,annual percentage change; CI, confidence interval; SDI, sociodemographic index. | | | | | | | |

## Supplementary Table 2

| Supplementary Table 2 The prevalence of glaucoma and their AAPCs from 1990 to 2021 at the national and subnational regional level. | | | | | | |
| --- | --- | --- | --- | --- | --- | --- |
|  | Case (n), 1990 | Case (n), 2021 | ASR (per 100, 000), 1990 | ASR (per 100, 000), 2021 | AAPC, 1990-2021 | *p* |
| Afghanistan | 19165.46 (15246.84, 23869.15) | 17420.37 (13845.32, 21418.19) | 335.68 (269.34, 418.18) | 262.73 (212.78, 324.56) | -0.68  (-0.78, -0.58) | 0 |
| Albania | 985.90 (814.78, 1184.22) | 1917.83 (1588.58, 2282.79) | 57.15 (47.41, 68.74) | 43.49 (36.25, 51.28) | -0.88  (-0.9, -0.85) | 0 |
| Algeria | 31235.59 (25463.36, 38074.17) | 64137.43 (53236.07, 76955.18) | 310.23 (258.12, 376.71) | 209.89 (174.66, 250.79) | -1.25  (-1.28, -1.22) | 0 |
| American Samoa | 15.68 (12.75, 18.98) | 29.94 (24.47, 36.52) | 94.40 (77.73, 114.19) | 74.84 (61.82, 90.33) | -0.76  (-0.82, -0.71) | 0 |
| Andorra | 28.19 (23.49, 33.69) | 81.01 (68.33, 94.75) | 57.33 (47.88, 67.69) | 47.39 (39.79, 55.93) | -0.63  (-0.64, -0.61) | 0 |
| Angola | 7537.33 (6150.93, 9124.52) | 17676.12 (14594.42, 21156.78) | 297.19 (247.31, 357.56) | 222.91 (187.07, 267.70) | -0.93  (-0.94, -0.91) | 0 |
| Antigua and Barbuda | 79.07 (65.85, 95.12) | 98.11 (82.15, 116.99) | 135.28 (112.61, 161.78) | 101.49 (84.78, 121.80) | -0.93  (-0.94, -0.91) | 0 |
| Argentina | 24489.51 (20473.75, 29764.36) | 37447.34 (31373.10, 44499.57) | 81.76 (68.89, 99.77) | 63.80 (53.39, 75.52) | -0.79  (-0.83, -0.76) | 0 |
| Armenia | 3072.27 (2540.03, 3714.77) | 4357.80 (3624.54, 5270.08) | 130.14 (108.63, 156.55) | 98.43 (81.94, 118.33) | -0.91  (-0.93, -0.88) | 0 |
| Australia | 10797.37 (9081.54, 12850.76) | 25489.02 (21251.98, 30259.00) | 56.84 (48.03, 66.99) | 49.61 (41.29, 58.83) | -0.43  (-0.46, -0.41) | 0 |
| Austria | 7966.59 (6579.49, 9518.23) | 10750.77 (8917.47, 12740.08) | 63.76 (53.14, 75.01) | 49.17 (40.82, 57.96) | -0.84  (-0.86, -0.83) | 0 |
| Azerbaijan | 5727.33 (4770.88, 6897.87) | 8723.43 (7229.48, 10658.36) | 132.55 (111.35, 160.34) | 104.87 (87.99, 125.48) | -0.76  (-0.81, -0.71) | 0 |
| Bahamas | 173.73 (144.10, 209.89) | 362.18 (303.34, 435.70) | 129.98 (107.94, 157.05) | 102.73 (86.25, 123.70) | -0.76  (-0.79, -0.73) | 0 |
| Bahrain | 316.82 (256.74, 384.32) | 1060.43 (868.75, 1281.28) | 277.36 (229.97, 334.30) | 177.39 (148.51, 212.25) | -1.43  (-1.45, -1.42) | 0 |
| Bangladesh | 50174.43 (40641.12, 60683.83) | 96599.80 (79519.54, 117163.67) | 127.67 (104.66, 154.53) | 77.27 (64.05, 92.91) | -1.62  (-1.65, -1.58) | 0 |
| Barbados | 192.62 (162.92, 231.21) | 256.89 (211.90, 310.64) | 58.96 (50.33, 69.74) | 47.98 (39.60, 57.90) | -0.67  (-0.69, -0.64) | 0 |
| Belarus | 8561.78 (7140.11, 10532.13) | 8906.70 (7358.85, 10794.59) | 68.02 (56.69, 84.19) | 53.56 (44.10, 65.03) | -0.77  (-0.8, -0.74) | 0 |
| Belgium | 10382.43 (8654.89, 12234.09) | 14297.78 (12011.29, 16905.56) | 64.82 (54.29, 75.68) | 50.19 (42.14, 58.83) | -0.82  (-0.83, -0.81) | 0 |
| Belize | 148.08 (123.32, 179.44) | 308.33 (255.89, 373.15) | 164.43 (137.03, 199.17) | 121.58 (101.28, 146.98) | -0.96  (-0.98, -0.94) | 0 |
| Benin | 3888.94 (3254.60, 4622.46) | 11746.46 (9717.11, 14077.44) | 231.64 (194.79, 275.55) | 285.42 (237.36, 342.55) | 0.68  (0.63, 0.72) | 0 |
| Bermuda | 69.13 (57.45, 83.43) | 133.32 (112.21, 160.31) | 118.46 (98.60, 142.66) | 88.47 (74.39, 107.04) | -0.93  (-0.95, -0.92) | 0 |
| Bhutan | 196.75 (161.76, 238.75) | 380.11 (315.30, 462.12) | 112.06 (92.44, 136.95) | 68.80 (57.22, 83.15) | -1.56  (-1.64, -1.49) | 0 |
| Bolivia (Plurinational State of) | 5338.49 (4404.06, 6576.25) | 11569.61 (9481.13, 13996.44) | 205.96 (170.66, 257.11) | 146.25 (120.71, 177.83) | -1.09  (-1.13, -1.06) | 0 |
| Bosnia and Herzegovina | 1851.11 (1523.04, 2223.50) | 2602.86 (2167.62, 3140.97) | 56.07 (46.39, 67.95) | 40.23 (33.57, 48.16) | -1.07  (-1.16, -0.98) | 0 |
| Botswana | 1745.26 (1383.92, 2202.63) | 3066.02 (2491.73, 3766.93) | 426.63 (343.08, 527.80) | 281.00 (229.06, 341.68) | -1.35  (-1.39, -1.3) | 0 |
| Brazil | 159497.19 (136708.81, 185234.50) | 411754.88 (353281.46, 482641.53) | 216.36 (185.57, 252.68) | 169.96 (145.45, 199.61) | -0.82  (-1.09, -0.55) | 0 |
| Brunei Darussalam | 69.11 (58.08, 82.64) | 158.91 (132.36, 190.77) | 91.34 (77.36, 108.62) | 68.38 (57.29, 80.81) | -0.92  (-0.96, -0.88) | 0 |
| Bulgaria | 3914.42 (3359.68, 4524.78) | 4819.94 (3975.69, 5871.04) | 36.73 (31.94, 42.12) | 31.22 (26.06, 37.51) | -0.52  (-0.56, -0.48) | 0 |
| Burkina Faso | 6252.92 (5021.42, 7831.16) | 18789.61 (15167.47, 22690.78) | 199.91 (161.77, 248.28) | 253.63 (206.02, 308.08) | 0.76  (0.64, 0.88) | 0 |
| Burundi | 3400.41 (2811.88, 4098.26) | 6331.73 (5215.78, 7559.84) | 179.06 (150.77, 212.36) | 174.98 (146.68, 206.30) | -0.15  (-0.27, -0.03) | 0.017 |
| Cabo Verde | 453.56 (383.45, 540.16) | 681.87 (567.78, 812.89) | 188.22 (159.94, 222.32) | 168.17 (139.42, 202.45) | -0.39  (-0.45, -0.33) | 0 |
| Cambodia | 3867.26 (3072.65, 4800.33) | 7242.02 (5911.79, 8777.94) | 111.06 (88.91, 135.88) | 71.81 (58.86, 87.03) | -1.37  (-1.42, -1.33) | 0 |
| Cameroon | 9365.60 (7834.05, 11143.57) | 21943.75 (18136.32, 26095.67) | 284.84 (241.09, 337.33) | 236.78 (198.03, 282.09) | -0.57  (-0.68, -0.45) | 0 |
| Canada | 14401.86 (12076.65, 17256.58) | 33741.57 (28554.36, 40295.64) | 44.62 (37.54, 53.39) | 41.25 (34.95, 49.05) | -0.26  (-0.29, -0.24) | 0 |
| Central African Republic | 939.89 (779.77, 1134.46) | 1780.56 (1466.35, 2122.87) | 149.42 (125.21, 180.17) | 151.49 (126.62, 184.43) | 0.04  (0.03, 0.06) | 0 |
| Chad | 6921.56 (5651.13, 8525.08) | 13250.77 (10939.07, 16060.77) | 284.00 (233.60, 345.72) | 303.56 (249.93, 368.01) | 0.22  (0.18, 0.26) | 0 |
| Chile | 7153.31 (5957.66, 8703.31) | 16503.56 (13760.57, 19648.47) | 80.57 (67.53, 97.29) | 62.32 (51.97, 74.33) | -0.83  (-0.86, -0.8) | 0 |
| China | 595000.99 (496854.12, 702738.74) | 1119892.27 (946317.67, 1326306.19) | 90.72 (76.94, 108.01) | 54.91 (46.72, 64.64) | -1.69  (-1.93, -1.44) | 0 |
| Colombia | 24839.67 (20606.39, 29612.10) | 69173.72 (58095.81, 83307.25) | 168.45 (141.04, 202.42) | 123.89 (103.33, 150.27) | -0.99  (-1, -0.97) | 0 |
| Comoros | 478.35 (397.91, 572.34) | 983.14 (814.70, 1171.57) | 310.58 (259.42, 371.03) | 239.02 (200.76, 284.22) | -0.84  (-0.86, -0.82) | 0 |
| Congo | 2055.78 (1693.84, 2506.48) | 4188.77 (3450.20, 5131.71) | 262.69 (221.79, 315.68) | 223.30 (186.63, 271.64) | -0.52  (-0.58, -0.46) | 0 |
| Cook Islands | 16.10 (13.00, 19.41) | 26.10 (21.13, 31.69) | 151.88 (122.66, 185.41) | 99.34 (81.02, 120.62) | -1.39  (-1.45, -1.33) | 0 |
| Costa Rica | 2151.33 (1802.61, 2634.60) | 5651.00 (4667.98, 6781.73) | 133.41 (111.95, 162.76) | 102.19 (84.55, 122.83) | -0.86  (-0.88, -0.84) | 0 |
| Coted'Ivoire | 3516.00 (2931.45, 4267.24) | 22291.12 (18471.54, 27182.91) | 154.29 (129.41, 186.99) | 271.43 (227.83, 329.37) | 1.83  (1.72, 1.94) | 0 |
| Croatia | 2421.26 (2014.18, 2927.30) | 3625.06 (2990.50, 4340.62) | 44.50 (37.49, 53.40) | 36.55 (30.30, 43.69) | -0.63  (-0.65, -0.62) | 0 |
| Cuba | 18843.95 (15486.38, 22997.79) | 29326.42 (24410.64, 35578.72) | 186.59 (155.63, 226.45) | 141.39 (117.70, 170.93) | -0.89  (-0.91, -0.87) | 0 |
| Cyprus | 486.10 (394.84, 587.26) | 994.52 (827.85, 1184.73) | 73.28 (60.44, 86.22) | 48.93 (41.31, 57.97) | -1.3  (-1.32, -1.28) | 0 |
| Czechia | 6134.68 (5081.65, 7407.75) | 8272.99 (6790.68, 9944.56) | 44.91 (37.72, 53.88) | 35.03 (28.99, 41.90) | -0.79  (-0.82, -0.77) | 0 |
| Democratic People's Republic of Korea | 4420.86 (3561.60, 5510.64) | 9407.40 (7365.88, 11864.80) | 36.60 (29.88, 44.81) | 31.21 (24.51, 38.97) | -0.51  (-0.54, -0.47) | 0 |
| Democratic Republic of the Congo | 13337.46 (11025.98, 16266.55) | 32012.11 (26592.81, 37751.15) | 134.64 (112.78, 161.70) | 136.82 (114.62, 161.24) | 0.05  (0.02, 0.07) | 0.001 |
| Denmark | 5215.80 (4457.12, 6212.36) | 6486.00 (5454.85, 7758.18) | 59.16 (50.91, 69.94) | 47.47 (39.95, 56.37) | -0.71  (-0.76, -0.66) | 0 |
| Djibouti | 283.76 (234.39, 339.30) | 1122.34 (924.57, 1360.27) | 309.11 (258.68, 366.71) | 245.49 (204.61, 293.61) | -0.75  (-0.76, -0.73) | 0 |
| Dominica | 87.21 (72.15, 105.54) | 86.55 (72.91, 104.47) | 144.96 (120.73, 174.65) | 109.21 (92.01, 132.58) | -0.91  (-0.92, -0.9) | 0 |
| Dominican Republic | 6353.36 (5268.75, 7694.46) | 13727.76 (11411.46, 16574.59) | 202.70 (169.98, 245.31) | 143.90 (119.66, 174.21) | -1.1  (-1.16, -1.04) | 0 |
| Ecuador | 6228.59 (5189.71, 7549.51) | 15652.13 (13030.96, 18951.87) | 133.97 (111.50, 162.77) | 99.43 (82.65, 119.87) | -0.95  (-1, -0.91) | 0 |
| Egypt | 49047.59 (39909.65, 59994.56) | 99232.89 (80616.76, 120591.61) | 253.54 (210.67, 308.23) | 212.89 (177.41, 254.38) | -0.57  (-0.65, -0.49) | 0 |
| El Salvador | 6342.01 (5282.08, 7654.21) | 10103.42 (8484.98, 12110.51) | 229.80 (191.17, 277.91) | 155.68 (130.28, 187.25) | -1.26  (-1.31, -1.21) | 0 |
| Equatorial Guinea | 413.79 (330.27, 519.25) | 673.84 (555.68, 820.90) | 299.13 (241.68, 373.71) | 190.86 (158.79, 230.26) | -1.44  (-1.47, -1.41) | 0 |
| Eritrea | 2238.94 (1826.22, 2777.41) | 4981.71 (4096.60, 6076.57) | 334.97 (278.68, 405.69) | 261.30 (217.88, 314.21) | -0.8  (-0.85, -0.75) | 0 |
| Estonia | 880.50 (735.34, 1073.63) | 1076.28 (892.70, 1308.74) | 43.52 (36.38, 52.67) | 34.00 (28.21, 40.92) | -0.8  (-0.82, -0.77) | 0 |
| Eswatini | 620.31 (499.98, 762.14) | 900.06 (729.14, 1098.73) | 293.40 (238.90, 357.67) | 223.55 (183.76, 270.88) | -0.88  (-0.9, -0.85) | 0 |
| Ethiopia | 41647.53 (34306.04, 50509.34) | 92388.91 (77281.52, 112037.21) | 293.02 (242.70, 350.58) | 259.82 (219.22, 315.34) | -0.37  (-0.53, -0.22) | 0 |
| Fiji | 359.52 (286.68, 436.52) | 654.55 (532.58, 794.25) | 138.50 (112.57, 168.65) | 107.40 (88.93, 130.19) | -0.86  (-0.97, -0.76) | 0 |
| Finland | 5331.02 (4414.36, 6359.04) | 8098.45 (6762.97, 9669.84) | 73.84 (61.64, 87.16) | 52.31 (43.69, 62.07) | -1.11  (-1.13, -1.09) | 0 |
| France | 49507.37 (40927.64, 59386.71) | 72879.85 (60657.42, 87234.63) | 55.07 (45.80, 64.97) | 41.05 (34.23, 48.80) | -0.94  (-0.98, -0.9) | 0 |
| Gabon | 1154.84 (952.69, 1379.91) | 1461.66 (1215.97, 1761.61) | 238.43 (198.03, 284.59) | 185.17 (155.34, 221.90) | -0.81  (-0.86, -0.77) | 0 |
| Gambia | 362.69 (305.69, 424.30) | 1319.46 (1068.95, 1605.68) | 137.00 (116.13, 160.74) | 166.01 (135.07, 202.25) | 0.65  (0.51, 0.79) | 0 |
| Georgia | 6757.21 (5645.86, 8110.97) | 6495.19 (5423.77, 7776.58) | 114.25 (95.53, 137.57) | 99.97 (83.41, 119.96) | -0.43  (-0.45, -0.41) | 0 |
| Germany | 79172.35 (65969.09, 95467.11) | 108769.93 (90520.44, 132139.01) | 59.23 (49.60, 70.32) | 45.29 (38.01, 53.65) | -0.87  (-0.89, -0.84) | 0 |
| Ghana | 10383.85 (8586.04, 12524.73) | 30321.72 (24633.08, 37211.45) | 232.92 (193.92, 278.72) | 228.22 (188.85, 276.35) | -0.08  (-0.22, 0.06) | 0.279 |
| Greece | 9612.90 (7884.78, 11740.89) | 15659.99 (12887.83, 19186.70) | 64.76 (53.44, 78.13) | 50.33 (41.41, 60.41) | -0.82  (-0.85, -0.8) | 0 |
| Greenland | 12.46 (10.43, 15.12) | 26.97 (22.75, 32.30) | 59.21 (49.59, 70.47) | 52.12 (43.90, 61.70) | -0.44  (-0.47, -0.41) | 0 |
| Grenada | 130.59 (107.04, 159.53) | 109.71 (91.10, 131.80) | 159.78 (131.73, 193.72) | 109.57 (91.74, 131.84) | -1.21  (-1.24, -1.19) | 0 |
| Guam | 48.59 (39.62, 58.80) | 162.93 (134.41, 195.04) | 86.33 (71.40, 105.10) | 73.50 (60.37, 88.43) | -0.56  (-0.67, -0.46) | 0 |
| Guatemala | 5204.31 (4288.78, 6230.80) | 13121.05 (10965.11, 15720.28) | 197.83 (164.55, 238.62) | 130.58 (109.42, 156.00) | -1.33  (-1.38, -1.28) | 0 |
| Guinea | 8493.13 (6921.04, 10251.88) | 14186.63 (11721.86, 17070.79) | 300.59 (249.55, 364.29) | 304.03 (251.06, 365.74) | 0.03  (0.01, 0.06) | 0.012 |
| Guinea-Bissau | 794.86 (653.00, 973.73) | 1387.61 (1133.61, 1708.74) | 282.39 (235.62, 345.41) | 283.18 (234.29, 339.73) | 0.02  (-0.01, 0.05) | 0.242 |
| Guyana | 555.33 (452.51, 675.59) | 652.06 (538.67, 783.71) | 179.03 (147.29, 217.93) | 123.89 (103.22, 150.55) | -1.18  (-1.19, -1.16) | 0 |
| Haiti | 5428.58 (4331.47, 6648.95) | 8682.02 (7057.48, 10469.40) | 226.89 (185.54, 283.75) | 165.74 (137.13, 200.68) | -1  (-1.03, -0.97) | 0 |
| Honduras | 3261.64 (2708.51, 3939.32) | 7534.84 (6238.04, 9101.78) | 192.23 (161.27, 230.12) | 141.83 (118.71, 168.64) | -0.97  (-0.98, -0.96) | 0 |
| Hungary | 10035.52 (8240.30, 12191.60) | 10382.12 (8666.64, 12426.11) | 69.77 (57.52, 84.07) | 48.23 (40.29, 57.67) | -1.18  (-1.22, -1.14) | 0 |
| Iceland | 194.99 (164.86, 229.67) | 357.79 (294.42, 431.49) | 63.51 (53.72, 74.67) | 53.54 (44.16, 64.31) | -0.55  (-0.6, -0.51) | 0 |
| India | 674142.08 (561360.84, 794124.39) | 1372410.29 (1145859.64, 1620636.16) | 187.15 (159.22, 221.15) | 128.22 (108.47, 151.28) | -1.2  (-1.37, -1.02) | 0 |
| Indonesia | 79466.73 (64783.67, 96531.06) | 143151.41 (117161.70, 173893.08) | 106.94 (88.35, 130.03) | 76.07 (63.67, 90.73) | -1.09  (-1.14, -1.04) | 0 |
| Iran (Islamic Republic of) | 87857.00 (74830.73, 104602.23) | 205137.92 (178275.35, 238833.54) | 457.25 (396.38, 532.10) | 306.88 (266.66, 357.80) | -1.25  (-1.29, -1.21) | 0 |
| Iraq | 22478.85 (18706.64, 27002.35) | 37842.20 (30924.39, 44940.82) | 320.49 (265.60, 383.89) | 205.65 (170.04, 246.90) | -1.43  (-1.45, -1.4) | 0 |
| Ireland | 2664.79 (2210.77, 3227.90) | 4103.17 (3453.75, 4838.54) | 67.11 (56.18, 79.93) | 48.19 (40.64, 56.88) | -1.07  (-1.09, -1.05) | 0 |
| Israel | 3231.86 (2670.90, 3890.09) | 7315.00 (6150.02, 8686.89) | 70.14 (58.41, 83.19) | 53.38 (44.81, 63.36) | -0.89  (-0.94, -0.83) | 0 |
| Italy | 141451.50 (117847.40, 167883.16) | 184911.85 (155790.02, 215842.64) | 155.53 (130.62, 182.94) | 102.49 (87.23, 120.07) | -1.36  (-1.43, -1.29) | 0 |
| Jamaica | 2661.36 (2212.60, 3222.83) | 3480.28 (2925.18, 4209.90) | 141.67 (117.92, 171.55) | 108.31 (90.31, 131.03) | -0.86  (-0.89, -0.84) | 0 |
| Japan | 100543.20 (88306.59, 114986.33) | 259039.59 (226868.86, 300428.85) | 62.55 (55.15, 71.37) | 53.10 (46.56, 60.66) | -0.53  (-0.54, -0.51) | 0 |
| Jordan | 2855.74 (2340.88, 3475.78) | 10511.36 (8666.56, 12701.39) | 302.47 (249.55, 365.50) | 189.15 (158.04, 227.10) | -1.51  (-1.56, -1.46) | 0 |
| Kazakhstan | 13740.51 (11348.60, 16682.11) | 14757.36 (12201.94, 17934.82) | 124.59 (104.03, 150.62) | 96.84 (81.16, 115.96) | -0.81  (-0.87, -0.75) | 0 |
| Kenya | 16262.30 (13848.42, 19161.58) | 37346.83 (31614.16, 43706.33) | 242.87 (208.06, 285.11) | 208.61 (178.42, 245.30) | -0.46  (-0.66, -0.27) | 0 |
| Kiribati | 33.60 (26.86, 41.77) | 54.98 (44.42, 67.15) | 122.08 (99.64, 150.02) | 104.58 (85.90, 127.12) | -0.51  (-0.59, -0.44) | 0 |
| Kuwait | 939.95 (783.61, 1113.98) | 3486.22 (2920.00, 4161.84) | 231.40 (192.91, 278.22) | 166.54 (139.71, 200.26) | -1.06  (-1.08, -1.04) | 0 |
| Kyrgyzstan | 3881.89 (3208.83, 4697.70) | 4699.37 (3891.52, 5661.44) | 143.91 (119.57, 174.35) | 120.23 (100.32, 144.34) | -0.58  (-0.65, -0.52) | 0 |
| Lao People's Democratic Republic | 1248.36 (1023.58, 1568.88) | 2230.47 (1844.77, 2698.18) | 81.98 (68.66, 99.56) | 62.91 (52.53, 75.67) | -0.85  (-0.91, -0.79) | 0 |
| Latvia | 2356.84 (1952.37, 2846.49) | 2449.66 (2047.09, 2985.36) | 65.63 (54.53, 78.74) | 53.07 (44.40, 63.56) | -0.68  (-0.7, -0.67) | 0 |
| Lebanon | 4807.11 (3898.01, 5790.14) | 11352.32 (9430.92, 13651.51) | 258.29 (210.99, 310.93) | 180.36 (149.64, 219.38) | -1.16  (-1.2, -1.12) | 0 |
| Lesotho | 2215.34 (1781.58, 2750.40) | 2086.84 (1661.95, 2585.82) | 306.74 (251.17, 381.68) | 248.70 (203.54, 303.49) | -0.68  (-0.69, -0.67) | 0 |
| Liberia | 3197.24 (2632.64, 3896.72) | 3849.40 (3210.05, 4596.28) | 330.31 (275.05, 400.29) | 254.18 (210.26, 305.03) | -0.85  (-0.87, -0.82) | 0 |
| Libya | 6476.34 (5273.98, 7798.30) | 10683.42 (8853.82, 12749.36) | 402.51 (327.29, 494.53) | 261.87 (217.51, 315.19) | -1.14  (-1.47, -0.8) | 0 |
| Lithuania | 2991.22 (2492.55, 3656.52) | 3514.28 (2951.57, 4261.03) | 66.10 (55.15, 80.77) | 52.52 (43.95, 64.16) | -0.75  (-0.76, -0.74) | 0 |
| Luxembourg | 343.78 (285.88, 411.66) | 548.27 (457.92, 652.36) | 63.31 (53.01, 74.81) | 46.22 (38.87, 54.82) | -1.01  (-1.02, -1) | 0 |
| Madagascar | 12167.67 (10201.84, 14585.40) | 19262.59 (15914.84, 23369.44) | 298.64 (253.38, 352.45) | 243.43 (202.74, 290.82) | -0.66  (-0.71, -0.62) | 0 |
| Malawi | 12027.82 (10295.89, 14145.89) | 16549.57 (13637.90, 19714.57) | 407.50 (354.14, 469.31) | 287.58 (239.30, 343.28) | -1.15  (-1.3, -1) | 0 |
| Malaysia | 6541.56 (5183.16, 8194.36) | 15645.73 (12738.45, 19135.03) | 82.00 (64.84, 102.38) | 62.23 (51.41, 75.24) | -0.89  (-0.96, -0.81) | 0 |
| Maldives | 84.02 (68.62, 103.52) | 220.59 (184.41, 266.93) | 141.26 (116.73, 172.06) | 82.81 (68.75, 100.13) | -1.71  (-1.81, -1.61) | 0 |
| Mali | 9549.46 (7975.71, 11259.13) | 21382.22 (17809.38, 25765.42) | 318.07 (266.82, 375.14) | 308.19 (259.76, 372.27) | -0.1  (-0.13, -0.07) | 0 |
| Malta | 277.19 (229.17, 329.42) | 576.93 (482.16, 681.84) | 70.68 (58.68, 83.26) | 50.75 (42.57, 59.52) | -1.07  (-1.09, -1.04) | 0 |
| Marshall Islands | 15.46 (12.22, 19.23) | 21.80 (17.26, 26.70) | 122.17 (99.11, 150.13) | 93.63 (76.93, 113.86) | -0.88  (-0.95, -0.8) | 0 |
| Mauritania | 2559.44 (2116.81, 3119.54) | 4602.31 (3815.43, 5510.87) | 299.62 (252.40, 360.40) | 249.59 (208.23, 298.95) | -0.59  (-0.6, -0.58) | 0 |
| Mauritius | 567.12 (467.43, 692.69) | 1249.68 (1020.19, 1550.82) | 94.19 (78.75, 114.95) | 70.29 (57.81, 86.44) | -0.93  (-0.96, -0.89) | 0 |
| Mexico | 58980.51 (50569.19, 68849.15) | 144748.09 (123584.84, 170243.95) | 164.47 (141.57, 192.28) | 123.52 (105.94, 145.57) | -0.92  (-0.94, -0.9) | 0 |
| Micronesia (Federated States of) | 50.78 (40.23, 62.26) | 52.73 (42.77, 64.22) | 124.32 (100.01, 151.40) | 95.55 (78.25, 114.95) | -0.86  (-0.94, -0.78) | 0 |
| Monaco | 45.73 (38.24, 55.06) | 56.97 (47.86, 68.05) | 55.70 (46.89, 66.29) | 47.34 (39.91, 56.19) | -0.54  (-0.56, -0.52) | 0 |
| Mongolia | 1812.07 (1491.03, 2221.52) | 2349.43 (1949.32, 2847.70) | 202.87 (168.76, 249.07) | 137.40 (114.20, 164.69) | -1.25  (-1.29, -1.21) | 0 |
| Montenegro | 253.29 (210.81, 303.42) | 352.40 (291.37, 427.98) | 43.86 (36.46, 52.75) | 37.68 (31.50, 45.19) | -0.49  (-0.52, -0.46) | 0 |
| Morocco | 30552.36 (26762.48, 34343.42) | 53161.43 (43709.13, 63717.20) | 243.25 (213.38, 275.62) | 173.84 (144.58, 206.19) | -1.08  (-1.13, -1.02) | 0 |
| Mozambique | 12058.63 (9760.56, 14758.78) | 17858.99 (14906.20, 21612.91) | 263.90 (216.90, 323.37) | 216.76 (183.97, 258.11) | -0.65  (-0.72, -0.57) | 0 |
| Myanmar | 22868.65 (17729.31, 28768.70) | 36478.04 (28917.61, 45286.40) | 127.35 (99.82, 160.06) | 88.06 (69.91, 108.73) | -1.19  (-1.28, -1.1) | 0 |
| Namibia | 1404.82 (1112.56, 1744.11) | 2242.60 (1824.23, 2713.06) | 287.55 (232.53, 356.03) | 211.21 (175.05, 256.77) | -0.99  (-1.02, -0.95) | 0 |
| Nauru | 3.73 (3.00, 4.65) | 3.91 (3.14, 4.80) | 119.53 (97.29, 146.37) | 92.71 (76.48, 111.67) | -0.83  (-0.93, -0.74) | 0 |
| Nepal | 8267.06 (7071.85, 9517.59) | 14895.62 (12627.23, 17232.55) | 119.07 (103.01, 137.63) | 73.28 (62.50, 85.18) | -1.57  (-1.64, -1.5) | 0 |
| Netherlands | 10651.57 (8911.76, 12672.60) | 16776.50 (14021.65, 20009.63) | 51.68 (43.52, 61.05) | 42.34 (35.46, 50.39) | -0.65  (-0.68, -0.62) | 0 |
| New Zealand | 2547.36 (2211.31, 2967.54) | 4896.92 (4212.25, 5684.17) | 65.84 (57.41, 76.45) | 53.01 (45.49, 61.42) | -0.69  (-0.71, -0.67) | 0 |
| Nicaragua | 2421.67 (2023.30, 2915.72) | 5997.41 (4982.95, 7242.21) | 187.08 (156.69, 226.74) | 136.70 (114.00, 165.15) | -1.01  (-1.01, -1) | 0 |
| Niger | 5964.07 (4803.63, 7420.41) | 21935.40 (17935.74, 26604.54) | 304.19 (248.78, 373.67) | 345.65 (284.89, 421.85) | 0.42  (0.37, 0.47) | 0 |
| Nigeria | 166240.91 (142140.93, 192692.55) | 230434.30 (197005.07, 267654.13) | 443.07 (381.68, 511.55) | 320.85 (275.77, 372.20) | -1.05  (-1.12, -0.98) | 0 |
| Niue | 2.36 (1.91, 2.85) | 1.59 (1.31, 1.94) | 99.67 (80.78, 121.22) | 76.01 (62.93, 92.09) | -0.88  (-0.92, -0.84) | 0 |
| North Macedonia | 861.29 (711.73, 1020.73) | 1173.12 (961.36, 1438.21) | 52.99 (43.95, 63.84) | 40.14 (33.56, 48.24) | -0.89  (-0.93, -0.86) | 0 |
| Northern Mariana Islands | 8.89 (7.21, 10.90) | 30.22 (24.65, 36.72) | 87.86 (72.53, 106.79) | 74.12 (61.83, 88.73) | -0.57  (-0.61, -0.53) | 0 |
| Norway | 3993.56 (3424.97, 4690.71) | 5223.65 (4493.45, 6074.25) | 52.23 (44.99, 60.74) | 44.75 (38.53, 51.99) | -0.5  (-0.52, -0.48) | 0 |
| Oman | 1457.00 (1171.50, 1803.76) | 3298.04 (2683.21, 3979.47) | 279.34 (223.51, 345.22) | 229.26 (188.63, 273.94) | -0.63  (-0.66, -0.61) | 0 |
| Pakistan | 70423.41 (58703.02, 84231.06) | 137343.71 (114056.41, 162803.42) | 143.40 (120.86, 171.68) | 139.14 (117.95, 164.75) | -0.11  (-0.16, -0.07) | 0 |
| Palau | 7.90 (6.38, 9.65) | 13.94 (11.37, 17.08) | 97.86 (80.59, 119.44) | 78.69 (64.85, 94.40) | -0.72  (-0.77, -0.66) | 0 |
| Palestine | 1705.95 (1361.13, 2094.85) | 2813.32 (2316.20, 3431.68) | 227.83 (183.08, 277.92) | 144.77 (119.25, 176.72) | -1.47  (-1.55, -1.39) | 0 |
| Panama | 1964.30 (1633.39, 2358.65) | 4701.72 (3931.05, 5603.39) | 144.49 (120.42, 173.67) | 105.06 (87.63, 125.62) | -1.03  (-1.06, -0.99) | 0 |
| Papua New Guinea | 1341.80 (1066.50, 1655.68) | 3295.27 (2681.64, 4002.92) | 105.39 (85.00, 129.42) | 90.52 (74.66, 108.93) | -0.49  (-0.55, -0.44) | 0 |
| Paraguay | 3893.56 (3344.98, 4610.72) | 8674.04 (7356.02, 10389.84) | 194.64 (167.44, 229.94) | 161.92 (137.13, 193.64) | -0.6  (-0.63, -0.56) | 0 |
| Peru | 23042.86 (19121.27, 28050.62) | 50141.25 (41677.74, 60298.91) | 217.15 (180.24, 265.44) | 153.44 (127.75, 184.83) | -1.12  (-1.16, -1.08) | 0 |
| Philippines | 24271.28 (20438.14, 28646.95) | 59754.30 (50599.00, 70470.29) | 105.60 (89.83, 125.04) | 88.66 (75.29, 104.17) | -0.57  (-0.59, -0.54) | 0 |
| Poland | 18665.34 (15994.54, 21885.66) | 29154.53 (25149.51, 33889.68) | 44.92 (38.78, 51.97) | 37.60 (32.39, 43.74) | -0.57  (-0.59, -0.56) | 0 |
| Portugal | 10547.15 (8633.17, 12766.52) | 16305.42 (13670.54, 19593.50) | 80.32 (66.77, 96.07) | 53.59 (44.97, 63.61) | -1.3  (-1.36, -1.24) | 0 |
| Puerto Rico | 4391.50 (3634.24, 5293.09) | 7586.46 (6366.48, 9100.62) | 121.74 (101.15, 145.87) | 88.49 (73.65, 106.47) | -1.02  (-1.03, -1.01) | 0 |
| Qatar | 217.83 (177.19, 266.89) | 1077.48 (879.85, 1309.25) | 371.20 (305.42, 450.14) | 216.19 (180.24, 259.71) | -1.8  (-2.05, -1.55) | 0 |
| Republic of Korea | 21836.51 (18240.71, 26366.27) | 57623.02 (49020.89, 68458.92) | 100.55 (84.82, 120.21) | 61.10 (52.03, 72.27) | -1.61  (-1.63, -1.58) | 0 |
| Republic of Moldova | 2902.21 (2417.55, 3541.73) | 3864.02 (3282.43, 4616.31) | 76.62 (64.44, 93.29) | 62.99 (53.60, 75.18) | -0.63  (-0.65, -0.61) | 0 |
| Romania | 13183.89 (10789.81, 16070.93) | 16658.01 (13814.50, 20145.36) | 53.17 (43.89, 64.30) | 40.60 (33.75, 48.88) | -0.86  (-0.89, -0.84) | 0 |
| Russian Federation | 162556.80 (140579.44, 190668.98) | 184206.91 (159355.23, 216200.58) | 98.02 (85.42, 115.90) | 75.01 (65.04, 87.98) | -0.87  (-0.89, -0.85) | 0 |
| Rwanda | 5696.65 (4742.51, 6863.80) | 9800.27 (8089.16, 11826.60) | 267.36 (225.36, 320.33) | 199.59 (165.92, 238.11) | -0.94  (-0.96, -0.91) | 0 |
| Saint Kitts and Nevis | 60.01 (49.10, 73.86) | 58.97 (49.00, 71.27) | 151.72 (126.20, 184.89) | 105.22 (87.92, 125.88) | -1.17  (-1.19, -1.16) | 0 |
| Saint Lucia | 126.42 (104.31, 153.72) | 252.10 (211.00, 305.56) | 152.48 (127.50, 185.22) | 107.62 (90.06, 130.37) | -1.11  (-1.14, -1.08) | 0 |
| Saint Vincent and the Grenadines | 109.30 (90.59, 132.19) | 160.52 (132.96, 194.39) | 157.03 (131.69, 189.29) | 116.81 (97.25, 142.65) | -0.94  (-0.96, -0.93) | 0 |
| Samoa | 76.77 (61.63, 93.26) | 108.34 (88.40, 131.00) | 108.11 (88.10, 132.01) | 87.64 (72.03, 106.27) | -0.72  (-0.75, -0.69) | 0 |
| San Marino | 21.31 (17.86, 25.46) | 47.62 (39.52, 57.07) | 56.54 (47.59, 66.95) | 48.17 (40.11, 56.99) | -0.52  (-0.55, -0.5) | 0 |
| Sao Tome and Principe | 187.61 (154.51, 227.43) | 221.30 (182.07, 268.07) | 318.55 (264.37, 383.56) | 249.70 (207.28, 300.83) | -0.79  (-0.8, -0.78) | 0 |
| Saudi Arabia | 16843.39 (14281.13, 19810.44) | 31037.30 (25536.41, 37906.48) | 374.84 (321.24, 446.91) | 260.25 (215.15, 314.69) | -1.19  (-1.25, -1.13) | 0 |
| Senegal | 9032.73 (7486.38, 10829.96) | 18437.47 (15227.76, 22221.66) | 342.73 (284.36, 414.23) | 287.54 (240.95, 344.19) | -0.51  (-0.56, -0.45) | 0 |
| Serbia | 4706.13 (3880.73, 5658.00) | 6820.03 (5688.64, 8202.36) | 50.09 (41.82, 59.81) | 38.59 (32.41, 46.16) | -0.83  (-0.85, -0.81) | 0 |
| Seychelles | 54.41 (45.25, 66.31) | 74.96 (61.63, 91.50) | 96.12 (79.98, 117.14) | 72.37 (59.91, 88.63) | -0.91  (-0.95, -0.86) | 0 |
| Sierra Leone | 5838.10 (4699.64, 7153.90) | 8042.88 (6711.82, 9689.97) | 336.57 (274.72, 417.86) | 277.17 (232.58, 335.56) | -0.62  (-0.73, -0.52) | 0 |
| Singapore | 1702.69 (1435.03, 2023.38) | 6268.40 (5267.86, 7355.14) | 97.93 (83.26, 116.19) | 75.46 (63.43, 88.62) | -0.83  (-0.86, -0.8) | 0 |
| Slovakia | 2762.57 (2288.74, 3328.78) | 3741.18 (3127.91, 4476.64) | 47.81 (39.99, 57.20) | 38.19 (31.89, 45.22) | -0.72  (-0.74, -0.7) | 0 |
| Slovenia | 1036.23 (863.61, 1265.21) | 1768.20 (1466.99, 2108.21) | 42.80 (35.83, 51.95) | 34.86 (28.91, 41.91) | -0.66  (-0.68, -0.63) | 0 |
| Solomon Islands | 137.35 (109.41, 169.53) | 284.98 (231.07, 345.40) | 141.69 (113.53, 172.67) | 112.70 (92.67, 137.64) | -0.75  (-0.81, -0.69) | 0 |
| Somalia | 3518.94 (2861.52, 4235.41) | 8969.96 (7244.77, 11020.72) | 232.70 (190.92, 280.97) | 222.97 (183.29, 271.65) | -0.14  (-0.17, -0.12) | 0 |
| South Africa | 41266.78 (35198.84, 49093.57) | 78587.16 (66715.02, 92765.30) | 232.73 (198.96, 278.13) | 200.69 (170.51, 238.81) | -0.46  (-0.64, -0.27) | 0 |
| South Sudan | 4411.18 (3536.47, 5547.90) | 4173.27 (3406.07, 5094.89) | 211.94 (171.15, 262.89) | 160.23 (130.23, 197.49) | -0.86  (-0.93, -0.78) | 0 |
| Spain | 80045.42 (66187.52, 96557.13) | 124853.77 (106524.25, 146285.64) | 145.33 (120.26, 172.63) | 103.50 (88.32, 119.81) | -1.1  (-1.15, -1.04) | 0 |
| Sri Lanka | 8350.30 (6760.79, 10155.31) | 18358.68 (14822.79, 22792.23) | 94.45 (77.08, 115.25) | 70.29 (57.61, 86.23) | -0.94  (-1.02, -0.86) | 0 |
| Sudan | 31881.76 (25890.84, 39320.74) | 39089.02 (32241.01, 46481.73) | 415.78 (345.52, 511.19) | 255.84 (213.37, 309.69) | -1.46  (-1.51, -1.4) | 0 |
| Suriname | 434.70 (359.26, 522.66) | 863.89 (735.30, 1017.85) | 193.61 (159.79, 235.40) | 145.97 (124.14, 171.78) | -0.91  (-0.94, -0.88) | 0 |
| Sweden | 4288.17 (3583.55, 5080.76) | 6346.19 (5317.75, 7555.33) | 25.29 (21.16, 29.58) | 23.46 (19.73, 27.89) | -0.2  (-0.24, -0.16) | 0 |
| Switzerland | 6142.68 (5071.83, 7359.30) | 9942.26 (8324.76, 11919.09) | 53.93 (44.77, 64.33) | 44.75 (37.41, 52.40) | -0.6  (-0.62, -0.59) | 0 |
| Syrian Arab Republic | 14798.47 (12117.88, 17852.97) | 23457.78 (19122.30, 28152.17) | 347.93 (285.94, 423.76) | 214.92 (178.24, 257.76) | -1.54  (-1.59, -1.5) | 0 |
| Taiwan (Province of China) | 3478.61 (2451.35, 4500.63) | 7835.74 (5952.22, 10429.50) | 27.49 (19.77, 35.06) | 17.63 (13.34, 23.60) | -1.43  (-1.61, -1.25) | 0 |
| Tajikistan | 3668.42 (3038.88, 4437.24) | 5584.00 (4598.67, 6782.55) | 151.45 (125.53, 183.29) | 128.73 (107.85, 155.86) | -0.53  (-0.58, -0.48) | 0 |
| Thailand | 26127.85 (22198.40, 31019.33) | 72560.78 (59978.65, 88007.35) | 90.82 (77.60, 106.77) | 65.60 (54.08, 79.62) | -1.04  (-1.07, -1.01) | 0 |
| Timor-Leste | 399.98 (324.53, 495.45) | 958.14 (787.50, 1184.00) | 224.68 (181.15, 279.17) | 131.37 (109.16, 161.40) | -1.65  (-1.7, -1.59) | 0 |
| Togo | 2252.34 (1868.64, 2760.91) | 7351.00 (6023.33, 8906.55) | 248.64 (208.83, 300.22) | 260.92 (218.09, 314.57) | 0.14  (0.09, 0.2) | 0 |
| Tokelau | 1.52 (1.21, 1.88) | 1.30 (1.07, 1.58) | 118.10 (96.27, 145.10) | 85.89 (71.09, 104.16) | -1.04  (-1.08, -0.99) | 0 |
| Tonga | 50.23 (42.49, 59.89) | 59.14 (49.04, 71.76) | 108.09 (92.10, 127.98) | 78.36 (64.92, 95.73) | -1.05  (-1.2, -0.89) | 0 |
| Trinidad and Tobago | 1056.64 (859.08, 1280.01) | 1852.39 (1558.23, 2193.52) | 138.18 (115.20, 165.77) | 97.66 (82.58, 115.11) | -1.12  (-1.14, -1.09) | 0 |
| Tunisia | 14030.18 (11369.40, 17236.34) | 22494.55 (18363.41, 27009.86) | 330.29 (271.11, 401.92) | 182.43 (149.77, 222.59) | -1.91  (-1.92, -1.89) | 0 |
| Turkey | 58134.26 (48281.41, 71051.07) | 116000.86 (96725.01, 139766.45) | 201.73 (168.24, 248.35) | 131.36 (110.11, 157.52) | -1.38  (-1.42, -1.34) | 0 |
| Turkmenistan | 1534.40 (1292.73, 1792.19) | 2561.12 (2091.50, 3093.63) | 96.59 (81.76, 113.14) | 76.56 (63.97, 92.09) | -0.75  (-0.79, -0.71) | 0 |
| Tuvalu | 6.66 (5.23, 8.31) | 8.51 (6.89, 10.34) | 124.84 (101.88, 152.31) | 93.64 (77.17, 113.69) | -0.94  (-1.01, -0.87) | 0 |
| Uganda | 14758.31 (12684.12, 17261.67) | 20223.51 (16924.57, 23859.97) | 295.39 (254.01, 342.36) | 179.81 (151.41, 212.03) | -1.47  (-1.68, -1.26) | 0 |
| Ukraine | 46648.68 (40161.05, 55152.54) | 48861.21 (41808.04, 58122.57) | 68.24 (59.11, 80.43) | 59.76 (51.48, 70.80) | -0.43  (-0.46, -0.4) | 0 |
| United Arab Emirates | 695.46 (569.93, 841.84) | 3803.01 (3024.39, 4744.13) | 283.06 (234.45, 346.00) | 194.01 (162.38, 232.15) | -1.21  (-1.25, -1.18) | 0 |
| United Kingdom | 61864.86 (52813.96, 72025.37) | 79427.66 (67902.14, 92156.29) | 64.30 (55.43, 74.26) | 52.25 (44.68, 60.10) | -0.68  (-0.71, -0.64) | 0 |
| United Republic of Tanzania | 31160.09 (25796.59, 37207.37) | 55861.74 (46997.68, 66056.41) | 362.92 (304.85, 427.88) | 272.92 (230.79, 322.70) | -0.93  (-1.07, -0.78) | 0 |
| United States of America | 87.75 (73.28, 105.63) | 195.02 (161.50, 236.88) | 127.54 (107.12, 153.14) | 97.80 (81.98, 117.14) | -0.19  (-0.26, -0.12) | 0 |
| United States Virgin Islands | 158492.51 (137698.86, 184238.95) | 283454.61 (246097.45, 327017.77) | 46.64 (40.70, 53.87) | 43.94 (38.00, 50.84) | -0.85  (-0.88, -0.82) | 0 |
| Uruguay | 3044.84 (2522.38, 3686.42) | 4220.41 (3537.77, 5044.81) | 76.98 (64.36, 92.48) | 65.31 (54.50, 78.68) | -0.53  (-0.54, -0.52) | 0 |
| Uzbekistan | 14947.00 (12463.43, 18171.15) | 23109.87 (19065.65, 27728.60) | 141.07 (117.75, 172.00) | 111.45 (92.86, 135.22) | -0.76  (-0.77, -0.75) | 0 |
| Vanuatu | 43.22 (36.12, 51.77) | 98.09 (79.51, 118.76) | 96.66 (81.33, 115.06) | 75.00 (61.96, 89.19) | -0.84  (-0.9, -0.78) | 0 |
| Venezuela (Bolivarian Republic of) | 13338.89 (11012.25, 16020.24) | 38279.25 (31884.82, 46460.18) | 161.75 (134.00, 196.26) | 136.65 (113.55, 165.54) | -0.55  (-0.63, -0.46) | 0 |
| Viet Nam | 37679.96 (31527.63, 44421.64) | 64081.79 (54045.50, 74521.31) | 105.88 (89.41, 125.08) | 74.43 (62.99, 86.70) | -1.15  (-1.22, -1.08) | 0 |
| Yemen | 6873.98 (5586.68, 8439.64) | 19074.82 (15649.71, 23448.10) | 191.45 (158.08, 233.74) | 178.32 (148.59, 217.87) | -0.23  (-0.31, -0.15) | 0 |
| Zambia | 6668.53 (5517.33, 8006.54) | 12036.39 (9868.12, 14383.10) | 303.74 (255.90, 363.40) | 232.47 (193.43, 278.01) | -0.85  (-0.9, -0.8) | 0 |
| Zimbabwe | 6515.05 (5420.94, 7934.58) | 9121.20 (7525.29, 11105.64) | 203.36 (172.37, 244.19) | 183.41 (153.10, 220.79) | -0.34  (-0.38, -0.3) | 0 |
| Geographic Standardization Note: All geographic and administrative divisions in this study comply with the one-China principle and the coding standards of the Global Burden of Disease (GBD) Study and World Health Organization (WHO). Taiwan (Province of China) is included as a provincial-level administrative region of the People's Republic of China. Displayed location-specific estimates may not sum exactly to aggregate estimates because of GBD estimation and rounding. | | | | | | |

## Supplementary Table 3

| Supplementary Table 3 The DALYs of glaucoma and their AAPCs from 1990 to 2021 at the national and subnational regional level | | | | | | |
| --- | --- | --- | --- | --- | --- | --- |
|  | Case (n), 1990 | Case (n), 2021 | ASR (per 100, 000), 1990 | ASR (per 100, 000), 2021 | AAPC, 1990-2021 | *P* |
| Afghanistan | 2662.86 (1734.88, 3878.24) | 2267.91 (1474.23, 3203.63) | 46.45 (30.48, 66.51) | 34.18 (22.45, 48.17) | -0.91  (-1.03, -0.79) | 0 |
| Albania | 110.42 (74.53, 159.72) | 188.35 (124.85, 264.01) | 6.41 (4.32, 9.20) | 4.30 (2.87, 6.00) | -1.29  (-1.34, -1.23) | 0 |
| Algeria | 3901.77 (2633.52, 5601.34) | 7092.86 (4762.00, 10064.84) | 39.09 (26.52, 55.03) | 23.47 (15.78, 33.04) | -1.62  (-1.66, -1.59) | 0 |
| American Samoa | 1.89 (1.25, 2.77) | 3.18 (2.10, 4.55) | 11.30 (7.37, 16.20) | 8.01 (5.39, 11.15) | -1.14  (-1.27, -1.01) | 0 |
| Andorra | 3.37 (2.19, 4.81) | 8.84 (6.00, 12.64) | 6.74 (4.40, 9.45) | 5.21 (3.52, 7.46) | -0.84  (-0.86, -0.82) | 0 |
| Angola | 832.33 (559.15, 1215.31) | 1673.44 (1124.97, 2388.63) | 32.63 (21.90, 46.60) | 21.12 (14.26, 30.07) | -1.4  (-1.43, -1.38) | 0 |
| Antigua and Barbuda | 9.23 (6.17, 12.96) | 9.88 (6.60, 13.68) | 15.80 (10.62, 22.18) | 10.32 (6.93, 14.36) | -1.37  (-1.4, -1.34) | 0 |
| Argentina | 2633.30 (1762.49, 3683.49) | 3521.79 (2385.46, 4848.54) | 8.81 (5.89, 12.47) | 6.00 (4.06, 8.28) | -1.23  (-1.27, -1.18) | 0 |
| Armenia | 358.73 (239.59, 510.62) | 451.34 (304.31, 637.79) | 15.18 (10.23, 21.54) | 10.22 (6.90, 14.41) | -1.28  (-1.33, -1.23) | 0 |
| Australia | 1150.91 (787.70, 1601.81) | 2471.58 (1646.72, 3400.41) | 6.05 (4.16, 8.34) | 4.84 (3.26, 6.73) | -0.72  (-0.77, -0.66) | 0 |
| Austria | 948.40 (630.21, 1353.81) | 1176.32 (795.45, 1661.99) | 7.63 (5.19, 10.89) | 5.45 (3.68, 7.68) | -1.08  (-1.13, -1.04) | 0 |
| Azerbaijan | 684.53 (452.49, 983.69) | 934.99 (638.36, 1300.37) | 15.82 (10.41, 22.76) | 11.33 (7.60, 15.93) | -1.08  (-1.12, -1.03) | 0 |
| Bahamas | 20.11 (13.49, 28.84) | 37.24 (25.07, 53.19) | 15.11 (10.23, 21.55) | 10.63 (7.09, 15.17) | -1.15  (-1.22, -1.08) | 0 |
| Bahrain | 38.57 (26.07, 56.22) | 105.73 (72.69, 150.35) | 34.00 (22.61, 48.13) | 18.38 (12.47, 25.37) | -1.96  (-2, -1.93) | 0 |
| Bangladesh | 5820.83 (3829.22, 8118.39) | 9829.72 (6658.79, 13890.62) | 14.92 (9.82, 20.77) | 7.98 (5.45, 11.31) | -2.01  (-2.08, -1.94) | 0 |
| Barbados | 21.78 (14.77, 30.17) | 25.71 (17.41, 35.52) | 6.70 (4.59, 9.24) | 4.82 (3.27, 6.64) | -1.06  (-1.12, -1) | 0 |
| Belarus | 918.34 (620.34, 1317.53) | 834.08 (560.62, 1167.14) | 7.32 (4.96, 10.50) | 5.02 (3.37, 7.01) | -1.2  (-1.27, -1.14) | 0 |
| Belgium | 1252.02 (835.84, 1766.58) | 1572.97 (1055.48, 2217.66) | 7.84 (5.29, 11.05) | 5.61 (3.71, 7.95) | -1.07  (-1.14, -1) | 0 |
| Belize | 18.49 (12.17, 26.34) | 34.20 (22.48, 48.76) | 20.53 (13.48, 29.18) | 13.50 (8.84, 19.25) | -1.33  (-1.37, -1.29) | 0 |
| Benin | 487.34 (321.98, 678.56) | 1301.29 (880.74, 1788.49) | 29.22 (19.51, 40.66) | 32.12 (21.40, 43.93) | 0.31  (0.2, 0.43) | 0 |
| Bermuda | 7.69 (5.13, 10.99) | 12.60 (8.67, 17.75) | 13.24 (8.82, 18.72) | 8.32 (5.75, 11.68) | -1.49  (-1.52, -1.47) | 0 |
| Bhutan | 20.87 (14.06, 29.66) | 33.60 (22.90, 45.77) | 12.08 (7.94, 17.12) | 6.12 (4.16, 8.31) | -2.18  (-2.29, -2.08) | 0 |
| Bolivia (Plurinational State of) | 645.80 (423.60, 921.48) | 1223.67 (812.17, 1747.20) | 25.18 (16.49, 35.54) | 15.75 (10.50, 22.16) | -1.5  (-1.55, -1.45) | 0 |
| Bosnia and Herzegovina | 204.00 (132.61, 295.53) | 242.66 (162.86, 340.89) | 6.20 (4.14, 8.83) | 3.77 (2.55, 5.26) | -1.61  (-1.72, -1.5) | 0 |
| Botswana | 257.14 (168.25, 377.59) | 418.65 (280.31, 599.83) | 62.24 (42.30, 89.43) | 38.31 (25.90, 54.37) | -1.56  (-1.6, -1.51) | 0 |
| Brazil | 16293.89 (11298.37, 22353.73) | 37997.10 (26357.51, 51285.58) | 22.40 (15.51, 30.49) | 15.76 (10.87, 21.19) | -1.11  (-1.16, -1.05) | 0 |
| Brunei Darussalam | 8.54 (5.77, 12.10) | 17.89 (12.17, 25.47) | 11.12 (7.56, 15.48) | 7.51 (5.14, 10.49) | -1.26  (-1.3, -1.22) | 0 |
| Bulgaria | 381.11 (260.01, 530.25) | 410.12 (273.34, 577.62) | 3.65 (2.49, 5.08) | 2.67 (1.79, 3.74) | -1  (-1.05, -0.94) | 0 |
| Burkina Faso | 788.04 (516.33, 1145.67) | 2196.12 (1457.28, 3185.96) | 25.60 (16.67, 36.84) | 30.16 (19.92, 43.53) | 0.5  (0.32, 0.67) | 0 |
| Burundi | 352.60 (243.23, 487.28) | 620.41 (431.95, 861.06) | 18.80 (13.20, 25.85) | 17.42 (12.00, 24.11) | -0.28  (-0.3, -0.25) | 0 |
| Cabo Verde | 59.13 (39.23, 80.93) | 74.08 (49.64, 102.99) | 24.46 (16.35, 33.63) | 18.39 (12.29, 25.60) | -0.93  (-0.99, -0.86) | 0 |
| Cambodia | 469.70 (313.78, 690.99) | 768.88 (505.04, 1095.45) | 13.55 (9.04, 19.38) | 7.79 (5.11, 11.02) | -1.74  (-1.79, -1.7) | 0 |
| Cameroon | 1016.69 (686.21, 1418.68) | 2233.94 (1532.07, 3151.93) | 31.98 (21.49, 44.39) | 24.98 (17.20, 35.13) | -0.76  (-0.87, -0.66) | 0 |
| Canada | 1580.89 (1067.65, 2256.91) | 3574.87 (2428.40, 4944.45) | 4.90 (3.34, 6.95) | 4.38 (3.00, 6.06) | -0.36  (-0.39, -0.34) | 0 |
| Central African Republic | 96.35 (64.93, 137.74) | 188.83 (126.32, 270.90) | 15.91 (10.68, 22.22) | 16.35 (11.04, 22.88) | 0.1  (0.03, 0.16) | 0.004 |
| Chad | 907.53 (593.82, 1293.82) | 1523.73 (1016.76, 2123.00) | 37.40 (24.27, 52.62) | 35.53 (23.66, 49.60) | -0.17  (-0.2, -0.14) | 0 |
| Chile | 757.76 (498.82, 1086.46) | 1523.94 (1035.64, 2135.60) | 8.57 (5.68, 12.29) | 5.75 (3.90, 8.05) | -1.27  (-1.31, -1.23) | 0 |
| China | 69104.02 (47537.58, 95916.96) | 100083.57 (67640.48, 139235.89) | 10.49 (7.19, 14.53) | 4.91 (3.35, 6.85) | -2.46  (-2.8, -2.11) | 0 |
| Colombia | 2615.28 (1734.95, 3682.78) | 6143.42 (4167.90, 8462.49) | 17.84 (11.84, 25.35) | 10.94 (7.40, 15.12) | -1.56  (-1.59, -1.53) | 0 |
| Comoros | 55.65 (37.48, 78.11) | 103.34 (70.15, 145.46) | 36.58 (24.48, 50.46) | 25.47 (17.21, 35.98) | -1.15  (-1.18, -1.12) | 0 |
| Congo | 179.04 (122.91, 252.04) | 304.48 (210.15, 421.60) | 23.21 (16.00, 32.40) | 16.54 (11.47, 22.66) | -1.09  (-1.14, -1.05) | 0 |
| Cook Islands | 2.21 (1.47, 3.14) | 3.16 (2.05, 4.49) | 20.61 (13.55, 29.05) | 12.01 (7.85, 17.02) | -1.77  (-1.85, -1.68) | 0 |
| Costa Rica | 231.06 (155.85, 331.45) | 524.73 (354.91, 730.67) | 14.38 (9.64, 20.70) | 9.47 (6.36, 13.25) | -1.34  (-1.37, -1.31) | 0 |
| Coted'Ivoire | 407.13 (271.57, 575.34) | 2315.12 (1576.28, 3263.53) | 18.42 (12.12, 25.58) | 29.13 (19.55, 40.67) | 1.51  (1.31, 1.71) | 0 |
| Croatia | 238.57 (159.77, 342.66) | 317.05 (221.41, 435.04) | 4.41 (2.98, 6.37) | 3.19 (2.27, 4.43) | -1.04  (-1.08, -0.99) | 0 |
| Cuba | 2279.39 (1486.79, 3277.22) | 3174.74 (2123.12, 4476.71) | 22.63 (14.90, 32.16) | 15.26 (10.15, 21.41) | -1.26  (-1.3, -1.21) | 0 |
| Cyprus | 62.36 (41.49, 91.31) | 111.18 (73.65, 157.30) | 9.17 (6.20, 13.18) | 5.45 (3.67, 7.66) | -1.67  (-1.73, -1.6) | 0 |
| Czechia | 600.71 (404.05, 842.08) | 706.03 (476.55, 977.44) | 4.42 (3.01, 6.27) | 2.98 (2.02, 4.12) | -1.26  (-1.32, -1.19) | 0 |
| Democratic People's Republic of Korea | 292.32 (197.09, 417.40) | 471.48 (315.85, 682.85) | 2.46 (1.67, 3.51) | 1.58 (1.06, 2.28) | -1.43  (-1.47, -1.39) | 0 |
| Democratic Republic of the Congo | 1162.71 (779.16, 1639.54) | 2726.60 (1880.52, 3816.12) | 12.35 (8.51, 16.87) | 12.11 (8.40, 16.37) | -0.08  (-0.16, 0) | 0.062 |
| Denmark | 600.20 (408.44, 824.13) | 700.60 (481.48, 986.17) | 6.85 (4.69, 9.38) | 5.16 (3.56, 7.24) | -0.91  (-1, -0.83) | 0 |
| Djibouti | 33.07 (22.24, 48.08) | 117.96 (77.89, 167.18) | 36.49 (24.44, 51.46) | 26.42 (17.80, 37.04) | -1.04  (-1.06, -1.01) | 0 |
| Dominica | 10.45 (6.86, 14.94) | 9.17 (6.05, 12.76) | 17.46 (11.45, 24.68) | 11.63 (7.68, 16.11) | -1.3  (-1.34, -1.26) | 0 |
| Dominican Republic | 786.77 (515.72, 1125.64) | 1484.19 (1008.69, 2079.60) | 25.15 (16.52, 35.36) | 15.56 (10.57, 21.76) | -1.54  (-1.61, -1.47) | 0 |
| Ecuador | 716.52 (474.15, 1018.63) | 1564.51 (1054.01, 2201.20) | 15.52 (10.27, 22.09) | 10.00 (6.77, 13.97) | -1.4  (-1.47, -1.33) | 0 |
| Egypt | 6201.48 (4160.26, 8946.01) | 10688.94 (7158.82, 15060.68) | 32.18 (21.57, 45.87) | 23.46 (15.48, 32.61) | -1.02  (-1.16, -0.88) | 0 |
| El Salvador | 688.94 (461.53, 988.70) | 888.77 (610.38, 1198.31) | 24.98 (16.66, 35.73) | 13.52 (9.27, 18.24) | -1.97  (-2, -1.93) | 0 |
| Equatorial Guinea | 49.00 (31.43, 72.20) | 64.74 (43.32, 90.85) | 34.90 (23.12, 49.89) | 18.32 (11.99, 25.81) | -2.06  (-2.11, -2.02) | 0 |
| Eritrea | 270.85 (182.32, 387.76) | 540.52 (363.78, 778.17) | 41.12 (27.74, 57.25) | 29.03 (19.66, 40.84) | -1.1  (-1.19, -1.01) | 0 |
| Estonia | 92.71 (62.76, 133.68) | 98.74 (65.81, 136.72) | 4.60 (3.15, 6.64) | 3.11 (2.10, 4.25) | -1.26  (-1.33, -1.19) | 0 |
| Eswatini | 83.61 (54.80, 123.61) | 110.21 (73.61, 161.38) | 39.28 (25.63, 56.59) | 27.42 (18.62, 38.98) | -1.15  (-1.19, -1.12) | 0 |
| Ethiopia | 6150.93 (4166.06, 8853.39) | 13152.34 (9033.79, 18575.16) | 42.64 (28.95, 59.42) | 36.85 (25.31, 51.77) | -0.46  (-0.63, -0.29) | 0 |
| Fiji | 47.42 (30.63, 68.50) | 78.49 (50.87, 112.99) | 17.98 (11.87, 25.53) | 12.84 (8.36, 18.09) | -1.08  (-1.29, -0.88) | 0 |
| Finland | 663.40 (450.23, 938.05) | 906.71 (608.79, 1291.72) | 9.19 (6.23, 13.01) | 5.93 (3.99, 8.50) | -1.41  (-1.46, -1.36) | 0 |
| France | 6083.38 (4118.83, 8693.08) | 8170.98 (5588.50, 11413.98) | 6.81 (4.60, 9.68) | 4.70 (3.26, 6.51) | -1.19  (-1.25, -1.12) | 0 |
| Gabon | 121.74 (80.75, 174.17) | 129.95 (88.33, 183.07) | 25.12 (16.72, 35.78) | 16.49 (11.27, 23.04) | -1.36  (-1.4, -1.32) | 0 |
| Gambia | 42.42 (29.47, 58.15) | 138.90 (91.11, 199.16) | 16.25 (11.38, 22.11) | 17.76 (11.68, 25.34) | 0.31  (0.13, 0.48) | 0.001 |
| Georgia | 758.43 (511.79, 1084.08) | 680.69 (457.17, 960.00) | 12.85 (8.61, 18.47) | 10.44 (6.99, 14.72) | -0.67  (-0.7, -0.65) | 0 |
| Germany | 9279.52 (6313.13, 13131.54) | 11470.68 (7748.17, 16225.06) | 6.97 (4.79, 9.90) | 4.84 (3.26, 6.80) | -1.17  (-1.23, -1.11) | 0 |
| Ghana | 992.72 (668.17, 1386.45) | 2273.35 (1584.30, 3093.87) | 23.57 (15.84, 32.93) | 18.24 (12.64, 24.52) | -0.84  (-0.98, -0.7) | 0 |
| Greece | 1158.39 (784.06, 1672.34) | 1718.55 (1140.41, 2486.72) | 7.77 (5.26, 11.10) | 5.61 (3.77, 8.12) | -1.07  (-1.12, -1.01) | 0 |
| Greenland | 1.54 (1.03, 2.21) | 3.24 (2.18, 4.56) | 7.12 (4.87, 9.97) | 6.13 (4.16, 8.62) | -0.51  (-0.56, -0.45) | 0 |
| Grenada | 16.14 (10.40, 22.80) | 11.55 (7.69, 16.35) | 19.75 (12.86, 28.14) | 11.65 (7.88, 16.55) | -1.69  (-1.73, -1.65) | 0 |
| Guam | 5.83 (3.89, 8.46) | 17.95 (11.87, 25.32) | 10.26 (6.79, 14.62) | 8.09 (5.35, 11.44) | -0.79  (-0.92, -0.67) | 0 |
| Guatemala | 604.42 (398.75, 862.29) | 1307.59 (892.91, 1792.72) | 23.24 (15.33, 32.34) | 13.12 (9.06, 17.88) | -1.84  (-1.92, -1.76) | 0 |
| Guinea | 1039.40 (671.52, 1472.88) | 1581.39 (1049.92, 2242.96) | 37.18 (24.73, 52.49) | 34.44 (22.94, 48.30) | -0.25  (-0.28, -0.22) | 0 |
| Guinea-Bissau | 87.90 (57.74, 125.54) | 137.35 (90.74, 194.39) | 32.36 (21.29, 45.25) | 29.61 (19.89, 41.67) | -0.27  (-0.31, -0.23) | 0 |
| Guyana | 69.52 (45.96, 98.73) | 71.49 (48.11, 102.53) | 22.44 (15.01, 31.88) | 13.71 (9.21, 19.33) | -1.58  (-1.61, -1.55) | 0 |
| Haiti | 724.94 (465.98, 1051.14) | 1062.63 (703.77, 1549.53) | 30.20 (19.68, 43.01) | 20.34 (13.49, 29.10) | -1.26  (-1.31, -1.22) | 0 |
| Honduras | 345.72 (230.56, 495.06) | 666.14 (452.00, 929.80) | 20.50 (13.66, 29.24) | 12.75 (8.62, 17.65) | -1.53  (-1.57, -1.49) | 0 |
| Hungary | 1165.51 (779.84, 1694.21) | 1060.42 (726.37, 1474.25) | 8.12 (5.46, 11.80) | 4.91 (3.37, 6.88) | -1.59  (-1.65, -1.54) | 0 |
| Iceland | 23.95 (16.23, 34.04) | 41.40 (27.43, 60.00) | 7.83 (5.28, 11.14) | 6.23 (4.09, 9.00) | -0.73  (-0.78, -0.68) | 0 |
| India | 72489.49 (50474.87, 101623.25) | 127993.07 (89931.42, 176095.50) | 20.43 (14.17, 28.17) | 12.16 (8.54, 16.62) | -1.65  (-1.91, -1.4) | 0 |
| Indonesia | 8732.86 (5840.69, 12360.88) | 14292.02 (9814.38, 19795.19) | 11.92 (7.94, 16.82) | 7.73 (5.34, 10.66) | -1.39  (-1.43, -1.34) | 0 |
| Iran (Islamic Republic of) | 11275.46 (7883.60, 15798.23) | 24325.85 (17181.25, 33409.04) | 59.01 (40.85, 81.62) | 36.58 (25.60, 50.34) | -1.49  (-1.56, -1.43) | 0 |
| Iraq | 2861.38 (1876.71, 4039.43) | 4111.56 (2710.86, 5824.07) | 40.81 (26.68, 57.67) | 22.67 (15.09, 31.66) | -1.88  (-1.92, -1.85) | 0 |
| Ireland | 328.80 (223.46, 468.10) | 452.53 (303.97, 650.46) | 8.24 (5.65, 11.64) | 5.34 (3.59, 7.64) | -1.39  (-1.42, -1.35) | 0 |
| Israel | 406.33 (271.58, 587.08) | 840.36 (571.15, 1190.64) | 8.77 (5.90, 12.48) | 6.17 (4.20, 8.74) | -1.14  (-1.19, -1.08) | 0 |
| Italy | 20196.92 (13736.08, 28215.29) | 24763.80 (16983.30, 34479.73) | 22.15 (15.19, 30.99) | 13.91 (9.51, 19.30) | -1.52  (-1.59, -1.44) | 0 |
| Jamaica | 316.92 (208.12, 458.96) | 372.01 (253.34, 524.46) | 16.89 (11.18, 24.50) | 11.48 (7.80, 16.30) | -1.24  (-1.3, -1.18) | 0 |
| Japan | 12002.41 (8380.98, 16547.21) | 27818.88 (19315.81, 37804.60) | 7.38 (5.14, 10.18) | 5.89 (4.08, 8.08) | -0.72  (-0.74, -0.7) | 0 |
| Jordan | 340.75 (230.60, 488.84) | 1031.42 (702.51, 1434.65) | 36.34 (24.16, 52.11) | 18.95 (12.84, 26.36) | -2.07  (-2.13, -2.02) | 0 |
| Kazakhstan | 1578.61 (1058.51, 2310.27) | 1496.84 (1009.73, 2108.83) | 14.35 (9.61, 20.73) | 9.92 (6.83, 13.89) | -1.19  (-1.25, -1.13) | 0 |
| Kenya | 1999.77 (1393.61, 2801.12) | 4300.33 (2976.24, 5978.28) | 30.15 (20.83, 41.71) | 24.47 (16.91, 33.62) | -0.64  (-0.88, -0.4) | 0 |
| Kiribati | 4.29 (2.80, 6.06) | 6.70 (4.44, 9.74) | 15.41 (10.25, 21.90) | 12.66 (8.24, 17.91) | -0.66  (-0.77, -0.56) | 0 |
| Kuwait | 109.26 (74.26, 154.54) | 347.16 (242.63, 479.63) | 27.05 (17.91, 38.03) | 16.79 (11.50, 23.43) | -1.53  (-1.57, -1.48) | 0 |
| Kyrgyzstan | 481.13 (319.66, 701.42) | 538.82 (367.85, 755.82) | 17.82 (11.88, 25.86) | 13.80 (9.33, 19.40) | -0.82  (-0.88, -0.75) | 0 |
| Lao People's Democratic Republic | 123.43 (82.79, 176.23) | 187.50 (125.91, 260.08) | 8.34 (5.67, 11.70) | 5.44 (3.64, 7.67) | -1.37  (-1.43, -1.32) | 0 |
| Latvia | 246.84 (166.96, 353.33) | 227.69 (153.88, 324.39) | 6.90 (4.67, 9.88) | 4.92 (3.35, 6.90) | -1.08  (-1.15, -1.02) | 0 |
| Lebanon | 562.05 (378.11, 797.29) | 1138.57 (756.69, 1574.29) | 30.23 (20.71, 42.30) | 17.94 (11.90, 24.84) | -1.68  (-1.73, -1.62) | 0 |
| Lesotho | 300.84 (196.38, 438.64) | 263.14 (171.78, 382.97) | 41.46 (27.57, 59.14) | 31.31 (20.64, 43.99) | -0.9  (-0.93, -0.87) | 0 |
| Liberia | 376.16 (246.31, 535.27) | 409.03 (270.79, 568.53) | 39.46 (26.22, 55.48) | 27.78 (18.46, 38.56) | -1.13  (-1.17, -1.09) | 0 |
| Libya | 888.10 (588.24, 1247.62) | 1318.66 (897.35, 1847.26) | 55.07 (36.56, 77.14) | 32.37 (21.83, 45.14) | -1.59  (-1.67, -1.5) | 0 |
| Lithuania | 316.08 (212.66, 444.18) | 323.77 (222.06, 468.06) | 7.00 (4.72, 9.84) | 4.82 (3.32, 6.90) | -1.19  (-1.27, -1.12) | 0 |
| Luxembourg | 41.40 (27.62, 59.49) | 59.43 (40.22, 84.59) | 7.61 (5.12, 10.76) | 5.04 (3.40, 7.17) | -1.33  (-1.36, -1.29) | 0 |
| Madagascar | 1211.09 (852.77, 1681.27) | 1707.27 (1190.18, 2348.08) | 30.16 (20.94, 41.72) | 22.17 (15.33, 30.10) | -0.99  (-1.02, -0.96) | 0 |
| Malawi | 1389.53 (985.56, 1890.08) | 1649.75 (1112.24, 2298.60) | 47.57 (33.33, 64.07) | 29.18 (19.56, 40.52) | -1.59  (-1.65, -1.54) | 0 |
| Malaysia | 565.59 (382.36, 810.79) | 1359.74 (931.32, 1951.80) | 7.21 (4.82, 10.15) | 5.57 (3.77, 7.95) | -0.84  (-0.94, -0.74) | 0 |
| Maldives | 9.22 (5.96, 13.35) | 18.34 (12.68, 25.45) | 15.70 (10.21, 22.39) | 6.93 (4.75, 9.61) | -2.61  (-2.72, -2.5) | 0 |
| Mali | 1192.75 (823.17, 1638.30) | 2361.95 (1599.11, 3297.07) | 40.44 (28.10, 54.82) | 34.90 (24.12, 48.78) | -0.47  (-0.52, -0.41) | 0 |
| Malta | 34.98 (23.42, 50.40) | 65.27 (43.60, 92.26) | 8.85 (5.90, 12.73) | 5.78 (3.88, 8.21) | -1.37  (-1.41, -1.34) | 0 |
| Marshall Islands | 1.99 (1.28, 2.91) | 2.50 (1.65, 3.71) | 15.59 (10.27, 22.21) | 10.81 (7.17, 15.37) | -1.17  (-1.24, -1.09) | 0 |
| Mauritania | 286.38 (193.36, 401.81) | 470.87 (319.51, 668.08) | 34.11 (23.01, 47.33) | 26.13 (17.62, 36.94) | -0.85  (-0.89, -0.81) | 0 |
| Mauritius | 56.21 (37.70, 80.44) | 102.27 (69.77, 141.40) | 9.57 (6.45, 13.48) | 5.89 (4.03, 8.13) | -1.55  (-1.59, -1.51) | 0 |
| Mexico | 6195.40 (4285.37, 8450.39) | 13010.46 (9127.26, 17251.71) | 17.42 (12.11, 23.53) | 11.19 (7.85, 14.83) | -1.43  (-1.46, -1.4) | 0 |
| Micronesia (Federated States of) | 6.57 (4.35, 9.60) | 6.25 (4.03, 9.08) | 15.98 (10.74, 22.81) | 11.25 (7.23, 16.11) | -1.16  (-1.31, -1.01) | 0 |
| Monaco | 5.28 (3.56, 7.61) | 6.25 (4.15, 8.94) | 6.52 (4.36, 9.38) | 5.26 (3.51, 7.48) | -0.7  (-0.74, -0.67) | 0 |
| Mongolia | 233.90 (157.13, 341.58) | 270.11 (182.11, 387.38) | 26.07 (17.60, 37.41) | 15.70 (10.66, 22.37) | -1.63  (-1.71, -1.56) | 0 |
| Montenegro | 25.02 (16.71, 35.64) | 31.32 (21.31, 44.72) | 4.35 (2.88, 6.19) | 3.40 (2.32, 4.83) | -0.8  (-0.85, -0.76) | 0 |
| Morocco | 3373.41 (2401.08, 4588.29) | 5263.12 (3646.11, 7323.83) | 27.08 (19.20, 36.75) | 17.46 (12.12, 24.05) | -1.41  (-1.47, -1.36) | 0 |
| Mozambique | 1545.04 (1004.50, 2190.49) | 2009.29 (1382.33, 2784.22) | 33.96 (22.54, 47.49) | 24.80 (17.09, 33.84) | -1.03  (-1.08, -0.97) | 0 |
| Myanmar | 2930.35 (1839.31, 4255.03) | 4258.19 (2677.07, 6034.87) | 16.32 (10.45, 23.17) | 10.36 (6.59, 14.63) | -1.47  (-1.7, -1.23) | 0 |
| Namibia | 188.56 (122.96, 282.56) | 274.04 (183.33, 394.25) | 38.41 (25.34, 55.15) | 25.83 (17.21, 36.69) | -1.28  (-1.31, -1.25) | 0 |
| Nauru | 0.48 (0.32, 0.70) | 0.46 (0.30, 0.67) | 15.19 (10.04, 21.56) | 10.83 (7.10, 15.38) | -1.12  (-1.22, -1.03) | 0 |
| Nepal | 941.06 (667.13, 1285.74) | 1365.30 (941.58, 1893.22) | 13.83 (9.83, 18.73) | 6.93 (4.79, 9.58) | -2.2  (-2.35, -2.05) | 0 |
| Netherlands | 1299.52 (887.11, 1869.78) | 1880.42 (1254.94, 2656.04) | 6.32 (4.35, 9.04) | 4.78 (3.19, 6.77) | -0.91  (-0.94, -0.87) | 0 |
| New Zealand | 279.96 (195.17, 390.06) | 491.59 (344.28, 677.51) | 7.23 (5.06, 10.09) | 5.35 (3.72, 7.39) | -0.96  (-1, -0.93) | 0 |
| Nicaragua | 267.36 (181.03, 378.69) | 556.23 (373.70, 779.69) | 20.71 (13.96, 29.44) | 12.76 (8.53, 17.83) | -1.56  (-1.58, -1.53) | 0 |
| Niger | 811.08 (532.08, 1170.33) | 2601.41 (1720.93, 3695.81) | 41.53 (27.24, 58.47) | 41.83 (26.98, 58.47) | 0.02  (-0.02, 0.06) | 0.242 |
| Nigeria | 18040.26 (12822.32, 25142.00) | 25559.95 (17963.27, 35361.26) | 49.08 (34.63, 67.60) | 36.43 (25.36, 50.24) | -0.96  (-1.05, -0.88) | 0 |
| Niue | 0.29 (0.19, 0.41) | 0.17 (0.12, 0.25) | 12.19 (8.11, 17.63) | 8.30 (5.52, 11.66) | -1.26  (-1.33, -1.2) | 0 |
| North Macedonia | 92.90 (63.12, 134.53) | 107.46 (71.34, 156.43) | 5.73 (3.88, 8.25) | 3.75 (2.52, 5.37) | -1.36  (-1.4, -1.31) | 0 |
| Northern Mariana Islands | 1.07 (0.71, 1.56) | 3.30 (2.16, 4.81) | 10.41 (6.86, 14.80) | 8.08 (5.30, 11.56) | -0.85  (-0.9, -0.79) | 0 |
| Norway | 442.59 (308.54, 610.76) | 549.19 (383.40, 756.74) | 5.83 (4.06, 8.03) | 4.75 (3.33, 6.56) | -0.66  (-0.7, -0.62) | 0 |
| Oman | 186.86 (121.98, 268.41) | 312.19 (213.87, 439.62) | 35.65 (23.67, 50.66) | 21.90 (14.89, 30.68) | -1.55  (-1.62, -1.48) | 0 |
| Pakistan | 6206.02 (4341.97, 8511.12) | 12984.58 (9174.96, 18270.37) | 12.86 (8.95, 17.37) | 13.42 (9.40, 18.64) | 0.12  (0.07, 0.17) | 0 |
| Palau | 0.97 (0.64, 1.41) | 1.53 (1.03, 2.19) | 11.90 (7.87, 16.76) | 8.65 (5.75, 11.97) | -1.05  (-1.13, -0.96) | 0 |
| Palestine | 220.00 (144.07, 313.82) | 314.43 (211.57, 443.23) | 29.44 (19.27, 41.61) | 16.46 (10.96, 22.98) | -1.88  (-1.98, -1.79) | 0 |
| Panama | 228.56 (148.79, 328.00) | 484.66 (325.41, 671.52) | 16.84 (11.06, 24.13) | 10.80 (7.25, 15.07) | -1.43  (-1.48, -1.38) | 0 |
| Papua New Guinea | 163.74 (107.28, 237.26) | 377.76 (247.98, 535.85) | 12.80 (8.40, 17.98) | 10.38 (6.92, 14.70) | -0.67  (-0.76, -0.58) | 0 |
| Paraguay | 399.89 (276.87, 545.76) | 742.29 (516.53, 1011.41) | 20.00 (13.85, 27.31) | 13.87 (9.60, 18.98) | -1.18  (-1.22, -1.14) | 0 |
| Peru | 2738.46 (1825.19, 3919.60) | 5087.90 (3458.27, 7105.10) | 25.89 (17.26, 36.92) | 15.53 (10.50, 21.77) | -1.63  (-1.71, -1.56) | 0 |
| Philippines | 2616.69 (1784.83, 3627.64) | 6027.62 (4132.56, 8265.05) | 11.49 (7.93, 15.89) | 9.08 (6.26, 12.55) | -0.75  (-0.78, -0.73) | 0 |
| Poland | 1816.69 (1271.90, 2489.22) | 2591.52 (1794.74, 3474.61) | 4.39 (3.07, 5.97) | 3.35 (2.31, 4.51) | -0.87  (-0.91, -0.83) | 0 |
| Portugal | 1362.43 (916.54, 1973.52) | 1853.15 (1253.27, 2646.16) | 10.29 (6.88, 14.57) | 6.17 (4.20, 8.73) | -1.66  (-1.74, -1.57) | 0 |
| Puerto Rico | 489.32 (322.79, 696.54) | 725.66 (490.14, 1014.05) | 13.64 (9.06, 19.44) | 8.32 (5.69, 11.60) | -1.58  (-1.62, -1.54) | 0 |
| Qatar | 28.77 (19.14, 41.58) | 119.13 (79.52, 172.21) | 48.59 (32.80, 68.23) | 24.28 (16.39, 34.06) | -2.31  (-2.6, -2.02) | 0 |
| Republic of Korea | 2807.32 (1914.91, 4020.43) | 6025.76 (4106.05, 8470.90) | 12.51 (8.43, 17.37) | 6.37 (4.35, 8.91) | -2.17  (-2.23, -2.12) | 0 |
| Republic of Moldova | 313.68 (206.25, 445.95) | 352.72 (247.21, 472.94) | 8.34 (5.60, 11.78) | 5.75 (4.03, 7.73) | -1.19  (-1.26, -1.12) | 0 |
| Romania | 1420.21 (934.40, 2029.27) | 1593.28 (1072.16, 2312.94) | 5.77 (3.89, 8.27) | 3.88 (2.63, 5.63) | -1.27  (-1.34, -1.2) | 0 |
| Russian Federation | 18496.52 (12726.95, 25489.45) | 18339.09 (12783.69, 24754.96) | 11.18 (7.76, 15.41) | 7.48 (5.18, 10.13) | -1.29  (-1.32, -1.27) | 0 |
| Rwanda | 589.95 (407.55, 822.46) | 870.10 (616.87, 1205.16) | 28.14 (19.29, 38.71) | 18.09 (12.64, 24.70) | -1.41  (-1.45, -1.37) | 0 |
| Saint Kitts and Nevis | 7.22 (4.65, 10.46) | 6.09 (4.07, 8.73) | 18.41 (11.96, 26.42) | 10.99 (7.33, 15.57) | -1.65  (-1.7, -1.6) | 0 |
| Saint Lucia | 15.20 (9.96, 21.93) | 26.36 (17.55, 37.42) | 18.43 (12.22, 26.24) | 11.27 (7.48, 15.99) | -1.57  (-1.61, -1.52) | 0 |
| Saint Vincent and the Grenadines | 13.30 (8.76, 18.97) | 17.31 (11.60, 24.55) | 19.19 (12.73, 27.31) | 12.66 (8.42, 17.81) | -1.33  (-1.36, -1.31) | 0 |
| Samoa | 9.65 (6.41, 14.59) | 12.41 (8.27, 17.87) | 13.48 (8.96, 19.93) | 10.02 (6.61, 14.26) | -0.98  (-1.05, -0.91) | 0 |
| San Marino | 2.48 (1.66, 3.52) | 5.21 (3.57, 7.38) | 6.61 (4.43, 9.41) | 5.36 (3.65, 7.63) | -0.68  (-0.72, -0.64) | 0 |
| Sao Tome and Principe | 21.60 (14.18, 30.69) | 22.35 (15.14, 31.21) | 37.10 (24.37, 52.34) | 25.89 (17.33, 35.82) | -1.16  (-1.19, -1.12) | 0 |
| Saudi Arabia | 2078.56 (1471.62, 2864.56) | 3336.75 (2255.87, 4778.88) | 46.21 (32.59, 62.93) | 28.58 (19.07, 40.53) | -1.55  (-1.62, -1.48) | 0 |
| Senegal | 1008.43 (686.41, 1411.91) | 1806.91 (1216.64, 2482.30) | 38.98 (26.17, 54.00) | 28.89 (19.48, 39.41) | -0.92  (-0.95, -0.89) | 0 |
| Serbia | 494.14 (334.05, 730.90) | 624.33 (420.86, 868.80) | 5.30 (3.56, 7.71) | 3.53 (2.39, 4.91) | -1.28  (-1.32, -1.24) | 0 |
| Seychelles | 5.66 (3.77, 8.09) | 6.27 (4.27, 8.66) | 10.01 (6.67, 14.37) | 6.20 (4.17, 8.53) | -1.53  (-1.59, -1.47) | 0 |
| Sierra Leone | 666.56 (426.43, 956.30) | 870.05 (570.90, 1240.71) | 38.90 (24.90, 56.21) | 30.35 (20.45, 42.98) | -0.82  (-1.01, -0.62) | 0 |
| Singapore | 198.71 (134.24, 283.50) | 655.84 (442.81, 912.44) | 11.22 (7.57, 15.79) | 7.85 (5.27, 10.91) | -1.14  (-1.2, -1.08) | 0 |
| Slovakia | 281.60 (192.16, 404.93) | 339.35 (232.81, 484.21) | 4.90 (3.32, 6.97) | 3.48 (2.39, 4.97) | -1.1  (-1.15, -1.06) | 0 |
| Slovenia | 99.36 (67.22, 144.55) | 150.47 (101.40, 212.19) | 4.12 (2.78, 6.00) | 2.94 (1.96, 4.15) | -1.07  (-1.14, -1) | 0 |
| Solomon Islands | 18.43 (11.94, 27.04) | 35.38 (23.32, 49.74) | 18.68 (12.19, 26.60) | 13.85 (9.19, 19.39) | -0.98  (-1.07, -0.9) | 0 |
| Somalia | 465.79 (309.27, 671.98) | 1176.01 (794.22, 1727.76) | 30.87 (20.70, 43.63) | 29.35 (19.71, 42.08) | -0.16  (-0.2, -0.13) | 0 |
| South Africa | 5305.29 (3686.14, 7483.88) | 9275.21 (6388.06, 12952.80) | 29.78 (20.57, 41.78) | 23.63 (16.33, 32.70) | -0.73  (-0.94, -0.51) | 0 |
| South Sudan | 532.28 (341.86, 776.10) | 478.91 (307.82, 694.15) | 25.83 (16.76, 36.69) | 18.77 (12.01, 26.92) | -0.97  (-1.06, -0.89) | 0 |
| Spain | 10734.44 (7224.55, 15535.20) | 15101.70 (10425.38, 21295.99) | 19.45 (13.23, 27.87) | 12.69 (8.68, 17.66) | -1.37  (-1.42, -1.32) | 0 |
| Sri Lanka | 745.65 (513.56, 1067.17) | 1303.24 (904.61, 1791.40) | 8.65 (5.96, 12.45) | 5.12 (3.57, 7.06) | -1.67  (-1.74, -1.6) | 0 |
| Sudan | 4053.53 (2683.58, 5775.29) | 4223.48 (2845.68, 5831.17) | 53.05 (35.55, 74.54) | 27.98 (18.52, 38.50) | -1.95  (-2.03, -1.87) | 0 |
| Suriname | 53.31 (36.28, 75.03) | 94.72 (64.79, 129.73) | 23.73 (15.74, 33.20) | 16.05 (10.93, 21.91) | -1.26  (-1.3, -1.22) | 0 |
| Sweden | 502.11 (346.68, 714.60) | 723.67 (491.78, 1022.98) | 2.97 (2.05, 4.24) | 2.70 (1.84, 3.84) | -0.25  (-0.31, -0.19) | 0 |
| Switzerland | 692.79 (468.26, 979.13) | 1046.31 (712.13, 1496.88) | 6.14 (4.16, 8.67) | 4.77 (3.24, 6.82) | -0.81  (-0.85, -0.78) | 0 |
| Syrian Arab Republic | 1916.31 (1280.72, 2731.97) | 2597.99 (1766.89, 3681.23) | 45.08 (30.13, 63.24) | 24.19 (16.45, 33.80) | -2  (-2.04, -1.95) | 0 |
| Taiwan (Province of China) | 126.49 (77.68, 197.26) | 262.42 (158.62, 404.93) | 1.02 (0.64, 1.56) | 0.59 (0.36, 0.91) | -1.79  (-1.9, -1.67) | 0 |
| Tajikistan | 457.05 (302.77, 662.44) | 652.16 (438.57, 944.78) | 18.85 (12.50, 27.26) | 15.14 (10.17, 21.29) | -0.7  (-0.75, -0.65) | 0 |
| Thailand | 2320.11 (1598.04, 3161.95) | 4771.34 (3294.59, 6793.25) | 8.39 (5.80, 11.38) | 4.34 (2.99, 6.16) | -2.09  (-2.18, -2.01) | 0 |
| Timor-Leste | 53.05 (35.10, 75.76) | 108.15 (72.60, 152.14) | 29.65 (19.58, 42.08) | 14.98 (10.08, 21.24) | -2.12  (-2.19, -2.05) | 0 |
| Togo | 242.59 (162.76, 335.09) | 749.42 (501.30, 1058.90) | 27.51 (18.44, 37.98) | 27.64 (18.39, 38.85) | 0.01  (-0.02, 0.05) | 0.451 |
| Tokelau | 0.19 (0.13, 0.28) | 0.15 (0.10, 0.21) | 14.98 (9.76, 21.69) | 9.75 (6.44, 13.97) | -1.4  (-1.46, -1.34) | 0 |
| Tonga | 6.30 (4.32, 9.01) | 6.54 (4.29, 9.15) | 13.40 (9.21, 18.87) | 8.65 (5.66, 12.12) | -1.42  (-1.62, -1.22) | 0 |
| Trinidad and Tobago | 123.89 (81.08, 178.32) | 182.81 (125.45, 256.77) | 16.30 (10.75, 23.24) | 9.71 (6.69, 13.58) | -1.66  (-1.7, -1.62) | 0 |
| Tunisia | 1903.86 (1272.91, 2745.31) | 2598.22 (1731.69, 3655.02) | 44.70 (30.29, 63.76) | 21.19 (14.14, 29.75) | -2.39  (-2.41, -2.37) | 0 |
| Turkey | 7408.45 (4979.84, 10562.75) | 11957.94 (8102.53, 17270.45) | 25.66 (17.19, 36.33) | 13.66 (9.19, 19.58) | -2.03  (-2.07, -1.99) | 0 |
| Turkmenistan | 179.30 (121.28, 250.07) | 277.95 (187.78, 401.38) | 11.45 (7.77, 15.82) | 8.44 (5.73, 12.15) | -0.98  (-1.03, -0.93) | 0 |
| Tuvalu | 0.87 (0.58, 1.26) | 1.00 (0.67, 1.44) | 16.17 (10.56, 22.64) | 11.04 (7.33, 15.63) | -1.24  (-1.35, -1.13) | 0 |
| Uganda | 2071.68 (1422.28, 2880.92) | 2193.30 (1543.10, 3017.79) | 41.46 (28.48, 56.71) | 19.91 (13.95, 27.30) | -2.21  (-2.49, -1.93) | 0 |
| Ukraine | 4925.64 (3402.94, 6919.59) | 4804.46 (3262.33, 6541.47) | 7.24 (5.00, 10.03) | 5.89 (4.00, 8.05) | -0.65  (-0.74, -0.56) | 0 |
| United Arab Emirates | 85.29 (57.51, 122.58) | 390.38 (260.47, 589.69) | 34.84 (23.34, 49.25) | 21.03 (14.51, 29.91) | -1.61  (-1.63, -1.58) | 0 |
| United Kingdom | 6947.35 (4788.81, 9690.78) | 8325.52 (5772.32, 11517.04) | 7.25 (5.02, 10.08) | 5.52 (3.86, 7.62) | -0.89  (-0.93, -0.85) | 0 |
| United Republic of Tanzania | 3817.58 (2586.53, 5325.60) | 6279.41 (4313.22, 8651.90) | 45.01 (30.50, 62.59) | 31.06 (21.30, 43.22) | -1.2  (-1.39, -1) | 0 |
| United States of America | 17269.58 (11978.37, 23578.80) | 29604.40 (20765.27, 40571.23) | 5.09 (3.53, 6.95) | 4.59 (3.20, 6.29) | -0.33  (-0.4, -0.25) | 0 |
| United States Virgin Islands | 9.97 (6.67, 14.19) | 19.34 (12.93, 27.29) | 14.60 (9.66, 20.68) | 9.78 (6.51, 13.66) | -1.28  (-1.31, -1.25) | 0 |
| Uruguay | 284.41 (192.61, 398.58) | 360.54 (240.35, 495.64) | 7.22 (4.93, 10.06) | 5.57 (3.77, 7.70) | -0.83  (-0.86, -0.81) | 0 |
| Uzbekistan | 1812.00 (1222.27, 2563.33) | 2549.66 (1728.97, 3650.52) | 17.08 (11.39, 24.19) | 12.37 (8.32, 17.75) | -1.04  (-1.08, -1) | 0 |
| Vanuatu | 5.37 (3.67, 7.72) | 10.93 (7.19, 15.66) | 11.84 (8.25, 16.70) | 8.32 (5.51, 11.78) | -1.16  (-1.28, -1.04) | 0 |
| Venezuela (Bolivarian Republic of) | 1275.26 (876.58, 1730.58) | 3178.21 (2176.89, 4425.71) | 15.49 (10.58, 21.03) | 11.32 (7.81, 15.68) | -1  (-1.08, -0.91) | 0 |
| Viet Nam | 4055.44 (2799.63, 5649.22) | 5527.34 (3896.08, 7654.72) | 11.53 (7.93, 15.96) | 6.56 (4.62, 9.03) | -1.83  (-1.92, -1.74) | 0 |
| Yemen | 951.78 (628.46, 1364.49) | 2317.92 (1563.01, 3264.02) | 26.39 (17.67, 37.43) | 21.84 (14.78, 30.88) | -0.63  (-0.71, -0.55) | 0 |
| Zambia | 776.35 (532.73, 1084.00) | 1247.36 (837.17, 1759.86) | 35.61 (24.08, 49.60) | 24.45 (16.46, 34.12) | -1.15  (-1.25, -1.04) | 0 |
| Zimbabwe | 768.72 (527.79, 1085.86) | 996.61 (675.09, 1370.40) | 23.91 (16.47, 33.79) | 20.17 (13.71, 27.63) | -0.55  (-0.58, -0.52) | 0 |
| Geographic Standardization Note: All geographic and administrative divisions in this study comply with the one-China principle and the coding standards of the Global Burden of Disease (GBD) Study and World Health Organization (WHO). Taiwan (Province of China) is included as a provincial-level administrative region of the People's Republic of China. Displayed location-specific estimates may not sum exactly to aggregate estimates because of GBD estimation and rounding. | | | | | | |

## Supplementary Table 4

| Supplementary Table 4 National-level trends in age-standardized glaucoma prevalence, 1990–2021: annual percentage changes (APC) from joinpoint regression analysis | | | | | | | |
| --- | --- | --- | --- | --- | --- | --- | --- |
| measure | location | sex | cause | Segment.Start | Segment.End | P.Value | APC_95CI |
| Prevalence | Afghanistan | Both | Glaucoma | 1990 | 2002 | 0.030793841 | 0.36  (0.17, 0.59) |
| Prevalence | Afghanistan | Both | Glaucoma | 2002 | 2008 | 0.136372725 | -0.63  (-1.04, 0.5) |
| Prevalence | Afghanistan | Both | Glaucoma | 2008 | 2019 | 0.00239952 | -1.56  (-1.7, -0.58) |
| Prevalence | China | Both | Glaucoma | 2019 | 2021 | 0 | -13.09  (-15.95, -8.47) |
| Prevalence | Afghanistan | Female | Glaucoma | 1990 | 1995 | 0.600679864 | 0.17  (-1.11, 0.78) |
| Prevalence | Afghanistan | Female | Glaucoma | 1995 | 2000 | 0.109978004 | 1.3  (-0.35, 2.27) |
| Prevalence | Afghanistan | Female | Glaucoma | 2000 | 2008 | 0.089182164 | -0.26  (-1.69, 0.09) |
| Prevalence | China | Male | Glaucoma | 2019 | 2021 | 0 | -13.19  (-16.03, -8.51) |
| Prevalence | Afghanistan | Male | Glaucoma | 1990 | 2002 | 0.00239952 | 0.12  (0.06, 0.22) |
| Prevalence | Afghanistan | Male | Glaucoma | 2002 | 2007 | 0.00519896 | -0.7  (-1.07, -0.27) |
| Prevalence | Afghanistan | Male | Glaucoma | 2007 | 2019 | 0 | -1.44  (-1.52, -1.36) |
| Prevalence | China | Female | Glaucoma | 2019 | 2021 | 0 | -13.18  (-16.09, -8.44) |
| Prevalence | Albania | Both | Glaucoma | 1990 | 1993 | 0 | -0.35  (-0.41, -0.26) |
| Prevalence | Albania | Both | Glaucoma | 1993 | 1996 | 0 | -0.74  (-0.8, -0.67) |
| Prevalence | Albania | Both | Glaucoma | 1996 | 2005 | 0 | -1.44  (-1.46, -1.43) |
| Prevalence | Albania | Both | Glaucoma | 2005 | 2009 | 0 | -1.07  (-1.12, -1) |
| Prevalence | Albania | Both | Glaucoma | 2009 | 2018 | 0 | -0.66  (-0.68, -0.64) |
| Prevalence | Qatar | Both | Glaucoma | 2019 | 2021 | 0 | -8.54  (-11.03, -5.82) |
| Prevalence | Albania | Female | Glaucoma | 1990 | 1995 | 0 | -0.65  (-0.72, -0.58) |
| Prevalence | Albania | Female | Glaucoma | 1995 | 2000 | 0 | -1.08  (-1.16, -1) |
| Prevalence | Albania | Female | Glaucoma | 2000 | 2005 | 0 | -1.52  (-1.64, -1.45) |
| Prevalence | Albania | Female | Glaucoma | 2005 | 2009 | 0 | -1.18  (-1.33, -1) |
| Prevalence | Albania | Female | Glaucoma | 2009 | 2019 | 0 | -0.77  (-0.79, -0.74) |
| Prevalence | Qatar | Female | Glaucoma | 2019 | 2021 | 0 | -12.92  (-16.81, -8.5) |
| Prevalence | Albania | Male | Glaucoma | 1990 | 1993 | 0.145170966 | -0.09  (-0.19, 0.06) |
| Prevalence | Albania | Male | Glaucoma | 1993 | 1996 | 0 | -0.72  (-0.81, -0.62) |
| Prevalence | Albania | Male | Glaucoma | 1996 | 2004 | 0 | -1.72  (-1.76, -1.69) |
| Prevalence | Albania | Male | Glaucoma | 2004 | 2009 | 0 | -1.15  (-1.25, -1.08) |
| Prevalence | Albania | Male | Glaucoma | 2009 | 2015 | 0 | -0.76  (-0.84, -0.69) |
| Prevalence | Uganda | Female | Glaucoma | 2019 | 2021 | 0 | -7.08  (-8.56, -5.04) |
| Prevalence | Algeria | Both | Glaucoma | 1990 | 1994 | 0 | -1.47  (-1.53, -1.44) |
| Prevalence | Algeria | Both | Glaucoma | 1994 | 2005 | 0 | -1.34  (-1.36, -1.32) |
| Prevalence | Algeria | Both | Glaucoma | 2005 | 2008 | 0 | -1.16  (-1.37, -1.12) |
| Prevalence | Algeria | Both | Glaucoma | 2008 | 2015 | 0 | -1.09  (-1.11, -1.05) |
| Prevalence | Algeria | Both | Glaucoma | 2015 | 2018 | 0 | -1.36  (-1.39, -1.3) |
| Prevalence | Uganda | Both | Glaucoma | 2019 | 2021 | 0 | -6.45  (-8.16, -4.3) |
| Prevalence | Algeria | Female | Glaucoma | 1990 | 1996 | 0 | -1.11  (-1.12, -1.09) |
| Prevalence | Algeria | Female | Glaucoma | 1996 | 2001 | 0 | -1.18  (-1.2, -1.15) |
| Prevalence | Algeria | Female | Glaucoma | 2001 | 2005 | 0 | -1.24  (-1.27, -1.21) |
| Prevalence | Algeria | Female | Glaucoma | 2005 | 2018 | 0 | -1.15  (-1.15, -1.14) |
| Prevalence | Qatar | Male | Glaucoma | 2019 | 2021 | 0 | -6.05  (-7.85, -4.34) |
| Prevalence | Algeria | Male | Glaucoma | 1990 | 1994 | 0 | -1.48  (-1.56, -1.43) |
| Prevalence | Algeria | Male | Glaucoma | 1994 | 2000 | 0 | -1.29  (-1.32, -1.23) |
| Prevalence | Algeria | Male | Glaucoma | 2000 | 2006 | 0 | -1.49  (-1.53, -1.46) |
| Prevalence | Algeria | Male | Glaucoma | 2006 | 2015 | 0 | -1.18  (-1.19, -1.16) |
| Prevalence | Algeria | Male | Glaucoma | 2015 | 2018 | 0 | -1.49  (-1.54, -1.41) |
| Prevalence | Sudan | Female | Glaucoma | 2019 | 2021 | 0 | -6.03  (-7.37, -3.79) |
| Prevalence | American Samoa | Both | Glaucoma | 1990 | 2000 | 0 | -1.06  (-1.18, -0.98) |
| Prevalence | American Samoa | Both | Glaucoma | 2000 | 2010 | 0.00479904 | -0.57  (-0.64, -0.37) |
| Prevalence | American Samoa | Both | Glaucoma | 2010 | 2015 | 0 | -1.1  (-1.53, -0.89) |
| Prevalence | Sudan | Both | Glaucoma | 2019 | 2021 | 0 | -5.89  (-7.16, -3.76) |
| Prevalence | American Samoa | Female | Glaucoma | 1990 | 1998 | 0 | -0.92  (-1.64, -0.7) |
| Prevalence | American Samoa | Female | Glaucoma | 1998 | 2010 | 0.091181764 | -0.37  (-0.48, 0.21) |
| Prevalence | American Samoa | Female | Glaucoma | 2010 | 2015 | 0.00119976 | -1.3  (-2.19, -0.88) |
| Prevalence | Uganda | Male | Glaucoma | 2019 | 2021 | 0 | -5.67  (-6.97, -3.86) |
| Prevalence | American Samoa | Male | Glaucoma | 1990 | 1994 | 0 | -1.08  (-1.12, -1.04) |
| Prevalence | American Samoa | Male | Glaucoma | 1994 | 2000 | 0 | -1.33  (-1.35, -1.31) |
| Prevalence | American Samoa | Male | Glaucoma | 2000 | 2006 | 0 | -0.74  (-0.76, -0.71) |
| Prevalence | American Samoa | Male | Glaucoma | 2006 | 2015 | 0 | -0.92  (-0.94, -0.91) |
| Prevalence | American Samoa | Male | Glaucoma | 2015 | 2018 | 0 | -1.1  (-1.14, -1.04) |
| Prevalence | Sudan | Male | Glaucoma | 2019 | 2021 | 0 | -5.59  (-6.86, -3.32) |
| Prevalence | Andorra | Both | Glaucoma | 1990 | 2000 | 0 | -0.9  (-0.95, -0.87) |
| Prevalence | Andorra | Both | Glaucoma | 2000 | 2006 | 0 | -0.72  (-0.81, -0.61) |
| Prevalence | Nepal | Both | Glaucoma | 2019 | 2021 | 0 | -4.31  (-4.98, -3.51) |
| Prevalence | Andorra | Female | Glaucoma | 1990 | 1995 | 0 | -0.83  (-0.9, -0.71) |
| Prevalence | Andorra | Female | Glaucoma | 1995 | 2000 | 0 | -0.95  (-1.08, -0.65) |
| Prevalence | Andorra | Female | Glaucoma | 2000 | 2006 | 0 | -0.64  (-0.71, -0.44) |
| Prevalence | Timor-Leste | Male | Glaucoma | 2019 | 2021 | 0 | -5.45  (-6.67, -3.73) |
| Prevalence | Andorra | Male | Glaucoma | 1990 | 2005 | 0 | -0.87  (-0.9, -0.85) |
| Prevalence | Fiji | Male | Glaucoma | 2018 | 2021 | 0 | -4.39  (-5.28, -3.34) |
| Prevalence | Angola | Both | Glaucoma | 1990 | 1996 | 0 | -0.53  (-0.55, -0.5) |
| Prevalence | Angola | Both | Glaucoma | 1996 | 2003 | 0 | -0.7  (-0.71, -0.67) |
| Prevalence | Angola | Both | Glaucoma | 2003 | 2006 | 0 | -0.85  (-0.91, -0.8) |
| Prevalence | Angola | Both | Glaucoma | 2006 | 2010 | 0 | -1.19  (-1.23, -1.16) |
| Prevalence | Angola | Both | Glaucoma | 2010 | 2015 | 0 | -1.45  (-1.47, -1.43) |
| Prevalence | Fiji | Both | Glaucoma | 2019 | 2021 | 0 | -4.17  (-6.07, -2.24) |
| Prevalence | Angola | Female | Glaucoma | 1990 | 2007 | 0 | -0.62  (-0.65, -0.6) |
| Prevalence | Angola | Female | Glaucoma | 2007 | 2015 | 0 | -1.44  (-1.55, -1.36) |
| Prevalence | Nepal | Female | Glaucoma | 2019 | 2021 | 0 | -4.37  (-5.37, -3.31) |
| Prevalence | Angola | Male | Glaucoma | 1990 | 2000 | 0.00159968 | -0.41  (-0.47, -0.36) |
| Prevalence | Angola | Male | Glaucoma | 2000 | 2009 | 0.00039992 | -0.96  (-1.03, -0.87) |
| Prevalence | Angola | Male | Glaucoma | 2009 | 2019 | 0 | -1.24  (-1.43, -1.01) |
| Prevalence | Nepal | Male | Glaucoma | 2019 | 2021 | 0 | -4.09  (-4.59, -3.46) |
| Prevalence | Antigua and Barbuda | Both | Glaucoma | 1990 | 1995 | 0 | -1.01  (-1.03, -0.98) |
| Prevalence | Antigua and Barbuda | Both | Glaucoma | 1995 | 2000 | 0 | -1.36  (-1.38, -1.33) |
| Prevalence | Antigua and Barbuda | Both | Glaucoma | 2000 | 2005 | 0 | -0.88  (-0.91, -0.85) |
| Prevalence | Antigua and Barbuda | Both | Glaucoma | 2005 | 2010 | 0 | -1.06  (-1.1, -1.03) |
| Prevalence | Antigua and Barbuda | Both | Glaucoma | 2010 | 2015 | 0 | -0.54  (-0.57, -0.5) |
| Prevalence | Timor-Leste | Both | Glaucoma | 2019 | 2021 | 0 | -4.04  (-5.16, -2.08) |
| Prevalence | Antigua and Barbuda | Female | Glaucoma | 1990 | 1995 | 0 | -0.96  (-1.05, -0.81) |
| Prevalence | Antigua and Barbuda | Female | Glaucoma | 1995 | 2000 | 0 | -1.43  (-1.65, -1.32) |
| Prevalence | Antigua and Barbuda | Female | Glaucoma | 2000 | 2005 | 0 | -0.87  (-0.98, -0.65) |
| Prevalence | Antigua and Barbuda | Female | Glaucoma | 2005 | 2010 | 0 | -1.21  (-1.41, -1.11) |
| Prevalence | Antigua and Barbuda | Female | Glaucoma | 2010 | 2014 | 0 | -0.42  (-0.56, -0.23) |
| Prevalence | Timor-Leste | Female | Glaucoma | 2019 | 2021 | 0 | -3.56  (-4.41, -2.13) |
| Prevalence | Antigua and Barbuda | Male | Glaucoma | 1990 | 1995 | 0 | -1.09  (-1.12, -1.06) |
| Prevalence | Antigua and Barbuda | Male | Glaucoma | 1995 | 2000 | 0 | -1.4  (-1.44, -1.36) |
| Prevalence | Antigua and Barbuda | Male | Glaucoma | 2000 | 2005 | 0 | -1.04  (-1.12, -1.01) |
| Prevalence | Antigua and Barbuda | Male | Glaucoma | 2005 | 2009 | 0 | -0.89  (-0.96, -0.76) |
| Prevalence | Antigua and Barbuda | Male | Glaucoma | 2009 | 2019 | 0 | -0.67  (-0.68, -0.65) |
| Prevalence | Afghanistan | Male | Glaucoma | 2019 | 2021 | 0 | -3.03  (-3.51, -2.28) |
| Prevalence | Argentina | Both | Glaucoma | 1990 | 1993 | 0 | -1.53  (-1.73, -1.43) |
| Prevalence | Argentina | Both | Glaucoma | 1993 | 1996 | 0 | -1.05  (-1.29, -0.81) |
| Prevalence | Argentina | Both | Glaucoma | 1996 | 2004 | 0 | -0.68  (-0.72, -0.63) |
| Prevalence | Argentina | Both | Glaucoma | 2004 | 2012 | 0 | -0.54  (-0.58, -0.49) |
| Prevalence | Argentina | Both | Glaucoma | 2012 | 2019 | 0 | -0.95  (-0.98, -0.91) |
| Prevalence | Libya | Both | Glaucoma | 2019 | 2021 | 0.00039992 | -3.13  (-4.76, -0.61) |
| Prevalence | Argentina | Female | Glaucoma | 1990 | 1994 | 0 | -1.25  (-1.38, -1.19) |
| Prevalence | Argentina | Female | Glaucoma | 1994 | 2005 | 0 | -0.74  (-0.78, -0.72) |
| Prevalence | Argentina | Female | Glaucoma | 2005 | 2011 | 0 | -0.63  (-0.68, -0.54) |
| Prevalence | Argentina | Female | Glaucoma | 2011 | 2019 | 0 | -1.01  (-1.04, -0.98) |
| Prevalence | Libya | Male | Glaucoma | 2019 | 2021 | 0.00039992 | -2.58  (-3.97, -0.5) |
| Prevalence | Argentina | Male | Glaucoma | 1990 | 1993 | 0 | -1.8  (-2.04, -1.65) |
| Prevalence | Argentina | Male | Glaucoma | 1993 | 1996 | 0 | -1.2  (-1.48, -0.86) |
| Prevalence | Argentina | Male | Glaucoma | 1996 | 2003 | 0 | -0.64  (-0.71, -0.56) |
| Prevalence | Argentina | Male | Glaucoma | 2003 | 2013 | 0 | -0.45  (-0.48, -0.39) |
| Prevalence | Argentina | Male | Glaucoma | 2013 | 2019 | 0 | -0.89  (-1.02, -0.65) |
| Prevalence | Guyana | Male | Glaucoma | 2019 | 2021 | 0 | -2.08  (-2.21, -1.88) |
| Prevalence | Armenia | Both | Glaucoma | 1990 | 1996 | 0 | -0.66  (-0.89, -0.44) |
| Prevalence | Armenia | Both | Glaucoma | 1996 | 2001 | 0 | -0.92  (-1.08, -0.48) |
| Prevalence | Armenia | Both | Glaucoma | 2001 | 2010 | 0 | -1.25  (-1.42, -0.88) |
| Prevalence | Armenia | Both | Glaucoma | 2010 | 2014 | 0 | -1  (-1.41, -0.85) |
| Prevalence | Armenia | Both | Glaucoma | 2014 | 2019 | 0 | -0.75  (-1.03, -0.63) |
| Prevalence | Afghanistan | Both | Glaucoma | 2019 | 2021 | 0 | -2.81  (-3.56, -1.63) |
| Prevalence | Armenia | Female | Glaucoma | 1990 | 1998 | 0 | -0.64  (-0.71, -0.54) |
| Prevalence | Armenia | Female | Glaucoma | 1998 | 2013 | 0 | -1.07  (-1.12, -1.04) |
| Prevalence | Senegal | Male | Glaucoma | 2019 | 2021 | 0 | -1.99  (-2.32, -1.47) |
| Prevalence | Armenia | Male | Glaucoma | 1990 | 1996 | 0 | -0.6  (-0.66, -0.55) |
| Prevalence | Armenia | Male | Glaucoma | 1996 | 2002 | 0 | -1.06  (-1.12, -0.98) |
| Prevalence | Armenia | Male | Glaucoma | 2002 | 2010 | 0 | -1.54  (-1.6, -1.21) |
| Prevalence | Armenia | Male | Glaucoma | 2010 | 2014 | 0 | -1.2  (-1.52, -1.09) |
| Prevalence | Armenia | Male | Glaucoma | 2014 | 2019 | 0 | -0.99  (-1.05, -0.89) |
| Prevalence | Zambia | Male | Glaucoma | 2012 | 2021 | 0 | -1.75  (-1.86, -1.68) |
| Prevalence | Australia | Both | Glaucoma | 1990 | 1998 | 0 | -1.01  (-1.07, -0.97) |
| Prevalence | Australia | Both | Glaucoma | 1998 | 2005 | 0 | -0.5  (-0.91, -0.45) |
| Prevalence | Australia | Both | Glaucoma | 2005 | 2010 | 0.097580484 | 0.58  (-0.49, 0.63) |
| Prevalence | Australia | Both | Glaucoma | 2010 | 2019 | 0.102379524 | -0.48  (-0.55, 0.55) |
| Prevalence | Brazil | Both | Glaucoma | 2015 | 2021 | 0 | -1.95  (-2.33, -1.54) |
| Prevalence | Australia | Female | Glaucoma | 1990 | 1998 | 0 | -1.01  (-1.05, -0.98) |
| Prevalence | Australia | Female | Glaucoma | 1998 | 2005 | 0 | -0.39  (-0.43, -0.35) |
| Prevalence | Australia | Female | Glaucoma | 2005 | 2010 | 0.01079784 | 0.39  (0.32, 0.47) |
| Prevalence | Australia | Female | Glaucoma | 2010 | 2015 | 0.013997201 | -0.24  (-0.3, -0.11) |
| Prevalence | Australia | Female | Glaucoma | 2015 | 2018 | 0.00039992 | -0.71  (-0.81, -0.55) |
| Prevalence | Tuvalu | Male | Glaucoma | 2019 | 2021 | 0 | -1.68  (-1.78, -1.56) |
| Prevalence | Australia | Male | Glaucoma | 1990 | 1998 | 0 | -1.04  (-1.09, -1) |
| Prevalence | Australia | Male | Glaucoma | 1998 | 2005 | 0 | -0.64  (-0.69, -0.58) |
| Prevalence | Australia | Male | Glaucoma | 2005 | 2010 | 0 | 0.73  (0.67, 0.8) |
| Prevalence | Australia | Male | Glaucoma | 2010 | 2015 | 0 | -0.74  (-0.85, -0.66) |
| Prevalence | Afghanistan | Female | Glaucoma | 2008 | 2021 | 0.00239952 | -1.68  (-1.87, -1.51) |
| Prevalence | Austria | Both | Glaucoma | 1990 | 1999 | 0 | -1.29  (-1.37, -1.25) |
| Prevalence | Austria | Both | Glaucoma | 1999 | 2004 | 0 | -1.03  (-1.24, -0.89) |
| Prevalence | Austria | Both | Glaucoma | 2004 | 2009 | 0 | -0.74  (-0.94, -0.56) |
| Prevalence | Austria | Both | Glaucoma | 2009 | 2016 | 0 | -0.44  (-0.49, -0.28) |
| Prevalence | Austria | Both | Glaucoma | 2016 | 2019 | 0 | -0.72  (-0.83, -0.56) |
| Prevalence | Senegal | Both | Glaucoma | 2019 | 2021 | 0 | -1.93  (-2.39, -1.18) |
| Prevalence | Austria | Female | Glaucoma | 1990 | 2000 | 0 | -1.28  (-1.33, -1.24) |
| Prevalence | Austria | Female | Glaucoma | 2000 | 2006 | 0 | -0.95  (-1.07, -0.82) |
| Prevalence | Dominican Republic | Male | Glaucoma | 2019 | 2021 | 0 | -1.66  (-1.81, -1.49) |
| Prevalence | Austria | Male | Glaucoma | 1990 | 2000 | 0 | -1.33  (-1.37, -1.31) |
| Prevalence | Austria | Male | Glaucoma | 2000 | 2005 | 0 | -1.06  (-1.19, -0.95) |
| Prevalence | Austria | Male | Glaucoma | 2005 | 2010 | 0 | -0.76  (-0.86, -0.63) |
| Prevalence | Austria | Male | Glaucoma | 2010 | 2015 | 0 | -0.32  (-0.39, -0.21) |
| Prevalence | Austria | Male | Glaucoma | 2015 | 2019 | 0 | -0.71  (-0.84, -0.64) |
| Prevalence | Brazil | Male | Glaucoma | 2015 | 2021 | 0 | -1.64  (-1.87, -1.43) |
| Prevalence | Azerbaijan | Both | Glaucoma | 1990 | 1994 | 0 | -0.61  (-0.84, -0.46) |
| Prevalence | Azerbaijan | Both | Glaucoma | 1994 | 2001 | 0 | 0.28  (0.22, 0.36) |
| Prevalence | Azerbaijan | Both | Glaucoma | 2001 | 2006 | 0.00039992 | -0.89  (-0.98, -0.79) |
| Prevalence | Azerbaijan | Both | Glaucoma | 2006 | 2010 | 0 | -2.04  (-2.24, -1.88) |
| Prevalence | Azerbaijan | Both | Glaucoma | 2010 | 2015 | 0 | -1.39  (-1.55, -1.2) |
| Prevalence | Guyana | Both | Glaucoma | 2019 | 2021 | 0 | -1.79  (-1.92, -1.59) |
| Prevalence | Azerbaijan | Female | Glaucoma | 1990 | 1994 | 0 | -0.65  (-0.9, -0.51) |
| Prevalence | Azerbaijan | Female | Glaucoma | 1994 | 2001 | 0.00759848 | 0.14  (0.08, 0.22) |
| Prevalence | Azerbaijan | Female | Glaucoma | 2001 | 2006 | 0.00879824 | -0.94  (-1.02, -0.8) |
| Prevalence | Azerbaijan | Female | Glaucoma | 2006 | 2009 | 0 | -1.92  (-2.04, -1.64) |
| Prevalence | Azerbaijan | Female | Glaucoma | 2009 | 2014 | 0 | -1.41  (-1.53, -1.09) |
| Prevalence | Cook Islands | Male | Glaucoma | 2015 | 2021 | 0 | -1.6  (-1.68, -1.54) |
| Prevalence | Azerbaijan | Male | Glaucoma | 1990 | 1994 | 0 | -0.63  (-0.98, -0.41) |
| Prevalence | Azerbaijan | Male | Glaucoma | 1994 | 2001 | 0.00079984 | 0.2  (0.1, 0.35) |
| Prevalence | Azerbaijan | Male | Glaucoma | 2001 | 2005 | 0.00039992 | -1.03  (-1.26, -0.79) |
| Prevalence | Azerbaijan | Male | Glaucoma | 2005 | 2012 | 0 | -2.18  (-2.32, -2.09) |
| Prevalence | Azerbaijan | Male | Glaucoma | 2012 | 2017 | 0 | -1.32  (-1.56, -1.08) |
| Prevalence | United Republic of Tanzania | Female | Glaucoma | 2019 | 2021 | 0.009998 | -1.59  (-2.62, -0.39) |
| Prevalence | Bahamas | Both | Glaucoma | 1990 | 1995 | 0 | -0.45  (-0.51, -0.39) |
| Prevalence | Bahamas | Both | Glaucoma | 1995 | 2000 | 0 | -1.68  (-1.74, -1.63) |
| Prevalence | Bahamas | Both | Glaucoma | 2000 | 2005 | 0 | -0.58  (-0.65, -0.49) |
| Prevalence | Bahamas | Both | Glaucoma | 2005 | 2010 | 0 | -0.86  (-1.01, -0.78) |
| Prevalence | Bahamas | Both | Glaucoma | 2010 | 2018 | 0 | -0.59  (-0.68, -0.54) |
| Prevalence | Zambia | Both | Glaucoma | 2010 | 2021 | 0 | -1.6  (-1.7, -1.53) |
| Prevalence | Bahamas | Female | Glaucoma | 1990 | 1995 | 0 | -0.33  (-0.4, -0.25) |
| Prevalence | Bahamas | Female | Glaucoma | 1995 | 2000 | 0 | -1.75  (-1.82, -1.68) |
| Prevalence | Bahamas | Female | Glaucoma | 2000 | 2005 | 0 | -0.52  (-0.6, -0.42) |
| Prevalence | Bahamas | Female | Glaucoma | 2005 | 2010 | 0 | -0.98  (-1.18, -0.89) |
| Prevalence | Bahamas | Female | Glaucoma | 2010 | 2019 | 0 | -0.65  (-0.69, -0.59) |
| Prevalence | Lao People's Democratic Republic | Female | Glaucoma | 2014 | 2021 | 0 | -1.58  (-2.03, -1.42) |
| Prevalence | Bahamas | Male | Glaucoma | 1990 | 1995 | 0 | -0.58  (-0.7, -0.46) |
| Prevalence | Bahamas | Male | Glaucoma | 1995 | 2000 | 0 | -1.62  (-1.82, -1.49) |
| Prevalence | Bahamas | Male | Glaucoma | 2000 | 2012 | 0 | -0.75  (-0.81, -0.71) |
| Prevalence | Honduras | Male | Glaucoma | 2019 | 2021 | 0 | -1.56  (-1.77, -1.15) |
| Prevalence | Bahrain | Both | Glaucoma | 1990 | 1996 | 0 | -1.5  (-1.53, -1.46) |
| Prevalence | Bahrain | Both | Glaucoma | 1996 | 2000 | 0 | -1.72  (-1.79, -1.63) |
| Prevalence | Bahrain | Both | Glaucoma | 2000 | 2008 | 0 | -1.85  (-1.88, -1.83) |
| Prevalence | Bahrain | Both | Glaucoma | 2008 | 2012 | 0 | -1.45  (-1.5, -1.4) |
| Prevalence | Bahrain | Both | Glaucoma | 2012 | 2018 | 0 | -1.06  (-1.09, -1.03) |
| Prevalence | Guinea-Bissau | Both | Glaucoma | 2019 | 2021 | 0 | -1.57  (-1.78, -1.21) |
| Prevalence | Bahrain | Female | Glaucoma | 1990 | 1996 | 0 | -1.39  (-1.42, -1.35) |
| Prevalence | Bahrain | Female | Glaucoma | 1996 | 2000 | 0 | -1.64  (-1.72, -1.54) |
| Prevalence | Bahrain | Female | Glaucoma | 2000 | 2008 | 0 | -1.78  (-1.8, -1.76) |
| Prevalence | Bahrain | Female | Glaucoma | 2008 | 2013 | 0 | -1.35  (-1.39, -1.32) |
| Prevalence | Bahrain | Female | Glaucoma | 2013 | 2018 | 0 | -1.02  (-1.06, -0.98) |
| Prevalence | Palestine | Male | Glaucoma | 2019 | 2021 | 0.042391522 | -1.54  (-2.56, -0.29) |
| Prevalence | Bahrain | Male | Glaucoma | 1990 | 1999 | 0 | -1.67  (-1.69, -1.65) |
| Prevalence | Bahrain | Male | Glaucoma | 1999 | 2009 | 0 | -1.91  (-1.92, -1.9) |
| Prevalence | Bahrain | Male | Glaucoma | 2009 | 2012 | 0 | -1.42  (-1.6, -1.36) |
| Prevalence | Bahrain | Male | Glaucoma | 2012 | 2018 | 0 | -1.18  (-1.21, -1.14) |
| Prevalence | Eritrea | Female | Glaucoma | 2013 | 2021 | 0 | -1.52  (-1.61, -1.46) |
| Prevalence | Bangladesh | Both | Glaucoma | 1990 | 1993 | 0 | -1.62  (-2, -1.36) |
| Prevalence | Bangladesh | Both | Glaucoma | 1993 | 2000 | 0 | -1.15  (-1.21, -1.02) |
| Prevalence | Bangladesh | Both | Glaucoma | 2000 | 2005 | 0 | -3.14  (-3.21, -3.07) |
| Prevalence | Bangladesh | Both | Glaucoma | 2005 | 2015 | 0 | -1.32  (-1.36, -1.28) |
| Prevalence | Bangladesh | Both | Glaucoma | 2015 | 2019 | 0 | -1.89  (-2.13, -1.73) |
| Prevalence | Rwanda | Both | Glaucoma | 2019 | 2021 | 0 | -1.44  (-1.61, -1.18) |
| Prevalence | Bangladesh | Female | Glaucoma | 1990 | 1993 | 0 | -1.94  (-2.47, -1.47) |
| Prevalence | Bangladesh | Female | Glaucoma | 1993 | 2000 | 0 | -1.37  (-2.76, -1.06) |
| Prevalence | Bangladesh | Female | Glaucoma | 2000 | 2005 | 0 | -2.82  (-3.09, -1.42) |
| Prevalence | Bangladesh | Female | Glaucoma | 2005 | 2016 | 0 | -1.29  (-1.34, -1.18) |
| Prevalence | Bangladesh | Female | Glaucoma | 2016 | 2019 | 0 | -2.39  (-2.67, -2.03) |
| Prevalence | Botswana | Male | Glaucoma | 2015 | 2021 | 0 | -1.47  (-1.59, -1.27) |
| Prevalence | Bangladesh | Male | Glaucoma | 1990 | 1993 | 0 | -1.38  (-1.74, -1.12) |
| Prevalence | Bangladesh | Male | Glaucoma | 1993 | 2000 | 0 | -0.96  (-1.02, -0.82) |
| Prevalence | Bangladesh | Male | Glaucoma | 2000 | 2005 | 0 | -3.29  (-3.37, -3.21) |
| Prevalence | Bangladesh | Male | Glaucoma | 2005 | 2012 | 0 | -1.26  (-1.33, -1.16) |
| Prevalence | Bangladesh | Male | Glaucoma | 2012 | 2019 | 0 | -1.6  (-1.75, -1.53) |
| Prevalence | Guinea-Bissau | Female | Glaucoma | 2018 | 2021 | 0 | -1.46  (-1.62, -1.26) |
| Prevalence | Barbados | Both | Glaucoma | 1990 | 1994 | 0 | -1.28  (-1.36, -1.23) |
| Prevalence | Barbados | Both | Glaucoma | 1994 | 1999 | 0 | -0.83  (-0.9, -0.78) |
| Prevalence | Barbados | Both | Glaucoma | 1999 | 2005 | 0 | -0.42  (-0.48, -0.38) |
| Prevalence | Barbados | Both | Glaucoma | 2005 | 2010 | 0 | -0.73  (-0.83, -0.62) |
| Prevalence | Barbados | Both | Glaucoma | 2010 | 2015 | 0 | -0.55  (-0.67, -0.47) |
| Prevalence | Palestine | Both | Glaucoma | 2019 | 2021 | 0.039192162 | -1.44  (-2.39, -0.36) |
| Prevalence | Barbados | Female | Glaucoma | 1990 | 1994 | 0 | -1.33  (-1.58, -1.21) |
| Prevalence | Barbados | Female | Glaucoma | 1994 | 1999 | 0 | -0.8  (-0.99, -0.65) |
| Prevalence | Barbados | Female | Glaucoma | 1999 | 2005 | 0 | -0.4  (-0.48, -0.23) |
| Prevalence | Barbados | Female | Glaucoma | 2005 | 2010 | 0 | -0.89  (-1.11, -0.77) |
| Prevalence | Barbados | Female | Glaucoma | 2010 | 2019 | 0 | -0.47  (-0.59, -0.42) |
| Prevalence | Tonga | Male | Glaucoma | 2015 | 2021 | 0 | -1.45  (-1.58, -1.37) |
| Prevalence | Barbados | Male | Glaucoma | 1990 | 1994 | 0 | -1.27  (-1.31, -1.23) |
| Prevalence | Barbados | Male | Glaucoma | 1994 | 1999 | 0 | -0.87  (-0.9, -0.84) |
| Prevalence | Barbados | Male | Glaucoma | 1999 | 2010 | 0 | -0.53  (-0.54, -0.52) |
| Prevalence | Barbados | Male | Glaucoma | 2010 | 2015 | 0 | -0.74  (-0.8, -0.7) |
| Prevalence | Barbados | Male | Glaucoma | 2015 | 2019 | 0 | -0.45  (-0.49, -0.37) |
| Prevalence | Egypt | Male | Glaucoma | 2015 | 2021 | 0 | -1.45  (-1.52, -1.38) |
| Prevalence | Belarus | Both | Glaucoma | 1990 | 1994 | 0 | -0.66  (-0.87, -0.55) |
| Prevalence | Belarus | Both | Glaucoma | 1994 | 2000 | 0 | -0.35  (-0.39, -0.26) |
| Prevalence | Belarus | Both | Glaucoma | 2000 | 2018 | 0 | -1.05  (-1.06, -1.04) |
| Prevalence | Eritrea | Both | Glaucoma | 2013 | 2021 | 0 | -1.37  (-1.41, -1.34) |
| Prevalence | Belarus | Female | Glaucoma | 1990 | 1993 | 0 | -0.62  (-0.75, -0.52) |
| Prevalence | Belarus | Female | Glaucoma | 1993 | 2001 | 0 | -0.45  (-0.47, -0.41) |
| Prevalence | Belarus | Female | Glaucoma | 2001 | 2005 | 0 | -1.29  (-1.34, -1.24) |
| Prevalence | Belarus | Female | Glaucoma | 2005 | 2009 | 0 | -0.73  (-0.8, -0.64) |
| Prevalence | Belarus | Female | Glaucoma | 2009 | 2018 | 0 | -0.98  (-1.01, -0.96) |
| Prevalence | Madagascar | Male | Glaucoma | 2018 | 2021 | 0.00039992 | -1.45  (-2.59, -0.83) |
| Prevalence | Belarus | Male | Glaucoma | 1990 | 1994 | 0 | -0.68  (-0.8, -0.6) |
| Prevalence | Belarus | Male | Glaucoma | 1994 | 2001 | 0 | -0.26  (-0.29, -0.22) |
| Prevalence | Belarus | Male | Glaucoma | 2001 | 2006 | 0 | -1.15  (-1.2, -1.07) |
| Prevalence | Belarus | Male | Glaucoma | 2006 | 2015 | 0 | -1.3  (-1.34, -1.28) |
| Prevalence | Belarus | Male | Glaucoma | 2015 | 2019 | 0 | -0.84  (-0.91, -0.76) |
| Prevalence | Cambodia | Male | Glaucoma | 2015 | 2021 | 0 | -1.43  (-1.63, -1.24) |
| Prevalence | Belgium | Both | Glaucoma | 1990 | 1995 | 0 | -1.2  (-1.29, -1.15) |
| Prevalence | Belgium | Both | Glaucoma | 1995 | 2005 | 0 | -0.88  (-0.91, -0.85) |
| Prevalence | Belgium | Both | Glaucoma | 2005 | 2010 | 0 | -0.61  (-0.66, -0.47) |
| Prevalence | Belgium | Both | Glaucoma | 2010 | 2019 | 0 | -0.78  (-0.83, -0.76) |
| Prevalence | United Republic of Tanzania | Both | Glaucoma | 2019 | 2021 | 0.116376725 | -1.36  (-2.52, 0.21) |
| Prevalence | Belgium | Female | Glaucoma | 1990 | 1994 | 0 | -1.26  (-1.48, -1.15) |
| Prevalence | Belgium | Female | Glaucoma | 1994 | 2004 | 0 | -0.91  (-0.95, -0.87) |
| Prevalence | Belgium | Female | Glaucoma | 2004 | 2011 | 0 | -0.69  (-0.73, -0.52) |
| Prevalence | Belgium | Female | Glaucoma | 2011 | 2019 | 0 | -0.86  (-1.01, -0.82) |
| Prevalence | Sierra Leone | Male | Glaucoma | 2008 | 2021 | 0 | -1.43  (-1.5, -1.36) |
| Prevalence | Belgium | Male | Glaucoma | 1990 | 1994 | 0 | -1.27  (-1.41, -1.2) |
| Prevalence | Belgium | Male | Glaucoma | 1994 | 2005 | 0 | -0.94  (-0.97, -0.92) |
| Prevalence | Belgium | Male | Glaucoma | 2005 | 2010 | 0 | -0.61  (-0.66, -0.46) |
| Prevalence | Belgium | Male | Glaucoma | 2010 | 2018 | 0 | -0.77  (-0.84, -0.74) |
| Prevalence | Myanmar | Male | Glaucoma | 2014 | 2021 | 0 | -1.42  (-1.54, -1.31) |
| Prevalence | Belize | Both | Glaucoma | 1990 | 1994 | 0 | -1.35  (-1.42, -1.28) |
| Prevalence | Belize | Both | Glaucoma | 1994 | 2000 | 0 | -0.73  (-0.77, -0.69) |
| Prevalence | Belize | Both | Glaucoma | 2000 | 2009 | 0 | -1.21  (-1.24, -1.19) |
| Prevalence | Belize | Both | Glaucoma | 2009 | 2016 | 0 | -0.88  (-0.93, -0.83) |
| Prevalence | Egypt | Both | Glaucoma | 2015 | 2021 | 0 | -1.36  (-1.45, -1.28) |
| Prevalence | Belize | Female | Glaucoma | 1990 | 1994 | 0 | -1.31  (-1.37, -1.25) |
| Prevalence | Belize | Female | Glaucoma | 1994 | 2001 | 0 | -0.8  (-0.83, -0.76) |
| Prevalence | Belize | Female | Glaucoma | 2001 | 2005 | 0 | -1.13  (-1.22, -0.97) |
| Prevalence | Belize | Female | Glaucoma | 2005 | 2010 | 0 | -1.3  (-1.36, -1.25) |
| Prevalence | Belize | Female | Glaucoma | 2010 | 2019 | 0 | -0.79  (-0.81, -0.77) |
| Prevalence | Saint Vincent and the Grenadines | Male | Glaucoma | 2019 | 2021 | 0 | -1.4  (-1.54, -1.16) |
| Prevalence | Belize | Male | Glaucoma | 1990 | 1994 | 0 | -1.48  (-1.58, -1.39) |
| Prevalence | Belize | Male | Glaucoma | 1994 | 2000 | 0 | -0.69  (-0.75, -0.62) |
| Prevalence | Belize | Male | Glaucoma | 2000 | 2007 | 0 | -1.34  (-1.42, -1.24) |
| Prevalence | Belize | Male | Glaucoma | 2007 | 2015 | 0 | -0.98  (-1.33, -0.93) |
| Prevalence | Belize | Male | Glaucoma | 2015 | 2019 | 0 | -0.61  (-0.98, -0.45) |
| Prevalence | Antigua and Barbuda | Male | Glaucoma | 2019 | 2021 | 0 | -1.4  (-1.48, -1.33) |
| Prevalence | Benin | Both | Glaucoma | 1990 | 1992 | 0 | 2.99  (2.73, 3.24) |
| Prevalence | Benin | Both | Glaucoma | 1992 | 1995 | 0 | 2.05  (1.77, 2.19) |
| Prevalence | Benin | Both | Glaucoma | 1995 | 2000 | 0 | 0.98  (0.82, 1.06) |
| Prevalence | Benin | Both | Glaucoma | 2000 | 2005 | 0.00719856 | 1.26  (1.18, 1.4) |
| Prevalence | Benin | Both | Glaucoma | 2005 | 2016 | 0.00919816 | 0.06  (0.03, 0.09) |
| Prevalence | Iraq | Both | Glaucoma | 2018 | 2021 | 0 | -1.33  (-1.42, -1.19) |
| Prevalence | Benin | Female | Glaucoma | 1990 | 1992 | 0 | 3.28  (2.98, 3.57) |
| Prevalence | Benin | Female | Glaucoma | 1992 | 1995 | 0 | 2.3  (1.88, 2.45) |
| Prevalence | Benin | Female | Glaucoma | 1995 | 2000 | 0 | 1.17  (0.98, 1.26) |
| Prevalence | Benin | Female | Glaucoma | 2000 | 2005 | 0.01159768 | 1.5  (1.41, 1.63) |
| Prevalence | Benin | Female | Glaucoma | 2005 | 2019 | 0.00439912 | -0.06  (-0.08, -0.03) |
| Prevalence | Palestine | Female | Glaucoma | 2019 | 2021 | 0.019196161 | -1.4  (-2.2, -0.55) |
| Prevalence | Benin | Male | Glaucoma | 1990 | 1994 | 0 | 2.44  (2.33, 2.57) |
| Prevalence | Benin | Male | Glaucoma | 1994 | 2005 | 0 | 1.07  (1.03, 1.1) |
| Prevalence | Benin | Male | Glaucoma | 2005 | 2015 | 0 | 0.29  (0.25, 0.33) |
| Prevalence | Cabo Verde | Female | Glaucoma | 2010 | 2021 | 0 | -1.37  (-1.47, -1.27) |
| Prevalence | Bermuda | Both | Glaucoma | 1990 | 1999 | 0 | -1.3  (-1.31, -1.3) |
| Prevalence | Bermuda | Both | Glaucoma | 1999 | 2002 | 0 | -1.08  (-1.19, -1.05) |
| Prevalence | Bermuda | Both | Glaucoma | 2002 | 2009 | 0 | -0.98  (-0.99, -0.96) |
| Prevalence | Bermuda | Both | Glaucoma | 2009 | 2014 | 0 | -0.85  (-0.87, -0.82) |
| Prevalence | Bermuda | Both | Glaucoma | 2014 | 2017 | 0 | -0.51  (-0.54, -0.48) |
| Prevalence | Lao People's Democratic Republic | Both | Glaucoma | 2007 | 2021 | 0 | -1.32  (-1.36, -1.28) |
| Prevalence | Bermuda | Female | Glaucoma | 1990 | 2000 | 0 | -1.3  (-1.31, -1.29) |
| Prevalence | Bermuda | Female | Glaucoma | 2000 | 2005 | 0 | -0.92  (-0.95, -0.88) |
| Prevalence | Bermuda | Female | Glaucoma | 2005 | 2010 | 0 | -1.09  (-1.14, -1.06) |
| Prevalence | Bermuda | Female | Glaucoma | 2010 | 2015 | 0 | -0.75  (-0.79, -0.72) |
| Prevalence | Bermuda | Female | Glaucoma | 2015 | 2019 | 0 | -0.5  (-0.55, -0.44) |
| Prevalence | Djibouti | Male | Glaucoma | 2015 | 2021 | 0 | -1.35  (-1.47, -1.28) |
| Prevalence | Bermuda | Male | Glaucoma | 1990 | 1998 | 0 | -1.36  (-1.39, -1.34) |
| Prevalence | Bermuda | Male | Glaucoma | 1998 | 2004 | 0 | -1.19  (-1.24, -1.13) |
| Prevalence | Bermuda | Male | Glaucoma | 2004 | 2015 | 0 | -0.9  (-0.91, -0.88) |
| Prevalence | Bermuda | Male | Glaucoma | 2015 | 2019 | 0 | -0.32  (-0.36, -0.27) |
| Prevalence | Guyana | Female | Glaucoma | 2018 | 2021 | 0 | -1.35  (-1.48, -1.27) |
| Prevalence | Bhutan | Both | Glaucoma | 1990 | 1995 | 0.00159968 | -0.82  (-1.09, -0.53) |
| Prevalence | Bhutan | Both | Glaucoma | 1995 | 2000 | 0 | -5.09  (-5.27, -4.88) |
| Prevalence | Bhutan | Both | Glaucoma | 2000 | 2019 | 0 | -1.05  (-1.12, -1.01) |
| Prevalence | Honduras | Both | Glaucoma | 2019 | 2021 | 0 | -1.31  (-1.41, -1.17) |
| Prevalence | Bhutan | Female | Glaucoma | 1990 | 1995 | 0.00119976 | -0.72  (-0.98, -0.45) |
| Prevalence | Bhutan | Female | Glaucoma | 1995 | 2000 | 0 | -5.17  (-5.35, -4.97) |
| Prevalence | Bhutan | Female | Glaucoma | 2000 | 2019 | 0 | -0.94  (-1, -0.9) |
| Prevalence | Iraq | Male | Glaucoma | 2019 | 2021 | 0 | -1.34  (-1.5, -1.23) |
| Prevalence | Bhutan | Male | Glaucoma | 1990 | 1995 | 0.00119976 | -1.09  (-1.31, -0.84) |
| Prevalence | Bhutan | Male | Glaucoma | 1995 | 2000 | 0 | -5.4  (-5.58, -5.18) |
| Prevalence | Bhutan | Male | Glaucoma | 2000 | 2006 | 0 | -0.99  (-1.16, -0.7) |
| Prevalence | Bhutan | Male | Glaucoma | 2006 | 2018 | 0 | -1.26  (-1.66, -1.2) |
| Prevalence | Morocco | Male | Glaucoma | 2007 | 2021 | 0 | -1.33  (-1.35, -1.32) |
| Prevalence | Bolivia (Plurinational State of) | Both | Glaucoma | 1990 | 1995 | 0 | -1.16  (-1.22, -1.09) |
| Prevalence | Bolivia (Plurinational State of) | Both | Glaucoma | 1995 | 2000 | 0 | -1.46  (-1.55, -1.4) |
| Prevalence | Bolivia (Plurinational State of) | Both | Glaucoma | 2000 | 2009 | 0 | -1.11  (-1.14, -1.09) |
| Prevalence | Bolivia (Plurinational State of) | Both | Glaucoma | 2009 | 2015 | 0 | -0.79  (-1, -0.73) |
| Prevalence | Bolivia (Plurinational State of) | Both | Glaucoma | 2015 | 2018 | 0 | -1.13  (-1.19, -0.82) |
| Prevalence | Cabo Verde | Both | Glaucoma | 2010 | 2021 | 0 | -1.31  (-1.39, -1.22) |
| Prevalence | Bolivia (Plurinational State of) | Female | Glaucoma | 1990 | 1995 | 0 | -1.08  (-1.13, -1.02) |
| Prevalence | Bolivia (Plurinational State of) | Female | Glaucoma | 1995 | 2000 | 0 | -1.53  (-1.63, -1.47) |
| Prevalence | Bolivia (Plurinational State of) | Female | Glaucoma | 2000 | 2008 | 0 | -1.07  (-1.11, -1.03) |
| Prevalence | Bolivia (Plurinational State of) | Female | Glaucoma | 2008 | 2019 | 0 | -0.86  (-0.88, -0.84) |
| Prevalence | Turkey | Male | Glaucoma | 2011 | 2021 | 0 | -1.33  (-1.4, -1.25) |
| Prevalence | Bolivia (Plurinational State of) | Male | Glaucoma | 1990 | 1995 | 0 | -1.29  (-1.33, -1.19) |
| Prevalence | Bolivia (Plurinational State of) | Male | Glaucoma | 1995 | 2000 | 0 | -1.4  (-1.48, -1.34) |
| Prevalence | Bolivia (Plurinational State of) | Male | Glaucoma | 2000 | 2009 | 0 | -1.17  (-1.19, -1.15) |
| Prevalence | Bolivia (Plurinational State of) | Male | Glaucoma | 2009 | 2015 | 0 | -0.77  (-0.8, -0.75) |
| Prevalence | Bolivia (Plurinational State of) | Male | Glaucoma | 2015 | 2018 | 0 | -1.36  (-1.41, -1.3) |
| Prevalence | Egypt | Female | Glaucoma | 2015 | 2021 | 0 | -1.32  (-1.44, -1.2) |
| Prevalence | Bosnia and Herzegovina | Both | Glaucoma | 1990 | 1993 | 0.00239952 | 0.33  (0.16, 0.69) |
| Prevalence | Bosnia and Herzegovina | Both | Glaucoma | 1993 | 1996 | 0 | -0.47  (-0.63, -0.28) |
| Prevalence | Bosnia and Herzegovina | Both | Glaucoma | 1996 | 2000 | 0 | -3.21  (-3.33, -3.09) |
| Prevalence | Bosnia and Herzegovina | Both | Glaucoma | 2000 | 2004 | 0 | -1.71  (-1.9, -1.57) |
| Prevalence | Bosnia and Herzegovina | Both | Glaucoma | 2004 | 2009 | 0 | -1.17  (-1.32, -0.99) |
| Prevalence | Djibouti | Both | Glaucoma | 2016 | 2021 | 0 | -1.3  (-1.38, -1.24) |
| Prevalence | Bosnia and Herzegovina | Female | Glaucoma | 1990 | 1993 | 0.772645471 | 0.01  (-0.29, 0.34) |
| Prevalence | Bosnia and Herzegovina | Female | Glaucoma | 1993 | 1996 | 0 | -0.52  (-3.08, -0.37) |
| Prevalence | Bosnia and Herzegovina | Female | Glaucoma | 1996 | 2000 | 0 | -2.99  (-3.1, -1.56) |
| Prevalence | Bosnia and Herzegovina | Female | Glaucoma | 2000 | 2006 | 0 | -1.58  (-1.71, -0.92) |
| Prevalence | Bosnia and Herzegovina | Female | Glaucoma | 2006 | 2018 | 0 | -0.9  (-0.95, -0.85) |
| Prevalence | Nauru | Male | Glaucoma | 2019 | 2021 | 0 | -1.31  (-1.53, -1.17) |
| Prevalence | Bosnia and Herzegovina | Male | Glaucoma | 1990 | 1993 | 0 | 0.54  (0.37, 0.88) |
| Prevalence | Bosnia and Herzegovina | Male | Glaucoma | 1993 | 1996 | 0 | -0.35  (-0.51, -0.14) |
| Prevalence | Bosnia and Herzegovina | Male | Glaucoma | 1996 | 2000 | 0 | -3.48  (-3.6, -3.36) |
| Prevalence | Bosnia and Herzegovina | Male | Glaucoma | 2000 | 2004 | 0 | -1.67  (-1.87, -1.53) |
| Prevalence | Bosnia and Herzegovina | Male | Glaucoma | 2004 | 2009 | 0 | -1.21  (-1.36, -0.93) |
| Prevalence | Rwanda | Female | Glaucoma | 2019 | 2021 | 0 | -1.3  (-1.46, -1.06) |
| Prevalence | Botswana | Both | Glaucoma | 1990 | 2005 | 0 | -1.06  (-1.09, -1.04) |
| Prevalence | Botswana | Both | Glaucoma | 2005 | 2010 | 0 | -2.06  (-2.21, -1.16) |
| Prevalence | Botswana | Both | Glaucoma | 2010 | 2014 | 0 | -1.65  (-1.97, -1.41) |
| Prevalence | Eswatini | Both | Glaucoma | 2015 | 2021 | 0 | -1.28  (-1.32, -1.2) |
| Prevalence | Botswana | Female | Glaucoma | 1990 | 1994 | 0 | -1.18  (-1.43, -1.03) |
| Prevalence | Botswana | Female | Glaucoma | 1994 | 2005 | 0 | -0.62  (-0.65, -0.57) |
| Prevalence | Botswana | Female | Glaucoma | 2005 | 2011 | 0 | -2.1  (-2.22, -1.66) |
| Prevalence | Botswana | Female | Glaucoma | 2011 | 2015 | 0 | -1.66  (-2, -1.42) |
| Prevalence | Eritrea | Male | Glaucoma | 2016 | 2021 | 0 | -1.29  (-1.33, -1.24) |
| Prevalence | Botswana | Male | Glaucoma | 1990 | 2005 | 0 | -0.93  (-0.96, -0.91) |
| Prevalence | Botswana | Male | Glaucoma | 2005 | 2015 | 0 | -1.94  (-2.01, -1.89) |
| Prevalence | Bolivia (Plurinational State of) | Female | Glaucoma | 2019 | 2021 | 0 | -1.26  (-1.39, -1.1) |
| Prevalence | Brazil | Both | Glaucoma | 1990 | 1992 | 0 | -5.63  (-6.61, -3.96) |
| Prevalence | Brazil | Both | Glaucoma | 1992 | 1995 | 0.24795041 | -3.34  (-3.76, 1.32) |
| Prevalence | Brazil | Both | Glaucoma | 1995 | 2001 | 0.188762248 | 1  (-1.13, 1.79) |
| Prevalence | Brazil | Both | Glaucoma | 2001 | 2010 | 0.102379524 | -0.88  (-1.22, 2.02) |
| Prevalence | Brazil | Both | Glaucoma | 2010 | 2015 | 0.067186563 | 2  (-1.58, 2.69) |
| Prevalence | Botswana | Both | Glaucoma | 2014 | 2021 | 0 | -1.26  (-1.32, -1.11) |
| Prevalence | Brazil | Female | Glaucoma | 1990 | 1994 | 0 | -5.42  (-5.84, -4.98) |
| Prevalence | Brazil | Female | Glaucoma | 1994 | 2003 | 0.009998 | 0.68  (0.44, 0.92) |
| Prevalence | Brazil | Female | Glaucoma | 2003 | 2010 | 0.069186163 | -0.94  (-1.39, 0.38) |
| Prevalence | Brazil | Female | Glaucoma | 2010 | 2015 | 0.115176965 | 2.62  (-0.99, 3.1) |
| Prevalence | Brazil | Female | Glaucoma | 2015 | 2019 | 0.128774245 | -2.79  (-3.54, 2.57) |
| Prevalence | Panama | Male | Glaucoma | 2014 | 2021 | 0 | -1.26  (-1.3, -1.21) |
| Prevalence | Brazil | Male | Glaucoma | 1990 | 1992 | 0 | -5.03  (-5.74, -3.85) |
| Prevalence | Brazil | Male | Glaucoma | 1992 | 1995 | 0.097580484 | -3.16  (-3.53, 1.26) |
| Prevalence | Brazil | Male | Glaucoma | 1995 | 2000 | 0.081983603 | 1.09  (-0.91, 1.51) |
| Prevalence | Brazil | Male | Glaucoma | 2000 | 2010 | 0.037592482 | -0.92  (-1.05, -0.7) |
| Prevalence | Brazil | Male | Glaucoma | 2010 | 2015 | 0.027594481 | 1.58  (1.19, 1.86) |
| Prevalence | Guatemala | Male | Glaucoma | 2017 | 2021 | 0 | -1.26  (-1.48, -1.11) |
| Prevalence | Brunei Darussalam | Both | Glaucoma | 1990 | 1994 | 0 | -1.8  (-1.94, -1.73) |
| Prevalence | Brunei Darussalam | Both | Glaucoma | 1994 | 1998 | 0 | -1.34  (-1.56, -1.22) |
| Prevalence | Brunei Darussalam | Both | Glaucoma | 1998 | 2005 | 0 | -1.05  (-1.16, -1) |
| Prevalence | Brunei Darussalam | Both | Glaucoma | 2005 | 2008 | 0 | -0.89  (-1.03, -0.53) |
| Prevalence | Brunei Darussalam | Both | Glaucoma | 2008 | 2015 | 0 | -0.52  (-0.6, -0.43) |
| Prevalence | Sierra Leone | Both | Glaucoma | 2009 | 2021 | 0 | -1.23  (-1.32, -1.16) |
| Prevalence | Brunei Darussalam | Female | Glaucoma | 1990 | 1995 | 0 | -1.48  (-1.64, -1.19) |
| Prevalence | Brunei Darussalam | Female | Glaucoma | 1995 | 1999 | 0 | -1.99  (-2.29, -1.02) |
| Prevalence | Brunei Darussalam | Female | Glaucoma | 1999 | 2012 | 0.013597281 | -0.76  (-0.82, -0.7) |
| Prevalence | Djibouti | Female | Glaucoma | 2016 | 2021 | 0 | -1.24  (-1.28, -1.2) |
| Prevalence | Brunei Darussalam | Male | Glaucoma | 1990 | 1994 | 0 | -1.84  (-1.94, -1.75) |
| Prevalence | Brunei Darussalam | Male | Glaucoma | 1994 | 2000 | 0 | -1  (-1.06, -0.91) |
| Prevalence | Brunei Darussalam | Male | Glaucoma | 2000 | 2005 | 0 | -1.5  (-1.63, -1.41) |
| Prevalence | Brunei Darussalam | Male | Glaucoma | 2005 | 2009 | 0 | -0.7  (-0.89, -0.53) |
| Prevalence | Brunei Darussalam | Male | Glaucoma | 2009 | 2017 | 0.00079984 | -0.26  (-0.3, -0.18) |
| Prevalence | Senegal | Female | Glaucoma | 2015 | 2021 | 0.00159968 | -1.24  (-1.57, -1.01) |
| Prevalence | Bulgaria | Both | Glaucoma | 1990 | 1994 | 0 | -1.4  (-1.52, -1.29) |
| Prevalence | Bulgaria | Both | Glaucoma | 1994 | 2000 | 0 | -0.35  (-0.42, -0.28) |
| Prevalence | Bulgaria | Both | Glaucoma | 2000 | 2005 | 0 | 0.84  (0.76, 0.91) |
| Prevalence | Bulgaria | Both | Glaucoma | 2005 | 2014 | 0 | -1.01  (-1.07, -0.96) |
| Prevalence | Gambia | Both | Glaucoma | 2010 | 2021 | 0.0039992 | -1.2  (-1.42, -1.01) |
| Prevalence | Bulgaria | Female | Glaucoma | 1990 | 1993 | 0 | -1.58  (-1.93, -1.39) |
| Prevalence | Bulgaria | Female | Glaucoma | 1993 | 1996 | 0.009998 | -0.6  (-1.03, -0.16) |
| Prevalence | Bulgaria | Female | Glaucoma | 1996 | 2000 | 0.093981204 | 0.09  (-0.07, 0.62) |
| Prevalence | Bulgaria | Female | Glaucoma | 2000 | 2005 | 0.038392322 | 0.89  (0.78, 1.05) |
| Prevalence | Bulgaria | Female | Glaucoma | 2005 | 2015 | 0.00039992 | -0.96  (-1.02, -0.91) |
| Prevalence | Botswana | Female | Glaucoma | 2015 | 2021 | 0 | -1.23  (-1.32, -1.07) |
| Prevalence | Bulgaria | Male | Glaucoma | 1990 | 1994 | 0 | -1.12  (-1.3, -1.02) |
| Prevalence | Bulgaria | Male | Glaucoma | 1994 | 2000 | 0 | -0.44  (-0.5, -0.37) |
| Prevalence | Bulgaria | Male | Glaucoma | 2000 | 2005 | 0 | 0.86  (0.8, 0.91) |
| Prevalence | Bulgaria | Male | Glaucoma | 2005 | 2014 | 0 | -0.98  (-1.03, -0.94) |
| Prevalence | Cabo Verde | Male | Glaucoma | 2010 | 2021 | 0 | -1.22  (-1.3, -1.15) |
| Prevalence | Burkina Faso | Both | Glaucoma | 1990 | 1992 | 0 | 11.17  (10.38, 12) |
| Prevalence | Burkina Faso | Both | Glaucoma | 1992 | 1995 | 0 | 4.78  (4.34, 5.21) |
| Prevalence | Burkina Faso | Both | Glaucoma | 1995 | 2000 | 0.00319936 | 0.55  (0.26, 0.92) |
| Prevalence | Burkina Faso | Both | Glaucoma | 2000 | 2014 | 0.00039992 | -0.77  (-1.08, -0.7) |
| Prevalence | Tunisia | Both | Glaucoma | 2014 | 2021 | 0 | -1.19  (-1.24, -1.11) |
| Prevalence | Burkina Faso | Female | Glaucoma | 1990 | 1992 | 0 | 10.43  (9.6, 11.36) |
| Prevalence | Burkina Faso | Female | Glaucoma | 1992 | 1995 | 0 | 4.91  (4.41, 5.38) |
| Prevalence | Burkina Faso | Female | Glaucoma | 1995 | 2000 | 0 | 0.81  (0.52, 1.19) |
| Prevalence | Burkina Faso | Female | Glaucoma | 2000 | 2010 | 0 | -0.47  (-0.57, -0.31) |
| Prevalence | Burkina Faso | Female | Glaucoma | 2010 | 2014 | 0 | -1.3  (-1.75, -0.92) |
| Prevalence | Philippines | Male | Glaucoma | 2019 | 2021 | 0 | -1.22  (-1.41, -1.06) |
| Prevalence | Burkina Faso | Male | Glaucoma | 1990 | 1992 | 0 | 11.59  (10.76, 12.45) |
| Prevalence | Burkina Faso | Male | Glaucoma | 1992 | 1995 | 0 | 4.73  (4.26, 5.18) |
| Prevalence | Burkina Faso | Male | Glaucoma | 1995 | 2000 | 0.073585283 | 0.27  (-0.02, 0.66) |
| Prevalence | Burkina Faso | Male | Glaucoma | 2000 | 2010 | 0 | -0.92  (-1.27, -0.81) |
| Prevalence | Indonesia | Female | Glaucoma | 2015 | 2021 | 0 | -1.22  (-1.35, -1.09) |
| Prevalence | Turkey | Both | Glaucoma | 2010 | 2021 | 0 | -1.18  (-1.23, -1.12) |
| Prevalence | Tokelau | Male | Glaucoma | 2019 | 2021 | 0 | -1.22  (-1.39, -1.12) |
| Prevalence | Burundi | Male | Glaucoma | 1990 | 1995 | 0.358728254 | 0.09  (-0.19, 0.72) |
| Prevalence | Burundi | Male | Glaucoma | 1995 | 2001 | 0.034393121 | -0.51  (-1.02, -0.27) |
| Prevalence | Burundi | Male | Glaucoma | 2001 | 2009 | 0.336332733 | 0.06  (-0.12, 0.58) |
| Prevalence | Solomon Islands | Male | Glaucoma | 2015 | 2021 | 0 | -1.22  (-1.27, -1.18) |
| Prevalence | Cabo Verde | Both | Glaucoma | 1990 | 1996 | 0.00519896 | -0.72  (-0.92, -0.41) |
| Prevalence | Cabo Verde | Both | Glaucoma | 1996 | 2005 | 0 | -1.37  (-1.51, -1.27) |
| Prevalence | Cabo Verde | Both | Glaucoma | 2005 | 2010 | 0 | 3.88  (3.72, 4.06) |
| Prevalence | Syrian Arab Republic | Both | Glaucoma | 2015 | 2021 | 0 | -1.17  (-1.22, -1.13) |
| Prevalence | Cabo Verde | Female | Glaucoma | 1990 | 1995 | 0.087582484 | -0.56  (-0.87, 0.14) |
| Prevalence | Cabo Verde | Female | Glaucoma | 1995 | 2005 | 0.026794641 | -1.1  (-1.32, -1) |
| Prevalence | Cabo Verde | Female | Glaucoma | 2005 | 2010 | 0.026394721 | 4.52  (4.31, 4.73) |
| Prevalence | Saint Kitts and Nevis | Male | Glaucoma | 2019 | 2021 | 0 | -1.21  (-1.45, -0.9) |
| Prevalence | Cabo Verde | Male | Glaucoma | 1990 | 1997 | 0.00079984 | -0.79  (-0.93, -0.59) |
| Prevalence | Cabo Verde | Male | Glaucoma | 1997 | 2005 | 0 | -1.48  (-1.62, -1.38) |
| Prevalence | Cabo Verde | Male | Glaucoma | 2005 | 2010 | 0 | 3.02  (2.87, 3.17) |
| Prevalence | Marshall Islands | Male | Glaucoma | 2018 | 2021 | 0 | -1.21  (-1.3, -1.1) |
| Prevalence | Cambodia | Both | Glaucoma | 1990 | 1994 | 0 | -4.15  (-4.35, -3.94) |
| Prevalence | Cambodia | Both | Glaucoma | 1994 | 2001 | 0 | -0.81  (-0.92, -0.69) |
| Prevalence | Cambodia | Both | Glaucoma | 2001 | 2009 | 0 | -1.75  (-1.85, -1.66) |
| Prevalence | Cambodia | Both | Glaucoma | 2009 | 2015 | 0.229154169 | 0.06  (-0.06, 0.24) |
| Prevalence | Guatemala | Both | Glaucoma | 2017 | 2021 | 0 | -1.1  (-1.42, -0.95) |
| Prevalence | Cambodia | Female | Glaucoma | 1990 | 1994 | 0 | -4.07  (-4.29, -3.84) |
| Prevalence | Cambodia | Female | Glaucoma | 1994 | 2000 | 0 | -0.66  (-0.8, -0.47) |
| Prevalence | Cambodia | Female | Glaucoma | 2000 | 2009 | 0 | -1.45  (-1.56, -1.36) |
| Prevalence | Cambodia | Female | Glaucoma | 2009 | 2016 | 0.524295141 | -0.04  (-0.16, 0.18) |
| Prevalence | Syrian Arab Republic | Male | Glaucoma | 2015 | 2021 | 0 | -1.2  (-1.26, -1.16) |
| Prevalence | Cambodia | Male | Glaucoma | 1990 | 1994 | 0 | -4.22  (-4.45, -3.96) |
| Prevalence | Cambodia | Male | Glaucoma | 1994 | 2001 | 0 | -0.85  (-0.99, -0.71) |
| Prevalence | Cambodia | Male | Glaucoma | 2001 | 2009 | 0 | -1.94  (-2.04, -1.83) |
| Prevalence | Cambodia | Male | Glaucoma | 2009 | 2015 | 0.017596481 | 0.21  (0.05, 0.37) |
| Prevalence | Gambia | Female | Glaucoma | 2010 | 2021 | 0.00079984 | -1.2  (-1.41, -1.01) |
| Prevalence | Cameroon | Both | Glaucoma | 1990 | 2001 | 0.00759848 | -0.17  (-0.24, -0.1) |
| Prevalence | Cameroon | Both | Glaucoma | 2001 | 2004 | 0.00559888 | -3.65  (-3.92, -3.39) |
| Prevalence | Cameroon | Both | Glaucoma | 2004 | 2011 | 0.305538892 | -0.09  (-0.21, 0.11) |
| Prevalence | Cameroon | Both | Glaucoma | 2011 | 2014 | 0.00319936 | -0.99  (-1.24, -0.51) |
| Prevalence | Dominican Republic | Both | Glaucoma | 2015 | 2021 | 0 | -1.1  (-1.16, -1.04) |
| Prevalence | Cameroon | Female | Glaucoma | 1990 | 1995 | 0.00559888 | -0.22  (-0.57, -0.08) |
| Prevalence | Cameroon | Female | Glaucoma | 1995 | 2000 | 0.027194561 | 0.15  (0.03, 0.37) |
| Prevalence | Cameroon | Female | Glaucoma | 2000 | 2005 | 0.022795441 | -2.27  (-2.38, -2.16) |
| Prevalence | Cameroon | Female | Glaucoma | 2005 | 2010 | 0.022395521 | 0.34  (0.19, 0.5) |
| Prevalence | Cameroon | Female | Glaucoma | 2010 | 2014 | 0.013197361 | -0.78  (-1.05, -0.51) |
| Prevalence | Saudi Arabia | Male | Glaucoma | 2015 | 2021 | 0 | -1.18  (-1.38, -0.9) |
| Prevalence | Cameroon | Male | Glaucoma | 1990 | 2001 | 0.00759848 | -0.19  (-0.29, -0.06) |
| Prevalence | Cameroon | Male | Glaucoma | 2001 | 2004 | 0 | -3.95  (-4.28, -3.08) |
| Prevalence | Puerto Rico | Male | Glaucoma | 2019 | 2021 | 0 | -1.18  (-1.36, -0.94) |
| Prevalence | Canada | Both | Glaucoma | 1990 | 1996 | 0.012797441 | -0.25  (-0.33, -0.12) |
| Prevalence | Canada | Both | Glaucoma | 1996 | 2004 | 0.00079984 | -0.55  (-0.72, -0.5) |
| Prevalence | Mauritania | Both | Glaucoma | 2019 | 2021 | 0 | -1.07  (-1.15, -0.99) |
| Prevalence | Canada | Female | Glaucoma | 1990 | 1996 | 0.01079784 | -0.24  (-0.31, -0.16) |
| Prevalence | Canada | Female | Glaucoma | 1996 | 2003 | 0.00119976 | -0.5  (-0.65, -0.29) |
| Prevalence | Canada | Female | Glaucoma | 2003 | 2009 | 0 | -0.26  (-0.49, -0.19) |
| Prevalence | Jamaica | Male | Glaucoma | 2019 | 2021 | 0 | -1.16  (-1.27, -1) |
| Prevalence | Canada | Male | Glaucoma | 1990 | 1995 | 0.076784643 | -0.21  (-0.34, 0.04) |
| Prevalence | Canada | Male | Glaucoma | 1995 | 2005 | 0.00919816 | -0.6  (-0.73, -0.54) |
| Prevalence | Canada | Male | Glaucoma | 2005 | 2015 | 0.166766647 | -0.07  (-0.14, 0.21) |
| Prevalence | Zambia | Female | Glaucoma | 2014 | 2021 | 0 | -1.15  (-1.24, -0.92) |
| Prevalence | Central African Republic | Both | Glaucoma | 1990 | 2000 | 0.00039992 | 0.06  (0.04, 0.09) |
| Prevalence | Central African Republic | Both | Glaucoma | 2000 | 2005 | 0.00079984 | -0.18  (-0.25, -0.13) |
| Prevalence | Central African Republic | Both | Glaucoma | 2005 | 2010 | 0.00079984 | 0.86  (0.82, 0.91) |
| Prevalence | Central African Republic | Both | Glaucoma | 2010 | 2015 | 0.00079984 | -0.44  (-0.5, -0.39) |
| Prevalence | Central African Republic | Both | Glaucoma | 2015 | 2019 | 0.00879824 | 0.11  (0.04, 0.23) |
| Prevalence | Cambodia | Both | Glaucoma | 2015 | 2021 | 0 | -1.07  (-1.28, -0.91) |
| Prevalence | Central African Republic | Female | Glaucoma | 1990 | 2005 | 0 | 0.17  (0.16, 0.19) |
| Prevalence | Central African Republic | Female | Glaucoma | 2005 | 2010 | 0 | 1.01  (0.96, 1.05) |
| Prevalence | Central African Republic | Female | Glaucoma | 2010 | 2015 | 0 | -0.35  (-0.42, -0.29) |
| Prevalence | Central African Republic | Female | Glaucoma | 2015 | 2019 | 0 | 0.45  (0.37, 0.58) |
| Prevalence | Gambia | Male | Glaucoma | 2010 | 2021 | 0 | -1.14  (-1.36, -0.94) |
| Prevalence | Central African Republic | Male | Glaucoma | 1990 | 2000 | 0.291141772 | -0.02  (-0.05, 0.02) |
| Prevalence | Central African Republic | Male | Glaucoma | 2000 | 2005 | 0.028394321 | -0.57  (-0.64, -0.19) |
| Prevalence | Central African Republic | Male | Glaucoma | 2005 | 2010 | 0.061587682 | 0.72  (-0.54, 0.79) |
| Prevalence | Central African Republic | Male | Glaucoma | 2010 | 2015 | 0.061987602 | -0.47  (-0.59, 0.65) |
| Prevalence | United States Virgin Islands | Male | Glaucoma | 2019 | 2021 | 0 | -1.13  (-1.27, -0.95) |
| Prevalence | Chad | Both | Glaucoma | 1990 | 1994 | 0.00079984 | 0.36  (0.26, 0.48) |
| Prevalence | Chad | Both | Glaucoma | 1994 | 2000 | 0.013197361 | -0.11  (-0.26, -0.05) |
| Prevalence | Chad | Both | Glaucoma | 2000 | 2003 | 0.029994001 | 0.76  (0.05, 0.91) |
| Prevalence | Chad | Both | Glaucoma | 2003 | 2009 | 0.00319936 | 1  (0.91, 1.14) |
| Prevalence | Chad | Both | Glaucoma | 2009 | 2012 | 0.059988002 | 0.18  (-0.03, 0.36) |
| Prevalence | Panama | Both | Glaucoma | 2013 | 2021 | 0 | -1.07  (-1.12, -1.02) |
| Prevalence | Chad | Female | Glaucoma | 1990 | 2000 | 0 | -0.48  (-0.52, -0.45) |
| Prevalence | Chad | Female | Glaucoma | 2000 | 2003 | 0.00919816 | 1.35  (1.23, 1.51) |
| Prevalence | Chad | Female | Glaucoma | 2003 | 2010 | 0 | 1.77  (1.72, 1.87) |
| Prevalence | Chad | Female | Glaucoma | 2010 | 2015 | 0.00039992 | 0.23  (0.13, 0.33) |
| Prevalence | Maldives | Female | Glaucoma | 2000 | 2021 | 0 | -1.12  (-1.16, -1.08) |
| Prevalence | Chad | Male | Glaucoma | 1990 | 1994 | 0 | 0.8  (0.75, 0.85) |
| Prevalence | Chad | Male | Glaucoma | 1994 | 2000 | 0.029194161 | 0.06  (0.01, 0.09) |
| Prevalence | Chad | Male | Glaucoma | 2000 | 2008 | 0 | 0.3  (0.28, 0.34) |
| Prevalence | Chad | Male | Glaucoma | 2008 | 2011 | 0.169966007 | -0.06  (-0.13, 0.04) |
| Prevalence | Chad | Male | Glaucoma | 2011 | 2014 | 0.00119976 | -0.73  (-0.79, -0.64) |
| Prevalence | Tunisia | Female | Glaucoma | 2014 | 2021 | 0 | -1.12  (-1.18, -1.04) |
| Prevalence | Chile | Both | Glaucoma | 1990 | 1993 | 0 | -1.18  (-1.29, -1.1) |
| Prevalence | Chile | Both | Glaucoma | 1993 | 2005 | 0 | -1.05  (-1.06, -1.03) |
| Prevalence | Chile | Both | Glaucoma | 2005 | 2011 | 0 | -0.47  (-0.5, -0.44) |
| Prevalence | Chile | Both | Glaucoma | 2011 | 2015 | 0 | -0.62  (-0.68, -0.57) |
| Prevalence | Chile | Both | Glaucoma | 2015 | 2018 | 0 | -0.96  (-1.01, -0.88) |
| Prevalence | Comoros | Both | Glaucoma | 2015 | 2021 | 0 | -1.07  (-1.11, -1.03) |
| Prevalence | Chile | Female | Glaucoma | 1990 | 1994 | 0 | -1.11  (-1.29, -1.03) |
| Prevalence | Chile | Female | Glaucoma | 1994 | 2000 | 0 | -0.91  (-0.95, -0.79) |
| Prevalence | Chile | Female | Glaucoma | 2000 | 2005 | 0 | -1.13  (-1.21, -1.08) |
| Prevalence | Chile | Female | Glaucoma | 2005 | 2015 | 0 | -0.55  (-0.57, -0.53) |
| Prevalence | Chile | Female | Glaucoma | 2015 | 2019 | 0 | -1.01  (-1.09, -0.91) |
| Prevalence | Cuba | Male | Glaucoma | 2019 | 2021 | 0 | -1.11  (-1.25, -0.91) |
| Prevalence | Chile | Male | Glaucoma | 1990 | 1997 | 0 | -1.22  (-1.38, -1.16) |
| Prevalence | Chile | Male | Glaucoma | 1997 | 2004 | 0 | -1.09  (-1.13, -0.37) |
| Prevalence | Chile | Male | Glaucoma | 2004 | 2011 | 0.00039992 | -0.4  (-0.6, -0.31) |
| Prevalence | Tunisia | Male | Glaucoma | 2016 | 2021 | 0 | -1.1  (-1.18, -1) |
| Prevalence | China | Both | Glaucoma | 1990 | 1994 | 0 | -7.79  (-10.36, -6.37) |
| Prevalence | China | Both | Glaucoma | 1994 | 2000 | 0 | 3.54  (2.57, 4.99) |
| Prevalence | China | Both | Glaucoma | 2000 | 2010 | 0 | -1.86  (-2.78, -1.39) |
| Prevalence | China | Both | Glaucoma | 2010 | 2019 | 0.021995601 | 0.63  (0.11, 1.62) |
| Prevalence | Nauru | Both | Glaucoma | 2015 | 2021 | 0.0059988 | -1.03  (-1.24, -0.57) |
| Prevalence | China | Female | Glaucoma | 1990 | 1995 | 0 | -5.06  (-6.84, -3.97) |
| Prevalence | China | Female | Glaucoma | 1995 | 2000 | 0 | 4.81  (3.5, 7.03) |
| Prevalence | China | Female | Glaucoma | 2000 | 2011 | 0 | -1.73  (-2.36, -1.31) |
| Prevalence | China | Female | Glaucoma | 2011 | 2019 | 0.00479904 | 1.04  (0.45, 2.22) |
| Prevalence | Turkey | Female | Glaucoma | 2010 | 2021 | 0 | -1.1  (-1.17, -1.04) |
| Prevalence | China | Male | Glaucoma | 1990 | 1994 | 0 | -8.89  (-10.52, -7.49) |
| Prevalence | China | Male | Glaucoma | 1994 | 2000 | 0 | 3.22  (2.25, 4.54) |
| Prevalence | China | Male | Glaucoma | 2000 | 2009 | 0 | -2.18  (-3.39, -1.63) |
| Prevalence | China | Male | Glaucoma | 2009 | 2019 | 0.147970406 | 0.31  (-0.13, 1.13) |
| Prevalence | Rwanda | Male | Glaucoma | 2016 | 2021 | 0 | -1.09  (-1.21, -1) |
| Prevalence | Colombia | Both | Glaucoma | 1990 | 1995 | 0 | -1.21  (-1.25, -1.15) |
| Prevalence | Colombia | Both | Glaucoma | 1995 | 2000 | 0 | -1.41  (-1.46, -1.38) |
| Prevalence | Colombia | Both | Glaucoma | 2000 | 2005 | 0 | -0.58  (-0.62, -0.55) |
| Prevalence | Colombia | Both | Glaucoma | 2005 | 2011 | 0 | -1.02  (-1.08, -0.99) |
| Prevalence | Colombia | Both | Glaucoma | 2011 | 2019 | 0 | -0.92  (-0.95, -0.89) |
| Prevalence | Morocco | Both | Glaucoma | 2018 | 2021 | 0 | -1.03  (-1.21, -0.81) |
| Prevalence | Colombia | Female | Glaucoma | 1990 | 1994 | 0 | -1.01  (-1.08, -0.9) |
| Prevalence | Colombia | Female | Glaucoma | 1994 | 2000 | 0 | -1.25  (-1.33, -1.21) |
| Prevalence | Colombia | Female | Glaucoma | 2000 | 2005 | 0 | -0.67  (-0.75, -0.59) |
| Prevalence | Colombia | Female | Glaucoma | 2005 | 2016 | 0 | -0.86  (-0.88, -0.78) |
| Prevalence | Colombia | Female | Glaucoma | 2016 | 2019 | 0 | -1  (-1.08, -0.89) |
| Prevalence | Micronesia (Federated States of) | Male | Glaucoma | 2015 | 2021 | 0 | -1.08  (-1.11, -1.05) |
| Prevalence | Colombia | Male | Glaucoma | 1990 | 1994 | 0 | -1.27  (-1.35, -1.13) |
| Prevalence | Colombia | Male | Glaucoma | 1994 | 2000 | 0 | -1.45  (-1.5, -1.41) |
| Prevalence | Colombia | Male | Glaucoma | 2000 | 2005 | 0 | -0.45  (-0.53, -0.41) |
| Prevalence | Colombia | Male | Glaucoma | 2005 | 2014 | 0 | -1.05  (-1.08, -1.02) |
| Prevalence | Algeria | Male | Glaucoma | 2018 | 2021 | 0 | -1.07  (-1.13, -1) |
| Prevalence | Comoros | Both | Glaucoma | 1990 | 1994 | 0 | -0.78  (-0.92, -0.7) |
| Prevalence | Comoros | Both | Glaucoma | 1994 | 2001 | 0 | -0.56  (-0.59, -0.51) |
| Prevalence | Comoros | Both | Glaucoma | 2001 | 2009 | 0 | -1.2  (-1.23, -1.18) |
| Prevalence | Comoros | Both | Glaucoma | 2009 | 2015 | 0 | -0.51  (-0.54, -0.48) |
| Prevalence | Angola | Both | Glaucoma | 2015 | 2021 | 0 | -1.02  (-1.04, -1) |
| Prevalence | Comoros | Female | Glaucoma | 1990 | 1994 | 0 | -0.9  (-1.02, -0.81) |
| Prevalence | Comoros | Female | Glaucoma | 1994 | 2001 | 0 | -0.49  (-0.53, -0.45) |
| Prevalence | Comoros | Female | Glaucoma | 2001 | 2009 | 0 | -1.12  (-1.14, -1.09) |
| Prevalence | Comoros | Female | Glaucoma | 2009 | 2015 | 0 | -0.41  (-0.45, -0.38) |
| Prevalence | Eswatini | Female | Glaucoma | 2015 | 2021 | 0 | -1.07  (-1.11, -0.98) |
| Prevalence | Comoros | Male | Glaucoma | 1990 | 2001 | 0 | -0.61  (-0.63, -0.58) |
| Prevalence | Comoros | Male | Glaucoma | 2001 | 2009 | 0 | -1.23  (-1.27, -1.19) |
| Prevalence | Comoros | Male | Glaucoma | 2009 | 2015 | 0 | -0.55  (-0.61, -0.47) |
| Prevalence | Lebanon | Male | Glaucoma | 2018 | 2021 | 0 | -1.07  (-1.27, -0.76) |
| Prevalence | Congo | Both | Glaucoma | 1990 | 2011 | 0 | -0.48  (-0.5, -0.46) |
| Prevalence | Congo | Both | Glaucoma | 2011 | 2014 | 0 | -0.95  (-1.07, -0.66) |
| Prevalence | Philippines | Both | Glaucoma | 2015 | 2021 | 0 | -1.02  (-1.05, -0.99) |
| Prevalence | Congo | Female | Glaucoma | 1990 | 2010 | 0 | -0.51  (-0.53, -0.5) |
| Prevalence | Congo | Female | Glaucoma | 2010 | 2015 | 0 | -1.24  (-1.33, -1.11) |
| Prevalence | Papua New Guinea | Male | Glaucoma | 2015 | 2021 | 0 | -1.06  (-1.08, -1.03) |
| Prevalence | Congo | Male | Glaucoma | 1990 | 2000 | 0.026794641 | -0.39  (-0.49, -0.18) |
| Prevalence | Palau | Male | Glaucoma | 2015 | 2021 | 0 | -1.05  (-1.09, -1.03) |
| Prevalence | Cook Islands | Both | Glaucoma | 1990 | 1994 | 0 | -2.95  (-3.66, -2.58) |
| Prevalence | Cook Islands | Both | Glaucoma | 1994 | 2015 | 0 | -1.26  (-1.31, -1.22) |
| Prevalence | Myanmar | Both | Glaucoma | 2014 | 2021 | 0 | -1.01  (-1.13, -0.89) |
| Prevalence | Cook Islands | Female | Glaucoma | 1990 | 1995 | 0 | -2.47  (-3.72, -1.91) |
| Prevalence | Cook Islands | Female | Glaucoma | 1995 | 2010 | 0.048390322 | -0.89  (-0.98, -0.03) |
| Prevalence | Cook Islands | Female | Glaucoma | 2010 | 2014 | 0.00279944 | -1.86  (-2.66, -1.14) |
| Prevalence | Comoros | Male | Glaucoma | 2015 | 2021 | 0 | -1.05  (-1.13, -0.98) |
| Prevalence | Cook Islands | Male | Glaucoma | 1990 | 1993 | 0 | -3.12  (-3.29, -2.98) |
| Prevalence | Cook Islands | Male | Glaucoma | 1993 | 1996 | 0 | -2.16  (-2.31, -1.94) |
| Prevalence | Cook Islands | Male | Glaucoma | 1996 | 2005 | 0 | -1.34  (-1.37, -1.24) |
| Prevalence | Cook Islands | Male | Glaucoma | 2005 | 2010 | 0 | -1.52  (-1.68, -1.43) |
| Prevalence | Cook Islands | Male | Glaucoma | 2010 | 2015 | 0 | -0.97  (-1.06, -0.83) |
| Prevalence | Sao Tome and Principe | Male | Glaucoma | 2016 | 2021 | 0 | -1.05  (-1.09, -0.97) |
| Prevalence | Costa Rica | Both | Glaucoma | 1990 | 1995 | 0 | -0.71  (-0.74, -0.68) |
| Prevalence | Costa Rica | Both | Glaucoma | 1995 | 2005 | 0 | -1.1  (-1.12, -1) |
| Prevalence | Costa Rica | Both | Glaucoma | 2005 | 2010 | 0 | -0.6  (-1.1, -0.57) |
| Prevalence | Costa Rica | Both | Glaucoma | 2010 | 2015 | 0 | -1  (-1.05, -0.6) |
| Prevalence | Costa Rica | Both | Glaucoma | 2015 | 2018 | 0 | -0.86  (-0.98, -0.73) |
| Prevalence | Indonesia | Both | Glaucoma | 2015 | 2021 | 0 | -1  (-1.12, -0.89) |
| Prevalence | Costa Rica | Female | Glaucoma | 1990 | 1995 | 0 | -0.58  (-0.65, -0.49) |
| Prevalence | Costa Rica | Female | Glaucoma | 1995 | 2000 | 0 | -1.1  (-1.18, -0.98) |
| Prevalence | Costa Rica | Female | Glaucoma | 2000 | 2005 | 0 | -1.43  (-1.54, -1.36) |
| Prevalence | Costa Rica | Female | Glaucoma | 2005 | 2010 | 0 | -0.62  (-0.7, -0.52) |
| Prevalence | Costa Rica | Female | Glaucoma | 2010 | 2018 | 0 | -1.06  (-1.1, -1.02) |
| Prevalence | Comoros | Female | Glaucoma | 2015 | 2021 | 0 | -1.04  (-1.08, -1) |
| Prevalence | Costa Rica | Male | Glaucoma | 1990 | 1995 | 0 | -0.77  (-0.8, -0.75) |
| Prevalence | Costa Rica | Male | Glaucoma | 1995 | 2000 | 0 | -1.08  (-1.12, -1.06) |
| Prevalence | Costa Rica | Male | Glaucoma | 2000 | 2005 | 0 | -0.77  (-0.81, -0.74) |
| Prevalence | Costa Rica | Male | Glaucoma | 2005 | 2010 | 0 | -0.55  (-0.58, -0.51) |
| Prevalence | Costa Rica | Male | Glaucoma | 2010 | 2015 | 0 | -0.87  (-0.92, -0.84) |
| Prevalence | Belize | Male | Glaucoma | 2019 | 2021 | 0 | -1.04  (-1.23, -0.73) |
| Prevalence | Coted'Ivoire | Both | Glaucoma | 1990 | 1992 | 0 | 20.37  (18.93, 21.96) |
| Prevalence | Coted'Ivoire | Both | Glaucoma | 1992 | 1995 | 0 | 7.28  (6.64, 7.96) |
| Prevalence | Coted'Ivoire | Both | Glaucoma | 1995 | 2010 | 0 | 0.41  (0.31, 0.52) |
| Prevalence | Saudi Arabia | Both | Glaucoma | 2015 | 2021 | 0.00239952 | -0.99  (-1.21, -0.56) |
| Prevalence | Coted'Ivoire | Female | Glaucoma | 1990 | 1992 | 0 | 20.55  (19.17, 22.08) |
| Prevalence | Coted'Ivoire | Female | Glaucoma | 1992 | 1995 | 0 | 7.58  (6.96, 8.26) |
| Prevalence | Coted'Ivoire | Female | Glaucoma | 1995 | 2010 | 0 | 0.6  (0.5, 0.71) |
| Prevalence | Suriname | Male | Glaucoma | 2019 | 2021 | 0 | -1.03  (-1.35, -0.62) |
| Prevalence | Coted'Ivoire | Male | Glaucoma | 1990 | 1992 | 0 | 20.2  (18.77, 21.77) |
| Prevalence | Coted'Ivoire | Male | Glaucoma | 1992 | 1995 | 0 | 7.29  (6.66, 7.96) |
| Prevalence | Coted'Ivoire | Male | Glaucoma | 1995 | 2010 | 0 | 0.29  (0.19, 0.41) |
| Prevalence | Malta | Female | Glaucoma | 2015 | 2021 | 0 | -1.01  (-1.34, -0.89) |
| Prevalence | Croatia | Both | Glaucoma | 1990 | 1995 | 0 | -0.43  (-0.46, -0.38) |
| Prevalence | Croatia | Both | Glaucoma | 1995 | 2000 | 0 | -0.59  (-0.63, -0.56) |
| Prevalence | Croatia | Both | Glaucoma | 2000 | 2006 | 0 | -1.01  (-1.03, -0.99) |
| Prevalence | Croatia | Both | Glaucoma | 2006 | 2013 | 0 | -0.72  (-0.75, -0.7) |
| Prevalence | Croatia | Both | Glaucoma | 2013 | 2018 | 0 | -0.59  (-0.63, -0.53) |
| Prevalence | Mozambique | Both | Glaucoma | 2014 | 2021 | 0 | -0.97  (-1.09, -0.89) |
| Prevalence | Croatia | Female | Glaucoma | 1990 | 1995 | 0 | -0.59  (-0.65, -0.54) |
| Prevalence | Croatia | Female | Glaucoma | 1995 | 2000 | 0 | -0.37  (-0.41, -0.31) |
| Prevalence | Croatia | Female | Glaucoma | 2000 | 2005 | 0 | -1.06  (-1.12, -1.01) |
| Prevalence | Croatia | Female | Glaucoma | 2005 | 2019 | 0 | -0.79  (-0.8, -0.78) |
| Prevalence | Vanuatu | Male | Glaucoma | 2014 | 2021 | 0 | -1.01  (-1.05, -0.97) |
| Prevalence | Croatia | Male | Glaucoma | 1990 | 1994 | 0.017996401 | -0.15  (-0.23, -0.04) |
| Prevalence | Croatia | Male | Glaucoma | 1994 | 1997 | 0 | -0.56  (-0.81, -0.44) |
| Prevalence | Croatia | Male | Glaucoma | 1997 | 2008 | 0 | -1.01  (-1.04, -0.99) |
| Prevalence | Croatia | Male | Glaucoma | 2008 | 2014 | 0 | -0.74  (-0.81, -0.66) |
| Prevalence | Malawi | Male | Glaucoma | 2010 | 2021 | 0 | -1  (-1.09, -0.92) |
| Prevalence | Cuba | Both | Glaucoma | 1990 | 1992 | 0 | -1.43  (-1.51, -1.32) |
| Prevalence | Cuba | Both | Glaucoma | 1992 | 1995 | 0 | -1.17  (-1.21, -0.82) |
| Prevalence | Cuba | Both | Glaucoma | 1995 | 2000 | 0 | -0.74  (-0.78, -0.7) |
| Prevalence | Cuba | Both | Glaucoma | 2000 | 2005 | 0 | -0.97  (-1, -0.93) |
| Prevalence | Cuba | Both | Glaucoma | 2005 | 2010 | 0 | -0.6  (-0.64, -0.57) |
| Prevalence | Algeria | Both | Glaucoma | 2018 | 2021 | 0 | -0.97  (-1.01, -0.93) |
| Prevalence | Cuba | Female | Glaucoma | 1990 | 1994 | 0 | -1.28  (-1.46, -1.2) |
| Prevalence | Cuba | Female | Glaucoma | 1994 | 2000 | 0 | -0.69  (-0.73, -0.56) |
| Prevalence | Cuba | Female | Glaucoma | 2000 | 2011 | 0 | -0.77  (-0.81, -0.75) |
| Prevalence | Cuba | Female | Glaucoma | 2011 | 2019 | 0 | -0.88  (-1.02, -0.85) |
| Prevalence | Mongolia | Female | Glaucoma | 2011 | 2021 | 0 | -1  (-1.06, -0.93) |
| Prevalence | Cuba | Male | Glaucoma | 1990 | 1994 | 0 | -1.24  (-1.38, -1.16) |
| Prevalence | Cuba | Male | Glaucoma | 1994 | 2000 | 0 | -0.77  (-0.82, -0.69) |
| Prevalence | Cuba | Male | Glaucoma | 2000 | 2005 | 0 | -1.06  (-1.13, -0.98) |
| Prevalence | Cuba | Male | Glaucoma | 2005 | 2010 | 0 | -0.46  (-0.85, -0.39) |
| Prevalence | Cuba | Male | Glaucoma | 2010 | 2019 | 0 | -0.89  (-0.9, -0.46) |
| Prevalence | Grenada | Male | Glaucoma | 2012 | 2021 | 0 | -0.99  (-1.04, -0.95) |
| Prevalence | Cyprus | Both | Glaucoma | 1990 | 1995 | 0 | -2.02  (-2.17, -1.93) |
| Prevalence | Cyprus | Both | Glaucoma | 1995 | 2000 | 0 | -1.58  (-1.66, -1.39) |
| Prevalence | Cyprus | Both | Glaucoma | 2000 | 2005 | 0 | -1.95  (-2.12, -1.87) |
| Prevalence | Cyprus | Both | Glaucoma | 2005 | 2010 | 0 | -1.39  (-1.46, -1.28) |
| Prevalence | Cyprus | Both | Glaucoma | 2010 | 2019 | 0 | -0.59  (-0.65, -0.56) |
| Prevalence | Ethiopia | Both | Glaucoma | 2019 | 2021 | 0.061987602 | -0.92  (-1.77, 0.15) |
| Prevalence | Cyprus | Female | Glaucoma | 1990 | 1994 | 0 | -2.2  (-2.44, -2.08) |
| Prevalence | Cyprus | Female | Glaucoma | 1994 | 2001 | 0 | -1.65  (-1.7, -1.46) |
| Prevalence | Cyprus | Female | Glaucoma | 2001 | 2004 | 0 | -1.94  (-2.05, -1.74) |
| Prevalence | Cyprus | Female | Glaucoma | 2004 | 2009 | 0 | -1.5  (-1.57, -1) |
| Prevalence | Cyprus | Female | Glaucoma | 2009 | 2019 | 0 | -0.66  (-0.75, -0.63) |
| Prevalence | Hungary | Male | Glaucoma | 2019 | 2021 | 0 | -0.98  (-1.18, -0.75) |
| Prevalence | Cyprus | Male | Glaucoma | 1990 | 1995 | 0 | -2.02  (-2.16, -1.93) |
| Prevalence | Cyprus | Male | Glaucoma | 1995 | 2000 | 0 | -1.59  (-1.68, -1.41) |
| Prevalence | Cyprus | Male | Glaucoma | 2000 | 2005 | 0 | -2.02  (-2.17, -1.94) |
| Prevalence | Cyprus | Male | Glaucoma | 2005 | 2010 | 0 | -1.38  (-1.46, -1.28) |
| Prevalence | Cyprus | Male | Glaucoma | 2010 | 2019 | 0 | -0.52  (-0.61, -0.5) |
| Prevalence | Bermuda | Male | Glaucoma | 2019 | 2021 | 0 | -0.98  (-1.1, -0.84) |
| Prevalence | Czechia | Both | Glaucoma | 1990 | 1994 | 0 | -2.01  (-2.1, -1.89) |
| Prevalence | Czechia | Both | Glaucoma | 1994 | 2008 | 0 | -0.77  (-0.82, -0.74) |
| Prevalence | Czechia | Both | Glaucoma | 2008 | 2017 | 0 | -0.62  (-0.68, -0.5) |
| Prevalence | Ghana | Both | Glaucoma | 2014 | 2021 | 0 | -0.91  (-1, -0.77) |
| Prevalence | Czechia | Female | Glaucoma | 1990 | 1992 | 0 | -2.54  (-2.72, -2.36) |
| Prevalence | Czechia | Female | Glaucoma | 1992 | 1995 | 0 | -1.74  (-1.85, -1.63) |
| Prevalence | Czechia | Female | Glaucoma | 1995 | 2000 | 0 | -0.53  (-0.6, -0.42) |
| Prevalence | Czechia | Female | Glaucoma | 2000 | 2010 | 0 | -0.79  (-0.82, -0.71) |
| Prevalence | Czechia | Female | Glaucoma | 2010 | 2018 | 0 | -0.87  (-0.97, -0.84) |
| Prevalence | Democratic Republic of the Congo | Male | Glaucoma | 2015 | 2021 | 0 | -0.96  (-1.08, -0.8) |
| Prevalence | Czechia | Male | Glaucoma | 1990 | 1993 | 0 | -1.79  (-1.85, -1.73) |
| Prevalence | Czechia | Male | Glaucoma | 1993 | 1996 | 0 | -1.27  (-1.32, -1.2) |
| Prevalence | Czechia | Male | Glaucoma | 1996 | 2004 | 0 | -0.95  (-0.98, -0.93) |
| Prevalence | Czechia | Male | Glaucoma | 2004 | 2008 | 0 | -0.75  (-0.83, -0.67) |
| Prevalence | Czechia | Male | Glaucoma | 2008 | 2014 | 0 | -0.56  (-0.59, -0.52) |
| Prevalence | Iraq | Female | Glaucoma | 2019 | 2021 | 0 | -0.96  (-1.09, -0.86) |
| Prevalence | Democratic People's Republic of Korea | Both | Glaucoma | 1990 | 1995 | 0 | -0.62  (-0.87, -0.5) |
| Prevalence | Democratic People's Republic of Korea | Both | Glaucoma | 1995 | 2000 | 0.148770246 | -0.1  (-0.41, 0.1) |
| Prevalence | Democratic People's Republic of Korea | Both | Glaucoma | 2000 | 2010 | 0.020795841 | -0.82  (-0.91, -0.09) |
| Prevalence | Democratic People's Republic of Korea | Both | Glaucoma | 2010 | 2018 | 0.013197361 | -0.32  (-0.9, -0.1) |
| Prevalence | Cuba | Both | Glaucoma | 2010 | 2021 | 0 | -0.89  (-0.9, -0.88) |
| Prevalence | Democratic People's Republic of Korea | Female | Glaucoma | 1990 | 1995 | 0.00079984 | -0.67  (-1.1, -0.52) |
| Prevalence | Democratic People's Republic of Korea | Female | Glaucoma | 1995 | 2000 | 0.327134573 | -0.09  (-0.26, 0.25) |
| Prevalence | Democratic People's Republic of Korea | Female | Glaucoma | 2000 | 2010 | 0.00079984 | -0.91  (-1.01, -0.85) |
| Prevalence | Democratic People's Republic of Korea | Female | Glaucoma | 2010 | 2018 | 0.059588082 | -0.18  (-0.26, 0.03) |
| Prevalence | Thailand | Female | Glaucoma | 2014 | 2021 | 0 | -0.95  (-1.16, -0.86) |
| Prevalence | Democratic People's Republic of Korea | Male | Glaucoma | 1990 | 1994 | 0 | -0.56  (-0.65, -0.5) |
| Prevalence | Democratic People's Republic of Korea | Male | Glaucoma | 1994 | 2004 | 0 | -0.25  (-0.26, -0.23) |
| Prevalence | Democratic People's Republic of Korea | Male | Glaucoma | 2004 | 2014 | 0 | -0.68  (-0.69, -0.66) |
| Prevalence | Democratic People's Republic of Korea | Male | Glaucoma | 2014 | 2019 | 0 | -0.37  (-0.42, -0.3) |
| Prevalence | Nicaragua | Male | Glaucoma | 2016 | 2021 | 0 | -0.95  (-0.97, -0.92) |
| Prevalence | Democratic Republic of the Congo | Both | Glaucoma | 1990 | 1994 | 0.024395121 | 0.35  (0.1, 0.49) |
| Prevalence | Democratic Republic of the Congo | Both | Glaucoma | 1994 | 2001 | 0 | 0.8  (0.74, 0.95) |
| Prevalence | Democratic Republic of the Congo | Both | Glaucoma | 2001 | 2010 | 0 | 0.45  (0.4, 0.49) |
| Prevalence | Democratic Republic of the Congo | Both | Glaucoma | 2010 | 2016 | 0 | -1.08  (-1.17, -1.02) |
| Prevalence | Lebanon | Both | Glaucoma | 2018 | 2021 | 0 | -0.88  (-1.07, -0.66) |
| Prevalence | Democratic Republic of the Congo | Female | Glaucoma | 1990 | 1995 | 0 | 0.87  (0.73, 0.94) |
| Prevalence | Democratic Republic of the Congo | Female | Glaucoma | 1995 | 2003 | 0 | 1.11  (1.07, 1.17) |
| Prevalence | Democratic Republic of the Congo | Female | Glaucoma | 2003 | 2008 | 0 | 0.6  (0.56, 0.69) |
| Prevalence | Democratic Republic of the Congo | Female | Glaucoma | 2008 | 2011 | 0.016396721 | 0.15  (0.07, 0.28) |
| Prevalence | Democratic Republic of the Congo | Female | Glaucoma | 2011 | 2015 | 0 | -0.96  (-1.08, -0.88) |
| Prevalence | Mauritania | Male | Glaucoma | 2015 | 2021 | 0 | -0.95  (-0.97, -0.91) |
| Prevalence | Democratic Republic of the Congo | Male | Glaucoma | 1990 | 1995 | 0.105178964 | 0.22  (-0.08, 0.37) |
| Prevalence | Democratic Republic of the Congo | Male | Glaucoma | 1995 | 1999 | 0 | 0.92  (0.69, 1.21) |
| Prevalence | Democratic Republic of the Congo | Male | Glaucoma | 1999 | 2011 | 0.00119976 | 0.18  (0.13, 0.23) |
| Prevalence | Democratic Republic of the Congo | Male | Glaucoma | 2011 | 2015 | 0 | -1.87  (-2.12, -1.7) |
| Prevalence | Sri Lanka | Female | Glaucoma | 2018 | 2021 | 0.00159968 | -0.94  (-1.23, -0.38) |
| Prevalence | Denmark | Both | Glaucoma | 1990 | 1995 | 0 | -0.86  (-0.96, -0.77) |
| Prevalence | Denmark | Both | Glaucoma | 1995 | 1999 | 0 | -1.55  (-1.64, -1.41) |
| Prevalence | Denmark | Both | Glaucoma | 1999 | 2006 | 0.00159968 | -0.24  (-0.38, -0.18) |
| Prevalence | Denmark | Both | Glaucoma | 2006 | 2010 | 0.00119976 | -0.95  (-1.11, -0.23) |
| Prevalence | Denmark | Both | Glaucoma | 2010 | 2016 | 0 | -0.49  (-0.92, -0.29) |
| Prevalence | Bolivia (Plurinational State of) | Both | Glaucoma | 2018 | 2021 | 0 | -0.87  (-0.98, -0.73) |
| Prevalence | Denmark | Female | Glaucoma | 1990 | 1995 | 0 | -0.82  (-0.9, -0.73) |
| Prevalence | Denmark | Female | Glaucoma | 1995 | 1999 | 0 | -1.3  (-1.41, -1.2) |
| Prevalence | Denmark | Female | Glaucoma | 1999 | 2006 | 0.00079984 | -0.2  (-0.25, -0.15) |
| Prevalence | Denmark | Female | Glaucoma | 2006 | 2010 | 0.00039992 | -1.11  (-1.28, -0.96) |
| Prevalence | Denmark | Female | Glaucoma | 2010 | 2015 | 0 | -0.44  (-0.53, -0.23) |
| Prevalence | Northern Mariana Islands | Male | Glaucoma | 2019 | 2021 | 0 | -0.94  (-1.12, -0.8) |
| Prevalence | Denmark | Male | Glaucoma | 1990 | 1995 | 0 | -0.9  (-0.99, -0.8) |
| Prevalence | Denmark | Male | Glaucoma | 1995 | 1999 | 0 | -1.83  (-1.93, -1.7) |
| Prevalence | Denmark | Male | Glaucoma | 1999 | 2006 | 0.00079984 | -0.41  (-0.48, -0.34) |
| Prevalence | Denmark | Male | Glaucoma | 2006 | 2012 | 0 | -0.81  (-1.01, -0.73) |
| Prevalence | Ethiopia | Female | Glaucoma | 2019 | 2021 | 0.091581684 | -0.93  (-2.12, 0.56) |
| Prevalence | Djibouti | Both | Glaucoma | 1990 | 2001 | 0 | -0.22  (-0.24, -0.2) |
| Prevalence | Djibouti | Both | Glaucoma | 2001 | 2009 | 0 | -0.85  (-0.87, -0.82) |
| Prevalence | Djibouti | Both | Glaucoma | 2009 | 2016 | 0 | -1.06  (-1.1, -1.02) |
| Prevalence | Madagascar | Both | Glaucoma | 2014 | 2021 | 0 | -0.85  (-1.03, -0.78) |
| Prevalence | Djibouti | Female | Glaucoma | 1990 | 1993 | 0 | -0.36  (-0.46, -0.28) |
| Prevalence | Djibouti | Female | Glaucoma | 1993 | 2001 | 0 | -0.23  (-0.24, -0.19) |
| Prevalence | Djibouti | Female | Glaucoma | 2001 | 2006 | 0 | -0.8  (-0.84, -0.78) |
| Prevalence | Djibouti | Female | Glaucoma | 2006 | 2016 | 0 | -1.02  (-1.04, -1.01) |
| Prevalence | Samoa | Male | Glaucoma | 2019 | 2021 | 0 | -0.92  (-1.09, -0.81) |
| Prevalence | Djibouti | Male | Glaucoma | 1990 | 1998 | 0.019996001 | -0.2  (-0.25, -0.13) |
| Prevalence | Djibouti | Male | Glaucoma | 1998 | 2001 | 0.00319936 | -0.43  (-0.86, -0.22) |
| Prevalence | Djibouti | Male | Glaucoma | 2001 | 2010 | 0 | -0.87  (-1.13, -0.76) |
| Prevalence | Djibouti | Male | Glaucoma | 2010 | 2015 | 0 | -1.18  (-1.3, -0.91) |
| Prevalence | Dominica | Male | Glaucoma | 2019 | 2021 | 0 | -0.91  (-1.02, -0.69) |
| Prevalence | Dominica | Both | Glaucoma | 1990 | 1995 | 0 | -1.3  (-1.33, -1.25) |
| Prevalence | Dominica | Both | Glaucoma | 1995 | 2000 | 0 | -1.42  (-1.46, -1.4) |
| Prevalence | Dominica | Both | Glaucoma | 2000 | 2010 | 0 | -0.88  (-0.89, -0.87) |
| Prevalence | Dominica | Both | Glaucoma | 2010 | 2015 | 0 | -0.56  (-0.58, -0.52) |
| Prevalence | Dominica | Both | Glaucoma | 2015 | 2018 | 0 | -0.68  (-0.71, -0.62) |
| Prevalence | Turkmenistan | Both | Glaucoma | 2014 | 2021 | 0 | -0.84  (-0.89, -0.75) |
| Prevalence | Dominica | Female | Glaucoma | 1990 | 1995 | 0 | -1.16  (-1.21, -1.09) |
| Prevalence | Dominica | Female | Glaucoma | 1995 | 2000 | 0 | -1.43  (-1.5, -1.38) |
| Prevalence | Dominica | Female | Glaucoma | 2000 | 2006 | 0 | -0.86  (-0.89, -0.79) |
| Prevalence | Dominica | Female | Glaucoma | 2006 | 2009 | 0 | -1.06  (-1.12, -0.95) |
| Prevalence | Dominica | Female | Glaucoma | 2009 | 2019 | 0 | -0.71  (-0.73, -0.69) |
| Prevalence | Mauritania | Female | Glaucoma | 2010 | 2021 | 0 | -0.91  (-0.92, -0.9) |
| Prevalence | Dominica | Male | Glaucoma | 1990 | 2000 | 0 | -1.34  (-1.37, -1.32) |
| Prevalence | Dominica | Male | Glaucoma | 2000 | 2008 | 0 | -0.92  (-1.04, -0.89) |
| Prevalence | Dominica | Male | Glaucoma | 2008 | 2019 | 0 | -0.67  (-0.91, -0.62) |
| Prevalence | Haiti | Male | Glaucoma | 2001 | 2021 | 0 | -0.91  (-0.93, -0.88) |
| Prevalence | Dominican Republic | Both | Glaucoma | 1990 | 1995 | 0 | -1.09  (-1.17, -1) |
| Prevalence | Dominican Republic | Both | Glaucoma | 1995 | 2000 | 0 | -1.72  (-1.84, -1.63) |
| Prevalence | Dominican Republic | Both | Glaucoma | 2000 | 2006 | 0 | -1.13  (-1.21, -1) |
| Prevalence | Dominican Republic | Both | Glaucoma | 2006 | 2009 | 0 | -1.36  (-1.45, -0.59) |
| Prevalence | Dominican Republic | Both | Glaucoma | 2009 | 2015 | 0 | -0.42  (-0.5, -0.35) |
| Prevalence | Sri Lanka | Both | Glaucoma | 2018 | 2021 | 0.00759848 | -0.83  (-1.11, -0.37) |
| Prevalence | Dominican Republic | Female | Glaucoma | 1990 | 1995 | 0 | -1.02  (-1.15, -0.83) |
| Prevalence | Dominican Republic | Female | Glaucoma | 1995 | 1999 | 0 | -1.68  (-1.91, -1.48) |
| Prevalence | Dominican Republic | Female | Glaucoma | 1999 | 2010 | 0 | -1.17  (-1.21, -1.11) |
| Prevalence | Dominican Republic | Female | Glaucoma | 2010 | 2015 | 0.071985603 | -0.16  (-0.28, 0.04) |
| Prevalence | Dominican Republic | Female | Glaucoma | 2015 | 2018 | 0 | -1.65  (-1.83, -1.36) |
| Prevalence | Equatorial Guinea | Female | Glaucoma | 2019 | 2021 | 0 | -0.9  (-1.25, -0.4) |
| Prevalence | Dominican Republic | Male | Glaucoma | 1990 | 1995 | 0 | -1.16  (-1.21, -1.11) |
| Prevalence | Dominican Republic | Male | Glaucoma | 1995 | 2000 | 0 | -1.65  (-1.71, -1.59) |
| Prevalence | Dominican Republic | Male | Glaucoma | 2000 | 2009 | 0 | -1.2  (-1.23, -1.18) |
| Prevalence | Dominican Republic | Male | Glaucoma | 2009 | 2016 | 0 | -0.58  (-0.61, -0.54) |
| Prevalence | Dominican Republic | Male | Glaucoma | 2016 | 2019 | 0 | -0.84  (-0.9, -0.7) |
| Prevalence | Democratic People's Republic of Korea | Female | Glaucoma | 2018 | 2021 | 0 | -0.9  (-1.54, -0.58) |
| Prevalence | Ecuador | Both | Glaucoma | 1990 | 1995 | 0 | -1.24  (-1.35, -1.05) |
| Prevalence | Ecuador | Both | Glaucoma | 1995 | 2000 | 0 | -1.7  (-1.91, -1.59) |
| Prevalence | Ecuador | Both | Glaucoma | 2000 | 2008 | 0 | -0.73  (-0.78, -0.61) |
| Prevalence | Ecuador | Both | Glaucoma | 2008 | 2018 | 0 | -0.92  (-0.99, -0.88) |
| Prevalence | Sao Tome and Principe | Both | Glaucoma | 2019 | 2021 | 0 | -0.83  (-0.96, -0.76) |
| Prevalence | Ecuador | Female | Glaucoma | 1990 | 1995 | 0 | -1.08  (-1.22, -0.75) |
| Prevalence | Ecuador | Female | Glaucoma | 1995 | 1999 | 0 | -1.69  (-1.9, -1.45) |
| Prevalence | American Samoa | Male | Glaucoma | 2018 | 2021 | 0 | -0.9  (-0.95, -0.81) |
| Prevalence | Ecuador | Male | Glaucoma | 1990 | 1995 | 0 | -1.35  (-1.49, -1.08) |
| Prevalence | Ecuador | Male | Glaucoma | 1995 | 2000 | 0 | -1.85  (-2.26, -1.69) |
| Prevalence | Ecuador | Male | Glaucoma | 2000 | 2011 | 0 | -0.85  (-0.89, -0.74) |
| Prevalence | Ecuador | Male | Glaucoma | 2011 | 2018 | 0 | -1.14  (-1.34, -1.05) |
| Prevalence | Jordan | Male | Glaucoma | 2009 | 2021 | 0 | -0.9  (-0.98, -0.78) |
| Prevalence | Egypt | Both | Glaucoma | 1990 | 1994 | 0 | 0.85  (0.67, 1.01) |
| Prevalence | Egypt | Both | Glaucoma | 1994 | 2000 | 0.00039992 | -0.36  (-0.46, -0.25) |
| Prevalence | Egypt | Both | Glaucoma | 2000 | 2005 | 0 | -1.53  (-1.69, -1.41) |
| Prevalence | Egypt | Both | Glaucoma | 2005 | 2009 | 0 | -0.7  (-0.97, -0.44) |
| Prevalence | Egypt | Both | Glaucoma | 2009 | 2015 | 0.429114177 | -0.04  (-0.13, 0.1) |
| Prevalence | Liberia | Both | Glaucoma | 2019 | 2021 | 0 | -0.82  (-1.03, -0.7) |
| Prevalence | Egypt | Female | Glaucoma | 1990 | 1994 | 0 | 1.7  (1.5, 1.9) |
| Prevalence | Egypt | Female | Glaucoma | 1994 | 2000 | 0.033193361 | -0.14  (-0.27, -0.02) |
| Prevalence | Egypt | Female | Glaucoma | 2000 | 2005 | 0 | -1.62  (-1.82, -1.46) |
| Prevalence | Egypt | Female | Glaucoma | 2005 | 2010 | 0.00079984 | -0.37  (-0.62, -0.18) |
| Prevalence | Egypt | Female | Glaucoma | 2010 | 2015 | 0.00119976 | 0.36  (0.2, 0.6) |
| Prevalence | Mozambique | Male | Glaucoma | 2006 | 2021 | 0 | -0.9  (-0.95, -0.84) |
| Prevalence | Egypt | Male | Glaucoma | 1990 | 1994 | 0.00359928 | 0.23  (0.07, 0.41) |
| Prevalence | Egypt | Male | Glaucoma | 1994 | 2000 | 0 | -0.52  (-0.61, -0.44) |
| Prevalence | Egypt | Male | Glaucoma | 2000 | 2005 | 0 | -1.55  (-1.69, -1.45) |
| Prevalence | Egypt | Male | Glaucoma | 2005 | 2009 | 0 | -0.88  (-1.11, -0.64) |
| Prevalence | Egypt | Male | Glaucoma | 2009 | 2015 | 0.0019996 | -0.28  (-0.35, -0.17) |
| Prevalence | Honduras | Female | Glaucoma | 2015 | 2021 | 0 | -0.89  (-1.04, -0.79) |
| Prevalence | El Salvador | Both | Glaucoma | 1990 | 1995 | 0 | -1.86  (-1.93, -1.75) |
| Prevalence | El Salvador | Both | Glaucoma | 1995 | 2000 | 0 | -2.1  (-2.21, -2.04) |
| Prevalence | El Salvador | Both | Glaucoma | 2000 | 2005 | 0 | -1.36  (-1.41, -1.3) |
| Prevalence | Namibia | Both | Glaucoma | 2019 | 2021 | 0 | -0.81  (-1.01, -0.68) |
| Prevalence | El Salvador | Female | Glaucoma | 1990 | 1996 | 0 | -1.66  (-1.7, -1.61) |
| Prevalence | El Salvador | Female | Glaucoma | 1996 | 1999 | 0 | -2.06  (-2.13, -1.91) |
| Prevalence | El Salvador | Female | Glaucoma | 1999 | 2002 | 0 | -1.58  (-1.71, -1.34) |
| Prevalence | El Salvador | Female | Glaucoma | 2002 | 2005 | 0 | -1.16  (-1.22, -0.74) |
| Prevalence | El Salvador | Female | Glaucoma | 2005 | 2019 | 0 | -0.73  (-0.75, -0.72) |
| Prevalence | Gabon | Male | Glaucoma | 1999 | 2021 | 0 | -0.88  (-1.27, -0.86) |
| Prevalence | El Salvador | Male | Glaucoma | 1990 | 1995 | 0 | -1.95  (-1.99, -1.9) |
| Prevalence | El Salvador | Male | Glaucoma | 1995 | 2000 | 0 | -2.1  (-2.14, -2.07) |
| Prevalence | El Salvador | Male | Glaucoma | 2000 | 2003 | 0 | -1.44  (-1.49, -1.39) |
| Prevalence | El Salvador | Male | Glaucoma | 2003 | 2006 | 0 | -1.07  (-1.12, -0.99) |
| Prevalence | El Salvador | Male | Glaucoma | 2006 | 2016 | 0 | -0.72  (-0.74, -0.71) |
| Prevalence | Sweden | Male | Glaucoma | 2010 | 2021 | 0.00519896 | -0.87  (-1.07, -0.69) |
| Prevalence | Equatorial Guinea | Both | Glaucoma | 1990 | 1993 | 0.0059988 | -0.54  (-0.73, -0.17) |
| Prevalence | Equatorial Guinea | Both | Glaucoma | 1993 | 1996 | 0 | -1.25  (-2.02, -1.09) |
| Prevalence | Equatorial Guinea | Both | Glaucoma | 1996 | 2005 | 0 | -2.67  (-2.71, -2.62) |
| Prevalence | Equatorial Guinea | Both | Glaucoma | 2005 | 2010 | 0 | -0.8  (-0.89, -0.7) |
| Prevalence | Equatorial Guinea | Both | Glaucoma | 2010 | 2015 | 0 | -1.46  (-1.62, -1.35) |
| Prevalence | Pakistan | Both | Glaucoma | 2010 | 2021 | 0 | -0.81  (-0.86, -0.76) |
| Prevalence | Equatorial Guinea | Female | Glaucoma | 1990 | 1996 | 0 | -0.73  (-0.82, -0.63) |
| Prevalence | Equatorial Guinea | Female | Glaucoma | 1996 | 2005 | 0 | -2.47  (-2.53, -1.48) |
| Prevalence | Equatorial Guinea | Female | Glaucoma | 2005 | 2010 | 0 | -0.67  (-2.49, -0.54) |
| Prevalence | Equatorial Guinea | Female | Glaucoma | 2010 | 2015 | 0 | -1.61  (-1.79, -0.65) |
| Prevalence | Equatorial Guinea | Female | Glaucoma | 2015 | 2019 | 0.050389922 | -0.24  (-1.61, 0) |
| Prevalence | Guam | Male | Glaucoma | 2016 | 2021 | 0 | -0.87  (-1.14, -0.69) |
| Prevalence | Equatorial Guinea | Male | Glaucoma | 1990 | 1993 | 0.043991202 | -0.5  (-0.88, -0.03) |
| Prevalence | Equatorial Guinea | Male | Glaucoma | 1993 | 1996 | 0 | -1.31  (-2.91, -1.1) |
| Prevalence | Equatorial Guinea | Male | Glaucoma | 1996 | 2005 | 0 | -2.88  (-2.94, -2.79) |
| Prevalence | Equatorial Guinea | Male | Glaucoma | 2005 | 2018 | 0 | -1.22  (-1.26, -1.19) |
| Prevalence | Ghana | Male | Glaucoma | 2015 | 2021 | 0 | -0.87  (-1.01, -0.64) |
| Prevalence | Eritrea | Both | Glaucoma | 1990 | 1994 | 0 | -0.67  (-0.86, -0.57) |
| Prevalence | Eritrea | Both | Glaucoma | 1994 | 2000 | 0 | -0.47  (-0.53, -0.34) |
| Prevalence | Eritrea | Both | Glaucoma | 2000 | 2005 | 0 | -0.81  (-0.94, -0.73) |
| Prevalence | Eritrea | Both | Glaucoma | 2005 | 2010 | 0 | -0.32  (-0.39, -0.2) |
| Prevalence | Eritrea | Both | Glaucoma | 2010 | 2013 | 0 | -0.9  (-1.11, -0.73) |
| Prevalence | Cook Islands | Both | Glaucoma | 2015 | 2021 | 0.026794641 | -0.8  (-1.02, -0.18) |
| Prevalence | Eritrea | Female | Glaucoma | 1990 | 1994 | 0 | -0.82  (-1.21, -0.63) |
| Prevalence | Eritrea | Female | Glaucoma | 1994 | 2000 | 0.039992002 | -0.27  (-0.37, -0.02) |
| Prevalence | Eritrea | Female | Glaucoma | 2000 | 2005 | 0.00039992 | -0.89  (-1.17, -0.76) |
| Prevalence | Eritrea | Female | Glaucoma | 2005 | 2010 | 0.23835233 | -0.1  (-0.22, 0.16) |
| Prevalence | Eritrea | Female | Glaucoma | 2010 | 2013 | 0.00119976 | -0.93  (-1.29, -0.53) |
| Prevalence | Indonesia | Male | Glaucoma | 2015 | 2021 | 0 | -0.86  (-0.97, -0.79) |
| Prevalence | Eritrea | Male | Glaucoma | 1990 | 1995 | 0 | -0.46  (-0.51, -0.42) |
| Prevalence | Eritrea | Male | Glaucoma | 1995 | 2000 | 0 | -0.75  (-0.82, -0.7) |
| Prevalence | Eritrea | Male | Glaucoma | 2000 | 2010 | 0 | -0.67  (-0.68, -0.64) |
| Prevalence | Eritrea | Male | Glaucoma | 2010 | 2016 | 0 | -1.02  (-1.06, -0.99) |
| Prevalence | Benin | Female | Glaucoma | 2019 | 2021 | 0 | -0.86  (-1.1, -0.49) |
| Prevalence | Estonia | Both | Glaucoma | 1990 | 1996 | 0 | -0.78  (-0.81, -0.72) |
| Prevalence | Estonia | Both | Glaucoma | 1996 | 2000 | 0 | -0.91  (-1.14, -0.87) |
| Prevalence | Estonia | Both | Glaucoma | 2000 | 2009 | 0 | -1.31  (-1.33, -1.3) |
| Prevalence | Estonia | Both | Glaucoma | 2009 | 2014 | 0 | -0.68  (-0.71, -0.65) |
| Prevalence | Estonia | Both | Glaucoma | 2014 | 2018 | 0 | -0.33  (-0.4, -0.26) |
| Prevalence | Gabon | Both | Glaucoma | 2018 | 2021 | 0 | -0.8  (-1.01, -0.66) |
| Prevalence | Estonia | Female | Glaucoma | 1990 | 1996 | 0 | -0.79  (-0.83, -0.73) |
| Prevalence | Estonia | Female | Glaucoma | 1996 | 2000 | 0 | -0.99  (-1.15, -0.91) |
| Prevalence | Estonia | Female | Glaucoma | 2000 | 2006 | 0 | -1.39  (-1.45, -1.35) |
| Prevalence | Estonia | Female | Glaucoma | 2006 | 2010 | 0 | -1.06  (-1.13, -0.97) |
| Prevalence | Estonia | Female | Glaucoma | 2010 | 2015 | 0 | -0.6  (-0.65, -0.54) |
| Prevalence | Kiribati | Male | Glaucoma | 2019 | 2021 | 0 | -0.86  (-0.96, -0.78) |
| Prevalence | Estonia | Male | Glaucoma | 1990 | 2001 | 0 | -0.72  (-0.74, -0.71) |
| Prevalence | Estonia | Male | Glaucoma | 2001 | 2006 | 0 | -1.22  (-1.24, -1.12) |
| Prevalence | Estonia | Male | Glaucoma | 2006 | 2009 | 0 | -1.42  (-1.48, -1.34) |
| Prevalence | Estonia | Male | Glaucoma | 2009 | 2012 | 0 | -0.8  (-0.86, -0.71) |
| Prevalence | Estonia | Male | Glaucoma | 2012 | 2018 | 0 | -0.42  (-0.45, -0.38) |
| Prevalence | Panama | Female | Glaucoma | 2011 | 2021 | 0 | -0.85  (-0.89, -0.82) |
| Prevalence | Eswatini | Both | Glaucoma | 1990 | 1994 | 0 | -0.67  (-0.84, -0.58) |
| Prevalence | Eswatini | Both | Glaucoma | 1994 | 2000 | 0 | -0.34  (-0.4, -0.22) |
| Prevalence | Eswatini | Both | Glaucoma | 2000 | 2010 | 0 | -0.7  (-0.73, -0.68) |
| Prevalence | Eswatini | Both | Glaucoma | 2010 | 2015 | 0 | -1.55  (-1.63, -1.5) |
| Prevalence | Saint Vincent and the Grenadines | Both | Glaucoma | 2015 | 2021 | 0 | -0.79  (-0.84, -0.75) |
| Prevalence | Eswatini | Female | Glaucoma | 1990 | 1994 | 0 | -0.62  (-0.81, -0.53) |
| Prevalence | Eswatini | Female | Glaucoma | 1994 | 2001 | 0 | -0.31  (-0.35, -0.22) |
| Prevalence | Eswatini | Female | Glaucoma | 2001 | 2008 | 0 | -0.61  (-0.64, -0.56) |
| Prevalence | Eswatini | Female | Glaucoma | 2008 | 2011 | 0 | -0.94  (-1.11, -0.78) |
| Prevalence | Eswatini | Female | Glaucoma | 2011 | 2015 | 0 | -1.39  (-1.51, -1.31) |
| Prevalence | Coted'Ivoire | Male | Glaucoma | 2010 | 2021 | 0 | -0.84  (-1.04, -0.68) |
| Prevalence | Eswatini | Male | Glaucoma | 1990 | 1994 | 0 | -0.74  (-0.99, -0.61) |
| Prevalence | Eswatini | Male | Glaucoma | 1994 | 2001 | 0.00039992 | -0.38  (-0.44, -0.24) |
| Prevalence | Eswatini | Male | Glaucoma | 2001 | 2005 | 0 | -0.92  (-1.08, -0.79) |
| Prevalence | Eswatini | Male | Glaucoma | 2005 | 2010 | 0 | -0.57  (-0.64, -0.44) |
| Prevalence | Eswatini | Male | Glaucoma | 2010 | 2019 | 0 | -1.46  (-1.5, -1.43) |
| Prevalence | Guinea | Female | Glaucoma | 2018 | 2021 | 0 | -0.84  (-0.95, -0.7) |
| Prevalence | Ethiopia | Both | Glaucoma | 1990 | 1996 | 0 | -1.65  (-2.03, -1.39) |
| Prevalence | Ethiopia | Both | Glaucoma | 1996 | 2006 | 0.26194761 | 0.06  (-0.07, 0.22) |
| Prevalence | Ethiopia | Both | Glaucoma | 2006 | 2010 | 0.00479904 | -2.09  (-2.63, -1.57) |
| Prevalence | Ethiopia | Both | Glaucoma | 2010 | 2015 | 0.063187363 | -0.58  (-0.91, 0.08) |
| Prevalence | Ethiopia | Both | Glaucoma | 2015 | 2019 | 0 | 2.8  (2.48, 3.42) |
| Prevalence | Peru | Both | Glaucoma | 2015 | 2021 | 0 | -0.79  (-0.85, -0.73) |
| Prevalence | Ethiopia | Female | Glaucoma | 1990 | 1996 | 0.00079984 | -1.76  (-2.34, -1.39) |
| Prevalence | Ethiopia | Female | Glaucoma | 1996 | 2006 | 0.031593681 | 0.26  (0.07, 0.51) |
| Prevalence | Ethiopia | Female | Glaucoma | 2006 | 2010 | 0.027594481 | -2.67  (-3.38, -1.9) |
| Prevalence | Ethiopia | Female | Glaucoma | 2010 | 2015 | 0.027994401 | -1.13  (-1.64, -0.24) |
| Prevalence | Ethiopia | Female | Glaucoma | 2015 | 2019 | 0 | 4.24  (3.8, 4.99) |
| Prevalence | Colombia | Male | Glaucoma | 2014 | 2021 | 0 | -0.84  (-0.88, -0.78) |
| Prevalence | Ethiopia | Male | Glaucoma | 1990 | 1995 | 0 | -2.03  (-2.36, -1.78) |
| Prevalence | Ethiopia | Male | Glaucoma | 1995 | 2006 | 0.00879824 | -0.2  (-0.29, -0.09) |
| Prevalence | Ethiopia | Male | Glaucoma | 2006 | 2010 | 0.0079984 | -1.46  (-1.9, -1.02) |
| Prevalence | Ethiopia | Male | Glaucoma | 2010 | 2014 | 0.197160568 | -0.36  (-0.85, 0.54) |
| Prevalence | Ethiopia | Male | Glaucoma | 2014 | 2019 | 0 | 1.56  (1.36, 2.04) |
| Prevalence | Portugal | Female | Glaucoma | 2007 | 2021 | 0 | -0.84  (-0.9, -0.76) |
| Prevalence | Fiji | Both | Glaucoma | 1990 | 1995 | 0.019596081 | -1.11  (-2.95, -0.25) |
| Prevalence | Fiji | Both | Glaucoma | 1995 | 2000 | 0.287942412 | 0.3  (-1.78, 1.61) |
| Prevalence | Fiji | Both | Glaucoma | 2000 | 2019 | 0.045990802 | -0.75  (-0.84, -0.1) |
| Prevalence | Guinea | Both | Glaucoma | 2017 | 2021 | 0 | -0.77  (-0.83, -0.7) |
| Prevalence | Fiji | Female | Glaucoma | 1990 | 2007 | 0.226754649 | -0.14  (-0.3, 0.17) |
| Prevalence | Peru | Female | Glaucoma | 2015 | 2021 | 0 | -0.84  (-0.92, -0.75) |
| Prevalence | Fiji | Male | Glaucoma | 1990 | 1994 | 0 | -1.25  (-2.65, -0.57) |
| Prevalence | Fiji | Male | Glaucoma | 1994 | 2000 | 0.189962008 | 0.19  (-0.18, 1.18) |
| Prevalence | Fiji | Male | Glaucoma | 2000 | 2018 | 0.0019996 | -0.87  (-0.95, -0.79) |
| Prevalence | Lesotho | Male | Glaucoma | 2019 | 2021 | 0 | -0.83  (-1.16, -0.69) |
| Prevalence | Finland | Both | Glaucoma | 1990 | 1996 | 0 | -1.32  (-1.37, -1.26) |
| Prevalence | Finland | Both | Glaucoma | 1996 | 2005 | 0 | -1.56  (-1.6, -1.54) |
| Prevalence | Finland | Both | Glaucoma | 2005 | 2009 | 0 | -1.15  (-1.25, -1.01) |
| Prevalence | Finland | Both | Glaucoma | 2009 | 2015 | 0 | -0.54  (-0.59, -0.47) |
| Prevalence | Finland | Both | Glaucoma | 2015 | 2019 | 0 | -1.01  (-1.13, -0.92) |
| Prevalence | El Salvador | Both | Glaucoma | 2005 | 2021 | 0 | -0.77  (-0.78, -0.76) |
| Prevalence | Finland | Female | Glaucoma | 1990 | 1996 | 0 | -1.27  (-1.32, -1.22) |
| Prevalence | Finland | Female | Glaucoma | 1996 | 2005 | 0 | -1.65  (-1.68, -1.62) |
| Prevalence | Finland | Female | Glaucoma | 2005 | 2009 | 0 | -1.23  (-1.34, -1.1) |
| Prevalence | Finland | Female | Glaucoma | 2009 | 2015 | 0 | -0.65  (-0.69, -0.58) |
| Prevalence | Finland | Female | Glaucoma | 2015 | 2019 | 0 | -1.09  (-1.21, -1) |
| Prevalence | Mauritius | Female | Glaucoma | 1998 | 2021 | 0 | -0.83  (-0.85, -0.81) |
| Prevalence | Finland | Male | Glaucoma | 1990 | 1999 | 0 | -1.54  (-1.57, -1.49) |
| Prevalence | Finland | Male | Glaucoma | 1999 | 2005 | 0 | -1.7  (-1.87, -1.64) |
| Prevalence | Finland | Male | Glaucoma | 2005 | 2009 | 0 | -1.25  (-1.45, -1.02) |
| Prevalence | Finland | Male | Glaucoma | 2009 | 2016 | 0 | -0.72  (-0.75, -0.62) |
| Prevalence | Finland | Male | Glaucoma | 2016 | 2019 | 0 | -1.17  (-1.29, -1.03) |
| Prevalence | El Salvador | Male | Glaucoma | 2016 | 2021 | 0 | -0.83  (-0.88, -0.8) |
| Prevalence | France | Both | Glaucoma | 1990 | 1995 | 0 | -1.17  (-1.25, -0.98) |
| Prevalence | France | Both | Glaucoma | 1995 | 2005 | 0 | -1.31  (-1.39, -1.29) |
| Prevalence | France | Both | Glaucoma | 2005 | 2011 | 0 | -0.49  (-0.55, -0.41) |
| Prevalence | France | Both | Glaucoma | 2011 | 2018 | 0 | -0.83  (-0.95, -0.77) |
| Prevalence | Portugal | Both | Glaucoma | 2009 | 2021 | 0 | -0.77  (-0.84, -0.66) |
| Prevalence | France | Female | Glaucoma | 1990 | 1994 | 0 | -1.32  (-1.47, -1.25) |
| Prevalence | France | Female | Glaucoma | 1994 | 2005 | 0 | -1.19  (-1.21, -1.14) |
| Prevalence | France | Female | Glaucoma | 2005 | 2010 | 0 | -0.57  (-1.03, -0.51) |
| Prevalence | France | Female | Glaucoma | 2010 | 2014 | 0 | -0.99  (-1.1, -0.58) |
| Prevalence | France | Female | Glaucoma | 2014 | 2018 | 0 | -0.68  (-0.92, -0.58) |
| Prevalence | Turkmenistan | Female | Glaucoma | 2014 | 2021 | 0 | -0.82  (-0.92, -0.51) |
| Prevalence | France | Male | Glaucoma | 1990 | 1995 | 0 | -0.98  (-1.09, -0.84) |
| Prevalence | France | Male | Glaucoma | 1995 | 2005 | 0 | -1.54  (-1.57, -1.51) |
| Prevalence | France | Male | Glaucoma | 2005 | 2015 | 0 | -0.39  (-0.41, -0.36) |
| Prevalence | France | Male | Glaucoma | 2015 | 2018 | 0 | -1.42  (-1.54, -1.22) |
| Prevalence | South Sudan | Female | Glaucoma | 2019 | 2021 | 0 | -0.82  (-1.07, -0.46) |
| Prevalence | Gabon | Both | Glaucoma | 1990 | 2001 | 0 | -0.8  (-0.87, -0.7) |
| Prevalence | Gabon | Both | Glaucoma | 2001 | 2005 | 0 | -0.89  (-0.99, -0.7) |
| Prevalence | Gabon | Both | Glaucoma | 2005 | 2010 | 0 | -0.66  (-0.91, -0.54) |
| Prevalence | Gabon | Both | Glaucoma | 2010 | 2015 | 0 | -1.11  (-1.22, -0.65) |
| Prevalence | Gabon | Both | Glaucoma | 2015 | 2018 | 0 | -0.54  (-1.11, -0.45) |
| Prevalence | Mongolia | Both | Glaucoma | 2019 | 2021 | 0 | -0.75  (-1.2, -0.49) |
| Prevalence | Gabon | Female | Glaucoma | 1990 | 2004 | 0 | -0.75  (-0.77, -0.74) |
| Prevalence | Gabon | Female | Glaucoma | 2004 | 2010 | 0 | -0.61  (-0.66, -0.52) |
| Prevalence | Gabon | Female | Glaucoma | 2010 | 2015 | 0 | -1.22  (-1.28, -1.17) |
| Prevalence | Guinea | Male | Glaucoma | 2015 | 2021 | 0 | -0.81  (-0.85, -0.78) |
| Prevalence | Gabon | Male | Glaucoma | 1990 | 1999 | 0.012797441 | -0.78  (-0.85, -0.38) |
| Prevalence | Kyrgyzstan | Female | Glaucoma | 2015 | 2021 | 0 | -0.81  (-0.88, -0.71) |
| Prevalence | Gambia | Both | Glaucoma | 1990 | 2001 | 0.597880424 | -0.07  (-0.23, 0.41) |
| Prevalence | Gambia | Both | Glaucoma | 2001 | 2005 | 0.047990402 | -0.96  (-1.63, -0.29) |
| Prevalence | Gambia | Both | Glaucoma | 2005 | 2010 | 0.043191362 | 7.88  (7.29, 8.3) |
| Prevalence | Antigua and Barbuda | Both | Glaucoma | 2015 | 2021 | 0 | -0.75  (-0.78, -0.72) |
| Prevalence | Gambia | Female | Glaucoma | 1990 | 2001 | 0.0119976 | 0.26  (0.1, 0.58) |
| Prevalence | Gambia | Female | Glaucoma | 2001 | 2005 | 0.024795041 | -0.89  (-1.63, -0.2) |
| Prevalence | Gambia | Female | Glaucoma | 2005 | 2010 | 0.014797041 | 7.55  (7.05, 8) |
| Prevalence | Cambodia | Female | Glaucoma | 2016 | 2021 | 0 | -0.8  (-1.18, -0.58) |
| Prevalence | Gambia | Male | Glaucoma | 1990 | 2005 | 0 | -0.51  (-0.64, -0.39) |
| Prevalence | Gambia | Male | Glaucoma | 2005 | 2010 | 0 | 7.96  (7.57, 8.32) |
| Prevalence | Eswatini | Male | Glaucoma | 2019 | 2021 | 0 | -0.8  (-1.09, -0.64) |
| Prevalence | Georgia | Both | Glaucoma | 1990 | 1994 | 0 | -0.54  (-0.69, -0.45) |
| Prevalence | Georgia | Both | Glaucoma | 1994 | 2000 | 0.058788242 | 0.06  (0, 0.13) |
| Prevalence | Georgia | Both | Glaucoma | 2000 | 2010 | 0.00519896 | -0.46  (-0.49, -0.42) |
| Prevalence | Georgia | Both | Glaucoma | 2010 | 2019 | 0 | -0.7  (-0.78, -0.67) |
| Prevalence | Marshall Islands | Both | Glaucoma | 2015 | 2021 | 0.00559888 | -0.74  (-0.9, -0.39) |
| Prevalence | Georgia | Female | Glaucoma | 1990 | 1994 | 0.00119976 | -0.58  (-1.1, -0.37) |
| Prevalence | Georgia | Female | Glaucoma | 1994 | 2000 | 0.279144171 | 0.05  (-0.07, 0.38) |
| Prevalence | Georgia | Female | Glaucoma | 2000 | 2011 | 0.031993601 | -0.42  (-0.63, -0.33) |
| Prevalence | Georgia | Female | Glaucoma | 2011 | 2014 | 0.0019996 | -0.87  (-1.02, -0.21) |
| Prevalence | Italy | Female | Glaucoma | 2015 | 2021 | 0 | -0.8  (-0.95, -0.67) |
| Prevalence | Georgia | Male | Glaucoma | 1990 | 1994 | 0 | -0.63  (-0.79, -0.55) |
| Prevalence | Georgia | Male | Glaucoma | 1994 | 2001 | 0.042791442 | -0.05  (-0.08, 0) |
| Prevalence | Georgia | Male | Glaucoma | 2001 | 2010 | 0.00239952 | -0.59  (-0.61, -0.55) |
| Prevalence | Georgia | Male | Glaucoma | 2010 | 2015 | 0 | -0.81  (-0.86, -0.71) |
| Prevalence | Georgia | Male | Glaucoma | 2015 | 2018 | 0 | -1.15  (-1.24, -1.03) |
| Prevalence | Namibia | Female | Glaucoma | 2017 | 2021 | 0 | -0.8  (-0.85, -0.75) |
| Prevalence | Germany | Both | Glaucoma | 1990 | 1993 | 0 | -1.86  (-2.18, -1.68) |
| Prevalence | Germany | Both | Glaucoma | 1993 | 1996 | 0 | -1.23  (-1.56, -0.84) |
| Prevalence | Germany | Both | Glaucoma | 1996 | 2005 | 0 | -0.8  (-0.88, -0.71) |
| Prevalence | Germany | Both | Glaucoma | 2005 | 2014 | 0 | -0.65  (-0.69, -0.48) |
| Prevalence | Germany | Both | Glaucoma | 2014 | 2019 | 0 | -0.86  (-1.09, -0.79) |
| Prevalence | Lesotho | Both | Glaucoma | 2019 | 2021 | 0 | -0.73  (-0.87, -0.65) |
| Prevalence | Germany | Female | Glaucoma | 1990 | 1993 | 0 | -1.88  (-2.19, -1.7) |
| Prevalence | Germany | Female | Glaucoma | 1993 | 1996 | 0 | -1.26  (-1.59, -0.87) |
| Prevalence | Germany | Female | Glaucoma | 1996 | 2015 | 0 | -0.79  (-0.8, -0.77) |
| Prevalence | Germany | Female | Glaucoma | 2015 | 2019 | 0 | -1.04  (-1.23, -0.93) |
| Prevalence | Sierra Leone | Female | Glaucoma | 2014 | 2021 | 0.00559888 | -0.79  (-0.98, -0.3) |
| Prevalence | Germany | Male | Glaucoma | 1990 | 1995 | 0 | -1.7  (-1.91, -1.56) |
| Prevalence | Germany | Male | Glaucoma | 1995 | 2005 | 0 | -0.88  (-0.96, -0.82) |
| Prevalence | Mongolia | Male | Glaucoma | 2018 | 2021 | 0 | -0.79  (-1.04, -0.36) |
| Prevalence | Ghana | Both | Glaucoma | 1990 | 1992 | 0 | 4.57  (4.19, 4.98) |
| Prevalence | Ghana | Both | Glaucoma | 1992 | 1995 | 0 | 2.59  (2.4, 2.79) |
| Prevalence | Ghana | Both | Glaucoma | 1995 | 2006 | 0 | -0.25  (-0.3, -0.19) |
| Prevalence | Ghana | Both | Glaucoma | 2006 | 2011 | 0 | -0.79  (-0.97, -0.6) |
| Prevalence | Ghana | Both | Glaucoma | 2011 | 2014 | 0 | -1.97  (-2.15, -1.62) |
| Prevalence | Mali | Both | Glaucoma | 2010 | 2021 | 0 | -0.73  (-0.76, -0.69) |
| Prevalence | Ghana | Female | Glaucoma | 1990 | 1993 | 0 | 4.11  (3.78, 4.49) |
| Prevalence | Ghana | Female | Glaucoma | 1993 | 1996 | 0 | 1.58  (1.04, 1.85) |
| Prevalence | Ghana | Female | Glaucoma | 1996 | 2010 | 0 | -0.36  (-0.41, -0.32) |
| Prevalence | Ghana | Female | Glaucoma | 2010 | 2015 | 0 | -1.62  (-2, -1.36) |
| Prevalence | Algeria | Female | Glaucoma | 2018 | 2021 | 0 | -0.79  (-0.81, -0.77) |
| Prevalence | Ghana | Male | Glaucoma | 1990 | 1992 | 0 | 4.69  (4.2, 5.21) |
| Prevalence | Ghana | Male | Glaucoma | 1992 | 1995 | 0 | 2.48  (2.23, 2.73) |
| Prevalence | Ghana | Male | Glaucoma | 1995 | 2006 | 0.0079984 | -0.18  (-0.23, -0.11) |
| Prevalence | Ghana | Male | Glaucoma | 2006 | 2010 | 0 | -0.74  (-1.08, -0.42) |
| Prevalence | Ghana | Male | Glaucoma | 2010 | 2015 | 0 | -1.61  (-1.96, -1.43) |
| Prevalence | Greece | Both | Glaucoma | 1990 | 1997 | 0 | -1.41  (-1.52, -1.32) |
| Prevalence | Greece | Both | Glaucoma | 1997 | 2010 | 0 | -0.89  (-0.93, -0.85) |
| Prevalence | Greece | Both | Glaucoma | 2010 | 2021 | 0 | -0.37  (-0.42, -0.32) |
| Prevalence | Greece | Female | Glaucoma | 1990 | 1997 | 0 | -1.36  (-1.54, -1.25) |
| Prevalence | Greece | Female | Glaucoma | 1997 | 2006 | 0 | -0.79  (-0.97, -0.49) |
| Prevalence | Greece | Female | Glaucoma | 2006 | 2010 | 0 | -1.12  (-1.37, -0.77) |
| Prevalence | Greece | Female | Glaucoma | 2010 | 2019 | 0 | -0.52  (-0.73, -0.42) |
| Prevalence | Greece | Female | Glaucoma | 2019 | 2021 | 0.185163 | 0.33  (-0.29, 0.61) |
| Prevalence | Greece | Male | Glaucoma | 1990 | 1994 | 0 | -1.64  (-1.9, -1.52) |
| Prevalence | Greece | Male | Glaucoma | 1994 | 1999 | 0 | -1.32  (-1.43, -1.03) |
| Prevalence | Greece | Male | Glaucoma | 1999 | 2009 | 0.0004 | -0.94  (-0.97, -0.88) |
| Prevalence | Greece | Male | Glaucoma | 2009 | 2016 | 0.026395 | -0.17  (-0.21, -0.08) |
| Prevalence | Greece | Male | Glaucoma | 2016 | 2021 | 0.0004 | -0.43  (-0.57, -0.35) |
| Prevalence | Angola | Female | Glaucoma | 2015 | 2021 | 0 | -0.79  (-0.91, -0.63) |
| Prevalence | Greenland | Both | Glaucoma | 1990 | 1994 | 0 | 0.45  (0.28, 0.68) |
| Prevalence | Greenland | Both | Glaucoma | 1994 | 1998 | 0.024395121 | -0.3  (-0.55, -0.07) |
| Prevalence | Greenland | Both | Glaucoma | 1998 | 2008 | 0 | -0.7  (-0.91, -0.64) |
| Prevalence | Coted'Ivoire | Both | Glaucoma | 2010 | 2021 | 0 | -0.72  (-0.92, -0.56) |
| Prevalence | Greenland | Female | Glaucoma | 1990 | 1994 | 0 | 0.44  (0.3, 0.68) |
| Prevalence | Greenland | Female | Glaucoma | 1994 | 1997 | 0.084383123 | -0.22  (-0.63, 0.11) |
| Prevalence | Greenland | Female | Glaucoma | 1997 | 2009 | 0 | -0.72  (-0.82, -0.69) |
| Prevalence | Somalia | Male | Glaucoma | 2019 | 2021 | 0 | -0.79  (-0.95, -0.57) |
| Prevalence | Greenland | Male | Glaucoma | 1990 | 1995 | 0.023195361 | 0.35  (0.16, 0.56) |
| Prevalence | Greenland | Male | Glaucoma | 1995 | 2000 | 0.037592482 | -0.55  (-0.78, -0.14) |
| Prevalence | Greenland | Male | Glaucoma | 2000 | 2007 | 0 | -0.87  (-1.2, -0.56) |
| Prevalence | Brunei Darussalam | Male | Glaucoma | 2017 | 2021 | 0 | -0.77  (-0.91, -0.65) |
| Prevalence | Grenada | Both | Glaucoma | 1990 | 2000 | 0 | -1.83  (-1.84, -1.82) |
| Prevalence | Grenada | Both | Glaucoma | 2000 | 2004 | 0 | -1.26  (-1.31, -1.22) |
| Prevalence | Grenada | Both | Glaucoma | 2004 | 2009 | 0 | -1.03  (-1.07, -1) |
| Prevalence | Grenada | Both | Glaucoma | 2009 | 2015 | 0 | -0.66  (-0.68, -0.63) |
| Prevalence | Grenada | Both | Glaucoma | 2015 | 2018 | 0 | -1.04  (-1.09, -0.98) |
| Prevalence | Saint Kitts and Nevis | Both | Glaucoma | 2010 | 2021 | 0 | -0.72  (-0.73, -0.71) |
| Prevalence | Grenada | Female | Glaucoma | 1990 | 1994 | 0 | -1.66  (-1.74, -1.52) |
| Prevalence | Grenada | Female | Glaucoma | 1994 | 2000 | 0 | -1.78  (-1.83, -0.99) |
| Prevalence | Grenada | Female | Glaucoma | 2000 | 2010 | 0 | -0.97  (-1, -0.91) |
| Prevalence | Grenada | Female | Glaucoma | 2010 | 2015 | 0 | -0.69  (-0.73, -0.6) |
| Prevalence | Grenada | Female | Glaucoma | 2015 | 2018 | 0 | -1.23  (-1.31, -1.12) |
| Prevalence | Denmark | Female | Glaucoma | 2015 | 2021 | 0 | -0.77  (-0.87, -0.71) |
| Prevalence | Grenada | Male | Glaucoma | 1990 | 1999 | 0 | -1.84  (-1.87, -1.81) |
| Prevalence | Grenada | Male | Glaucoma | 1999 | 2004 | 0 | -1.21  (-1.33, -1.13) |
| Prevalence | Grenada | Male | Glaucoma | 2004 | 2012 | 0 | -0.75  (-0.8, -0.68) |
| Prevalence | Liberia | Male | Glaucoma | 2019 | 2021 | 0 | -0.77  (-0.96, -0.66) |
| Prevalence | Guam | Both | Glaucoma | 1990 | 1996 | 0.319936013 | -0.08  (-0.24, 0.12) |
| Prevalence | Guam | Both | Glaucoma | 1996 | 1999 | 0 | -2.98  (-3.19, -2.42) |
| Prevalence | Somalia | Both | Glaucoma | 2019 | 2021 | 0 | -0.72  (-0.92, -0.46) |
| Prevalence | Guam | Female | Glaucoma | 1990 | 1996 | 0.437112577 | -0.08  (-0.32, 0.24) |
| Prevalence | Guam | Female | Glaucoma | 1996 | 1999 | 0.027594481 | -2.58  (-2.98, -1.48) |
| Prevalence | Guam | Female | Glaucoma | 1999 | 2009 | 0.220355929 | -0.14  (-0.3, 0.67) |
| Prevalence | Guam | Female | Glaucoma | 2009 | 2015 | 0.01079784 | -0.6  (-1.34, -0.3) |
| Prevalence | United Arab Emirates | Male | Glaucoma | 2018 | 2021 | 0 | -0.76  (-0.88, -0.56) |
| Prevalence | Guam | Male | Glaucoma | 1990 | 1995 | 0.0039992 | 0.2  (0.06, 0.33) |
| Prevalence | Guam | Male | Glaucoma | 1995 | 2000 | 0 | -2.33  (-2.43, -2.23) |
| Prevalence | Guam | Male | Glaucoma | 2000 | 2016 | 0 | -0.38  (-0.41, -0.34) |
| Prevalence | Syrian Arab Republic | Female | Glaucoma | 2018 | 2021 | 0 | -0.76  (-0.84, -0.59) |
| Prevalence | Guatemala | Both | Glaucoma | 1990 | 1999 | 0 | -1.93  (-2.03, -1.86) |
| Prevalence | Guatemala | Both | Glaucoma | 1999 | 2010 | 0 | -1.21  (-1.94, -1.15) |
| Prevalence | Guatemala | Both | Glaucoma | 2010 | 2017 | 0 | -0.9  (-1.29, -0.68) |
| Prevalence | Tokelau | Both | Glaucoma | 2015 | 2021 | 0.014397121 | -0.71  (-0.91, -0.27) |
| Prevalence | Guatemala | Female | Glaucoma | 1990 | 1993 | 0 | -2.06  (-2.29, -1.86) |
| Prevalence | Guatemala | Female | Glaucoma | 1993 | 2000 | 0 | -1.8  (-1.85, -1.04) |
| Prevalence | Guatemala | Female | Glaucoma | 2000 | 2005 | 0 | -0.93  (-1.86, -0.77) |
| Prevalence | Guatemala | Female | Glaucoma | 2005 | 2010 | 0 | -1.23  (-1.39, -0.88) |
| Prevalence | Guatemala | Female | Glaucoma | 2010 | 2019 | 0 | -1.01  (-1.24, -0.94) |
| Prevalence | Ghana | Female | Glaucoma | 2015 | 2021 | 0.0039992 | -0.75  (-0.93, -0.44) |
| Prevalence | Guatemala | Male | Glaucoma | 1990 | 2000 | 0 | -1.89  (-1.95, -1.85) |
| Prevalence | Guatemala | Male | Glaucoma | 2000 | 2010 | 0 | -1.24  (-1.29, -1.18) |
| Prevalence | Guatemala | Male | Glaucoma | 2010 | 2017 | 0 | -0.75  (-0.82, -0.59) |
| Prevalence | Namibia | Male | Glaucoma | 2019 | 2021 | 0 | -0.75  (-1.38, -0.5) |
| Prevalence | Guinea | Both | Glaucoma | 1990 | 1993 | 0 | 1.26  (1.18, 1.34) |
| Prevalence | Guinea | Both | Glaucoma | 1993 | 1996 | 0 | 0.69  (0.57, 0.75) |
| Prevalence | Guinea | Both | Glaucoma | 1996 | 2004 | 0 | 0.18  (0.15, 0.2) |
| Prevalence | Guinea | Both | Glaucoma | 2004 | 2011 | 0 | -0.12  (-0.16, -0.08) |
| Prevalence | Guinea | Both | Glaucoma | 2011 | 2017 | 0 | -0.36  (-0.41, -0.32) |
| Prevalence | Nicaragua | Both | Glaucoma | 2019 | 2021 | 0 | -0.71  (-0.79, -0.67) |
| Prevalence | Guinea | Female | Glaucoma | 1990 | 1993 | 0 | 1.62  (1.52, 1.74) |
| Prevalence | Guinea | Female | Glaucoma | 1993 | 1996 | 0 | 0.98  (0.81, 1.09) |
| Prevalence | Guinea | Female | Glaucoma | 1996 | 2004 | 0 | 0.38  (0.35, 0.41) |
| Prevalence | Guinea | Female | Glaucoma | 2004 | 2011 | 0.063187363 | -0.05  (-0.09, 0.01) |
| Prevalence | Guinea | Female | Glaucoma | 2011 | 2018 | 0 | -0.3  (-0.35, -0.25) |
| Prevalence | Barbados | Male | Glaucoma | 2019 | 2021 | 0 | -0.75  (-0.85, -0.63) |
| Prevalence | Guinea | Male | Glaucoma | 1990 | 1994 | 0 | 1.01  (0.96, 1.06) |
| Prevalence | Guinea | Male | Glaucoma | 1994 | 1998 | 0 | 0.37  (0.31, 0.43) |
| Prevalence | Guinea | Male | Glaucoma | 1998 | 2004 | 0.00359928 | 0.07  (0.03, 0.11) |
| Prevalence | Guinea | Male | Glaucoma | 2004 | 2010 | 0 | -0.22  (-0.26, -0.18) |
| Prevalence | Guinea | Male | Glaucoma | 2010 | 2015 | 0 | -0.45  (-0.51, -0.41) |
| Prevalence | Guatemala | Female | Glaucoma | 2019 | 2021 | 0 | -0.75  (-1, -0.6) |
| Prevalence | Guinea-Bissau | Both | Glaucoma | 1990 | 1993 | 0 | 1.64  (1.41, 2) |
| Prevalence | Guinea-Bissau | Both | Glaucoma | 1993 | 1998 | 0.00039992 | 0.9  (0.67, 1.07) |
| Prevalence | Guinea-Bissau | Both | Glaucoma | 1998 | 2005 | 0.00559888 | 0.23  (0.11, 0.35) |
| Prevalence | Guinea-Bissau | Both | Glaucoma | 2005 | 2011 | 0.01159768 | -0.2  (-0.38, -0.06) |
| Prevalence | Guinea-Bissau | Both | Glaucoma | 2011 | 2019 | 0 | -0.75  (-0.82, -0.66) |
| Prevalence | Tajikistan | Both | Glaucoma | 2015 | 2021 | 0 | -0.71  (-0.79, -0.58) |
| Prevalence | Guinea-Bissau | Female | Glaucoma | 1990 | 1993 | 0 | 1.68  (1.48, 1.89) |
| Prevalence | Guinea-Bissau | Female | Glaucoma | 1993 | 1999 | 0 | 1.05  (0.99, 1.11) |
| Prevalence | Guinea-Bissau | Female | Glaucoma | 1999 | 2010 | 0 | 0.16  (0.13, 0.18) |
| Prevalence | Guinea-Bissau | Female | Glaucoma | 2010 | 2018 | 0 | -0.76  (-0.8, -0.72) |
| Prevalence | Benin | Male | Glaucoma | 2015 | 2021 | 0 | -0.75  (-0.83, -0.67) |
| Prevalence | Guinea-Bissau | Male | Glaucoma | 1990 | 1995 | 0 | 1.54  (1.14, 2.27) |
| Prevalence | Guinea-Bissau | Male | Glaucoma | 1995 | 2006 | 0.00239952 | 0.38  (0.2, 0.53) |
| Prevalence | Fiji | Female | Glaucoma | 2007 | 2021 | 0 | -0.75  (-1.22, -0.55) |
| Prevalence | Guyana | Both | Glaucoma | 1990 | 1994 | 0 | -1.28  (-1.36, -1.19) |
| Prevalence | Guyana | Both | Glaucoma | 1994 | 2000 | 0 | -1.59  (-1.62, -1.55) |
| Prevalence | Guyana | Both | Glaucoma | 2000 | 2010 | 0 | -0.78  (-0.79, -0.76) |
| Prevalence | Guyana | Both | Glaucoma | 2010 | 2019 | 0 | -1.17  (-1.19, -1.15) |
| Prevalence | Yemen | Both | Glaucoma | 2011 | 2021 | 0 | -0.7  (-0.75, -0.65) |
| Prevalence | Guyana | Female | Glaucoma | 1990 | 1995 | 0 | -1.32  (-1.36, -1.26) |
| Prevalence | Guyana | Female | Glaucoma | 1995 | 2000 | 0 | -1.58  (-1.63, -1.53) |
| Prevalence | Guyana | Female | Glaucoma | 2000 | 2006 | 0 | -0.76  (-0.8, -0.71) |
| Prevalence | Guyana | Female | Glaucoma | 2006 | 2010 | 0 | -0.86  (-1.09, -0.8) |
| Prevalence | Guyana | Female | Glaucoma | 2010 | 2018 | 0 | -1.13  (-1.16, -1.09) |
| Prevalence | Morocco | Female | Glaucoma | 2018 | 2021 | 0 | -0.75  (-0.95, -0.48) |
| Prevalence | Guyana | Male | Glaucoma | 1990 | 1994 | 0 | -1.25  (-1.35, -1.08) |
| Prevalence | Guyana | Male | Glaucoma | 1994 | 2000 | 0 | -1.58  (-1.65, -1.54) |
| Prevalence | Guyana | Male | Glaucoma | 2000 | 2010 | 0 | -0.76  (-0.78, -0.73) |
| Prevalence | Guyana | Male | Glaucoma | 2010 | 2019 | 0 | -1.14  (-1.17, -1.11) |
| Prevalence | Tajikistan | Female | Glaucoma | 2013 | 2021 | 0 | -0.74  (-0.82, -0.59) |
| Prevalence | Haiti | Both | Glaucoma | 1990 | 1995 | 0 | -1.07  (-1.13, -0.96) |
| Prevalence | Haiti | Both | Glaucoma | 1995 | 2000 | 0 | -1.41  (-1.5, -1.34) |
| Prevalence | Haiti | Both | Glaucoma | 2000 | 2012 | 0 | -0.87  (-0.89, -0.85) |
| Prevalence | Haiti | Both | Glaucoma | 2012 | 2018 | 0 | -1.04  (-1.15, -0.99) |
| Prevalence | Haiti | Both | Glaucoma | 2018 | 2021 | 0 | -0.67  (-0.78, -0.48) |
| Prevalence | Haiti | Female | Glaucoma | 1990 | 1995 | 0 | -1.09  (-1.17, -0.96) |
| Prevalence | Haiti | Female | Glaucoma | 1995 | 1999 | 0 | -1.48  (-1.66, -1.34) |
| Prevalence | Haiti | Female | Glaucoma | 1999 | 2015 | 0 | -0.94  (-0.95, -0.92) |
| Prevalence | Haiti | Female | Glaucoma | 2015 | 2018 | 0 | -1.27  (-1.37, -1.1) |
| Prevalence | Libya | Female | Glaucoma | 2009 | 2021 | 0.084383123 | -0.73  (-1.15, 0.24) |
| Prevalence | Haiti | Male | Glaucoma | 1990 | 1994 | 0 | -1.14  (-1.35, -0.79) |
| Prevalence | Haiti | Male | Glaucoma | 1994 | 2001 | 0 | -1.45  (-1.68, -0.9) |
| Prevalence | Ecuador | Female | Glaucoma | 1999 | 2021 | 0 | -0.72  (-0.74, -0.7) |
| Prevalence | Honduras | Both | Glaucoma | 1990 | 1996 | 0 | -1.06  (-1.09, -1.02) |
| Prevalence | Honduras | Both | Glaucoma | 1996 | 2000 | 0 | -1.34  (-1.42, -1.27) |
| Prevalence | Honduras | Both | Glaucoma | 2000 | 2005 | 0 | -0.96  (-1.01, -0.91) |
| Prevalence | Honduras | Both | Glaucoma | 2005 | 2015 | 0 | -0.74  (-0.76, -0.71) |
| Prevalence | Honduras | Both | Glaucoma | 2015 | 2019 | 0 | -0.9  (-0.97, -0.8) |
| Prevalence | Denmark | Both | Glaucoma | 2016 | 2021 | 0 | -0.66  (-0.87, -0.57) |
| Prevalence | Honduras | Female | Glaucoma | 1990 | 1996 | 0 | -1.05  (-1.16, -0.88) |
| Prevalence | Honduras | Female | Glaucoma | 1996 | 2005 | 0 | -1.26  (-1.47, -1.07) |
| Prevalence | Honduras | Female | Glaucoma | 2005 | 2009 | 0 | -0.94  (-1.26, -0.67) |
| Prevalence | Honduras | Female | Glaucoma | 2009 | 2015 | 0 | -0.63  (-0.9, -0.41) |
| Prevalence | Uzbekistan | Female | Glaucoma | 2014 | 2021 | 0 | -0.72  (-0.79, -0.57) |
| Prevalence | Honduras | Male | Glaucoma | 1990 | 1995 | 0 | -0.97  (-1.1, -0.67) |
| Prevalence | Honduras | Male | Glaucoma | 1995 | 2001 | 0 | -1.47  (-1.67, -1.34) |
| Prevalence | Honduras | Male | Glaucoma | 2001 | 2019 | 0 | -0.71  (-0.73, -0.69) |
| Prevalence | Antigua and Barbuda | Female | Glaucoma | 2014 | 2021 | 0 | -0.71  (-0.84, -0.65) |
| Prevalence | Hungary | Both | Glaucoma | 1990 | 2001 | 0 | -1.54  (-1.55, -1.52) |
| Prevalence | Hungary | Both | Glaucoma | 2001 | 2004 | 0 | -1.83  (-1.88, -1.72) |
| Prevalence | Hungary | Both | Glaucoma | 2004 | 2009 | 0 | -1.3  (-1.34, -1.25) |
| Prevalence | Hungary | Both | Glaucoma | 2009 | 2015 | 0 | -0.59  (-0.62, -0.55) |
| Prevalence | Hungary | Both | Glaucoma | 2015 | 2018 | 0 | -1.11  (-1.18, -1) |
| Prevalence | Equatorial Guinea | Both | Glaucoma | 2015 | 2021 | 0 | -0.65  (-0.73, -0.56) |
| Prevalence | Hungary | Female | Glaucoma | 1990 | 1994 | 0 | -1.89  (-2.27, -1.7) |
| Prevalence | Hungary | Female | Glaucoma | 1994 | 2000 | 0 | -1.31  (-1.41, -1.04) |
| Prevalence | Hungary | Female | Glaucoma | 2000 | 2008 | 0 | -1.58  (-1.73, -1.52) |
| Prevalence | Hungary | Female | Glaucoma | 2008 | 2015 | 0 | -0.68  (-0.76, -0.52) |
| Prevalence | Hungary | Female | Glaucoma | 2015 | 2019 | 0 | -1.27  (-1.52, -1.12) |
| Prevalence | Chile | Male | Glaucoma | 2011 | 2021 | 0 | -0.71  (-0.76, -0.67) |
| Prevalence | Hungary | Male | Glaucoma | 1990 | 1995 | 0 | -1.23  (-1.33, -1.15) |
| Prevalence | Hungary | Male | Glaucoma | 1995 | 2004 | 0 | -1.82  (-1.85, -1.79) |
| Prevalence | Hungary | Male | Glaucoma | 2004 | 2009 | 0 | -1.18  (-1.31, -1.08) |
| Prevalence | Hungary | Male | Glaucoma | 2009 | 2019 | 0 | -0.59  (-0.61, -0.54) |
| Prevalence | Peru | Male | Glaucoma | 2015 | 2021 | 0 | -0.7  (-0.79, -0.59) |
| Prevalence | Iceland | Both | Glaucoma | 1990 | 2002 | 0 | -1.26  (-1.33, -1.23) |
| Prevalence | Iceland | Both | Glaucoma | 2002 | 2005 | 0.00639872 | -0.94  (-1.13, -0.82) |
| Prevalence | Iceland | Both | Glaucoma | 2005 | 2010 | 0.0059988 | 1.6  (1.52, 1.7) |
| Prevalence | Iceland | Both | Glaucoma | 2010 | 2018 | 0 | -0.83  (-0.92, -0.78) |
| Prevalence | Grenada | Both | Glaucoma | 2018 | 2021 | 0 | -0.64  (-0.69, -0.58) |
| Prevalence | Iceland | Female | Glaucoma | 1990 | 1998 | 0 | -1.46  (-1.56, -1.39) |
| Prevalence | Iceland | Female | Glaucoma | 1998 | 2005 | 0 | -1.07  (-1.15, -0.95) |
| Prevalence | Iceland | Female | Glaucoma | 2005 | 2010 | 0 | 1.63  (1.54, 1.73) |
| Prevalence | Iceland | Female | Glaucoma | 2010 | 2018 | 0 | -0.98  (-1.07, -0.91) |
| Prevalence | Myanmar | Female | Glaucoma | 2014 | 2021 | 0.00119976 | -0.7  (-0.86, -0.54) |
| Prevalence | Iceland | Male | Glaucoma | 1990 | 1995 | 0.00119976 | -0.66  (-0.83, -0.46) |
| Prevalence | Iceland | Male | Glaucoma | 1995 | 2002 | 0 | -1.38  (-1.68, -1.27) |
| Prevalence | Iceland | Male | Glaucoma | 2002 | 2005 | 0.052789442 | -0.81  (-1.1, 0.05) |
| Prevalence | Iceland | Male | Glaucoma | 2005 | 2010 | 0.030393921 | 1.71  (1.51, 1.88) |
| Prevalence | Iceland | Male | Glaucoma | 2010 | 2019 | 0.027994401 | -0.57  (-0.75, -0.49) |
| Prevalence | Bahrain | Male | Glaucoma | 2018 | 2021 | 0 | -0.7  (-0.75, -0.64) |
| Prevalence | India | Both | Glaucoma | 1990 | 1995 | 0.154369126 | -0.33  (-0.65, 0.2) |
| Prevalence | India | Both | Glaucoma | 1995 | 2006 | 0 | -1.21  (-1.35, -1.11) |
| Prevalence | India | Both | Glaucoma | 2006 | 2010 | 0 | -4.11  (-4.64, -3.63) |
| Prevalence | India | Both | Glaucoma | 2010 | 2018 | 0 | -1.01  (-1.24, -0.84) |
| Prevalence | Malta | Both | Glaucoma | 2019 | 2021 | 0 | -0.64  (-0.97, -0.42) |
| Prevalence | India | Female | Glaucoma | 1990 | 1996 | 0.016396721 | -0.5  (-0.69, -0.17) |
| Prevalence | India | Female | Glaucoma | 1996 | 2006 | 0 | -1.1  (-1.22, -1.01) |
| Prevalence | India | Female | Glaucoma | 2006 | 2010 | 0 | -4.51  (-4.69, -4.3) |
| Prevalence | India | Female | Glaucoma | 2010 | 2018 | 0 | -0.69  (-0.83, -0.58) |
| Prevalence | Pakistan | Male | Glaucoma | 2011 | 2021 | 0 | -0.67  (-0.71, -0.63) |
| Prevalence | India | Male | Glaucoma | 1990 | 1996 | 0.080383923 | -0.3  (-0.52, 0.06) |
| Prevalence | India | Male | Glaucoma | 1996 | 2000 | 0 | -1.77  (-2.31, -1.35) |
| Prevalence | India | Male | Glaucoma | 2000 | 2005 | 0.033993201 | -0.58  (-0.84, -0.11) |
| Prevalence | India | Male | Glaucoma | 2005 | 2010 | 0 | -3.51  (-3.83, -3.23) |
| Prevalence | India | Male | Glaucoma | 2010 | 2019 | 0 | -1.1  (-1.25, -0.98) |
| Prevalence | Thailand | Male | Glaucoma | 2009 | 2021 | 0 | -0.67  (-0.72, -0.6) |
| Prevalence | Indonesia | Both | Glaucoma | 1990 | 1994 | 0 | -2.41  (-2.58, -2.24) |
| Prevalence | Indonesia | Both | Glaucoma | 1994 | 2001 | 0.00519896 | -0.42  (-0.5, -0.3) |
| Prevalence | Indonesia | Both | Glaucoma | 2001 | 2005 | 0 | -0.97  (-1.26, -0.77) |
| Prevalence | Indonesia | Both | Glaucoma | 2005 | 2010 | 0.00039992 | -2.03  (-2.18, -1.9) |
| Prevalence | Indonesia | Both | Glaucoma | 2010 | 2015 | 0.034393121 | -0.21  (-0.34, -0.04) |
| Prevalence | Tuvalu | Both | Glaucoma | 2014 | 2021 | 0.032793441 | -0.63  (-0.79, -0.12) |
| Prevalence | Indonesia | Female | Glaucoma | 1990 | 1994 | 0 | -2.88  (-3.06, -2.69) |
| Prevalence | Indonesia | Female | Glaucoma | 1994 | 2001 | 0.00359928 | -0.39  (-0.49, -0.25) |
| Prevalence | Indonesia | Female | Glaucoma | 2001 | 2005 | 0 | -1.22  (-1.55, -0.9) |
| Prevalence | Indonesia | Female | Glaucoma | 2005 | 2010 | 0.00159968 | -2.29  (-2.47, -2.14) |
| Prevalence | Indonesia | Female | Glaucoma | 2010 | 2015 | 0.190761848 | 0.08  (-0.08, 0.25) |
| Prevalence | Malta | Male | Glaucoma | 2019 | 2021 | 0 | -0.67  (-0.81, -0.57) |
| Prevalence | Indonesia | Male | Glaucoma | 1990 | 1994 | 0 | -1.95  (-2.06, -1.84) |
| Prevalence | Indonesia | Male | Glaucoma | 1994 | 2002 | 0.00039992 | -0.43  (-0.48, -0.37) |
| Prevalence | Indonesia | Male | Glaucoma | 2002 | 2006 | 0 | -0.92  (-1.14, -0.78) |
| Prevalence | Indonesia | Male | Glaucoma | 2006 | 2010 | 0 | -1.86  (-1.99, -1.74) |
| Prevalence | Indonesia | Male | Glaucoma | 2010 | 2015 | 0.00039992 | -0.45  (-0.54, -0.31) |
| Prevalence | Malaysia | Male | Glaucoma | 2015 | 2021 | 0 | -0.67  (-0.76, -0.58) |
| Prevalence | Iran (Islamic Republic of) | Both | Glaucoma | 1990 | 1995 | 0.01159768 | -1.22  (-1.5, -0.49) |
| Prevalence | Iran (Islamic Republic of) | Both | Glaucoma | 1995 | 2000 | 0 | -2.5  (-3.14, -2.19) |
| Prevalence | Iran (Islamic Republic of) | Both | Glaucoma | 2000 | 2010 | 0 | -1.36  (-1.5, -1.24) |
| Prevalence | Iran (Islamic Republic of) | Both | Glaucoma | 2010 | 2015 | 0.094781044 | -0.52  (-0.8, 0.22) |
| Prevalence | Iran (Islamic Republic of) | Both | Glaucoma | 2015 | 2019 | 0 | -2.46  (-3.05, -2.06) |
| Prevalence | Northern Mariana Islands | Both | Glaucoma | 2015 | 2021 | 0 | -0.63  (-0.92, -0.49) |
| Prevalence | Iran (Islamic Republic of) | Female | Glaucoma | 1990 | 1995 | 0.031593681 | -0.49  (-0.76, -0.15) |
| Prevalence | Iran (Islamic Republic of) | Female | Glaucoma | 1995 | 2000 | 0.00079984 | -3.09  (-3.53, -2.78) |
| Prevalence | Iran (Islamic Republic of) | Female | Glaucoma | 2000 | 2010 | 0 | -1.28  (-1.46, -1.19) |
| Prevalence | Iran (Islamic Republic of) | Female | Glaucoma | 2010 | 2015 | 0.065186963 | -0.6  (-0.89, 0.06) |
| Prevalence | Iran (Islamic Republic of) | Female | Glaucoma | 2015 | 2019 | 0 | -2.57  (-3.13, -2.19) |
| Prevalence | Yemen | Male | Glaucoma | 2012 | 2021 | 0 | -0.67  (-0.73, -0.59) |
| Prevalence | Iran (Islamic Republic of) | Male | Glaucoma | 1990 | 2001 | 0 | -2.01  (-2.16, -1.92) |
| Prevalence | Iran (Islamic Republic of) | Male | Glaucoma | 2001 | 2009 | 0 | -1.44  (-1.66, -1.21) |
| Prevalence | Iran (Islamic Republic of) | Male | Glaucoma | 2009 | 2015 | 0.094381124 | -0.51  (-0.74, 0.22) |
| Prevalence | Iran (Islamic Republic of) | Male | Glaucoma | 2015 | 2019 | 0 | -2.29  (-2.87, -1.88) |
| Prevalence | Paraguay | Female | Glaucoma | 1990 | 2021 | 0 | -0.66  (-0.67, -0.65) |
| Prevalence | Iraq | Both | Glaucoma | 1990 | 1994 | 0 | -0.72  (-0.77, -0.66) |
| Prevalence | Iraq | Both | Glaucoma | 1994 | 1997 | 0 | -1.05  (-1.19, -0.97) |
| Prevalence | Iraq | Both | Glaucoma | 1997 | 2005 | 0 | -1.37  (-1.39, -1.35) |
| Prevalence | Iraq | Both | Glaucoma | 2005 | 2014 | 0 | -1.87  (-1.89, -1.85) |
| Prevalence | Iraq | Both | Glaucoma | 2014 | 2018 | 0 | -1.61  (-1.73, -1.51) |
| Prevalence | Jamaica | Both | Glaucoma | 2015 | 2021 | 0 | -0.63  (-0.67, -0.6) |
| Prevalence | Iraq | Female | Glaucoma | 1990 | 1996 | 0 | -0.72  (-0.75, -0.69) |
| Prevalence | Iraq | Female | Glaucoma | 1996 | 2001 | 0 | -1.1  (-1.14, -1.04) |
| Prevalence | Iraq | Female | Glaucoma | 2001 | 2005 | 0 | -1.35  (-1.45, -1.3) |
| Prevalence | Iraq | Female | Glaucoma | 2005 | 2015 | 0 | -1.73  (-1.75, -1.72) |
| Prevalence | Iraq | Female | Glaucoma | 2015 | 2019 | 0 | -1.41  (-1.49, -1.33) |
| Prevalence | Liberia | Female | Glaucoma | 2019 | 2021 | 0 | -0.66  (-0.9, -0.53) |
| Prevalence | Iraq | Male | Glaucoma | 1990 | 1993 | 0 | -0.72  (-0.79, -0.59) |
| Prevalence | Iraq | Male | Glaucoma | 1993 | 1996 | 0 | -1.01  (-1.24, -0.94) |
| Prevalence | Iraq | Male | Glaucoma | 1996 | 2005 | 0 | -1.42  (-1.44, -1.4) |
| Prevalence | Iraq | Male | Glaucoma | 2005 | 2015 | 0 | -1.96  (-1.98, -1.95) |
| Prevalence | Iraq | Male | Glaucoma | 2015 | 2019 | 0 | -1.64  (-1.91, -1.58) |
| Prevalence | Angola | Male | Glaucoma | 2019 | 2021 | 0 | -0.66  (-1.17, -0.45) |
| Prevalence | Ireland | Both | Glaucoma | 1990 | 2000 | 0 | -1.13  (-1.16, -1.1) |
| Prevalence | Ireland | Both | Glaucoma | 2000 | 2005 | 0 | -1.79  (-1.85, -1.72) |
| Prevalence | Ireland | Both | Glaucoma | 2005 | 2016 | 0 | -0.82  (-0.84, -0.79) |
| Prevalence | Ireland | Both | Glaucoma | 2016 | 2019 | 0 | -1.11  (-1.23, -0.97) |
| Prevalence | Tonga | Both | Glaucoma | 2016 | 2021 | 0.065986803 | -0.62  (-0.97, 0.07) |
| Prevalence | Ireland | Female | Glaucoma | 1990 | 2000 | 0 | -1.11  (-1.16, -1.06) |
| Prevalence | Ireland | Female | Glaucoma | 2000 | 2005 | 0 | -1.72  (-1.96, -1.52) |
| Prevalence | Ireland | Female | Glaucoma | 2005 | 2015 | 0 | -0.87  (-1.21, -0.75) |
| Prevalence | Ireland | Female | Glaucoma | 2015 | 2019 | 0 | -1.15  (-1.42, -0.87) |
| Prevalence | Ethiopia | Male | Glaucoma | 2019 | 2021 | 0.090781844 | -0.66  (-1.31, 0.13) |
| Prevalence | Ireland | Male | Glaucoma | 1990 | 2000 | 0 | -1.15  (-1.17, -1.13) |
| Prevalence | Ireland | Male | Glaucoma | 2000 | 2004 | 0 | -1.97  (-2.07, -1.91) |
| Prevalence | Ireland | Male | Glaucoma | 2004 | 2007 | 0 | -1.24  (-1.53, -1.05) |
| Prevalence | Ireland | Male | Glaucoma | 2007 | 2015 | 0 | -0.74  (-0.77, -0.68) |
| Prevalence | Ireland | Male | Glaucoma | 2015 | 2019 | 0 | -1.03  (-1.16, -0.94) |
| Prevalence | Philippines | Female | Glaucoma | 2019 | 2021 | 0 | -0.66  (-0.84, -0.5) |
| Prevalence | Israel | Both | Glaucoma | 1990 | 2006 | 0 | -1.19  (-1.22, -1.16) |
| Prevalence | Puerto Rico | Both | Glaucoma | 2015 | 2021 | 0 | -0.61  (-0.63, -0.59) |
| Prevalence | Israel | Female | Glaucoma | 1990 | 2006 | 0 | -1.17  (-1.2, -1.15) |
| Prevalence | Mali | Male | Glaucoma | 2004 | 2021 | 0 | -0.65  (-0.67, -0.62) |
| Prevalence | Israel | Male | Glaucoma | 1990 | 2007 | 0 | -1.15  (-1.17, -1.13) |
| Prevalence | Yemen | Female | Glaucoma | 2011 | 2021 | 0.01079784 | -0.64  (-1.25, -0.35) |
| Prevalence | Italy | Both | Glaucoma | 1990 | 1995 | 0 | -2.12  (-2.28, -1.97) |
| Prevalence | Italy | Both | Glaucoma | 1995 | 2000 | 0 | -3.31  (-3.54, -3.15) |
| Prevalence | Italy | Both | Glaucoma | 2000 | 2004 | 0 | -1.73  (-2.49, -1.54) |
| Prevalence | Italy | Both | Glaucoma | 2004 | 2009 | 0.025994801 | -1.21  (-1.33, -0.52) |
| Prevalence | Italy | Both | Glaucoma | 2009 | 2015 | 0.013597281 | 0.27  (0.15, 0.45) |
| Prevalence | Belize | Both | Glaucoma | 2016 | 2021 | 0 | -0.61  (-0.67, -0.51) |
| Prevalence | Italy | Female | Glaucoma | 1990 | 1995 | 0 | -2.13  (-2.34, -1.92) |
| Prevalence | Italy | Female | Glaucoma | 1995 | 2000 | 0 | -3.46  (-3.88, -3.25) |
| Prevalence | Italy | Female | Glaucoma | 2000 | 2005 | 0 | -2.16  (-2.55, -1.81) |
| Prevalence | Italy | Female | Glaucoma | 2005 | 2010 | 0.00839832 | -1.15  (-1.37, -0.78) |
| Prevalence | Italy | Female | Glaucoma | 2010 | 2015 | 0.00119976 | 1  (0.81, 1.21) |
| Prevalence | Costa Rica | Male | Glaucoma | 2015 | 2021 | 0 | -0.64  (-0.67, -0.61) |
| Prevalence | Italy | Male | Glaucoma | 1990 | 1995 | 0 | -2.14  (-2.32, -1.95) |
| Prevalence | Italy | Male | Glaucoma | 1995 | 2000 | 0 | -3.22  (-3.39, -3.07) |
| Prevalence | Italy | Male | Glaucoma | 2000 | 2008 | 0 | -1.2  (-1.32, -1.12) |
| Prevalence | Germany | Male | Glaucoma | 2005 | 2021 | 0 | -0.64  (-0.66, -0.61) |
| Prevalence | Jamaica | Both | Glaucoma | 1990 | 1995 | 0 | -1.03  (-1.08, -0.98) |
| Prevalence | Jamaica | Both | Glaucoma | 1995 | 2000 | 0 | -1.26  (-1.33, -1.21) |
| Prevalence | Jamaica | Both | Glaucoma | 2000 | 2005 | 0 | -0.85  (-0.89, -0.77) |
| Prevalence | Jamaica | Both | Glaucoma | 2005 | 2010 | 0 | -1.01  (-1.07, -0.97) |
| Prevalence | Jamaica | Both | Glaucoma | 2010 | 2015 | 0 | -0.45  (-0.49, -0.39) |
| Prevalence | Malawi | Both | Glaucoma | 1994 | 2021 | 0 | -0.61  (-0.64, -0.57) |
| Prevalence | Jamaica | Female | Glaucoma | 1990 | 2001 | 0 | -1.15  (-1.18, -1.12) |
| Prevalence | Jamaica | Female | Glaucoma | 2001 | 2005 | 0 | -0.71  (-0.84, -0.56) |
| Prevalence | Jamaica | Female | Glaucoma | 2005 | 2010 | 0 | -1.15  (-1.28, -1.07) |
| Prevalence | Jamaica | Female | Glaucoma | 2010 | 2015 | 0.00359928 | -0.29  (-0.39, -0.17) |
| Prevalence | Jamaica | Female | Glaucoma | 2015 | 2019 | 0 | -0.87  (-1.04, -0.75) |
| Prevalence | Slovenia | Male | Glaucoma | 2019 | 2021 | 0 | -0.64  (-0.84, -0.4) |
| Prevalence | Jamaica | Male | Glaucoma | 1990 | 1994 | 0 | -1.09  (-1.16, -0.98) |
| Prevalence | Jamaica | Male | Glaucoma | 1994 | 2001 | 0 | -1.2  (-1.28, -1.16) |
| Prevalence | Jamaica | Male | Glaucoma | 2001 | 2009 | 0 | -0.95  (-0.98, -0.91) |
| Prevalence | Jamaica | Male | Glaucoma | 2009 | 2015 | 0 | -0.57  (-0.76, -0.55) |
| Prevalence | Jamaica | Male | Glaucoma | 2015 | 2019 | 0 | -0.45  (-0.51, -0.36) |
| Prevalence | Guinea-Bissau | Male | Glaucoma | 2006 | 2021 | 0 | -0.64  (-0.74, -0.55) |
| Prevalence | Japan | Both | Glaucoma | 1990 | 1993 | 0 | -1.59  (-1.72, -1.51) |
| Prevalence | Japan | Both | Glaucoma | 1993 | 1996 | 0 | -1.13  (-1.28, -0.96) |
| Prevalence | Japan | Both | Glaucoma | 1996 | 2000 | 0 | -0.76  (-0.84, -0.65) |
| Prevalence | Japan | Both | Glaucoma | 2000 | 2010 | 0.017596481 | -0.58  (-0.59, -0.55) |
| Prevalence | Japan | Both | Glaucoma | 2010 | 2015 | 0.013597281 | 0.34  (0.3, 0.37) |
| Prevalence | Spain | Both | Glaucoma | 2009 | 2021 | 0 | -0.6  (-0.64, -0.56) |
| Prevalence | Japan | Female | Glaucoma | 1990 | 1994 | 0 | -1.34  (-1.52, -1.22) |
| Prevalence | Japan | Female | Glaucoma | 1994 | 2000 | 0 | -0.96  (-1.02, -0.85) |
| Prevalence | Japan | Female | Glaucoma | 2000 | 2005 | 0.019196161 | -0.21  (-0.3, -0.08) |
| Prevalence | Japan | Female | Glaucoma | 2005 | 2010 | 0 | -0.76  (-0.86, -0.68) |
| Prevalence | Japan | Female | Glaucoma | 2010 | 2015 | 0 | 0.32  (0.24, 0.43) |
| Prevalence | Coted'Ivoire | Female | Glaucoma | 2010 | 2021 | 0 | -0.63  (-0.83, -0.47) |
| Prevalence | Japan | Male | Glaucoma | 1990 | 1994 | 0 | -1.76  (-1.85, -1.67) |
| Prevalence | Japan | Male | Glaucoma | 1994 | 2001 | 0 | -0.75  (-0.8, -0.68) |
| Prevalence | Japan | Male | Glaucoma | 2001 | 2005 | 0 | -1.1  (-1.26, -0.98) |
| Prevalence | Japan | Male | Glaucoma | 2005 | 2009 | 0.00039992 | -0.58  (-0.77, -0.34) |
| Prevalence | Japan | Male | Glaucoma | 2009 | 2017 | 0.399520096 | 0.02  (-0.03, 0.09) |
| Prevalence | Saint Lucia | Male | Glaucoma | 2015 | 2021 | 0.00279944 | -0.62  (-0.73, -0.37) |
| Prevalence | Jordan | Both | Glaucoma | 1990 | 1994 | 0 | -2.33  (-2.51, -2.17) |
| Prevalence | Jordan | Both | Glaucoma | 1994 | 2000 | 0 | -0.99  (-1.07, -0.89) |
| Prevalence | Jordan | Both | Glaucoma | 2000 | 2009 | 0 | -2.26  (-2.31, -2.21) |
| Prevalence | Jordan | Both | Glaucoma | 2009 | 2018 | 0 | -1.15  (-1.22, -1.09) |
| Prevalence | Thailand | Both | Glaucoma | 2018 | 2021 | 0.0019996 | -0.6  (-0.88, -0.25) |
| Prevalence | Jordan | Female | Glaucoma | 1990 | 1992 | 0 | -3.39  (-3.69, -3.02) |
| Prevalence | Jordan | Female | Glaucoma | 1992 | 1995 | 0 | -2.36  (-2.52, -1.58) |
| Prevalence | Jordan | Female | Glaucoma | 1995 | 2000 | 0 | -0.92  (-1.07, -0.78) |
| Prevalence | Jordan | Female | Glaucoma | 2000 | 2014 | 0 | -1.76  (-1.79, -1.73) |
| Prevalence | Jordan | Female | Glaucoma | 2014 | 2018 | 0 | -1.06  (-1.22, -0.79) |
| Prevalence | Armenia | Female | Glaucoma | 2013 | 2021 | 0 | -0.62  (-0.71, -0.48) |
| Prevalence | Jordan | Male | Glaucoma | 1990 | 1994 | 0 | -1.88  (-2.44, -1.6) |
| Prevalence | Jordan | Male | Glaucoma | 1994 | 2000 | 0 | -1.04  (-1.21, -0.75) |
| Prevalence | Jordan | Male | Glaucoma | 2000 | 2006 | 0 | -2.8  (-3.08, -2.24) |
| Prevalence | Jordan | Male | Glaucoma | 2006 | 2009 | 0 | -2.33  (-2.66, -0.65) |
| Prevalence | Bolivia (Plurinational State of) | Male | Glaucoma | 2018 | 2021 | 0 | -0.61  (-0.68, -0.55) |
| Prevalence | Kazakhstan | Both | Glaucoma | 1990 | 1994 | 0 | -0.35  (-0.62, -0.21) |
| Prevalence | Kazakhstan | Both | Glaucoma | 1994 | 2001 | 0.031193761 | 0.07  (0.01, 0.16) |
| Prevalence | Kazakhstan | Both | Glaucoma | 2001 | 2011 | 0.027594481 | -1.38  (-1.41, -1.32) |
| Prevalence | Kazakhstan | Both | Glaucoma | 2011 | 2014 | 0 | -1.78  (-1.89, -1.38) |
| Prevalence | Kazakhstan | Both | Glaucoma | 2014 | 2017 | 0 | -1.04  (-1.64, -0.72) |
| Prevalence | Bosnia and Herzegovina | Both | Glaucoma | 2009 | 2021 | 0 | -0.6  (-0.64, -0.57) |
| Prevalence | Kazakhstan | Female | Glaucoma | 1990 | 1993 | 0 | -0.37  (-0.71, -0.09) |
| Prevalence | Kazakhstan | Female | Glaucoma | 1993 | 2001 | 0.621875625 | -0.02  (-1.25, 0.12) |
| Prevalence | Kazakhstan | Female | Glaucoma | 2001 | 2011 | 0.00039992 | -1.28  (-1.33, -1.2) |
| Prevalence | Kazakhstan | Female | Glaucoma | 2011 | 2015 | 0 | -1.61  (-1.82, -1.39) |
| Prevalence | Azerbaijan | Female | Glaucoma | 2014 | 2021 | 0 | -0.61  (-0.68, -0.51) |
| Prevalence | Kazakhstan | Male | Glaucoma | 1990 | 1994 | 0 | -0.41  (-0.64, -0.27) |
| Prevalence | Kazakhstan | Male | Glaucoma | 1994 | 2001 | 0.00679864 | 0.14  (0.08, 0.21) |
| Prevalence | Kazakhstan | Male | Glaucoma | 2001 | 2011 | 0.01079784 | -1.71  (-1.73, -1.66) |
| Prevalence | Kazakhstan | Male | Glaucoma | 2011 | 2014 | 0 | -2  (-2.11, -1.8) |
| Prevalence | Kazakhstan | Male | Glaucoma | 2014 | 2018 | 0 | -1.26  (-1.39, -1.05) |
| Prevalence | Congo | Male | Glaucoma | 2000 | 2021 | 0 | -0.61  (-0.67, -0.57) |
| Prevalence | Kenya | Both | Glaucoma | 1990 | 1992 | 0 | 6.01  (5.18, 6.86) |
| Prevalence | Kenya | Both | Glaucoma | 1992 | 1995 | 0.00159968 | 2.19  (1.74, 2.65) |
| Prevalence | Kenya | Both | Glaucoma | 1995 | 2000 | 0 | -3.98  (-4.2, -3.77) |
| Prevalence | Kenya | Both | Glaucoma | 2000 | 2010 | 0.00239952 | -0.55  (-0.65, -0.36) |
| Prevalence | Kenya | Both | Glaucoma | 2010 | 2014 | 0 | -1.32  (-1.78, -0.9) |
| Prevalence | Democratic Republic of the Congo | Both | Glaucoma | 2016 | 2021 | 0 | -0.6  (-0.7, -0.45) |
| Prevalence | Kenya | Female | Glaucoma | 1990 | 1992 | 0 | 6.82  (6.01, 7.69) |
| Prevalence | Kenya | Female | Glaucoma | 1992 | 1995 | 0 | 2.49  (2.06, 2.96) |
| Prevalence | Kenya | Female | Glaucoma | 1995 | 2000 | 0 | -4.15  (-4.35, -3.94) |
| Prevalence | Kenya | Female | Glaucoma | 2000 | 2009 | 0.00719856 | -0.35  (-0.46, -0.19) |
| Prevalence | Kenya | Female | Glaucoma | 2009 | 2014 | 0.00239952 | -1.15  (-1.68, -0.85) |
| Prevalence | Israel | Female | Glaucoma | 2006 | 2021 | 0 | -0.6  (-0.63, -0.57) |
| Prevalence | Kenya | Male | Glaucoma | 1990 | 1992 | 0 | 5.5  (4.61, 6.38) |
| Prevalence | Kenya | Male | Glaucoma | 1992 | 1995 | 0.00839832 | 2.11  (1.63, 2.58) |
| Prevalence | Kenya | Male | Glaucoma | 1995 | 2000 | 0 | -3.59  (-3.84, -3.33) |
| Prevalence | Kenya | Male | Glaucoma | 2000 | 2010 | 0.00479904 | -0.6  (-0.71, -0.36) |
| Prevalence | Kenya | Male | Glaucoma | 2010 | 2014 | 0.00079984 | -1.42  (-1.92, -0.96) |
| Prevalence | Grenada | Female | Glaucoma | 2018 | 2021 | 0 | -0.6  (-0.69, -0.49) |
| Prevalence | Kiribati | Both | Glaucoma | 1990 | 2010 | 0.012797441 | -0.52  (-0.56, -0.46) |
| Prevalence | Kiribati | Both | Glaucoma | 2010 | 2015 | 0.0019996 | -1.01  (-1.61, -0.71) |
| Prevalence | Kyrgyzstan | Both | Glaucoma | 2017 | 2021 | 0.0039992 | -0.6  (-0.75, -0.33) |
| Prevalence | Kiribati | Female | Glaucoma | 1990 | 2010 | 0.00639872 | -0.29  (-0.35, -0.2) |
| Prevalence | Kiribati | Female | Glaucoma | 2010 | 2015 | 0 | -1.2  (-2.24, -0.68) |
| Prevalence | Trinidad and Tobago | Male | Glaucoma | 2014 | 2021 | 0 | -0.6  (-0.67, -0.48) |
| Prevalence | Kiribati | Male | Glaucoma | 1990 | 2005 | 0 | -0.7  (-0.7, -0.69) |
| Prevalence | Kiribati | Male | Glaucoma | 2005 | 2009 | 0 | -0.81  (-0.86, -0.77) |
| Prevalence | Kiribati | Male | Glaucoma | 2009 | 2015 | 0 | -0.73  (-0.75, -0.68) |
| Prevalence | Kiribati | Male | Glaucoma | 2015 | 2019 | 0 | -1.2  (-1.25, -1.17) |
| Prevalence | Greenland | Male | Glaucoma | 2007 | 2021 | 0.00079984 | -0.6  (-0.67, -0.52) |
| Prevalence | Kuwait | Both | Glaucoma | 1990 | 1993 | 0 | -0.4  (-0.47, -0.31) |
| Prevalence | Kuwait | Both | Glaucoma | 1993 | 1996 | 0 | -0.86  (-0.94, -0.78) |
| Prevalence | Kuwait | Both | Glaucoma | 1996 | 2000 | 0 | -1.49  (-1.56, -1.44) |
| Prevalence | Kuwait | Both | Glaucoma | 2000 | 2006 | 0 | -1.05  (-1.09, -1) |
| Prevalence | Kuwait | Both | Glaucoma | 2006 | 2018 | 0 | -1.25  (-1.27, -1.24) |
| Prevalence | Azerbaijan | Both | Glaucoma | 2015 | 2021 | 0 | -0.59  (-0.67, -0.48) |
| Prevalence | Kuwait | Female | Glaucoma | 1990 | 1993 | 0 | -0.55  (-0.63, -0.41) |
| Prevalence | Kuwait | Female | Glaucoma | 1993 | 1996 | 0 | -0.97  (-1.05, -0.88) |
| Prevalence | Kuwait | Female | Glaucoma | 1996 | 2000 | 0 | -1.61  (-1.66, -1.56) |
| Prevalence | Kuwait | Female | Glaucoma | 2000 | 2006 | 0 | -1.02  (-1.05, -0.96) |
| Prevalence | Kuwait | Female | Glaucoma | 2006 | 2017 | 0 | -1.11  (-1.14, -1.1) |
| Prevalence | Saudi Arabia | Female | Glaucoma | 2018 | 2021 | 0.00559888 | -0.59  (-0.83, -0.24) |
| Prevalence | Kuwait | Male | Glaucoma | 1990 | 1993 | 0 | -0.79  (-0.84, -0.69) |
| Prevalence | Kuwait | Male | Glaucoma | 1993 | 1996 | 0 | -1.11  (-1.22, -1.03) |
| Prevalence | Kuwait | Male | Glaucoma | 1996 | 2000 | 0 | -1.62  (-1.68, -1.58) |
| Prevalence | Kuwait | Male | Glaucoma | 2000 | 2006 | 0 | -1.15  (-1.19, -1.11) |
| Prevalence | Kuwait | Male | Glaucoma | 2006 | 2018 | 0 | -1.28  (-1.29, -1.27) |
| Prevalence | Denmark | Male | Glaucoma | 2012 | 2021 | 0 | -0.59  (-0.64, -0.48) |
| Prevalence | Kyrgyzstan | Both | Glaucoma | 1990 | 1992 | 0 | -1.03  (-1.29, -0.58) |
| Prevalence | Kyrgyzstan | Both | Glaucoma | 1992 | 1995 | 0.305538892 | -0.39  (-0.51, 0.73) |
| Prevalence | Kyrgyzstan | Both | Glaucoma | 1995 | 2000 | 0.185962807 | 0.65  (-0.73, 0.82) |
| Prevalence | Kyrgyzstan | Both | Glaucoma | 2000 | 2010 | 0 | -0.67  (-1.23, -0.6) |
| Prevalence | Kyrgyzstan | Both | Glaucoma | 2010 | 2017 | 0 | -1.27  (-1.39, -1.17) |
| Prevalence | Italy | Both | Glaucoma | 2015 | 2021 | 0.00079984 | -0.58  (-0.71, -0.46) |
| Prevalence | Kyrgyzstan | Female | Glaucoma | 1990 | 1995 | 0 | -0.58  (-0.66, -0.5) |
| Prevalence | Kyrgyzstan | Female | Glaucoma | 1995 | 2000 | 0 | 0.68  (0.61, 0.76) |
| Prevalence | Kyrgyzstan | Female | Glaucoma | 2000 | 2005 | 0 | -0.84  (-0.97, -0.76) |
| Prevalence | Kyrgyzstan | Female | Glaucoma | 2005 | 2010 | 0.00079984 | -0.38  (-0.49, -0.22) |
| Prevalence | Kyrgyzstan | Female | Glaucoma | 2010 | 2015 | 0 | -1.3  (-1.44, -1.2) |
| Prevalence | Kuwait | Female | Glaucoma | 2017 | 2021 | 0 | -0.59  (-0.64, -0.54) |
| Prevalence | Kyrgyzstan | Male | Glaucoma | 1990 | 1994 | 0 | -0.86  (-0.97, -0.75) |
| Prevalence | Kyrgyzstan | Male | Glaucoma | 1994 | 2001 | 0 | 0.42  (0.37, 0.47) |
| Prevalence | Kyrgyzstan | Male | Glaucoma | 2001 | 2009 | 0 | -0.86  (-0.92, -0.81) |
| Prevalence | Kyrgyzstan | Male | Glaucoma | 2009 | 2018 | 0 | -1.36  (-1.42, -1.31) |
| Prevalence | Greenland | Female | Glaucoma | 2009 | 2021 | 0 | -0.59  (-0.62, -0.53) |
| Prevalence | Lao People's Democratic Republic | Both | Glaucoma | 1990 | 1994 | 0 | -0.77  (-1.18, -0.54) |
| Prevalence | Lao People's Democratic Republic | Both | Glaucoma | 1994 | 2001 | 0.50789842 | -0.05  (-0.15, 0.23) |
| Prevalence | Lao People's Democratic Republic | Both | Glaucoma | 2001 | 2007 | 0 | -0.74  (-0.92, -0.55) |
| Prevalence | Malaysia | Both | Glaucoma | 2015 | 2021 | 0 | -0.57  (-0.71, -0.45) |
| Prevalence | Lao People's Democratic Republic | Female | Glaucoma | 1990 | 2005 | 0.0059988 | -0.18  (-0.24, -0.11) |
| Prevalence | Lao People's Democratic Republic | Female | Glaucoma | 2005 | 2014 | 0.00239952 | -1.18  (-1.3, -0.76) |
| Prevalence | New Zealand | Male | Glaucoma | 2015 | 2021 | 0 | -0.58  (-0.61, -0.53) |
| Prevalence | Lao People's Democratic Republic | Male | Glaucoma | 1990 | 1994 | 0 | -1.08  (-1.14, -1.01) |
| Prevalence | Lao People's Democratic Republic | Male | Glaucoma | 1994 | 2001 | 0.00039992 | -0.14  (-0.17, -0.1) |
| Prevalence | Lao People's Democratic Republic | Male | Glaucoma | 2001 | 2006 | 0 | -0.94  (-1, -0.86) |
| Prevalence | Lao People's Democratic Republic | Male | Glaucoma | 2006 | 2019 | 0 | -1.28  (-1.3, -1.27) |
| Prevalence | Austria | Female | Glaucoma | 2006 | 2021 | 0 | -0.58  (-0.59, -0.55) |
| Prevalence | Latvia | Both | Glaucoma | 1990 | 1994 | 0 | -1.05  (-1.12, -0.98) |
| Prevalence | Latvia | Both | Glaucoma | 1994 | 2001 | 0 | -0.37  (-0.41, -0.34) |
| Prevalence | Latvia | Both | Glaucoma | 2001 | 2010 | 0 | -1.04  (-1.06, -1.01) |
| Prevalence | Latvia | Both | Glaucoma | 2010 | 2015 | 0 | -0.38  (-0.43, -0.32) |
| Prevalence | Latvia | Both | Glaucoma | 2015 | 2019 | 0 | -0.99  (-1.07, -0.9) |
| Prevalence | Benin | Both | Glaucoma | 2016 | 2021 | 0 | -0.57  (-0.68, -0.46) |
| Prevalence | Latvia | Female | Glaucoma | 1990 | 1994 | 0 | -0.97  (-1.15, -0.87) |
| Prevalence | Latvia | Female | Glaucoma | 1994 | 2001 | 0 | -0.42  (-0.47, -0.35) |
| Prevalence | Latvia | Female | Glaucoma | 2001 | 2009 | 0 | -1  (-1.05, -0.95) |
| Prevalence | Latvia | Female | Glaucoma | 2009 | 2015 | 0 | -0.43  (-0.5, -0.32) |
| Prevalence | Latvia | Female | Glaucoma | 2015 | 2019 | 0 | -1  (-1.12, -0.89) |
| Prevalence | Bahamas | Male | Glaucoma | 2012 | 2021 | 0.00119976 | -0.57  (-0.63, -0.46) |
| Prevalence | Latvia | Male | Glaucoma | 1990 | 1994 | 0 | -1.05  (-1.31, -0.91) |
| Prevalence | Latvia | Male | Glaucoma | 1994 | 2002 | 0.00039992 | -0.34  (-0.39, -0.27) |
| Prevalence | Latvia | Male | Glaucoma | 2002 | 2010 | 0 | -1.22  (-1.29, -1.17) |
| Prevalence | Latvia | Male | Glaucoma | 2010 | 2015 | 0 | -0.47  (-0.57, -0.23) |
| Prevalence | Latvia | Male | Glaucoma | 2015 | 2019 | 0 | -1.01  (-1.24, -0.88) |
| Prevalence | Brazil | Female | Glaucoma | 2019 | 2021 | 0.24315137 | -0.57  (-2.3, 0.47) |
| Prevalence | Lebanon | Both | Glaucoma | 1990 | 2004 | 0 | -1.44  (-1.46, -1.41) |
| Prevalence | Lebanon | Both | Glaucoma | 2004 | 2011 | 0.00279944 | 0.11  (0.06, 0.16) |
| Prevalence | Lebanon | Both | Glaucoma | 2011 | 2015 | 0.00359928 | -1.71  (-1.8, -1.53) |
| Prevalence | Lebanon | Both | Glaucoma | 2015 | 2018 | 0 | -2.38  (-2.53, -2.15) |
| Prevalence | Paraguay | Both | Glaucoma | 2014 | 2021 | 0.00039992 | -0.57  (-0.63, -0.43) |
| Prevalence | Lebanon | Female | Glaucoma | 1990 | 1995 | 0 | -1.23  (-1.32, -1.02) |
| Prevalence | Lebanon | Female | Glaucoma | 1995 | 2004 | 0.00279944 | -1.41  (-1.49, -1.37) |
| Prevalence | Lebanon | Female | Glaucoma | 2004 | 2011 | 0.025594881 | 0.07  (0.02, 0.13) |
| Prevalence | Lebanon | Female | Glaucoma | 2011 | 2015 | 0.026394721 | -1.51  (-1.6, -1.29) |
| Prevalence | Lebanon | Female | Glaucoma | 2015 | 2019 | 0 | -1.87  (-2.04, -1.75) |
| Prevalence | Malaysia | Female | Glaucoma | 2015 | 2021 | 0 | -0.57  (-0.73, -0.34) |
| Prevalence | Lebanon | Male | Glaucoma | 1990 | 2004 | 0 | -1.64  (-1.66, -1.61) |
| Prevalence | Lebanon | Male | Glaucoma | 2004 | 2011 | 0.00159968 | 0.16  (0.1, 0.21) |
| Prevalence | Lebanon | Male | Glaucoma | 2011 | 2015 | 0.00239952 | -1.75  (-1.86, -1.58) |
| Prevalence | Lebanon | Male | Glaucoma | 2015 | 2018 | 0 | -2.49  (-2.65, -2.25) |
| Prevalence | Turkmenistan | Male | Glaucoma | 2018 | 2021 | 0 | -0.56  (-0.71, -0.22) |
| Prevalence | Lesotho | Both | Glaucoma | 1990 | 1994 | 0 | -0.52  (-0.57, -0.41) |
| Prevalence | Lesotho | Both | Glaucoma | 1994 | 2000 | 0 | -0.65  (-0.74, -0.62) |
| Prevalence | Lesotho | Both | Glaucoma | 2000 | 2008 | 0 | -0.32  (-0.34, -0.29) |
| Prevalence | Lesotho | Both | Glaucoma | 2008 | 2011 | 0 | -0.63  (-0.74, -0.55) |
| Prevalence | Lesotho | Both | Glaucoma | 2011 | 2019 | 0 | -1.14  (-1.17, -1.12) |
| Prevalence | Israel | Both | Glaucoma | 2006 | 2021 | 0 | -0.57  (-0.59, -0.53) |
| Prevalence | Lesotho | Female | Glaucoma | 1990 | 2000 | 0 | -0.45  (-0.47, -0.43) |
| Prevalence | Lesotho | Female | Glaucoma | 2000 | 2006 | 0 | -0.2  (-0.24, -0.15) |
| Prevalence | Lesotho | Female | Glaucoma | 2006 | 2011 | 0 | -0.6  (-0.64, -0.57) |
| Prevalence | Lesotho | Female | Glaucoma | 2011 | 2019 | 0 | -1.18  (-1.2, -1.16) |
| Prevalence | Lesotho | Female | Glaucoma | 2019 | 2021 | 0 | -0.56  (-0.68, -0.47) |
| Prevalence | Lesotho | Male | Glaucoma | 1990 | 2002 | 0 | -0.53  (-0.58, -0.5) |
| Prevalence | Lesotho | Male | Glaucoma | 2002 | 2010 | 0 | -0.39  (-0.43, -0.29) |
| Prevalence | Lesotho | Male | Glaucoma | 2010 | 2019 | 0 | -1.25  (-1.33, -1.22) |
| Prevalence | Gabon | Female | Glaucoma | 2015 | 2021 | 0 | -0.55  (-0.6, -0.51) |
| Prevalence | Liberia | Both | Glaucoma | 1990 | 1995 | 0.070385923 | -0.05  (-0.11, 0.01) |
| Prevalence | Liberia | Both | Glaucoma | 1995 | 1999 | 0.013597281 | -0.8  (-0.9, -0.65) |
| Prevalence | Liberia | Both | Glaucoma | 1999 | 2005 | 0 | -1.4  (-1.45, -1.24) |
| Prevalence | Liberia | Both | Glaucoma | 2005 | 2011 | 0 | -0.71  (-1.38, -0.65) |
| Prevalence | Liberia | Both | Glaucoma | 2011 | 2019 | 0 | -1.06  (-1.14, -0.7) |
| Prevalence | Colombia | Both | Glaucoma | 2019 | 2021 | 0 | -0.56  (-0.7, -0.47) |
| Prevalence | Liberia | Female | Glaucoma | 1990 | 1996 | 0.038392322 | -0.06  (-0.12, -0.01) |
| Prevalence | Liberia | Female | Glaucoma | 1996 | 2000 | 0.026394721 | -0.84  (-0.91, -0.14) |
| Prevalence | Liberia | Female | Glaucoma | 2000 | 2005 | 0 | -1.19  (-1.3, -0.9) |
| Prevalence | Liberia | Female | Glaucoma | 2005 | 2014 | 0 | -0.62  (-1.18, -0.58) |
| Prevalence | Liberia | Female | Glaucoma | 2014 | 2019 | 0 | -0.95  (-1.08, -0.58) |
| Prevalence | Dominican Republic | Female | Glaucoma | 2018 | 2021 | 0.01239752 | -0.55  (-0.77, -0.13) |
| Prevalence | Liberia | Male | Glaucoma | 1990 | 1996 | 0 | -0.17  (-0.21, -0.13) |
| Prevalence | Liberia | Male | Glaucoma | 1996 | 2000 | 0 | -1.1  (-1.18, -1.03) |
| Prevalence | Liberia | Male | Glaucoma | 2000 | 2005 | 0 | -1.52  (-1.57, -1.47) |
| Prevalence | Liberia | Male | Glaucoma | 2005 | 2011 | 0 | -0.76  (-0.8, -0.72) |
| Prevalence | Liberia | Male | Glaucoma | 2011 | 2019 | 0 | -1.22  (-1.26, -1.19) |
| Prevalence | Serbia | Male | Glaucoma | 2013 | 2021 | 0 | -0.55  (-0.6, -0.48) |
| Prevalence | Libya | Both | Glaucoma | 1990 | 1994 | 0.00119976 | -2.67  (-5.09, -1.49) |
| Prevalence | Libya | Both | Glaucoma | 1994 | 2001 | 0.063587283 | 0.42  (-0.03, 1.85) |
| Prevalence | Libya | Both | Glaucoma | 2001 | 2009 | 0.015596881 | -2.53  (-3.1, -2.05) |
| Prevalence | Libya | Both | Glaucoma | 2009 | 2019 | 0.133573285 | -0.42  (-2.14, 0.43) |
| Prevalence | Suriname | Both | Glaucoma | 2015 | 2021 | 0 | -0.55  (-0.6, -0.51) |
| Prevalence | Libya | Female | Glaucoma | 1990 | 1995 | 0.055188962 | -1.77  (-5.8, 0.08) |
| Prevalence | Libya | Female | Glaucoma | 1995 | 2000 | 0.049590082 | 1.86  (0.07, 4.5) |
| Prevalence | Libya | Female | Glaucoma | 2000 | 2009 | 0.019996001 | -2.17  (-4.15, -1.41) |
| Prevalence | Seychelles | Female | Glaucoma | 2018 | 2021 | 0.00759848 | -0.55  (-0.75, -0.17) |
| Prevalence | Libya | Male | Glaucoma | 1990 | 1994 | 0.00079984 | -2.93  (-4.8, -1.97) |
| Prevalence | Libya | Male | Glaucoma | 1994 | 2001 | 0.859828034 | 0.01  (-0.37, 0.91) |
| Prevalence | Libya | Male | Glaucoma | 2001 | 2009 | 0.014797041 | -2.58  (-2.97, -2.19) |
| Prevalence | Libya | Male | Glaucoma | 2009 | 2019 | 0.103979204 | -0.36  (-2.35, 0.21) |
| Prevalence | Burkina Faso | Male | Glaucoma | 2010 | 2021 | 0.00359928 | -0.54  (-0.64, -0.37) |
| Prevalence | Lithuania | Both | Glaucoma | 1990 | 1995 | 0 | -0.7  (-0.78, -0.55) |
| Prevalence | Lithuania | Both | Glaucoma | 1995 | 2009 | 0 | -0.86  (-0.9, -0.84) |
| Prevalence | Lithuania | Both | Glaucoma | 2009 | 2015 | 0 | -0.56  (-0.63, -0.4) |
| Prevalence | Lithuania | Both | Glaucoma | 2015 | 2019 | 0 | -1.05  (-1.24, -0.94) |
| Prevalence | Kuwait | Both | Glaucoma | 2018 | 2021 | 0 | -0.55  (-0.62, -0.5) |
| Prevalence | Lithuania | Female | Glaucoma | 1990 | 1996 | 0 | -0.59  (-0.65, -0.49) |
| Prevalence | Lithuania | Female | Glaucoma | 1996 | 2007 | 0 | -0.86  (-0.91, -0.83) |
| Prevalence | Lithuania | Female | Glaucoma | 2007 | 2015 | 0 | -0.54  (-0.59, -0.44) |
| Prevalence | Lithuania | Female | Glaucoma | 2015 | 2019 | 0 | -0.97  (-1.18, -0.85) |
| Prevalence | Argentina | Male | Glaucoma | 2019 | 2021 | 0 | -0.54  (-0.8, -0.38) |
| Prevalence | Lithuania | Male | Glaucoma | 1990 | 2000 | 0 | -0.8  (-0.9, -0.78) |
| Prevalence | Lithuania | Male | Glaucoma | 2000 | 2005 | 0 | -0.69  (-0.76, -0.58) |
| Prevalence | Lithuania | Male | Glaucoma | 2005 | 2010 | 0 | -0.95  (-1.12, -0.88) |
| Prevalence | Lithuania | Male | Glaucoma | 2010 | 2015 | 0 | -0.59  (-0.66, -0.46) |
| Prevalence | Lithuania | Male | Glaucoma | 2015 | 2019 | 0 | -1.22  (-1.31, -1.12) |
| Prevalence | Kazakhstan | Female | Glaucoma | 2015 | 2021 | 0.00039992 | -0.54  (-0.63, -0.41) |
| Prevalence | Luxembourg | Both | Glaucoma | 1990 | 1995 | 0 | -1.31  (-1.39, -1.26) |
| Prevalence | Luxembourg | Both | Glaucoma | 1995 | 2010 | 0 | -1.07  (-1.08, -1.06) |
| Prevalence | Luxembourg | Both | Glaucoma | 2010 | 2015 | 0 | -0.8  (-0.85, -0.68) |
| Prevalence | Luxembourg | Both | Glaucoma | 2015 | 2019 | 0 | -0.99  (-1.1, -0.94) |
| Prevalence | Democratic People's Republic of Korea | Both | Glaucoma | 2018 | 2021 | 0 | -0.55  (-0.91, -0.36) |
| Prevalence | Luxembourg | Female | Glaucoma | 1990 | 1994 | 0 | -1.32  (-1.55, -1.23) |
| Prevalence | Luxembourg | Female | Glaucoma | 1994 | 2019 | 0 | -1.08  (-1.08, -1.07) |
| Prevalence | Mauritius | Male | Glaucoma | 2016 | 2021 | 0 | -0.54  (-0.57, -0.5) |
| Prevalence | Luxembourg | Male | Glaucoma | 1990 | 1996 | 0 | -1.33  (-1.39, -1.29) |
| Prevalence | Luxembourg | Male | Glaucoma | 1996 | 2005 | 0 | -1.12  (-1.2, -1.1) |
| Prevalence | Luxembourg | Male | Glaucoma | 2005 | 2010 | 0 | -1.04  (-1.09, -0.86) |
| Prevalence | Luxembourg | Male | Glaucoma | 2010 | 2015 | 0 | -0.66  (-0.7, -0.58) |
| Prevalence | Luxembourg | Male | Glaucoma | 2015 | 2019 | 0 | -0.94  (-1.02, -0.88) |
| Prevalence | Bosnia and Herzegovina | Male | Glaucoma | 2009 | 2021 | 0 | -0.52  (-0.56, -0.48) |
| Prevalence | Madagascar | Both | Glaucoma | 1990 | 1994 | 0 | -1.01  (-1.53, -0.8) |
| Prevalence | Madagascar | Both | Glaucoma | 1994 | 2000 | 0.081983603 | -0.32  (-0.42, 0.03) |
| Prevalence | Madagascar | Both | Glaucoma | 2000 | 2014 | 0 | -0.61  (-0.65, -0.58) |
| Prevalence | Bahrain | Both | Glaucoma | 2018 | 2021 | 0 | -0.54  (-0.6, -0.49) |
| Prevalence | Madagascar | Female | Glaucoma | 1990 | 1994 | 0 | -1.32  (-1.41, -1.23) |
| Prevalence | Madagascar | Female | Glaucoma | 1994 | 2000 | 0.101579684 | -0.05  (-0.12, 0.03) |
| Prevalence | Madagascar | Female | Glaucoma | 2000 | 2014 | 0 | -0.55  (-0.57, -0.53) |
| Prevalence | Madagascar | Female | Glaucoma | 2014 | 2019 | 0 | -0.99  (-1.16, -0.9) |
| Prevalence | Sao Tome and Principe | Female | Glaucoma | 2019 | 2021 | 0 | -0.52  (-0.66, -0.42) |
| Prevalence | Madagascar | Male | Glaucoma | 1990 | 2001 | 0.079184163 | -0.5  (-0.59, 0.41) |
| Prevalence | Madagascar | Male | Glaucoma | 2001 | 2018 | 0.00879824 | -0.62  (-0.75, -0.54) |
| Prevalence | Luxembourg | Female | Glaucoma | 2019 | 2021 | 0 | -0.52  (-0.76, -0.41) |
| Prevalence | Malawi | Both | Glaucoma | 1990 | 1994 | 0 | -4.51  (-4.76, -4.19) |
| Prevalence | Saint Lucia | Both | Glaucoma | 2015 | 2021 | 0 | -0.54  (-0.58, -0.48) |
| Prevalence | Malawi | Female | Glaucoma | 1990 | 1994 | 0 | -4.57  (-5.03, -4.18) |
| Prevalence | Malawi | Female | Glaucoma | 1994 | 2005 | 0.00159968 | -0.77  (-1.24, -0.65) |
| Prevalence | Georgia | Female | Glaucoma | 2014 | 2021 | 0.00359928 | -0.52  (-0.64, -0.2) |
| Prevalence | Malawi | Male | Glaucoma | 1990 | 1994 | 0 | -4.45  (-4.68, -4.24) |
| Prevalence | Malawi | Male | Glaucoma | 1994 | 2000 | 0.340331934 | -0.1  (-0.25, 0.21) |
| Prevalence | Malawi | Male | Glaucoma | 2000 | 2005 | 0.00039992 | -0.8  (-1.27, -0.59) |
| Prevalence | Malawi | Male | Glaucoma | 2005 | 2010 | 0.366326735 | 0.1  (-0.14, 0.6) |
| Prevalence | Togo | Male | Glaucoma | 2010 | 2021 | 0 | -0.51  (-0.54, -0.49) |
| Prevalence | Malaysia | Both | Glaucoma | 1990 | 2001 | 0 | -0.83  (-0.89, -0.76) |
| Prevalence | Malaysia | Both | Glaucoma | 2001 | 2005 | 0 | 1.19  (1.03, 1.35) |
| Prevalence | Malaysia | Both | Glaucoma | 2005 | 2015 | 0 | -1.96  (-2.02, -1.9) |
| Prevalence | Greenland | Both | Glaucoma | 2008 | 2021 | 0.00039992 | -0.53  (-0.57, -0.46) |
| Prevalence | Malaysia | Female | Glaucoma | 1990 | 2000 | 0 | -0.9  (-1.01, -0.8) |
| Prevalence | Malaysia | Female | Glaucoma | 2000 | 2006 | 0 | 0.41  (0.22, 0.67) |
| Prevalence | Malaysia | Female | Glaucoma | 2006 | 2009 | 0 | -3.62  (-3.86, -3.26) |
| Prevalence | Malaysia | Female | Glaucoma | 2009 | 2015 | 0 | -1.73  (-1.93, -1.54) |
| Prevalence | Chad | Male | Glaucoma | 2014 | 2021 | 0 | -0.51  (-0.54, -0.46) |
| Prevalence | Malaysia | Male | Glaucoma | 1990 | 2000 | 0 | -0.87  (-0.92, -0.82) |
| Prevalence | Malaysia | Male | Glaucoma | 2000 | 2005 | 0 | 1.1  (1, 1.19) |
| Prevalence | Malaysia | Male | Glaucoma | 2005 | 2011 | 0 | -1.58  (-1.67, -1.46) |
| Prevalence | Malaysia | Male | Glaucoma | 2011 | 2015 | 0 | -2.26  (-2.48, -2.1) |
| Prevalence | United Arab Emirates | Female | Glaucoma | 2017 | 2021 | 0 | -0.51  (-0.64, -0.31) |
| Prevalence | Maldives | Both | Glaucoma | 1990 | 1995 | 0 | -2.21  (-2.39, -2.06) |
| Prevalence | Maldives | Both | Glaucoma | 1995 | 2000 | 0 | -3.51  (-3.63, -3.37) |
| Prevalence | Maldives | Both | Glaucoma | 2000 | 2010 | 0 | -1.35  (-1.44, -1.3) |
| Prevalence | Maldives | Both | Glaucoma | 2010 | 2015 | 0 | -0.87  (-1, -0.56) |
| Prevalence | Maldives | Both | Glaucoma | 2015 | 2018 | 0 | -1.68  (-1.89, -1.39) |
| Prevalence | Seychelles | Both | Glaucoma | 2018 | 2021 | 0 | -0.52  (-0.65, -0.27) |
| Prevalence | Maldives | Female | Glaucoma | 1990 | 1995 | 0 | -1.85  (-2.18, -1.43) |
| Prevalence | Maldives | Female | Glaucoma | 1995 | 2000 | 0 | -3.64  (-4.04, -3.34) |
| Prevalence | Tajikistan | Male | Glaucoma | 2018 | 2021 | 0.00359928 | -0.5  (-0.75, -0.18) |
| Prevalence | Maldives | Male | Glaucoma | 1990 | 1995 | 0 | -2.25  (-2.45, -2.07) |
| Prevalence | Maldives | Male | Glaucoma | 1995 | 1999 | 0 | -3.75  (-3.94, -3.52) |
| Prevalence | Maldives | Male | Glaucoma | 1999 | 2010 | 0 | -1.56  (-1.63, -1.5) |
| Prevalence | Maldives | Male | Glaucoma | 2010 | 2015 | 0 | -0.76  (-0.92, -0.39) |
| Prevalence | Maldives | Male | Glaucoma | 2015 | 2019 | 0 | -1.4  (-1.74, -1.2) |
| Prevalence | Malawi | Female | Glaucoma | 2005 | 2021 | 0.013597281 | -0.5  (-0.57, -0.27) |
| Prevalence | Mali | Both | Glaucoma | 1990 | 1993 | 0 | 2.15  (1.95, 2.33) |
| Prevalence | Mali | Both | Glaucoma | 1993 | 1998 | 0 | 1.3  (1.21, 1.39) |
| Prevalence | Mali | Both | Glaucoma | 1998 | 2001 | 0.00119976 | 0.26  (0.14, 0.4) |
| Prevalence | Mali | Both | Glaucoma | 2001 | 2005 | 0 | -1.74  (-1.83, -1.65) |
| Prevalence | Mali | Both | Glaucoma | 2005 | 2010 | 0.00119976 | -0.33  (-0.42, -0.22) |
| Prevalence | Burkina Faso | Both | Glaucoma | 2014 | 2021 | 0.052389522 | -0.51  (-0.67, 0.01) |
| Prevalence | Mali | Female | Glaucoma | 1990 | 1994 | 0 | 1.97  (1.77, 2.22) |
| Prevalence | Mali | Female | Glaucoma | 1994 | 2000 | 0 | 1.15  (1.03, 1.26) |
| Prevalence | Mali | Female | Glaucoma | 2000 | 2005 | 0.037192561 | -1.66  (-1.76, -1.51) |
| Prevalence | Mali | Female | Glaucoma | 2005 | 2015 | 0.0079984 | -0.32  (-0.6, -0.26) |
| Prevalence | Mali | Female | Glaucoma | 2015 | 2019 | 0.0039992 | -1.22  (-1.5, -0.29) |
| Prevalence | Israel | Male | Glaucoma | 2007 | 2021 | 0 | -0.49  (-0.52, -0.46) |
| Prevalence | Mali | Male | Glaucoma | 1990 | 1993 | 0 | 2.01  (1.75, 2.38) |
| Prevalence | Mali | Male | Glaucoma | 1993 | 1998 | 0 | 1.09  (0.96, 1.26) |
| Prevalence | Mali | Male | Glaucoma | 1998 | 2001 | 0.027194561 | 0.23  (0.04, 0.48) |
| Prevalence | Mali | Male | Glaucoma | 2001 | 2004 | 0 | -1.91  (-2.07, -1.71) |
| Prevalence | New Zealand | Female | Glaucoma | 2018 | 2021 | 0 | -0.49  (-0.58, -0.31) |
| Prevalence | Malta | Both | Glaucoma | 1990 | 1999 | 0 | -1.39  (-1.55, -1.19) |
| Prevalence | Malta | Both | Glaucoma | 1999 | 2004 | 0 | -1.25  (-1.48, -1.03) |
| Prevalence | Malta | Both | Glaucoma | 2004 | 2009 | 0 | -0.95  (-1.23, -0.77) |
| Prevalence | Malta | Both | Glaucoma | 2009 | 2015 | 0 | -0.68  (-0.93, -0.49) |
| Prevalence | Malta | Both | Glaucoma | 2015 | 2019 | 0 | -1.05  (-1.24, -0.7) |
| Prevalence | United States Virgin Islands | Both | Glaucoma | 2015 | 2021 | 0 | -0.51  (-0.54, -0.47) |
| Prevalence | Malta | Female | Glaucoma | 1990 | 2005 | 0 | -1.34  (-1.39, -1.31) |
| Prevalence | Malta | Female | Glaucoma | 2005 | 2015 | 0 | -0.83  (-1.35, -0.6) |
| Prevalence | Somalia | Female | Glaucoma | 2019 | 2021 | 0 | -0.48  (-0.65, -0.3) |
| Prevalence | Malta | Male | Glaucoma | 1990 | 1999 | 0 | -1.4  (-1.43, -1.38) |
| Prevalence | Malta | Male | Glaucoma | 1999 | 2004 | 0 | -1.24  (-1.32, -1.14) |
| Prevalence | Malta | Male | Glaucoma | 2004 | 2009 | 0 | -0.93  (-1.01, -0.85) |
| Prevalence | Malta | Male | Glaucoma | 2009 | 2015 | 0 | -0.65  (-0.69, -0.59) |
| Prevalence | Malta | Male | Glaucoma | 2015 | 2019 | 0 | -0.99  (-1.08, -0.93) |
| Prevalence | United Kingdom | Female | Glaucoma | 2015 | 2021 | 0.00039992 | -0.48  (-0.55, -0.37) |
| Prevalence | Marshall Islands | Both | Glaucoma | 1990 | 1994 | 0 | -1.05  (-1.6, -0.78) |
| Prevalence | Marshall Islands | Both | Glaucoma | 1994 | 2002 | 0.01239752 | -0.52  (-0.61, -0.09) |
| Prevalence | Marshall Islands | Both | Glaucoma | 2002 | 2009 | 0 | -0.81  (-1.1, -0.69) |
| Prevalence | Marshall Islands | Both | Glaucoma | 2009 | 2015 | 0 | -1.44  (-1.87, -1.25) |
| Prevalence | Solomon Islands | Both | Glaucoma | 2015 | 2021 | 0.039992002 | -0.51  (-0.71, -0.05) |
| Prevalence | Marshall Islands | Female | Glaucoma | 1990 | 2009 | 0 | -0.52  (-0.59, -0.43) |
| Prevalence | Marshall Islands | Female | Glaucoma | 2009 | 2015 | 0 | -1.61  (-2.75, -1.19) |
| Prevalence | Cameroon | Male | Glaucoma | 2004 | 2021 | 0 | -0.48  (-0.55, -0.41) |
| Prevalence | Marshall Islands | Male | Glaucoma | 1990 | 1994 | 0 | -1.22  (-1.31, -1.15) |
| Prevalence | Marshall Islands | Male | Glaucoma | 1994 | 2002 | 0 | -0.94  (-0.96, -0.87) |
| Prevalence | Marshall Islands | Male | Glaucoma | 2002 | 2007 | 0 | -1.08  (-1.19, -1.01) |
| Prevalence | Marshall Islands | Male | Glaucoma | 2007 | 2014 | 0 | -1.33  (-1.38, -1.29) |
| Prevalence | Marshall Islands | Male | Glaucoma | 2014 | 2018 | 0 | -1.64  (-1.74, -1.57) |
| Prevalence | Netherlands | Male | Glaucoma | 2011 | 2021 | 0 | -0.47  (-0.49, -0.45) |
| Prevalence | Mauritania | Both | Glaucoma | 1990 | 1995 | 0 | -0.11  (-0.14, -0.08) |
| Prevalence | Mauritania | Both | Glaucoma | 1995 | 2006 | 0 | -0.36  (-0.37, -0.35) |
| Prevalence | Mauritania | Both | Glaucoma | 2006 | 2010 | 0 | -0.65  (-0.67, -0.59) |
| Prevalence | Mauritania | Both | Glaucoma | 2010 | 2015 | 0 | -1.09  (-1.12, -1.06) |
| Prevalence | Mauritania | Both | Glaucoma | 2015 | 2019 | 0 | -0.89  (-0.93, -0.83) |
| Prevalence | Mauritius | Both | Glaucoma | 2018 | 2021 | 0 | -0.5  (-0.61, -0.32) |
| Prevalence | Mauritania | Female | Glaucoma | 1990 | 1993 | 0.00039992 | 0.08  (0.04, 0.15) |
| Prevalence | Mauritania | Female | Glaucoma | 1993 | 1996 | 0.062787443 | -0.03  (-0.25, 0) |
| Prevalence | Mauritania | Female | Glaucoma | 1996 | 2001 | 0 | -0.27  (-0.41, -0.24) |
| Prevalence | Mauritania | Female | Glaucoma | 2001 | 2006 | 0 | -0.42  (-0.6, -0.4) |
| Prevalence | Mauritania | Female | Glaucoma | 2006 | 2010 | 0 | -0.61  (-0.89, -0.59) |
| Prevalence | Uzbekistan | Male | Glaucoma | 2018 | 2021 | 0 | -0.46  (-0.57, -0.33) |
| Prevalence | Mauritania | Male | Glaucoma | 1990 | 1995 | 0 | -0.42  (-0.46, -0.38) |
| Prevalence | Mauritania | Male | Glaucoma | 1995 | 2000 | 0 | -0.63  (-0.71, -0.59) |
| Prevalence | Mauritania | Male | Glaucoma | 2000 | 2006 | 0 | -0.43  (-0.46, -0.38) |
| Prevalence | Mauritania | Male | Glaucoma | 2006 | 2010 | 0 | -0.76  (-0.8, -0.71) |
| Prevalence | Mauritania | Male | Glaucoma | 2010 | 2015 | 0 | -1.24  (-1.29, -1.21) |
| Prevalence | Oman | Male | Glaucoma | 2014 | 2021 | 0 | -0.46  (-0.49, -0.43) |
| Prevalence | Mauritius | Both | Glaucoma | 1990 | 1994 | 0 | -1.29  (-1.44, -1.22) |
| Prevalence | Mauritius | Both | Glaucoma | 1994 | 1999 | 0 | -1.12  (-1.18, -0.86) |
| Prevalence | Mauritius | Both | Glaucoma | 1999 | 2005 | 0 | -0.82  (-0.94, -0.7) |
| Prevalence | Mauritius | Both | Glaucoma | 2005 | 2010 | 0 | -1.05  (-1.17, -0.96) |
| Prevalence | Mauritius | Both | Glaucoma | 2010 | 2018 | 0 | -0.8  (-0.85, -0.77) |
| Prevalence | Uzbekistan | Both | Glaucoma | 2019 | 2021 | 0 | -0.49  (-0.61, -0.41) |
| Prevalence | Mauritius | Female | Glaucoma | 1990 | 1998 | 0 | -1.18  (-1.29, -1.1) |
| Prevalence | Albania | Male | Glaucoma | 2015 | 2021 | 0 | -0.46  (-0.51, -0.38) |
| Prevalence | Mauritius | Male | Glaucoma | 1990 | 1994 | 0 | -1.4  (-1.47, -1.35) |
| Prevalence | Mauritius | Male | Glaucoma | 1994 | 1999 | 0 | -1.14  (-1.19, -1.09) |
| Prevalence | Mauritius | Male | Glaucoma | 1999 | 2005 | 0 | -0.96  (-0.99, -0.9) |
| Prevalence | Mauritius | Male | Glaucoma | 2005 | 2011 | 0 | -1.15  (-1.2, -1.12) |
| Prevalence | Mauritius | Male | Glaucoma | 2011 | 2016 | 0 | -0.85  (-0.9, -0.81) |
| Prevalence | Suriname | Female | Glaucoma | 2014 | 2021 | 0 | -0.46  (-0.53, -0.35) |
| Prevalence | Mexico | Both | Glaucoma | 1990 | 1995 | 0 | -1.5  (-1.62, -1.42) |
| Prevalence | Mexico | Both | Glaucoma | 1995 | 2004 | 0 | -1.15  (-1.19, -1.12) |
| Prevalence | Mexico | Both | Glaucoma | 2004 | 2016 | 0 | -0.69  (-0.7, -0.64) |
| Prevalence | Mexico | Both | Glaucoma | 2016 | 2019 | 0 | -0.89  (-0.99, -0.74) |
| Prevalence | Bulgaria | Both | Glaucoma | 2014 | 2021 | 0 | -0.49  (-0.57, -0.37) |
| Prevalence | Mexico | Female | Glaucoma | 1990 | 2000 | 0 | -1.07  (-1.09, -1.06) |
| Prevalence | Mexico | Female | Glaucoma | 2000 | 2004 | 0 | -1.31  (-1.4, -1.25) |
| Prevalence | Mexico | Female | Glaucoma | 2004 | 2007 | 0 | -0.92  (-1.09, -0.78) |
| Prevalence | Mexico | Female | Glaucoma | 2007 | 2015 | 0 | -0.66  (-0.68, -0.63) |
| Prevalence | Mexico | Female | Glaucoma | 2015 | 2019 | 0 | -1.07  (-1.12, -1.03) |
| Prevalence | Portugal | Male | Glaucoma | 2018 | 2021 | 0 | -0.46  (-0.58, -0.22) |
| Prevalence | Mexico | Male | Glaucoma | 1990 | 1994 | 0 | -1.85  (-2.25, -1.66) |
| Prevalence | Mexico | Male | Glaucoma | 1994 | 1999 | 0 | -1.35  (-1.6, -1.04) |
| Prevalence | Mexico | Male | Glaucoma | 1999 | 2003 | 0 | -0.95  (-1.16, -0.47) |
| Prevalence | Mexico | Male | Glaucoma | 2003 | 2019 | 0 | -0.63  (-0.88, -0.57) |
| Prevalence | Italy | Male | Glaucoma | 2008 | 2021 | 0 | -0.46  (-0.5, -0.4) |
| Prevalence | Micronesia (Federated States of) | Both | Glaucoma | 1990 | 2010 | 0.017996401 | -0.96  (-0.99, -0.76) |
| Prevalence | Micronesia (Federated States of) | Both | Glaucoma | 2010 | 2014 | 0.00359928 | -1.36  (-1.78, -1) |
| Prevalence | New Zealand | Both | Glaucoma | 2018 | 2021 | 0 | -0.48  (-0.54, -0.37) |
| Prevalence | Micronesia (Federated States of) | Female | Glaucoma | 1990 | 2010 | 0.00279944 | -0.67  (-0.73, -0.59) |
| Prevalence | Micronesia (Federated States of) | Female | Glaucoma | 2010 | 2014 | 0 | -1.73  (-2.47, -1.1) |
| Prevalence | Poland | Male | Glaucoma | 2019 | 2021 | 0 | -0.45  (-0.63, -0.19) |
| Prevalence | Micronesia (Federated States of) | Male | Glaucoma | 1990 | 2000 | 0 | -1.12  (-1.13, -1.11) |
| Prevalence | Micronesia (Federated States of) | Male | Glaucoma | 2000 | 2004 | 0 | -1.33  (-1.39, -1.28) |
| Prevalence | Micronesia (Federated States of) | Male | Glaucoma | 2004 | 2010 | 0 | -1.14  (-1.17, -1.1) |
| Prevalence | Micronesia (Federated States of) | Male | Glaucoma | 2010 | 2015 | 0 | -0.89  (-0.92, -0.82) |
| Prevalence | Bahrain | Female | Glaucoma | 2018 | 2021 | 0 | -0.45  (-0.51, -0.39) |
| Prevalence | Monaco | Both | Glaucoma | 1990 | 2005 | 0 | -0.67  (-0.71, -0.63) |
| Prevalence | Monaco | Both | Glaucoma | 2005 | 2016 | 0 | -0.37  (-0.74, -0.32) |
| Prevalence | Monaco | Both | Glaucoma | 2016 | 2019 | 0 | -0.63  (-0.74, -0.25) |
| Prevalence | Costa Rica | Both | Glaucoma | 2018 | 2021 | 0 | -0.48  (-0.55, -0.38) |
| Prevalence | Monaco | Female | Glaucoma | 1990 | 2005 | 0 | -0.67  (-0.71, -0.62) |
| Prevalence | Monaco | Female | Glaucoma | 2005 | 2015 | 0 | -0.41  (-0.73, -0.32) |
| Prevalence | Monaco | Female | Glaucoma | 2015 | 2019 | 0 | -0.69  (-0.89, -0.28) |
| Prevalence | Mali | Female | Glaucoma | 2019 | 2021 | 0.015996801 | -0.45  (-1.04, -0.11) |
| Prevalence | Monaco | Male | Glaucoma | 1990 | 1996 | 0 | -0.65  (-0.77, -0.54) |
| Prevalence | Monaco | Male | Glaucoma | 1996 | 2005 | 0 | -0.75  (-0.87, -0.57) |
| Prevalence | Monaco | Male | Glaucoma | 2005 | 2009 | 0 | -0.43  (-0.77, -0.36) |
| Prevalence | Monaco | Male | Glaucoma | 2009 | 2015 | 0 | -0.29  (-0.45, -0.17) |
| Prevalence | Monaco | Male | Glaucoma | 2015 | 2019 | 0 | -0.5  (-0.62, -0.25) |
| Prevalence | Bulgaria | Male | Glaucoma | 2014 | 2021 | 0 | -0.45  (-0.52, -0.36) |
| Prevalence | Mongolia | Both | Glaucoma | 1990 | 1994 | 0 | -1.8  (-2.07, -1.64) |
| Prevalence | Mongolia | Both | Glaucoma | 1994 | 2000 | 0 | -0.74  (-0.83, -0.63) |
| Prevalence | Mongolia | Both | Glaucoma | 2000 | 2005 | 0 | -2.29  (-2.37, -2.2) |
| Prevalence | Mongolia | Both | Glaucoma | 2005 | 2013 | 0 | -0.7  (-0.78, -0.64) |
| Prevalence | Mongolia | Both | Glaucoma | 2013 | 2019 | 0 | -1.41  (-1.63, -1.28) |
| Prevalence | Kazakhstan | Both | Glaucoma | 2017 | 2021 | 0.00159968 | -0.48  (-0.61, -0.23) |
| Prevalence | Mongolia | Female | Glaucoma | 1990 | 1994 | 0 | -1.44  (-1.81, -1.22) |
| Prevalence | Mongolia | Female | Glaucoma | 1994 | 2001 | 0.00159968 | -0.69  (-0.78, -0.54) |
| Prevalence | Mongolia | Female | Glaucoma | 2001 | 2004 | 0 | -2.34  (-2.52, -2) |
| Prevalence | Mongolia | Female | Glaucoma | 2004 | 2011 | 0.00159968 | -0.37  (-0.47, -0.24) |
| Prevalence | Belgium | Female | Glaucoma | 2019 | 2021 | 0 | -0.44  (-0.72, -0.32) |
| Prevalence | Mongolia | Male | Glaucoma | 1990 | 1994 | 0 | -1.77  (-2.06, -1.58) |
| Prevalence | Mongolia | Male | Glaucoma | 1994 | 2000 | 0 | -0.74  (-0.83, -0.61) |
| Prevalence | Mongolia | Male | Glaucoma | 2000 | 2005 | 0 | -2.7  (-2.79, -2.61) |
| Prevalence | Mongolia | Male | Glaucoma | 2005 | 2014 | 0 | -1.11  (-1.16, -1.05) |
| Prevalence | Mongolia | Male | Glaucoma | 2014 | 2018 | 0 | -2.04  (-2.29, -1.82) |
| Prevalence | Andorra | Female | Glaucoma | 2006 | 2021 | 0 | -0.44  (-0.46, -0.43) |
| Prevalence | Montenegro | Both | Glaucoma | 1990 | 1993 | 0 | 0.49  (0.39, 0.61) |
| Prevalence | Montenegro | Both | Glaucoma | 1993 | 1996 | 0.192761448 | 0.09  (-0.13, 0.23) |
| Prevalence | Montenegro | Both | Glaucoma | 1996 | 2001 | 0 | -0.19  (-1.03, -0.15) |
| Prevalence | Montenegro | Both | Glaucoma | 2001 | 2009 | 0 | -1.04  (-1.08, -1) |
| Prevalence | Montenegro | Both | Glaucoma | 2009 | 2017 | 0 | -0.72  (-0.76, -0.68) |
| Prevalence | Congo | Both | Glaucoma | 2014 | 2021 | 0.0039992 | -0.47  (-0.56, -0.26) |
| Prevalence | Montenegro | Female | Glaucoma | 1990 | 1998 | 0 | 0.19  (0.17, 0.23) |
| Prevalence | Montenegro | Female | Glaucoma | 1998 | 2001 | 0 | -0.21  (-0.28, -0.11) |
| Prevalence | Montenegro | Female | Glaucoma | 2001 | 2010 | 0 | -1.11  (-1.14, -1.09) |
| Prevalence | Montenegro | Female | Glaucoma | 2010 | 2018 | 0 | -0.81  (-0.85, -0.77) |
| Prevalence | Croatia | Male | Glaucoma | 2014 | 2021 | 0 | -0.44  (-0.49, -0.39) |
| Prevalence | Montenegro | Male | Glaucoma | 1990 | 1993 | 0 | 0.87  (0.76, 1.03) |
| Prevalence | Montenegro | Male | Glaucoma | 1993 | 1996 | 0.052389522 | 0.18  (0, 0.33) |
| Prevalence | Montenegro | Male | Glaucoma | 1996 | 2001 | 0 | -0.41  (-0.49, -0.36) |
| Prevalence | Montenegro | Male | Glaucoma | 2001 | 2008 | 0 | -1.11  (-1.19, -1.07) |
| Prevalence | Montenegro | Male | Glaucoma | 2008 | 2014 | 0 | -0.81  (-0.9, -0.69) |
| Prevalence | Equatorial Guinea | Male | Glaucoma | 2018 | 2021 | 0.079984003 | -0.44  (-0.74, 0.06) |
| Prevalence | Morocco | Both | Glaucoma | 1990 | 1998 | 0 | -0.83  (-0.95, -0.8) |
| Prevalence | Morocco | Both | Glaucoma | 1998 | 2001 | 0 | -0.7  (-1.34, -0.63) |
| Prevalence | Morocco | Both | Glaucoma | 2001 | 2004 | 0 | -1.44  (-1.54, -0.96) |
| Prevalence | Morocco | Both | Glaucoma | 2004 | 2007 | 0 | -0.93  (-1.29, -0.83) |
| Prevalence | Morocco | Both | Glaucoma | 2007 | 2018 | 0 | -1.31  (-1.44, -1.28) |
| Prevalence | Togo | Both | Glaucoma | 2010 | 2021 | 0 | -0.46  (-0.48, -0.43) |
| Prevalence | Morocco | Female | Glaucoma | 1990 | 2001 | 0 | -0.72  (-0.77, -0.68) |
| Prevalence | Morocco | Female | Glaucoma | 2001 | 2004 | 0 | -1.73  (-1.83, -0.67) |
| Prevalence | Morocco | Female | Glaucoma | 2004 | 2007 | 0 | -0.98  (-1.74, -0.84) |
| Prevalence | Morocco | Female | Glaucoma | 2007 | 2010 | 0 | -1.45  (-1.57, -1.05) |
| Prevalence | Morocco | Female | Glaucoma | 2010 | 2018 | 0 | -1.22  (-1.32, -1.07) |
| Prevalence | Montenegro | Male | Glaucoma | 2014 | 2021 | 0 | -0.44  (-0.48, -0.36) |
| Prevalence | Morocco | Male | Glaucoma | 1990 | 1998 | 0 | -0.9  (-0.95, -0.87) |
| Prevalence | Morocco | Male | Glaucoma | 1998 | 2001 | 0 | -0.63  (-0.73, -0.56) |
| Prevalence | Morocco | Male | Glaucoma | 2001 | 2004 | 0 | -1.17  (-1.26, -1.06) |
| Prevalence | Morocco | Male | Glaucoma | 2004 | 2007 | 0 | -0.75  (-0.92, -0.66) |
| Prevalence | Japan | Male | Glaucoma | 2017 | 2021 | 0 | -0.43  (-0.59, -0.32) |
| Prevalence | Mozambique | Both | Glaucoma | 1990 | 1996 | 0 | -1.15  (-1.41, -1.04) |
| Prevalence | Mozambique | Both | Glaucoma | 1996 | 1999 | 0.100779844 | -0.44  (-0.9, 0.08) |
| Prevalence | Mozambique | Both | Glaucoma | 1999 | 2005 | 0.111177764 | 0.14  (-0.63, 0.35) |
| Prevalence | Mozambique | Both | Glaucoma | 2005 | 2014 | 0 | -0.64  (-0.76, -0.55) |
| Prevalence | Samoa | Both | Glaucoma | 2015 | 2021 | 0.062387522 | -0.45  (-0.64, 0.03) |
| Prevalence | Mozambique | Female | Glaucoma | 1990 | 1995 | 0 | -1.86  (-2.03, -1.72) |
| Prevalence | Mozambique | Female | Glaucoma | 1995 | 1998 | 0.078784243 | -0.25  (-0.99, 0.17) |
| Prevalence | Mozambique | Female | Glaucoma | 1998 | 2004 | 0.00439912 | 0.9  (0.82, 1.03) |
| Prevalence | Mozambique | Female | Glaucoma | 2004 | 2015 | 0.0059988 | -0.37  (-0.41, -0.33) |
| Prevalence | Mozambique | Female | Glaucoma | 2015 | 2019 | 0.00039992 | -1.28  (-1.51, -1.08) |
| Prevalence | Finland | Male | Glaucoma | 2019 | 2021 | 0 | -0.43  (-0.67, -0.23) |
| Prevalence | Mozambique | Male | Glaucoma | 1990 | 1995 | 0.048390322 | -0.44  (-0.67, -0.01) |
| Prevalence | Mozambique | Male | Glaucoma | 1995 | 1999 | 0.01039792 | -0.96  (-1.29, -0.28) |
| Prevalence | Mozambique | Male | Glaucoma | 1999 | 2006 | 0.092381524 | -0.31  (-0.95, 0.07) |
| Prevalence | Russian Federation | Female | Glaucoma | 2019 | 2021 | 0 | -0.42  (-0.78, -0.22) |
| Prevalence | Myanmar | Both | Glaucoma | 1990 | 1993 | 0 | 2.75  (2.34, 3.23) |
| Prevalence | Myanmar | Both | Glaucoma | 1993 | 1996 | 0.116376725 | 0.38  (-0.15, 0.73) |
| Prevalence | Myanmar | Both | Glaucoma | 1996 | 2008 | 0 | -1.04  (-1.1, -0.98) |
| Prevalence | Myanmar | Both | Glaucoma | 2008 | 2011 | 0 | -2.16  (-2.38, -1.8) |
| Prevalence | Myanmar | Both | Glaucoma | 2011 | 2014 | 0 | -6.48  (-6.73, -6.25) |
| Prevalence | United Arab Emirates | Both | Glaucoma | 2018 | 2021 | 0 | -0.44  (-0.58, -0.28) |
| Prevalence | Myanmar | Female | Glaucoma | 1990 | 1992 | 0 | 3.97  (3, 4.8) |
| Prevalence | Myanmar | Female | Glaucoma | 1992 | 1995 | 0.055588882 | 1.56  (-0.36, 1.91) |
| Prevalence | Myanmar | Female | Glaucoma | 1995 | 2008 | 0 | -0.97  (-1.04, -0.89) |
| Prevalence | Myanmar | Female | Glaucoma | 2008 | 2011 | 0 | -2.03  (-2.34, -1.58) |
| Prevalence | Myanmar | Female | Glaucoma | 2011 | 2014 | 0 | -7.41  (-7.75, -7.09) |
| Prevalence | Singapore | Male | Glaucoma | 2012 | 2021 | 0 | -0.42  (-0.45, -0.36) |
| Prevalence | Myanmar | Male | Glaucoma | 1990 | 1993 | 0 | 1.97  (1.62, 2.53) |
| Prevalence | Myanmar | Male | Glaucoma | 1993 | 1996 | 0.125974805 | 0.42  (-0.3, 0.74) |
| Prevalence | Myanmar | Male | Glaucoma | 1996 | 2008 | 0 | -1.09  (-1.15, -1.04) |
| Prevalence | Myanmar | Male | Glaucoma | 2008 | 2011 | 0 | -2.31  (-2.54, -1.94) |
| Prevalence | Myanmar | Male | Glaucoma | 2011 | 2014 | 0 | -5.25  (-5.49, -4.99) |
| Prevalence | Kenya | Female | Glaucoma | 2014 | 2021 | 0.067186563 | -0.4  (-0.59, 0.05) |
| Prevalence | Namibia | Both | Glaucoma | 1990 | 2002 | 0 | -0.64  (-0.67, -0.62) |
| Prevalence | Namibia | Both | Glaucoma | 2002 | 2006 | 0 | -0.92  (-1.01, -0.63) |
| Prevalence | Namibia | Both | Glaucoma | 2006 | 2010 | 0 | -1.2  (-1.31, -0.95) |
| Prevalence | Namibia | Both | Glaucoma | 2010 | 2015 | 0 | -1.52  (-1.64, -1.23) |
| Prevalence | Namibia | Both | Glaucoma | 2015 | 2019 | 0 | -1.29  (-1.47, -1.19) |
| Prevalence | Maldives | Both | Glaucoma | 2018 | 2021 | 0.076384723 | -0.44  (-0.71, 0.08) |
| Prevalence | Namibia | Female | Glaucoma | 1990 | 1994 | 0 | -0.77  (-0.88, -0.7) |
| Prevalence | Namibia | Female | Glaucoma | 1994 | 2002 | 0 | -0.5  (-0.53, -0.45) |
| Prevalence | Namibia | Female | Glaucoma | 2002 | 2006 | 0 | -0.78  (-0.87, -0.68) |
| Prevalence | Namibia | Female | Glaucoma | 2006 | 2010 | 0 | -1.13  (-1.22, -1.03) |
| Prevalence | Namibia | Female | Glaucoma | 2010 | 2017 | 0 | -1.4  (-1.43, -1.37) |
| Prevalence | Uruguay | Male | Glaucoma | 2018 | 2021 | 0 | -0.4  (-0.5, -0.23) |
| Prevalence | Namibia | Male | Glaucoma | 1990 | 2001 | 0.00119976 | -0.66  (-0.71, -0.59) |
| Prevalence | Namibia | Male | Glaucoma | 2001 | 2009 | 0 | -1.01  (-1.12, -0.9) |
| Prevalence | Namibia | Male | Glaucoma | 2009 | 2019 | 0 | -1.47  (-1.68, -1.27) |
| Prevalence | Democratic Republic of the Congo | Female | Glaucoma | 2015 | 2021 | 0 | -0.39  (-0.44, -0.34) |
| Prevalence | Nauru | Both | Glaucoma | 1990 | 2005 | 0.015996801 | -0.33  (-0.38, -0.24) |
| Prevalence | Nauru | Both | Glaucoma | 2005 | 2010 | 0.00079984 | -0.87  (-1.31, -0.54) |
| Prevalence | Nauru | Both | Glaucoma | 2010 | 2015 | 0 | -2.07  (-2.64, -1.78) |
| Prevalence | France | Both | Glaucoma | 2018 | 2021 | 0.00119976 | -0.44  (-0.6, -0.19) |
| Prevalence | Nauru | Female | Glaucoma | 1990 | 2009 | 0.960607878 | 0  (-0.07, 0.07) |
| Prevalence | Nauru | Female | Glaucoma | 2009 | 2015 | 0 | -2.07  (-2.93, -1.66) |
| Prevalence | Azerbaijan | Male | Glaucoma | 2017 | 2021 | 0.035992801 | -0.39  (-0.6, -0.05) |
| Prevalence | Nauru | Male | Glaucoma | 1990 | 2004 | 0.00919816 | -0.21  (-0.23, -0.2) |
| Prevalence | Nauru | Male | Glaucoma | 2004 | 2007 | 0 | -0.7  (-0.8, -0.21) |
| Prevalence | Nauru | Male | Glaucoma | 2007 | 2011 | 0.00039992 | -1.15  (-1.23, -0.74) |
| Prevalence | Nauru | Male | Glaucoma | 2011 | 2014 | 0 | -1.74  (-1.84, -1.24) |
| Prevalence | Nauru | Male | Glaucoma | 2014 | 2019 | 0 | -1.98  (-2.12, -1.93) |
| Prevalence | France | Female | Glaucoma | 2018 | 2021 | 0 | -0.39  (-0.5, -0.23) |
| Prevalence | Nepal | Both | Glaucoma | 1990 | 1994 | 0 | -1.92  (-2.54, -1.58) |
| Prevalence | Nepal | Both | Glaucoma | 1994 | 2001 | 0.00239952 | -0.48  (-0.61, -0.27) |
| Prevalence | Nepal | Both | Glaucoma | 2001 | 2004 | 0.00039992 | -3.03  (-3.25, -2.62) |
| Prevalence | Nepal | Both | Glaucoma | 2004 | 2015 | 0 | -1.02  (-1.08, -0.95) |
| Prevalence | Nepal | Both | Glaucoma | 2015 | 2019 | 0 | -2.12  (-2.36, -1.81) |
| Prevalence | Netherlands | Both | Glaucoma | 2014 | 2021 | 0.00319936 | -0.43  (-0.5, -0.25) |
| Prevalence | Nepal | Female | Glaucoma | 1990 | 1995 | 0.0019996 | -0.92  (-1.74, -0.53) |
| Prevalence | Nepal | Female | Glaucoma | 1995 | 2000 | 0.00639872 | 0.78  (0.4, 1.43) |
| Prevalence | Nepal | Female | Glaucoma | 2000 | 2005 | 0.00679864 | -2.75  (-3.23, -2.45) |
| Prevalence | Nepal | Female | Glaucoma | 2005 | 2015 | 0.00319936 | -0.85  (-0.97, -0.65) |
| Prevalence | Nepal | Female | Glaucoma | 2015 | 2019 | 0 | -2.02  (-2.36, -1.28) |
| Prevalence | Ireland | Female | Glaucoma | 2019 | 2021 | 0.023995201 | -0.39  (-0.96, -0.08) |
| Prevalence | Nepal | Male | Glaucoma | 1990 | 1995 | 0 | -2.32  (-2.64, -2.13) |
| Prevalence | Nepal | Male | Glaucoma | 1995 | 2001 | 0 | -1  (-1.11, -0.78) |
| Prevalence | Nepal | Male | Glaucoma | 2001 | 2004 | 0 | -2.49  (-2.66, -2.14) |
| Prevalence | Nepal | Male | Glaucoma | 2004 | 2015 | 0 | -1.09  (-1.13, -1.03) |
| Prevalence | Nepal | Male | Glaucoma | 2015 | 2019 | 0 | -2.16  (-2.3, -1.91) |
| Prevalence | Belgium | Male | Glaucoma | 2018 | 2021 | 0 | -0.39  (-0.51, -0.19) |
| Prevalence | Netherlands | Both | Glaucoma | 1990 | 1999 | 0 | -1.06  (-1.1, -1.02) |
| Prevalence | Netherlands | Both | Glaucoma | 1999 | 2006 | 0.00159968 | -0.36  (-0.42, -0.26) |
| Prevalence | Netherlands | Both | Glaucoma | 2006 | 2014 | 0 | -0.63  (-0.82, -0.58) |
| Prevalence | Palau | Both | Glaucoma | 2015 | 2021 | 0.065186963 | -0.43  (-0.61, 0.07) |
| Prevalence | Netherlands | Female | Glaucoma | 1990 | 1999 | 0 | -1  (-1.09, -0.94) |
| Prevalence | Netherlands | Female | Glaucoma | 1999 | 2007 | 0.00919816 | -0.36  (-0.88, -0.21) |
| Prevalence | Netherlands | Female | Glaucoma | 2007 | 2019 | 0.00159968 | -0.62  (-0.82, -0.33) |
| Prevalence | Luxembourg | Male | Glaucoma | 2019 | 2021 | 0 | -0.39  (-0.53, -0.28) |
| Prevalence | Netherlands | Male | Glaucoma | 1990 | 1999 | 0 | -1.12  (-1.14, -1.1) |
| Prevalence | Netherlands | Male | Glaucoma | 1999 | 2006 | 0 | -0.46  (-0.48, -0.43) |
| Prevalence | Netherlands | Male | Glaucoma | 2006 | 2011 | 0 | -0.88  (-0.96, -0.82) |
| Prevalence | Seychelles | Male | Glaucoma | 2019 | 2021 | 0 | -0.39  (-0.49, -0.3) |
| Prevalence | New Zealand | Both | Glaucoma | 1990 | 1995 | 0 | -0.96  (-1, -0.92) |
| Prevalence | New Zealand | Both | Glaucoma | 1995 | 2004 | 0 | -0.73  (-0.75, -0.71) |
| Prevalence | New Zealand | Both | Glaucoma | 2004 | 2015 | 0 | -0.58  (-0.59, -0.56) |
| Prevalence | New Zealand | Both | Glaucoma | 2015 | 2018 | 0 | -0.74  (-0.78, -0.67) |
| Prevalence | Chile | Both | Glaucoma | 2018 | 2021 | 0 | -0.43  (-0.49, -0.36) |
| Prevalence | New Zealand | Female | Glaucoma | 1990 | 1995 | 0 | -0.98  (-1.06, -0.92) |
| Prevalence | New Zealand | Female | Glaucoma | 1995 | 2010 | 0 | -0.69  (-0.7, -0.68) |
| Prevalence | New Zealand | Female | Glaucoma | 2010 | 2015 | 0 | -0.46  (-0.52, -0.32) |
| Prevalence | New Zealand | Female | Glaucoma | 2015 | 2018 | 0 | -0.85  (-0.93, -0.73) |
| Prevalence | Cuba | Female | Glaucoma | 2019 | 2021 | 0 | -0.39  (-0.67, -0.24) |
| Prevalence | New Zealand | Male | Glaucoma | 1990 | 1994 | 0 | -1.02  (-1.1, -0.97) |
| Prevalence | New Zealand | Male | Glaucoma | 1994 | 2005 | 0 | -0.83  (-0.84, -0.82) |
| Prevalence | New Zealand | Male | Glaucoma | 2005 | 2010 | 0 | -0.41  (-0.44, -0.38) |
| Prevalence | New Zealand | Male | Glaucoma | 2010 | 2015 | 0 | -0.81  (-0.86, -0.78) |
| Prevalence | United Republic of Tanzania | Male | Glaucoma | 2005 | 2021 | 0 | -0.38  (-0.5, -0.28) |
| Prevalence | Nicaragua | Both | Glaucoma | 1990 | 1995 | 0 | -1.18  (-1.2, -1.14) |
| Prevalence | Nicaragua | Both | Glaucoma | 1995 | 2000 | 0 | -1.28  (-1.33, -1.26) |
| Prevalence | Nicaragua | Both | Glaucoma | 2000 | 2005 | 0 | -1.09  (-1.11, -1.06) |
| Prevalence | Nicaragua | Both | Glaucoma | 2005 | 2011 | 0 | -0.8  (-0.82, -0.78) |
| Prevalence | Nicaragua | Both | Glaucoma | 2011 | 2019 | 0 | -0.92  (-0.93, -0.9) |
| Prevalence | Montenegro | Both | Glaucoma | 2017 | 2021 | 0 | -0.43  (-0.5, -0.3) |
| Prevalence | Nicaragua | Female | Glaucoma | 1990 | 2005 | 0 | -1.17  (-1.18, -1.16) |
| Prevalence | Nicaragua | Female | Glaucoma | 2005 | 2010 | 0 | -0.74  (-0.78, -0.69) |
| Prevalence | Nicaragua | Female | Glaucoma | 2010 | 2019 | 0 | -0.94  (-0.96, -0.92) |
| Prevalence | Kuwait | Male | Glaucoma | 2018 | 2021 | 0 | -0.38  (-0.44, -0.33) |
| Prevalence | Nicaragua | Male | Glaucoma | 1990 | 1995 | 0 | -1.22  (-1.24, -1.2) |
| Prevalence | Nicaragua | Male | Glaucoma | 1995 | 2000 | 0 | -1.43  (-1.45, -1.4) |
| Prevalence | Nicaragua | Male | Glaucoma | 2000 | 2003 | 0 | -1.05  (-1.33, -1.02) |
| Prevalence | Nicaragua | Male | Glaucoma | 2003 | 2006 | 0 | -0.93  (-0.97, -0.82) |
| Prevalence | Nicaragua | Male | Glaucoma | 2006 | 2016 | 0 | -0.81  (-0.82, -0.8) |
| Prevalence | Australia | Male | Glaucoma | 2015 | 2021 | 0.00079984 | -0.37  (-0.44, -0.27) |
| Prevalence | Niger | Both | Glaucoma | 1990 | 1994 | 0 | 0.71  (0.62, 0.77) |
| Prevalence | Niger | Both | Glaucoma | 1994 | 2006 | 0 | 0.13  (0.11, 0.15) |
| Prevalence | Niger | Both | Glaucoma | 2006 | 2009 | 0.00279944 | 0.93  (0.16, 0.99) |
| Prevalence | Niger | Both | Glaucoma | 2009 | 2013 | 0 | 0.43  (0.36, 0.84) |
| Prevalence | Niger | Both | Glaucoma | 2013 | 2016 | 0 | 0.16  (0.09, 0.32) |
| Prevalence | Luxembourg | Both | Glaucoma | 2019 | 2021 | 0 | -0.42  (-0.58, -0.3) |
| Prevalence | Niger | Female | Glaucoma | 1990 | 1993 | 0 | 0.65  (0.47, 0.93) |
| Prevalence | Niger | Female | Glaucoma | 1993 | 2006 | 0 | 0.16  (0.13, 0.19) |
| Prevalence | Niger | Female | Glaucoma | 2006 | 2013 | 0.00359928 | -0.3  (-0.43, -0.24) |
| Prevalence | Niger | Female | Glaucoma | 2013 | 2016 | 0.233953209 | 0.11  (-0.07, 0.22) |
| Prevalence | Niger | Female | Glaucoma | 2016 | 2019 | 0 | 2.59  (2.45, 2.73) |
| Prevalence | Bulgaria | Female | Glaucoma | 2015 | 2021 | 0.00359928 | -0.37  (-0.48, -0.2) |
| Prevalence | Niger | Male | Glaucoma | 1990 | 1994 | 0 | 0.54  (0.42, 0.66) |
| Prevalence | Niger | Male | Glaucoma | 1994 | 2003 | 0.208758248 | 0.05  (-0.06, 0.09) |
| Prevalence | Niger | Male | Glaucoma | 2003 | 2006 | 0.0039992 | 0.27  (0.13, 0.4) |
| Prevalence | Niger | Male | Glaucoma | 2006 | 2009 | 0 | 1.85  (1.62, 1.97) |
| Prevalence | Niger | Male | Glaucoma | 2009 | 2014 | 0.037992402 | 1.02  (0.92, 1.07) |
| Prevalence | Saint Lucia | Female | Glaucoma | 2017 | 2021 | 0 | -0.37  (-0.45, -0.25) |
| Prevalence | Nigeria | Both | Glaucoma | 1990 | 1996 | 0 | -1.18  (-1.31, -1.05) |
| Prevalence | Nigeria | Both | Glaucoma | 1996 | 1999 | 0 | -4.86  (-5.07, -4.64) |
| Prevalence | Nigeria | Both | Glaucoma | 1999 | 2008 | 0 | -0.27  (-0.36, -0.16) |
| Prevalence | Nigeria | Both | Glaucoma | 2008 | 2015 | 0 | -1.05  (-1.3, -0.91) |
| Prevalence | Central African Republic | Both | Glaucoma | 2019 | 2021 | 0 | -0.42  (-0.58, -0.22) |
| Prevalence | Nigeria | Female | Glaucoma | 1990 | 1996 | 0 | -1.8  (-1.94, -1.66) |
| Prevalence | Nigeria | Female | Glaucoma | 1996 | 1999 | 0 | -4.36  (-4.63, -3.71) |
| Prevalence | Nigeria | Female | Glaucoma | 1999 | 2002 | 0.00119976 | -0.63  (-0.99, -0.23) |
| Prevalence | Nigeria | Female | Glaucoma | 2002 | 2007 | 0.940211958 | -0.02  (-1, 0.34) |
| Prevalence | Nigeria | Female | Glaucoma | 2007 | 2015 | 0.043591282 | -0.98  (-1.13, -0.23) |
| Prevalence | Andorra | Male | Glaucoma | 2005 | 2021 | 0 | -0.36  (-0.39, -0.34) |
| Prevalence | Nigeria | Male | Glaucoma | 1990 | 1995 | 0 | -0.65  (-0.83, -0.47) |
| Prevalence | Nigeria | Male | Glaucoma | 1995 | 2000 | 0 | -3.98  (-4.14, -3.82) |
| Prevalence | Nigeria | Male | Glaucoma | 2000 | 2006 | 0.215956809 | -0.11  (-0.28, 0.1) |
| Prevalence | Nigeria | Male | Glaucoma | 2006 | 2016 | 0.00039992 | -0.89  (-1.09, -0.8) |
| Prevalence | Niue | Both | Glaucoma | 1990 | 2005 | 0 | -0.83  (-0.87, -0.78) |
| Prevalence | Niue | Both | Glaucoma | 2005 | 2015 | 0 | -1.25  (-1.42, -1.17) |
| Prevalence | Niue | Both | Glaucoma | 2015 | 2021 | 0.026795 | -0.4  (-0.58, -0.11) |
| Prevalence | Niue | Female | Glaucoma | 1990 | 2008 | 0.0004 | -0.71  (-0.76, -0.63) |
| Prevalence | Niue | Female | Glaucoma | 2008 | 2014 | 0 | -1.47  (-2.38, -1.18) |
| Prevalence | Niue | Female | Glaucoma | 2014 | 2021 | 0.273545 | 0.12  (-0.15, 0.54) |
| Prevalence | Niue | Male | Glaucoma | 1990 | 1996 | 0 | -1.09  (-1.11, -1.03) |
| Prevalence | Niue | Male | Glaucoma | 1996 | 2005 | 0 | -1.16  (-1.19, -1.15) |
| Prevalence | Niue | Male | Glaucoma | 2005 | 2010 | 0 | -1.48  (-1.5, -1.46) |
| Prevalence | Niue | Male | Glaucoma | 2010 | 2015 | 0 | -1.01  (-1.03, -0.98) |
| Prevalence | Niue | Male | Glaucoma | 2015 | 2018 | 0 | -1.39  (-1.43, -1.34) |
| Prevalence | Niue | Male | Glaucoma | 2018 | 2021 | 0 | -0.95  (-1, -0.9) |
| Prevalence | Romania | Male | Glaucoma | 2014 | 2021 | 0 | -0.36  (-0.41, -0.31) |
| Prevalence | North Macedonia | Both | Glaucoma | 1990 | 1995 | 0 | -0.61  (-0.67, -0.54) |
| Prevalence | North Macedonia | Both | Glaucoma | 1995 | 2000 | 0 | -1.07  (-1.14, -0.99) |
| Prevalence | North Macedonia | Both | Glaucoma | 2000 | 2004 | 0 | -1.53  (-1.65, -1.44) |
| Prevalence | North Macedonia | Both | Glaucoma | 2004 | 2010 | 0 | -1.26  (-1.3, -1.18) |
| Prevalence | North Macedonia | Both | Glaucoma | 2010 | 2015 | 0 | -0.77  (-0.85, -0.69) |
| Prevalence | Trinidad and Tobago | Both | Glaucoma | 2017 | 2021 | 0 | -0.41  (-0.5, -0.28) |
| Prevalence | North Macedonia | Female | Glaucoma | 1990 | 2000 | 0 | -0.89  (-0.91, -0.88) |
| Prevalence | North Macedonia | Female | Glaucoma | 2000 | 2005 | 0 | -1.42  (-1.47, -1.18) |
| Prevalence | North Macedonia | Female | Glaucoma | 2005 | 2009 | 0 | -1.15  (-1.38, -1.06) |
| Prevalence | North Macedonia | Female | Glaucoma | 2009 | 2014 | 0 | -0.92  (-1.05, -0.86) |
| Prevalence | North Macedonia | Female | Glaucoma | 2014 | 2018 | 0 | -0.76  (-0.81, -0.68) |
| Prevalence | Burundi | Male | Glaucoma | 2009 | 2021 | 0.016396721 | -0.35  (-0.48, -0.27) |
| Prevalence | North Macedonia | Male | Glaucoma | 1990 | 1993 | 0.096380724 | -0.24  (-0.49, 0.06) |
| Prevalence | North Macedonia | Male | Glaucoma | 1993 | 1996 | 0 | -0.67  (-1.45, -0.55) |
| Prevalence | North Macedonia | Male | Glaucoma | 1996 | 2008 | 0 | -1.39  (-1.44, -0.74) |
| Prevalence | North Macedonia | Male | Glaucoma | 2008 | 2014 | 0.00079984 | -0.73  (-0.85, -0.38) |
| Prevalence | Saint Vincent and the Grenadines | Female | Glaucoma | 2019 | 2021 | 0 | -0.34  (-0.5, -0.23) |
| Prevalence | Northern Mariana Islands | Both | Glaucoma | 1990 | 1999 | 0 | -1.09  (-1.2, -1) |
| Prevalence | Northern Mariana Islands | Both | Glaucoma | 1999 | 2015 | 0.00079984 | -0.26  (-0.29, -0.2) |
| Prevalence | Andorra | Both | Glaucoma | 2006 | 2021 | 0 | -0.4  (-0.42, -0.39) |
| Prevalence | Northern Mariana Islands | Female | Glaucoma | 1990 | 1998 | 0 | -0.95  (-1.2, -0.76) |
| Prevalence | Togo | Female | Glaucoma | 2010 | 2021 | 0 | -0.34  (-0.37, -0.32) |
| Prevalence | Northern Mariana Islands | Male | Glaucoma | 1990 | 1998 | 0 | -1.2  (-1.24, -1.17) |
| Prevalence | Northern Mariana Islands | Male | Glaucoma | 1998 | 2001 | 0 | -0.79  (-1.06, -0.51) |
| Prevalence | Northern Mariana Islands | Male | Glaucoma | 2001 | 2010 | 0 | -0.28  (-0.31, -0.25) |
| Prevalence | Northern Mariana Islands | Male | Glaucoma | 2010 | 2015 | 0.574685063 | 0.01  (-0.04, 0.1) |
| Prevalence | Northern Mariana Islands | Male | Glaucoma | 2015 | 2019 | 0.00079984 | -1.4  (-1.5, -1.35) |
| Prevalence | Bangladesh | Male | Glaucoma | 2019 | 2021 | 0.021995601 | -0.34  (-0.78, -0.06) |
| Prevalence | Norway | Both | Glaucoma | 1990 | 1995 | 0 | -0.78  (-0.84, -0.72) |
| Prevalence | Norway | Both | Glaucoma | 1995 | 2000 | 0 | -1.25  (-1.33, -1.18) |
| Prevalence | Norway | Both | Glaucoma | 2000 | 2005 | 0 | -0.63  (-0.72, -0.57) |
| Prevalence | Norway | Both | Glaucoma | 2005 | 2015 | 0.012797441 | -0.13  (-0.15, -0.09) |
| Prevalence | Norway | Both | Glaucoma | 2015 | 2019 | 0.00119976 | -0.3  (-0.42, -0.22) |
| Prevalence | Barbados | Both | Glaucoma | 2015 | 2021 | 0 | -0.4  (-0.44, -0.3) |
| Prevalence | Norway | Female | Glaucoma | 1990 | 1995 | 0 | -0.57  (-0.67, -0.48) |
| Prevalence | Norway | Female | Glaucoma | 1995 | 2000 | 0 | -1.37  (-1.53, -1.27) |
| Prevalence | Norway | Female | Glaucoma | 2000 | 2004 | 0 | -0.64  (-0.87, -0.41) |
| Prevalence | Norway | Female | Glaucoma | 2004 | 2012 | 0.443111378 | 0.01  (-0.04, 0.09) |
| Prevalence | Norway | Female | Glaucoma | 2012 | 2019 | 0.00119976 | -0.52  (-0.64, -0.46) |
| Prevalence | Finland | Female | Glaucoma | 2019 | 2021 | 0 | -0.33  (-0.54, -0.19) |
| Prevalence | Norway | Male | Glaucoma | 1990 | 2000 | 0 | -1.05  (-1.08, -1.03) |
| Prevalence | Norway | Male | Glaucoma | 2000 | 2004 | 0 | -0.81  (-1.03, -0.72) |
| Prevalence | Norway | Male | Glaucoma | 2004 | 2009 | 0.014797041 | -0.69  (-0.72, -0.05) |
| Prevalence | Central African Republic | Female | Glaucoma | 2019 | 2021 | 0.00919816 | -0.33  (-0.54, -0.05) |
| Prevalence | Oman | Both | Glaucoma | 1990 | 1995 | 0 | -0.69  (-0.8, -0.62) |
| Prevalence | Oman | Both | Glaucoma | 1995 | 2006 | 0 | -0.33  (-0.35, -0.31) |
| Prevalence | Oman | Both | Glaucoma | 2006 | 2014 | 0 | -1.22  (-1.24, -1.19) |
| Prevalence | Oman | Both | Glaucoma | 2014 | 2021 | 0 | -0.4  (-0.43, -0.37) |
| Prevalence | Oman | Female | Glaucoma | 1990 | 1993 | 0.057188562 | -0.19  (-0.48, 0.01) |
| Prevalence | Oman | Female | Glaucoma | 1993 | 2005 | 0.00079984 | 0.24  (0.22, 0.27) |
| Prevalence | Oman | Female | Glaucoma | 2005 | 2014 | 0.00039992 | -0.75  (-0.78, -0.72) |
| Prevalence | Sweden | Female | Glaucoma | 1990 | 2021 | 0 | -0.33  (-0.41, -0.24) |
| Prevalence | Oman | Male | Glaucoma | 1990 | 1994 | 0 | -1.57  (-1.68, -1.5) |
| Prevalence | Oman | Male | Glaucoma | 1994 | 1999 | 0 | -1.06  (-1.15, -0.99) |
| Prevalence | Oman | Male | Glaucoma | 1999 | 2005 | 0 | -0.69  (-0.73, -0.62) |
| Prevalence | Oman | Male | Glaucoma | 2005 | 2009 | 0 | -1.1  (-1.18, -0.99) |
| Prevalence | Oman | Male | Glaucoma | 2009 | 2014 | 0 | -1.36  (-1.42, -1.32) |
| Prevalence | France | Male | Glaucoma | 2018 | 2021 | 0.00959808 | -0.31  (-0.46, -0.12) |
| Prevalence | Pakistan | Both | Glaucoma | 1990 | 1994 | 0 | 1.28  (1.11, 1.47) |
| Prevalence | Pakistan | Both | Glaucoma | 1994 | 2000 | 0.054389122 | -0.19  (-0.29, 0.01) |
| Prevalence | Pakistan | Both | Glaucoma | 2000 | 2005 | 0.014397121 | -0.6  (-0.85, -0.46) |
| Prevalence | Pakistan | Both | Glaucoma | 2005 | 2010 | 0.0119976 | 0.87  (0.74, 1.01) |
| Prevalence | Papua New Guinea | Both | Glaucoma | 2014 | 2021 | 0.056388722 | -0.39  (-0.55, 0.03) |
| Prevalence | Pakistan | Female | Glaucoma | 1990 | 1993 | 0 | 1.79  (1.56, 2.06) |
| Prevalence | Pakistan | Female | Glaucoma | 1993 | 1996 | 0.037992402 | 0.36  (0.05, 0.63) |
| Prevalence | Pakistan | Female | Glaucoma | 1996 | 2005 | 0.00119976 | -0.42  (-0.51, -0.38) |
| Prevalence | Pakistan | Female | Glaucoma | 2005 | 2010 | 0.052789442 | 0.11  (0, 0.28) |
| Prevalence | Pakistan | Female | Glaucoma | 2010 | 2018 | 0 | -1.25  (-1.32, -1.19) |
| Prevalence | Central African Republic | Male | Glaucoma | 2015 | 2021 | 0.00239952 | -0.31  (-0.38, -0.13) |
| Prevalence | Pakistan | Male | Glaucoma | 1990 | 1994 | 0 | 1.06  (0.92, 1.22) |
| Prevalence | Pakistan | Male | Glaucoma | 1994 | 1999 | 0.133173365 | -0.12  (-0.23, 0.07) |
| Prevalence | Pakistan | Male | Glaucoma | 1999 | 2005 | 0.00119976 | -0.53  (-0.63, -0.45) |
| Prevalence | Pakistan | Male | Glaucoma | 2005 | 2011 | 0.00079984 | 1.25  (1.19, 1.32) |
| Prevalence | Congo | Female | Glaucoma | 2015 | 2021 | 0 | -0.3  (-0.38, -0.23) |
| Prevalence | Palau | Both | Glaucoma | 1990 | 1999 | 0.00039992 | -0.97  (-1.15, -0.87) |
| Prevalence | Palau | Both | Glaucoma | 1999 | 2006 | 0.068386323 | -0.42  (-0.56, 0.03) |
| Prevalence | Palau | Both | Glaucoma | 2006 | 2015 | 0 | -0.89  (-1.29, -0.81) |
| Prevalence | Vanuatu | Both | Glaucoma | 2015 | 2021 | 0.01039792 | -0.38  (-0.52, -0.15) |
| Prevalence | Palau | Female | Glaucoma | 1990 | 1998 | 0.00039992 | -0.94  (-1.79, -0.68) |
| Prevalence | Palau | Female | Glaucoma | 1998 | 2009 | 0.24795041 | -0.28  (-0.42, 0.59) |
| Prevalence | Palau | Female | Glaucoma | 2009 | 2015 | 0.0019996 | -1.17  (-2.23, -0.78) |
| Prevalence | North Macedonia | Male | Glaucoma | 2014 | 2021 | 0.00359928 | -0.3  (-0.39, -0.19) |
| Prevalence | Palau | Male | Glaucoma | 1990 | 1994 | 0 | -1.14  (-1.19, -1.06) |
| Prevalence | Palau | Male | Glaucoma | 1994 | 2000 | 0 | -1.22  (-1.27, -0.57) |
| Prevalence | Palau | Male | Glaucoma | 2000 | 2005 | 0 | -0.54  (-0.67, -0.52) |
| Prevalence | Palau | Male | Glaucoma | 2005 | 2010 | 0 | -1  (-1.04, -0.95) |
| Prevalence | Palau | Male | Glaucoma | 2010 | 2015 | 0 | -0.82  (-0.87, -0.76) |
| Prevalence | Burkina Faso | Female | Glaucoma | 2014 | 2021 | 0.076384723 | -0.3  (-0.47, 0.05) |
| Prevalence | Palestine | Both | Glaucoma | 1990 | 1995 | 0 | -1.24  (-1.56, -0.91) |
| Prevalence | Palestine | Both | Glaucoma | 1995 | 2000 | 0 | -5.58  (-5.83, -5.32) |
| Prevalence | Palestine | Both | Glaucoma | 2000 | 2005 | 0.00559888 | -0.95  (-1.23, -0.52) |
| Prevalence | Palestine | Both | Glaucoma | 2005 | 2015 | 0 | -1.93  (-2.06, -1.82) |
| Prevalence | Palestine | Both | Glaucoma | 2015 | 2019 | 0 | 4.1  (3.77, 4.46) |
| Prevalence | Brunei Darussalam | Both | Glaucoma | 2015 | 2021 | 0 | -0.37  (-0.42, -0.26) |
| Prevalence | Palestine | Female | Glaucoma | 1990 | 1995 | 0.00039992 | -1.17  (-1.44, -0.9) |
| Prevalence | Palestine | Female | Glaucoma | 1995 | 2000 | 0 | -5.41  (-5.63, -5.19) |
| Prevalence | Palestine | Female | Glaucoma | 2000 | 2005 | 0.00159968 | -0.89  (-1.16, -0.53) |
| Prevalence | Palestine | Female | Glaucoma | 2005 | 2015 | 0 | -1.97  (-2.09, -1.87) |
| Prevalence | Palestine | Female | Glaucoma | 2015 | 2019 | 0 | 3.93  (3.64, 4.25) |
| Prevalence | Taiwan (Province of China) | Female | Glaucoma | 2014 | 2021 | 0.035992801 | -0.29  (-0.56, -0.02) |
| Prevalence | Palestine | Male | Glaucoma | 1990 | 1995 | 0.00039992 | -1.24  (-1.6, -0.88) |
| Prevalence | Palestine | Male | Glaucoma | 1995 | 2000 | 0 | -5.71  (-5.98, -5.43) |
| Prevalence | Palestine | Male | Glaucoma | 2000 | 2005 | 0.00719856 | -0.94  (-1.24, -0.48) |
| Prevalence | Palestine | Male | Glaucoma | 2005 | 2015 | 0 | -1.87  (-2.02, -1.77) |
| Prevalence | Palestine | Male | Glaucoma | 2015 | 2019 | 0 | 4.08  (3.74, 4.44) |
| Prevalence | Saint Kitts and Nevis | Female | Glaucoma | 2019 | 2021 | 0 | -0.29  (-0.5, -0.16) |
| Prevalence | Panama | Both | Glaucoma | 1990 | 1995 | 0 | -0.7  (-0.79, -0.6) |
| Prevalence | Panama | Both | Glaucoma | 1995 | 2001 | 0 | -1.21  (-1.28, -1.14) |
| Prevalence | Panama | Both | Glaucoma | 2001 | 2004 | 0 | -2.08  (-2.17, -1.96) |
| Prevalence | Panama | Both | Glaucoma | 2004 | 2013 | 0 | -0.69  (-0.72, -0.65) |
| Prevalence | Serbia | Both | Glaucoma | 2017 | 2021 | 0.00039992 | -0.36  (-0.45, -0.23) |
| Prevalence | Panama | Female | Glaucoma | 1990 | 1995 | 0 | -0.31  (-0.4, -0.22) |
| Prevalence | Panama | Female | Glaucoma | 1995 | 2000 | 0 | -1.08  (-1.19, -0.97) |
| Prevalence | Panama | Female | Glaucoma | 2000 | 2005 | 0 | -1.77  (-1.86, -1.69) |
| Prevalence | Panama | Female | Glaucoma | 2005 | 2011 | 0 | -0.53  (-0.59, -0.45) |
| Prevalence | Russian Federation | Male | Glaucoma | 2019 | 2021 | 0.065986803 | -0.28  (-1.03, 0.03) |
| Prevalence | Panama | Male | Glaucoma | 1990 | 1995 | 0 | -0.93  (-1.01, -0.82) |
| Prevalence | Panama | Male | Glaucoma | 1995 | 2001 | 0 | -1.24  (-1.3, -1.17) |
| Prevalence | Panama | Male | Glaucoma | 2001 | 2004 | 0 | -1.94  (-2.02, -1.81) |
| Prevalence | Panama | Male | Glaucoma | 2004 | 2014 | 0 | -0.74  (-0.76, -0.71) |
| Prevalence | Pakistan | Female | Glaucoma | 2018 | 2021 | 0.091181764 | -0.28  (-0.54, 0.17) |
| Prevalence | Papua New Guinea | Both | Glaucoma | 1990 | 1997 | 0.00719856 | -0.82  (-1.14, -0.66) |
| Prevalence | Papua New Guinea | Both | Glaucoma | 1997 | 2008 | 0.032393521 | -0.24  (-0.56, -0.07) |
| Prevalence | Papua New Guinea | Both | Glaucoma | 2008 | 2014 | 0.027594481 | -0.69  (-1.04, -0.11) |
| Prevalence | Guam | Both | Glaucoma | 1999 | 2021 | 0 | -0.36  (-0.39, -0.33) |
| Prevalence | Papua New Guinea | Female | Glaucoma | 1990 | 1995 | 0.0039992 | -0.82  (-1.91, -0.32) |
| Prevalence | Papua New Guinea | Female | Glaucoma | 1995 | 2009 | 0.24795041 | -0.18  (-0.29, 0.51) |
| Prevalence | Papua New Guinea | Female | Glaucoma | 2009 | 2014 | 0.030793841 | -1.1  (-1.99, -0.5) |
| Prevalence | Canada | Male | Glaucoma | 2015 | 2021 | 0 | -0.27  (-0.56, -0.16) |
| Prevalence | Papua New Guinea | Male | Glaucoma | 1990 | 1993 | 0 | -1.03  (-1.14, -0.95) |
| Prevalence | Papua New Guinea | Male | Glaucoma | 1993 | 1997 | 0 | -0.82  (-0.9, -0.66) |
| Prevalence | Papua New Guinea | Male | Glaucoma | 1997 | 2000 | 0 | -0.6  (-0.67, -0.27) |
| Prevalence | Papua New Guinea | Male | Glaucoma | 2000 | 2006 | 0 | -0.28  (-0.57, -0.24) |
| Prevalence | Papua New Guinea | Male | Glaucoma | 2006 | 2015 | 0 | -0.57  (-0.65, -0.55) |
| Prevalence | Spain | Male | Glaucoma | 2012 | 2021 | 0 | -0.27  (-0.33, -0.19) |
| Prevalence | Paraguay | Both | Glaucoma | 1990 | 2000 | 0 | -0.66  (-0.7, -0.62) |
| Prevalence | Paraguay | Both | Glaucoma | 2000 | 2010 | 0 | -0.41  (-0.44, -0.35) |
| Prevalence | Paraguay | Both | Glaucoma | 2010 | 2014 | 0 | -0.97  (-1.13, -0.82) |
| Prevalence | Sweden | Both | Glaucoma | 2009 | 2021 | 0.049190162 | -0.35  (-2.02, -0.05) |
| Prevalence | Belarus | Female | Glaucoma | 2018 | 2021 | 0.00119976 | -0.27  (-0.34, -0.2) |
| Prevalence | Paraguay | Male | Glaucoma | 1990 | 2001 | 0 | -0.67  (-0.72, -0.63) |
| Prevalence | Paraguay | Male | Glaucoma | 2001 | 2005 | 0.292741452 | 0.06  (-0.68, 0.21) |
| Prevalence | Paraguay | Male | Glaucoma | 2005 | 2010 | 0.122775445 | -0.44  (-0.53, 0.07) |
| Prevalence | Paraguay | Male | Glaucoma | 2010 | 2014 | 0 | -0.94  (-1.08, -0.43) |
| Prevalence | Paraguay | Male | Glaucoma | 2014 | 2019 | 0 | -0.62  (-0.91, -0.49) |
| Prevalence | Spain | Female | Glaucoma | 2019 | 2021 | 0.058788242 | -0.27  (-0.66, 0.01) |
| Prevalence | Peru | Both | Glaucoma | 1990 | 1995 | 0 | -0.46  (-0.54, -0.39) |
| Prevalence | Peru | Both | Glaucoma | 1995 | 2000 | 0 | -1.48  (-1.6, -1.4) |
| Prevalence | Peru | Both | Glaucoma | 2000 | 2005 | 0 | -0.92  (-1.01, -0.76) |
| Prevalence | Peru | Both | Glaucoma | 2005 | 2011 | 0 | -1.31  (-1.39, -1.24) |
| Prevalence | Peru | Both | Glaucoma | 2011 | 2015 | 0 | -1.95  (-2.07, -1.85) |
| Prevalence | Bermuda | Both | Glaucoma | 2017 | 2021 | 0 | -0.34  (-0.37, -0.3) |
| Prevalence | Peru | Female | Glaucoma | 1990 | 1995 | 0.0019996 | -0.33  (-0.43, -0.21) |
| Prevalence | Peru | Female | Glaucoma | 1995 | 2001 | 0 | -1.53  (-1.69, -1.45) |
| Prevalence | Peru | Female | Glaucoma | 2001 | 2011 | 0 | -1.27  (-1.31, -1.2) |
| Prevalence | Peru | Female | Glaucoma | 2011 | 2015 | 0 | -2.14  (-2.3, -1.99) |
| Prevalence | Mozambique | Female | Glaucoma | 2019 | 2021 | 0.049990002 | -0.27  (-0.69, 0) |
| Prevalence | Peru | Male | Glaucoma | 1990 | 1994 | 0.00159968 | -0.47  (-0.63, -0.31) |
| Prevalence | Peru | Male | Glaucoma | 1994 | 2001 | 0 | -1.28  (-1.38, -1.21) |
| Prevalence | Peru | Male | Glaucoma | 2001 | 2005 | 0 | -0.51  (-0.67, -0.29) |
| Prevalence | Peru | Male | Glaucoma | 2005 | 2011 | 0 | -1.36  (-1.43, -1.27) |
| Prevalence | Peru | Male | Glaucoma | 2011 | 2015 | 0 | -1.78  (-2.01, -1.65) |
| Prevalence | Monaco | Female | Glaucoma | 2019 | 2021 | 0.041591682 | -0.26  (-0.63, -0.02) |
| Prevalence | Philippines | Both | Glaucoma | 1990 | 1993 | 0 | -0.8  (-0.94, -0.7) |
| Prevalence | Philippines | Both | Glaucoma | 1993 | 1999 | 0 | -0.55  (-0.58, -0.49) |
| Prevalence | Philippines | Both | Glaucoma | 1999 | 2006 | 0 | -0.18  (-0.21, -0.14) |
| Prevalence | Philippines | Both | Glaucoma | 2006 | 2011 | 0 | -0.39  (-0.44, -0.33) |
| Prevalence | Philippines | Both | Glaucoma | 2011 | 2015 | 0 | -0.64  (-0.73, -0.58) |
| Prevalence | Belgium | Both | Glaucoma | 2019 | 2021 | 0 | -0.34  (-0.53, -0.24) |
| Prevalence | Philippines | Female | Glaucoma | 1990 | 1994 | 0 | -0.56  (-0.71, -0.45) |
| Prevalence | Philippines | Female | Glaucoma | 1994 | 2007 | 0 | -0.22  (-0.23, -0.19) |
| Prevalence | Philippines | Female | Glaucoma | 2007 | 2015 | 0 | -0.42  (-0.46, -0.39) |
| Prevalence | Philippines | Female | Glaucoma | 2015 | 2019 | 0 | -1.83  (-1.89, -1.76) |
| Prevalence | Monaco | Male | Glaucoma | 2019 | 2021 | 0.00359928 | -0.25  (-0.46, -0.1) |
| Prevalence | Philippines | Male | Glaucoma | 1990 | 2000 | 0 | -0.89  (-0.92, -0.87) |
| Prevalence | Philippines | Male | Glaucoma | 2000 | 2005 | 0.081583683 | -0.06  (-0.11, 0.01) |
| Prevalence | Philippines | Male | Glaucoma | 2005 | 2010 | 0 | -0.39  (-0.48, -0.31) |
| Prevalence | Philippines | Male | Glaucoma | 2010 | 2015 | 0 | -0.81  (-0.9, -0.75) |
| Prevalence | Philippines | Male | Glaucoma | 2015 | 2019 | 0.835432913 | 0.01  (-0.07, 0.09) |
| Prevalence | Lao People's Democratic Republic | Male | Glaucoma | 2019 | 2021 | 0 | -0.24  (-0.4, -0.1) |
| Prevalence | Poland | Both | Glaucoma | 1990 | 1994 | 0 | -0.63  (-0.77, -0.56) |
| Prevalence | Poland | Both | Glaucoma | 1994 | 2000 | 0 | -0.41  (-0.44, -0.35) |
| Prevalence | Poland | Both | Glaucoma | 2000 | 2005 | 0 | -1.22  (-1.25, -1.19) |
| Prevalence | Poland | Both | Glaucoma | 2005 | 2017 | 0 | -0.52  (-0.54, -0.51) |
| Prevalence | Russian Federation | Both | Glaucoma | 2019 | 2021 | 0.00879824 | -0.34  (-0.79, -0.1) |
| Prevalence | Poland | Female | Glaucoma | 1990 | 1994 | 0 | -0.81  (-0.93, -0.72) |
| Prevalence | Poland | Female | Glaucoma | 1994 | 2000 | 0 | -0.3  (-0.34, -0.24) |
| Prevalence | Poland | Female | Glaucoma | 2000 | 2005 | 0 | -1.12  (-1.21, -1.07) |
| Prevalence | Poland | Female | Glaucoma | 2005 | 2019 | 0 | -0.69  (-0.7, -0.67) |
| Prevalence | Colombia | Female | Glaucoma | 2019 | 2021 | 0.00359928 | -0.23  (-0.42, -0.09) |
| Prevalence | Poland | Male | Glaucoma | 1990 | 1997 | 0 | -0.45  (-0.49, -0.39) |
| Prevalence | Poland | Male | Glaucoma | 1997 | 2001 | 0 | -0.77  (-0.89, -0.63) |
| Prevalence | Poland | Male | Glaucoma | 2001 | 2004 | 0 | -1.68  (-1.76, -1.49) |
| Prevalence | Poland | Male | Glaucoma | 2004 | 2015 | 0 | -0.45  (-0.53, -0.42) |
| Prevalence | Poland | Male | Glaucoma | 2015 | 2019 | 0.203559288 | -0.08  (-0.45, 0.07) |
| Prevalence | Nigeria | Male | Glaucoma | 2016 | 2021 | 0.213957209 | -0.23  (-0.48, 0.27) |
| Prevalence | Portugal | Both | Glaucoma | 1990 | 1993 | 0 | -2.02  (-2.35, -1.74) |
| Prevalence | Portugal | Both | Glaucoma | 1993 | 2006 | 0 | -1.66  (-1.72, -1.46) |
| Prevalence | Portugal | Both | Glaucoma | 2006 | 2009 | 0 | -1.24  (-1.65, -0.71) |
| Prevalence | United Kingdom | Both | Glaucoma | 2015 | 2021 | 0 | -0.33  (-0.37, -0.3) |
| Prevalence | Portugal | Female | Glaucoma | 1990 | 2007 | 0 | -1.77  (-1.82, -1.73) |
| Prevalence | Lithuania | Female | Glaucoma | 2019 | 2021 | 0.053589282 | -0.22  (-0.55, 0) |
| Prevalence | Portugal | Male | Glaucoma | 1990 | 1994 | 0 | -1.96  (-2.11, -1.86) |
| Prevalence | Portugal | Male | Glaucoma | 1994 | 2006 | 0 | -1.61  (-1.63, -1.59) |
| Prevalence | Portugal | Male | Glaucoma | 2006 | 2010 | 0 | -1.15  (-1.31, -1) |
| Prevalence | Portugal | Male | Glaucoma | 2010 | 2015 | 0 | -0.65  (-0.71, -0.5) |
| Prevalence | Portugal | Male | Glaucoma | 2015 | 2018 | 0 | -0.99  (-1.08, -0.86) |
| Prevalence | Haiti | Female | Glaucoma | 2018 | 2021 | 0.052789442 | -0.22  (-0.38, 0.01) |
| Prevalence | Puerto Rico | Both | Glaucoma | 1990 | 1995 | 0 | -1.07  (-1.11, -1.05) |
| Prevalence | Puerto Rico | Both | Glaucoma | 1995 | 2003 | 0 | -1.33  (-1.36, -1.32) |
| Prevalence | Puerto Rico | Both | Glaucoma | 2003 | 2007 | 0 | -1.12  (-1.2, -1.04) |
| Prevalence | Puerto Rico | Both | Glaucoma | 2007 | 2015 | 0 | -0.95  (-0.96, -0.93) |
| Prevalence | Bahamas | Both | Glaucoma | 2018 | 2021 | 0.00079984 | -0.33  (-0.49, -0.12) |
| Prevalence | Puerto Rico | Female | Glaucoma | 1990 | 1995 | 0 | -1.02  (-1.09, -0.94) |
| Prevalence | Puerto Rico | Female | Glaucoma | 1995 | 2000 | 0 | -1.42  (-1.56, -1.34) |
| Prevalence | Puerto Rico | Female | Glaucoma | 2000 | 2011 | 0 | -1.12  (-1.14, -1.09) |
| Prevalence | Puerto Rico | Female | Glaucoma | 2011 | 2019 | 0 | -0.76  (-0.81, -0.72) |
| Prevalence | Chad | Female | Glaucoma | 2015 | 2021 | 0 | -0.22  (-0.32, -0.14) |
| Prevalence | Puerto Rico | Male | Glaucoma | 1990 | 1999 | 0 | -1.12  (-1.15, -1.08) |
| Prevalence | Puerto Rico | Male | Glaucoma | 1999 | 2005 | 0 | -1.41  (-1.51, -1.36) |
| Prevalence | Puerto Rico | Male | Glaucoma | 2005 | 2010 | 0 | -0.79  (-0.86, -0.65) |
| Prevalence | Puerto Rico | Male | Glaucoma | 2010 | 2015 | 0 | -1.08  (-1.22, -1.01) |
| Prevalence | Puerto Rico | Male | Glaucoma | 2015 | 2019 | 0 | -0.49  (-0.58, -0.34) |
| Prevalence | San Marino | Male | Glaucoma | 2008 | 2021 | 0 | -0.21  (-0.24, -0.18) |
| Prevalence | Qatar | Both | Glaucoma | 1990 | 1994 | 0.00159968 | -2.36  (-5.72, -0.88) |
| Prevalence | Qatar | Both | Glaucoma | 1994 | 2000 | 0.00039992 | 1.47  (0.71, 3.63) |
| Prevalence | Qatar | Both | Glaucoma | 2000 | 2019 | 0 | -1.96  (-2.08, -1.82) |
| Prevalence | Chad | Both | Glaucoma | 2012 | 2021 | 0.00039992 | -0.32  (-0.35, -0.29) |
| Prevalence | Qatar | Female | Glaucoma | 1990 | 1995 | 0.057988402 | -1.65  (-6.68, 0.08) |
| Prevalence | Qatar | Female | Glaucoma | 1995 | 2000 | 0.00239952 | 4.13  (2.46, 7.73) |
| Prevalence | Qatar | Female | Glaucoma | 2000 | 2019 | 0.00039992 | -1.93  (-2.14, -1.7) |
| Prevalence | United Kingdom | Male | Glaucoma | 2017 | 2021 | 0 | -0.21  (-0.25, -0.13) |
| Prevalence | Qatar | Male | Glaucoma | 1990 | 1994 | 0.00039992 | -2.43  (-4.83, -1.35) |
| Prevalence | Qatar | Male | Glaucoma | 1994 | 2001 | 0.063187363 | 0.33  (-0.02, 1.54) |
| Prevalence | Qatar | Male | Glaucoma | 2001 | 2005 | 0.01039792 | -2.81  (-3.51, -2.06) |
| Prevalence | Qatar | Male | Glaucoma | 2005 | 2019 | 0 | -1.84  (-1.95, -1.31) |
| Prevalence | Burundi | Female | Glaucoma | 1990 | 2021 | 0 | -0.21  (-0.25, -0.17) |
| Prevalence | Republic of Korea | Both | Glaucoma | 1990 | 1995 | 0 | -2.74  (-2.89, -2.4) |
| Prevalence | Republic of Korea | Both | Glaucoma | 1995 | 2000 | 0 | -3.03  (-3.35, -2.01) |
| Prevalence | Republic of Korea | Both | Glaucoma | 2000 | 2005 | 0 | -1.95  (-2.26, -1.36) |
| Prevalence | Republic of Korea | Both | Glaucoma | 2005 | 2010 | 0 | -1.36  (-1.58, -0.62) |
| Prevalence | Republic of Korea | Both | Glaucoma | 2010 | 2019 | 0 | -0.65  (-0.75, -0.55) |
| Prevalence | Finland | Both | Glaucoma | 2019 | 2021 | 0.00039992 | -0.31  (-0.53, -0.17) |
| Prevalence | Republic of Korea | Female | Glaucoma | 1990 | 1995 | 0 | -2.64  (-2.77, -2.43) |
| Prevalence | Republic of Korea | Female | Glaucoma | 1995 | 1999 | 0 | -3.25  (-3.54, -3.04) |
| Prevalence | Republic of Korea | Female | Glaucoma | 1999 | 2002 | 0 | -2.3  (-2.81, -1.72) |
| Prevalence | Republic of Korea | Female | Glaucoma | 2002 | 2009 | 0 | -1.55  (-1.67, -1.24) |
| Prevalence | Republic of Korea | Female | Glaucoma | 2009 | 2019 | 0 | -0.65  (-0.71, -0.58) |
| Prevalence | Czechia | Male | Glaucoma | 2014 | 2021 | 0 | -0.21  (-0.23, -0.19) |
| Prevalence | Republic of Korea | Male | Glaucoma | 1990 | 1999 | 0 | -2.89  (-3.03, -2.82) |
| Prevalence | Republic of Korea | Male | Glaucoma | 1999 | 2005 | 0 | -2.4  (-2.65, -2.18) |
| Prevalence | Republic of Korea | Male | Glaucoma | 2005 | 2012 | 0 | -1.27  (-1.67, -1.13) |
| Prevalence | Republic of Korea | Male | Glaucoma | 2012 | 2019 | 0 | -0.67  (-1.16, -0.53) |
| Prevalence | Costa Rica | Female | Glaucoma | 2018 | 2021 | 0.025194961 | -0.21  (-0.33, -0.09) |
| Prevalence | Republic of Moldova | Both | Glaucoma | 1990 | 1994 | 0 | -0.96  (-1.04, -0.87) |
| Prevalence | Republic of Moldova | Both | Glaucoma | 1994 | 2004 | 0 | -0.11  (-0.13, -0.08) |
| Prevalence | Republic of Moldova | Both | Glaucoma | 2004 | 2019 | 0 | -0.95  (-0.97, -0.94) |
| Prevalence | Dominica | Both | Glaucoma | 2018 | 2021 | 0 | -0.31  (-0.36, -0.26) |
| Prevalence | Republic of Moldova | Female | Glaucoma | 1990 | 1994 | 0 | -0.92  (-1, -0.84) |
| Prevalence | Republic of Moldova | Female | Glaucoma | 1994 | 2003 | 0.01119776 | -0.05  (-0.08, -0.02) |
| Prevalence | Republic of Moldova | Female | Glaucoma | 2003 | 2012 | 0 | -0.69  (-0.72, -0.66) |
| Prevalence | Republic of Moldova | Female | Glaucoma | 2012 | 2019 | 0 | -0.92  (-0.98, -0.88) |
| Prevalence | Niger | Male | Glaucoma | 2014 | 2021 | 0.00359928 | -0.2  (-0.25, -0.16) |
| Prevalence | Republic of Moldova | Male | Glaucoma | 1990 | 1994 | 0 | -0.89  (-1.11, -0.76) |
| Prevalence | Republic of Moldova | Male | Glaucoma | 1994 | 2004 | 0.00279944 | -0.1  (-0.13, -0.07) |
| Prevalence | Republic of Moldova | Male | Glaucoma | 2004 | 2011 | 0 | -1.29  (-1.37, -1.24) |
| Prevalence | Republic of Moldova | Male | Glaucoma | 2011 | 2019 | 0 | -1.12  (-1.16, -1.05) |
| Prevalence | Georgia | Male | Glaucoma | 2018 | 2021 | 0.030393921 | -0.2  (-0.32, -0.08) |
| Prevalence | Romania | Both | Glaucoma | 1990 | 1995 | 0 | -1.02  (-1.09, -0.98) |
| Prevalence | Romania | Both | Glaucoma | 1995 | 2000 | 0 | -0.72  (-0.76, -0.67) |
| Prevalence | Romania | Both | Glaucoma | 2000 | 2009 | 0 | -1.19  (-1.22, -1.18) |
| Prevalence | Romania | Both | Glaucoma | 2009 | 2014 | 0 | -0.9  (-1.02, -0.84) |
| Prevalence | Romania | Both | Glaucoma | 2014 | 2017 | 0 | -0.6  (-0.79, -0.42) |
| Prevalence | American Samoa | Both | Glaucoma | 2015 | 2021 | 0.075984803 | -0.31  (-0.47, 0.09) |
| Prevalence | Romania | Female | Glaucoma | 1990 | 1994 | 0 | -1.37  (-1.44, -1.31) |
| Prevalence | Romania | Female | Glaucoma | 1994 | 2001 | 0 | -0.51  (-0.54, -0.48) |
| Prevalence | Romania | Female | Glaucoma | 2001 | 2009 | 0 | -1.19  (-1.23, -1.16) |
| Prevalence | Romania | Female | Glaucoma | 2009 | 2014 | 0 | -0.95  (-1.07, -0.86) |
| Prevalence | Romania | Female | Glaucoma | 2014 | 2018 | 0 | -0.63  (-0.75, -0.48) |
| Prevalence | Republic of Moldova | Male | Glaucoma | 2019 | 2021 | 0.028794241 | -0.2  (-0.48, -0.02) |
| Prevalence | Romania | Male | Glaucoma | 1990 | 1995 | 0 | -0.71  (-0.79, -0.57) |
| Prevalence | Romania | Male | Glaucoma | 1995 | 1999 | 0 | -0.97  (-1.19, -0.85) |
| Prevalence | Romania | Male | Glaucoma | 1999 | 2008 | 0 | -1.21  (-1.29, -0.92) |
| Prevalence | Romania | Male | Glaucoma | 2008 | 2014 | 0 | -0.88  (-0.95, -0.59) |
| Prevalence | Montenegro | Female | Glaucoma | 2018 | 2021 | 0.045190962 | -0.18  (-0.3, -0.06) |
| Prevalence | Russian Federation | Both | Glaucoma | 1990 | 1994 | 0 | -0.7  (-1.01, -0.52) |
| Prevalence | Russian Federation | Both | Glaucoma | 1994 | 2001 | 0.136372725 | 0.06  (-0.07, 0.2) |
| Prevalence | Russian Federation | Both | Glaucoma | 2001 | 2005 | 0.062787443 | -1.78  (-1.96, 0.07) |
| Prevalence | Russian Federation | Both | Glaucoma | 2005 | 2010 | 0 | -1.42  (-1.7, -1.1) |
| Prevalence | Russian Federation | Both | Glaucoma | 2010 | 2019 | 0 | -1.06  (-1.2, -0.96) |
| Prevalence | Armenia | Both | Glaucoma | 2019 | 2021 | 0.01079784 | -0.31  (-0.67, -0.1) |
| Prevalence | Russian Federation | Female | Glaucoma | 1990 | 1994 | 0 | -0.68  (-0.92, -0.53) |
| Prevalence | Russian Federation | Female | Glaucoma | 1994 | 2001 | 0.058388322 | -0.09  (-0.15, 0) |
| Prevalence | Russian Federation | Female | Glaucoma | 2001 | 2005 | 0.00559888 | -1.93  (-2.1, -1.8) |
| Prevalence | Russian Federation | Female | Glaucoma | 2005 | 2010 | 0 | -1.37  (-1.62, -1.21) |
| Prevalence | Russian Federation | Female | Glaucoma | 2010 | 2019 | 0 | -1.05  (-1.13, -0.98) |
| Prevalence | Australia | Female | Glaucoma | 2018 | 2021 | 0.100779844 | -0.18  (-0.31, 0.06) |
| Prevalence | Russian Federation | Male | Glaucoma | 1990 | 1994 | 0 | -0.83  (-1.32, -0.58) |
| Prevalence | Russian Federation | Male | Glaucoma | 1994 | 2001 | 0.021595681 | 0.18  (0.08, 0.33) |
| Prevalence | Russian Federation | Male | Glaucoma | 2001 | 2011 | 0.021995601 | -1.68  (-1.79, -1.59) |
| Prevalence | Russian Federation | Male | Glaucoma | 2011 | 2019 | 0 | -1.15  (-1.65, -1.03) |
| Prevalence | Kazakhstan | Male | Glaucoma | 2018 | 2021 | 0.075184963 | -0.18  (-0.36, 0.07) |
| Prevalence | Rwanda | Both | Glaucoma | 1990 | 1993 | 0.330733853 | 0.06  (-0.06, 0.25) |
| Prevalence | Rwanda | Both | Glaucoma | 1993 | 1997 | 0 | -0.3  (-0.48, -0.22) |
| Prevalence | Rwanda | Both | Glaucoma | 1997 | 2001 | 0 | -0.94  (-1.07, -0.88) |
| Prevalence | Rwanda | Both | Glaucoma | 2001 | 2010 | 0 | -1.68  (-1.7, -1.66) |
| Prevalence | Rwanda | Both | Glaucoma | 2010 | 2019 | 0 | -0.69  (-0.72, -0.67) |
| Prevalence | Georgia | Both | Glaucoma | 2019 | 2021 | 0 | -0.31  (-0.58, -0.18) |
| Prevalence | Rwanda | Female | Glaucoma | 1990 | 1996 | 0.00079984 | -0.17  (-0.23, -0.1) |
| Prevalence | Rwanda | Female | Glaucoma | 1996 | 2001 | 0.00039992 | -0.74  (-0.82, -0.63) |
| Prevalence | Rwanda | Female | Glaucoma | 2001 | 2005 | 0 | -1.29  (-1.45, -1.12) |
| Prevalence | Rwanda | Female | Glaucoma | 2005 | 2010 | 0 | -1.69  (-1.78, -1.64) |
| Prevalence | Rwanda | Female | Glaucoma | 2010 | 2019 | 0 | -0.69  (-0.71, -0.65) |
| Prevalence | Kyrgyzstan | Male | Glaucoma | 2018 | 2021 | 0.020395921 | -0.18  (-0.34, -0.03) |
| Prevalence | Rwanda | Male | Glaucoma | 1990 | 1996 | 0.23915217 | 0.03  (-0.03, 0.1) |
| Prevalence | Rwanda | Male | Glaucoma | 1996 | 2001 | 0 | -0.8  (-0.88, -0.71) |
| Prevalence | Rwanda | Male | Glaucoma | 2001 | 2005 | 0 | -2.08  (-2.24, -1.96) |
| Prevalence | Rwanda | Male | Glaucoma | 2005 | 2010 | 0 | -1.47  (-1.57, -1.32) |
| Prevalence | Rwanda | Male | Glaucoma | 2010 | 2016 | 0 | -0.58  (-0.65, -0.48) |
| Prevalence | Estonia | Female | Glaucoma | 2015 | 2021 | 0.00039992 | -0.17  (-0.21, -0.13) |
| Prevalence | Saint Kitts and Nevis | Both | Glaucoma | 1990 | 1995 | 0 | -1.8  (-1.87, -1.75) |
| Prevalence | Saint Kitts and Nevis | Both | Glaucoma | 1995 | 2000 | 0 | -1.64  (-1.67, -1.57) |
| Prevalence | Saint Kitts and Nevis | Both | Glaucoma | 2000 | 2010 | 0 | -1.12  (-1.14, -1.11) |
| Prevalence | Bangladesh | Both | Glaucoma | 2019 | 2021 | 0.053589282 | -0.3  (-0.69, 0) |
| Prevalence | Saint Kitts and Nevis | Female | Glaucoma | 1990 | 2000 | 0 | -1.72  (-1.74, -1.7) |
| Prevalence | Saint Kitts and Nevis | Female | Glaucoma | 2000 | 2005 | 0 | -1.04  (-1.08, -0.96) |
| Prevalence | Saint Kitts and Nevis | Female | Glaucoma | 2005 | 2010 | 0 | -1.25  (-1.38, -1.2) |
| Prevalence | Saint Kitts and Nevis | Female | Glaucoma | 2010 | 2019 | 0 | -0.7  (-0.74, -0.67) |
| Prevalence | Canada | Female | Glaucoma | 2009 | 2021 | 0.012797441 | -0.16  (-0.19, -0.08) |
| Prevalence | Saint Kitts and Nevis | Male | Glaucoma | 1990 | 1999 | 0 | -1.79  (-1.85, -1.75) |
| Prevalence | Saint Kitts and Nevis | Male | Glaucoma | 1999 | 2006 | 0 | -1.31  (-1.42, -1.24) |
| Prevalence | Saint Kitts and Nevis | Male | Glaucoma | 2006 | 2014 | 0 | -0.86  (-1.02, -0.81) |
| Prevalence | Saint Kitts and Nevis | Male | Glaucoma | 2014 | 2019 | 0 | -0.64  (-0.73, -0.43) |
| Prevalence | Cyprus | Female | Glaucoma | 2019 | 2021 | 0.055188962 | -0.16  (-0.52, 0) |
| Prevalence | Saint Lucia | Both | Glaucoma | 1990 | 1994 | 0 | -1.88  (-2, -1.81) |
| Prevalence | Saint Lucia | Both | Glaucoma | 1994 | 1999 | 0 | -1.64  (-1.68, -1.56) |
| Prevalence | Saint Lucia | Both | Glaucoma | 1999 | 2010 | 0 | -1.05  (-1.06, -1.03) |
| Prevalence | Saint Lucia | Both | Glaucoma | 2010 | 2015 | 0 | -0.79  (-0.87, -0.71) |
| Prevalence | Micronesia (Federated States of) | Both | Glaucoma | 2014 | 2021 | 0.097180564 | -0.29  (-0.48, 0.14) |
| Prevalence | Saint Lucia | Female | Glaucoma | 1990 | 1997 | 0 | -1.79  (-1.86, -1.76) |
| Prevalence | Saint Lucia | Female | Glaucoma | 1997 | 2000 | 0 | -1.54  (-1.64, -1.23) |
| Prevalence | Saint Lucia | Female | Glaucoma | 2000 | 2005 | 0 | -0.91  (-0.97, -0.82) |
| Prevalence | Saint Lucia | Female | Glaucoma | 2005 | 2010 | 0 | -1.3  (-1.38, -1.24) |
| Prevalence | Saint Lucia | Female | Glaucoma | 2010 | 2017 | 0 | -0.75  (-0.79, -0.71) |
| Prevalence | Ireland | Male | Glaucoma | 2019 | 2021 | 0.060787842 | -0.15  (-0.36, 0.01) |
| Prevalence | Saint Lucia | Male | Glaucoma | 1990 | 1997 | 0 | -1.82  (-1.95, -1.74) |
| Prevalence | Saint Lucia | Male | Glaucoma | 1997 | 2004 | 0 | -1.31  (-1.45, -1.19) |
| Prevalence | Saint Lucia | Male | Glaucoma | 2004 | 2015 | 0 | -0.9  (-0.98, -0.85) |
| Prevalence | Austria | Male | Glaucoma | 2019 | 2021 | 0.040791842 | -0.15  (-0.34, -0.01) |
| Prevalence | Saint Vincent and the Grenadines | Both | Glaucoma | 1990 | 1994 | 0 | -1.28  (-1.37, -1.22) |
| Prevalence | Saint Vincent and the Grenadines | Both | Glaucoma | 1994 | 2009 | 0 | -1.05  (-1.06, -1.04) |
| Prevalence | Saint Vincent and the Grenadines | Both | Glaucoma | 2009 | 2015 | 0 | -0.6  (-0.62, -0.56) |
| Prevalence | North Macedonia | Both | Glaucoma | 2015 | 2021 | 0 | -0.29  (-0.33, -0.23) |
| Prevalence | Saint Vincent and the Grenadines | Female | Glaucoma | 1990 | 1996 | 0 | -1.27  (-1.34, -1.23) |
| Prevalence | Saint Vincent and the Grenadines | Female | Glaucoma | 1996 | 2005 | 0 | -1.04  (-1.06, -0.95) |
| Prevalence | Saint Vincent and the Grenadines | Female | Glaucoma | 2005 | 2010 | 0 | -1.14  (-1.26, -1.09) |
| Prevalence | Saint Vincent and the Grenadines | Female | Glaucoma | 2010 | 2015 | 0 | -0.63  (-0.67, -0.52) |
| Prevalence | Saint Vincent and the Grenadines | Female | Glaucoma | 2015 | 2019 | 0 | -0.9  (-1.02, -0.85) |
| Prevalence | Cyprus | Male | Glaucoma | 2019 | 2021 | 0.072785443 | -0.14  (-0.43, 0.02) |
| Prevalence | Saint Vincent and the Grenadines | Male | Glaucoma | 1990 | 1994 | 0 | -1.5  (-1.63, -1.4) |
| Prevalence | Saint Vincent and the Grenadines | Male | Glaucoma | 1994 | 2009 | 0 | -1.09  (-1.1, -1.08) |
| Prevalence | Saint Vincent and the Grenadines | Male | Glaucoma | 2009 | 2019 | 0 | -0.7  (-0.72, -0.67) |
| Prevalence | Switzerland | Female | Glaucoma | 2019 | 2021 | 0.097980404 | -0.14  (-0.51, 0.05) |
| Prevalence | Samoa | Both | Glaucoma | 1990 | 1998 | 0.00719856 | -0.93  (-1.38, -0.78) |
| Prevalence | Samoa | Both | Glaucoma | 1998 | 2015 | 0.00039992 | -0.68  (-0.8, -0.6) |
| Prevalence | Hungary | Both | Glaucoma | 2018 | 2021 | 0.00479904 | -0.27  (-0.36, -0.19) |
| Prevalence | Samoa | Female | Glaucoma | 1990 | 1997 | 0.017996401 | -0.88  (-1.78, -0.59) |
| Prevalence | Samoa | Female | Glaucoma | 1997 | 2010 | 0.176364727 | -0.47  (-0.59, 0.28) |
| Prevalence | Samoa | Female | Glaucoma | 2010 | 2014 | 0.031993601 | -1.17  (-1.85, -0.58) |
| Prevalence | United States Virgin Islands | Female | Glaucoma | 2018 | 2021 | 0.017596481 | -0.14  (-0.21, -0.08) |
| Prevalence | Samoa | Male | Glaucoma | 1990 | 2000 | 0 | -1.11  (-1.16, -1.08) |
| Prevalence | Samoa | Male | Glaucoma | 2000 | 2005 | 0 | -0.8  (-1.14, -0.69) |
| Prevalence | Samoa | Male | Glaucoma | 2005 | 2010 | 0 | -0.88  (-0.98, -0.83) |
| Prevalence | Samoa | Male | Glaucoma | 2010 | 2015 | 0 | -0.42  (-0.46, -0.36) |
| Prevalence | Samoa | Male | Glaucoma | 2015 | 2019 | 0 | -1.38  (-1.47, -1.34) |
| Prevalence | Puerto Rico | Female | Glaucoma | 2019 | 2021 | 0.062787443 | -0.13  (-0.38, 0.01) |
| Prevalence | San Marino | Both | Glaucoma | 1990 | 1999 | 0 | -0.92  (-1, -0.88) |
| Prevalence | San Marino | Both | Glaucoma | 1999 | 2004 | 0 | -0.62  (-0.94, -0.52) |
| Prevalence | San Marino | Both | Glaucoma | 2004 | 2009 | 0 | -0.41  (-0.59, -0.29) |
| Prevalence | San Marino | Both | Glaucoma | 2009 | 2016 | 0.00039992 | -0.23  (-0.27, -0.08) |
| Prevalence | San Marino | Both | Glaucoma | 2016 | 2019 | 0 | -0.49  (-0.6, -0.37) |
| Prevalence | Jordan | Both | Glaucoma | 2018 | 2021 | 0.082783443 | -0.26  (-0.55, 0.17) |
| Prevalence | San Marino | Female | Glaucoma | 1990 | 1999 | 0 | -0.89  (-0.97, -0.85) |
| Prevalence | San Marino | Female | Glaucoma | 1999 | 2004 | 0 | -0.63  (-0.95, -0.55) |
| Prevalence | San Marino | Female | Glaucoma | 2004 | 2009 | 0 | -0.45  (-0.63, -0.36) |
| Prevalence | San Marino | Female | Glaucoma | 2009 | 2015 | 0 | -0.31  (-0.36, -0.18) |
| Prevalence | San Marino | Female | Glaucoma | 2015 | 2019 | 0 | -0.56  (-0.72, -0.49) |
| Prevalence | Japan | Female | Glaucoma | 2015 | 2021 | 0 | -0.13  (-0.22, -0.07) |
| Prevalence | San Marino | Male | Glaucoma | 1990 | 2000 | 0 | -0.97  (-1.02, -0.93) |
| Prevalence | San Marino | Male | Glaucoma | 2000 | 2008 | 0 | -0.52  (-0.61, -0.44) |
| Prevalence | Slovakia | Male | Glaucoma | 2015 | 2021 | 0.00039992 | -0.13  (-0.15, -0.11) |
| Prevalence | Sao Tome and Principe | Both | Glaucoma | 1990 | 1995 | 0.00159968 | -0.11  (-0.14, -0.08) |
| Prevalence | Sao Tome and Principe | Both | Glaucoma | 1995 | 1998 | 0.00119976 | -0.47  (-0.54, -0.35) |
| Prevalence | Sao Tome and Principe | Both | Glaucoma | 1998 | 2002 | 0 | -0.73  (-0.8, -0.69) |
| Prevalence | Sao Tome and Principe | Both | Glaucoma | 2002 | 2010 | 0 | -0.98  (-1, -0.96) |
| Prevalence | Sao Tome and Principe | Both | Glaucoma | 2010 | 2019 | 0 | -1.11  (-1.14, -1.1) |
| Prevalence | Ireland | Both | Glaucoma | 2019 | 2021 | 0.029194161 | -0.24  (-0.5, -0.04) |
| Prevalence | Sao Tome and Principe | Female | Glaucoma | 1990 | 1993 | 0.135172965 | -0.08  (-0.15, 0.05) |
| Prevalence | Sao Tome and Principe | Female | Glaucoma | 1993 | 1996 | 0 | -0.32  (-0.64, -0.26) |
| Prevalence | Sao Tome and Principe | Female | Glaucoma | 1996 | 2004 | 0 | -0.74  (-0.77, -0.71) |
| Prevalence | Sao Tome and Principe | Female | Glaucoma | 2004 | 2015 | 0 | -0.97  (-0.99, -0.95) |
| Prevalence | Sao Tome and Principe | Female | Glaucoma | 2015 | 2019 | 0 | -1.18  (-1.26, -1.12) |
| Prevalence | Nicaragua | Female | Glaucoma | 2019 | 2021 | 0.00879824 | -0.13  (-0.25, -0.03) |
| Prevalence | Sao Tome and Principe | Male | Glaucoma | 1990 | 1996 | 0 | -0.35  (-0.38, -0.32) |
| Prevalence | Sao Tome and Principe | Male | Glaucoma | 1996 | 2000 | 0 | -0.62  (-0.68, -0.57) |
| Prevalence | Sao Tome and Principe | Male | Glaucoma | 2000 | 2010 | 0 | -0.99  (-1.01, -0.98) |
| Prevalence | Sao Tome and Principe | Male | Glaucoma | 2010 | 2016 | 0 | -1.16  (-1.22, -1.12) |
| Prevalence | Northern Mariana Islands | Female | Glaucoma | 1998 | 2021 | 0 | -0.13  (-0.17, -0.08) |
| Prevalence | Saudi Arabia | Both | Glaucoma | 1990 | 2000 | 0 | -1.84  (-1.91, -1.76) |
| Prevalence | Saudi Arabia | Both | Glaucoma | 2000 | 2005 | 0 | 1.42  (1.26, 1.56) |
| Prevalence | Saudi Arabia | Both | Glaucoma | 2005 | 2015 | 0 | -1.93  (-2.06, -1.84) |
| Prevalence | Monaco | Both | Glaucoma | 2019 | 2021 | 0.021195761 | -0.24  (-0.55, -0.06) |
| Prevalence | Saudi Arabia | Female | Glaucoma | 1990 | 1996 | 0 | -1.58  (-1.65, -1.46) |
| Prevalence | Saudi Arabia | Female | Glaucoma | 1996 | 1999 | 0.00119976 | -2.08  (-2.19, -1.91) |
| Prevalence | Saudi Arabia | Female | Glaucoma | 1999 | 2006 | 0.00039992 | -0.19  (-0.26, -0.14) |
| Prevalence | Saudi Arabia | Female | Glaucoma | 2006 | 2010 | 0 | -2.13  (-2.32, -1.99) |
| Prevalence | Saudi Arabia | Female | Glaucoma | 2010 | 2018 | 0 | -1.47  (-1.54, -1.39) |
| Prevalence | Madagascar | Female | Glaucoma | 2019 | 2021 | 0.226754649 | -0.12  (-0.45, 0.08) |
| Prevalence | Saudi Arabia | Male | Glaucoma | 1990 | 2000 | 0 | -1.98  (-2.04, -1.91) |
| Prevalence | Saudi Arabia | Male | Glaucoma | 2000 | 2005 | 0 | 2.38  (2.24, 2.5) |
| Prevalence | Saudi Arabia | Male | Glaucoma | 2005 | 2015 | 0 | -2.21  (-2.31, -2.13) |
| Prevalence | Bosnia and Herzegovina | Female | Glaucoma | 2018 | 2021 | 0.176364727 | -0.12  (-0.37, 0.26) |
| Prevalence | Senegal | Both | Glaucoma | 1990 | 2000 | 0.076784643 | -0.11  (-0.21, 0.04) |
| Prevalence | Senegal | Both | Glaucoma | 2000 | 2013 | 0.033593281 | -0.48  (-0.55, -0.18) |
| Prevalence | Senegal | Both | Glaucoma | 2013 | 2019 | 0.00039992 | -0.88  (-1.07, -0.48) |
| Prevalence | Romania | Both | Glaucoma | 2017 | 2021 | 0.00159968 | -0.23  (-0.29, -0.14) |
| Prevalence | Senegal | Female | Glaucoma | 1990 | 2000 | 0.299940012 | 0.05  (-0.13, 0.2) |
| Prevalence | Senegal | Female | Glaucoma | 2000 | 2007 | 0.058788242 | -0.53  (-1.01, 0.14) |
| Prevalence | Senegal | Female | Glaucoma | 2007 | 2015 | 0.175164967 | -0.24  (-0.64, 0.28) |
| Prevalence | Iceland | Female | Glaucoma | 2018 | 2021 | 0.352329534 | -0.12  (-0.44, 0.37) |
| Prevalence | Senegal | Male | Glaucoma | 1990 | 1994 | 0 | -0.56  (-1, -0.36) |
| Prevalence | Senegal | Male | Glaucoma | 1994 | 2000 | 0.762647471 | -0.03  (-0.15, 0.3) |
| Prevalence | Senegal | Male | Glaucoma | 2000 | 2010 | 0 | -0.48  (-0.56, -0.4) |
| Prevalence | Senegal | Male | Glaucoma | 2010 | 2019 | 0 | -0.87  (-0.95, -0.79) |
| Prevalence | Netherlands | Female | Glaucoma | 2019 | 2021 | 0.212757449 | -0.12  (-0.6, 0.1) |
| Prevalence | Serbia | Both | Glaucoma | 1990 | 1995 | 0 | -0.67  (-0.79, -0.61) |
| Prevalence | Serbia | Both | Glaucoma | 1995 | 2000 | 0 | -0.42  (-0.47, -0.34) |
| Prevalence | Serbia | Both | Glaucoma | 2000 | 2010 | 0 | -1.34  (-1.36, -1.32) |
| Prevalence | Serbia | Both | Glaucoma | 2010 | 2017 | 0 | -0.79  (-0.83, -0.75) |
| Prevalence | Albania | Both | Glaucoma | 2018 | 2021 | 0.00719856 | -0.23  (-0.3, -0.16) |
| Prevalence | Serbia | Female | Glaucoma | 1990 | 1995 | 0 | -0.79  (-0.91, -0.71) |
| Prevalence | Serbia | Female | Glaucoma | 1995 | 2000 | 0.00079984 | -0.2  (-0.67, -0.13) |
| Prevalence | Serbia | Female | Glaucoma | 2000 | 2010 | 0 | -1.37  (-1.41, -0.2) |
| Prevalence | Serbia | Female | Glaucoma | 2010 | 2014 | 0 | -1.03  (-1.4, -0.94) |
| Prevalence | Serbia | Female | Glaucoma | 2014 | 2018 | 0 | -0.83  (-0.96, -0.65) |
| Prevalence | Paraguay | Male | Glaucoma | 2019 | 2021 | 0.118776245 | -0.12  (-0.47, 0.06) |
| Prevalence | Serbia | Male | Glaucoma | 1990 | 1996 | 0.00079984 | -0.34  (-0.41, -0.25) |
| Prevalence | Serbia | Male | Glaucoma | 1996 | 2001 | 0 | -0.78  (-0.92, -0.66) |
| Prevalence | Serbia | Male | Glaucoma | 2001 | 2009 | 0 | -1.47  (-1.53, -1.42) |
| Prevalence | Serbia | Male | Glaucoma | 2009 | 2013 | 0 | -0.89  (-1.18, -0.7) |
| Prevalence | Venezuela (Bolivarian Republic of) | Male | Glaucoma | 2010 | 2021 | 0.00039992 | -0.11  (-0.16, -0.07) |
| Prevalence | Seychelles | Both | Glaucoma | 1990 | 1999 | 0 | -1.24  (-1.28, -1.21) |
| Prevalence | Seychelles | Both | Glaucoma | 1999 | 2015 | 0 | -0.74  (-0.75, -0.72) |
| Prevalence | Seychelles | Both | Glaucoma | 2015 | 2018 | 0 | -1.18  (-1.27, -1.01) |
| Prevalence | Argentina | Both | Glaucoma | 2019 | 2021 | 0 | -0.23  (-0.39, -0.12) |
| Prevalence | Seychelles | Female | Glaucoma | 1990 | 2000 | 0 | -1.15  (-1.21, -1.1) |
| Prevalence | Seychelles | Female | Glaucoma | 2000 | 2015 | 0 | -0.7  (-0.73, -0.67) |
| Prevalence | Seychelles | Female | Glaucoma | 2015 | 2018 | 0 | -1.27  (-1.4, -1.01) |
| Prevalence | Serbia | Female | Glaucoma | 2018 | 2021 | 0.129174165 | -0.11  (-0.24, 0.1) |
| Prevalence | Seychelles | Male | Glaucoma | 1990 | 1995 | 0 | -1.35  (-1.41, -1.31) |
| Prevalence | Seychelles | Male | Glaucoma | 1995 | 2000 | 0 | -1.18  (-1.21, -1.12) |
| Prevalence | Seychelles | Male | Glaucoma | 2000 | 2005 | 0 | -0.69  (-0.72, -0.64) |
| Prevalence | Seychelles | Male | Glaucoma | 2005 | 2015 | 0 | -0.86  (-0.87, -0.85) |
| Prevalence | Seychelles | Male | Glaucoma | 2015 | 2019 | 0 | -1.06  (-1.13, -1.02) |
| Prevalence | Nauru | Female | Glaucoma | 2015 | 2021 | 0.525894821 | -0.11  (-0.5, 0.52) |
| Prevalence | Sierra Leone | Both | Glaucoma | 1990 | 2001 | 0.171965607 | 0.06  (-0.03, 0.17) |
| Prevalence | Sierra Leone | Both | Glaucoma | 2001 | 2009 | 0 | -0.61  (-0.78, -0.44) |
| Prevalence | Zimbabwe | Both | Glaucoma | 2009 | 2021 | 0.00039992 | -0.22  (-0.25, -0.19) |
| Prevalence | Sierra Leone | Female | Glaucoma | 1990 | 2000 | 0.349530094 | 0.04  (-0.15, 0.17) |
| Prevalence | Sierra Leone | Female | Glaucoma | 2000 | 2010 | 0.073185363 | -0.44  (-0.64, 0.24) |
| Prevalence | Sierra Leone | Female | Glaucoma | 2010 | 2014 | 0.020395921 | -1.27  (-1.6, -0.24) |
| Prevalence | Bangladesh | Female | Glaucoma | 2019 | 2021 | 0.629074185 | -0.07  (-0.73, 0.43) |
| Prevalence | Sierra Leone | Male | Glaucoma | 1990 | 1995 | 0.037992402 | -0.29  (-1.01, -0.02) |
| Prevalence | Sierra Leone | Male | Glaucoma | 1995 | 2000 | 0.019596081 | 0.49  (0.22, 1.01) |
| Prevalence | Sierra Leone | Male | Glaucoma | 2000 | 2008 | 0.0019996 | -0.57  (-0.74, -0.41) |
| Prevalence | El Salvador | Female | Glaucoma | 2019 | 2021 | 0.146370726 | -0.07  (-0.25, 0.02) |
| Prevalence | Singapore | Both | Glaucoma | 1990 | 1994 | 0 | -1.51  (-1.68, -1.4) |
| Prevalence | Singapore | Both | Glaucoma | 1994 | 2002 | 0 | -0.87  (-0.91, -0.81) |
| Prevalence | Singapore | Both | Glaucoma | 2002 | 2010 | 0 | -1.18  (-1.23, -1.13) |
| Prevalence | Singapore | Both | Glaucoma | 2010 | 2017 | 0 | -0.37  (-1, -0.34) |
| Prevalence | Austria | Both | Glaucoma | 2019 | 2021 | 0.030393921 | -0.21  (-0.5, -0.04) |
| Prevalence | Singapore | Female | Glaucoma | 1990 | 1995 | 0 | -1.32  (-1.48, -1.21) |
| Prevalence | Singapore | Female | Glaucoma | 1995 | 2005 | 0 | -0.84  (-0.88, -0.72) |
| Prevalence | Singapore | Female | Glaucoma | 2005 | 2010 | 0 | -1.15  (-1.34, -1) |
| Prevalence | Singapore | Female | Glaucoma | 2010 | 2017 | 0 | -0.31  (-0.53, -0.25) |
| Prevalence | Kenya | Male | Glaucoma | 2014 | 2021 | 0.586282743 | -0.07  (-0.26, 0.23) |
| Prevalence | Singapore | Male | Glaucoma | 1990 | 1992 | 0 | -1.84  (-2, -1.58) |
| Prevalence | Singapore | Male | Glaucoma | 1992 | 1995 | 0 | -1.45  (-1.53, -0.69) |
| Prevalence | Singapore | Male | Glaucoma | 1995 | 2000 | 0 | -0.73  (-1.39, -0.64) |
| Prevalence | Singapore | Male | Glaucoma | 2000 | 2009 | 0 | -1.38  (-1.42, -0.67) |
| Prevalence | Singapore | Male | Glaucoma | 2009 | 2012 | 0 | -0.65  (-1.25, -0.42) |
| Prevalence | Norway | Male | Glaucoma | 2009 | 2021 | 0.00639872 | -0.07  (-0.09, -0.05) |
| Prevalence | Slovakia | Both | Glaucoma | 1990 | 1994 | 0 | -1.34  (-1.39, -1.29) |
| Prevalence | Slovakia | Both | Glaucoma | 1994 | 2001 | 0 | -0.68  (-0.71, -0.65) |
| Prevalence | Slovakia | Both | Glaucoma | 2001 | 2005 | 0 | -1.06  (-1.16, -0.99) |
| Prevalence | Slovakia | Both | Glaucoma | 2005 | 2014 | 0 | -0.72  (-0.75, -0.69) |
| Prevalence | Slovakia | Both | Glaucoma | 2014 | 2018 | 0 | -0.5  (-0.57, -0.4) |
| Prevalence | Kenya | Both | Glaucoma | 2014 | 2021 | 0.153169366 | -0.21  (-0.39, 0.12) |
| Prevalence | Slovakia | Female | Glaucoma | 1990 | 1992 | 0 | -1.72  (-1.89, -1.5) |
| Prevalence | Slovakia | Female | Glaucoma | 1992 | 1995 | 0 | -1.23  (-1.31, -0.43) |
| Prevalence | Slovakia | Female | Glaucoma | 1995 | 2000 | 0.00119976 | -0.32  (-0.45, -0.22) |
| Prevalence | Slovakia | Female | Glaucoma | 2000 | 2005 | 0 | -0.97  (-1.08, -0.86) |
| Prevalence | Slovakia | Female | Glaucoma | 2005 | 2018 | 0 | -0.71  (-0.73, -0.68) |
| Prevalence | Germany | Female | Glaucoma | 2019 | 2021 | 0.552289542 | -0.05  (-0.34, 0.18) |
| Prevalence | Slovakia | Male | Glaucoma | 1990 | 1994 | 0 | -0.98  (-1.07, -0.93) |
| Prevalence | Slovakia | Male | Glaucoma | 1994 | 2000 | 0 | -0.9  (-1.03, -0.83) |
| Prevalence | Slovakia | Male | Glaucoma | 2000 | 2005 | 0 | -1.05  (-1.09, -0.72) |
| Prevalence | Slovakia | Male | Glaucoma | 2005 | 2015 | 0 | -0.69  (-0.7, -0.68) |
| Prevalence | Switzerland | Male | Glaucoma | 2019 | 2021 | 0.428714257 | -0.04  (-0.34, 0.09) |
| Prevalence | Slovenia | Both | Glaucoma | 1990 | 1994 | 0 | -0.83  (-0.93, -0.76) |
| Prevalence | Slovenia | Both | Glaucoma | 1994 | 2000 | 0 | -0.61  (-0.64, -0.56) |
| Prevalence | Slovenia | Both | Glaucoma | 2000 | 2005 | 0 | -1.37  (-1.4, -1.34) |
| Prevalence | Slovenia | Both | Glaucoma | 2005 | 2014 | 0 | -0.58  (-0.61, -0.56) |
| Prevalence | Slovenia | Both | Glaucoma | 2014 | 2018 | 0 | -0.33  (-0.44, -0.23) |
| Prevalence | Republic of Moldova | Both | Glaucoma | 2019 | 2021 | 0.00159968 | -0.19  (-0.42, -0.06) |
| Prevalence | Slovenia | Female | Glaucoma | 1990 | 1995 | 0 | -0.91  (-0.97, -0.85) |
| Prevalence | Slovenia | Female | Glaucoma | 1995 | 2000 | 0 | -0.34  (-0.39, -0.28) |
| Prevalence | Slovenia | Female | Glaucoma | 2000 | 2005 | 0 | -1.29  (-1.33, -1.25) |
| Prevalence | Slovenia | Female | Glaucoma | 2005 | 2018 | 0 | -0.63  (-0.64, -0.61) |
| Prevalence | Chile | Female | Glaucoma | 2019 | 2021 | 0.695860828 | -0.02  (-0.22, 0.12) |
| Prevalence | Slovenia | Male | Glaucoma | 1990 | 1995 | 0 | -0.53  (-0.6, -0.4) |
| Prevalence | Slovenia | Male | Glaucoma | 1995 | 2000 | 0 | -0.83  (-0.97, -0.75) |
| Prevalence | Slovenia | Male | Glaucoma | 2000 | 2005 | 0 | -1.5  (-1.58, -1.44) |
| Prevalence | Slovenia | Male | Glaucoma | 2005 | 2015 | 0 | -0.65  (-0.68, -0.62) |
| Prevalence | Slovenia | Male | Glaucoma | 2015 | 2019 | 0.446310738 | -0.03  (-0.14, 0.14) |
| Prevalence | Tokelau | Female | Glaucoma | 2014 | 2021 | 0.841831634 | -0.02  (-0.26, 0.36) |
| Prevalence | Solomon Islands | Both | Glaucoma | 1990 | 1998 | 0 | -0.9  (-1.18, -0.77) |
| Prevalence | Solomon Islands | Both | Glaucoma | 1998 | 2007 | 0.029994001 | -0.37  (-0.48, -0.06) |
| Prevalence | Solomon Islands | Both | Glaucoma | 2007 | 2015 | 0 | -1.22  (-1.55, -1.09) |
| Prevalence | Croatia | Both | Glaucoma | 2018 | 2021 | 0.00239952 | -0.19  (-0.25, -0.12) |
| Prevalence | Solomon Islands | Female | Glaucoma | 1990 | 1996 | 0.00159968 | -0.82  (-2.13, -0.38) |
| Prevalence | Solomon Islands | Female | Glaucoma | 1996 | 2008 | 0.600679864 | -0.07  (-0.22, 0.68) |
| Prevalence | Solomon Islands | Female | Glaucoma | 2008 | 2015 | 0.00839832 | -1.37  (-2.56, -0.98) |
| Prevalence | Zimbabwe | Female | Glaucoma | 2014 | 2021 | 0.375524895 | -0.02  (-0.07, 0.06) |
| Prevalence | Solomon Islands | Male | Glaucoma | 1990 | 2000 | 0 | -0.92  (-0.94, -0.9) |
| Prevalence | Solomon Islands | Male | Glaucoma | 2000 | 2005 | 0 | -0.25  (-0.28, -0.22) |
| Prevalence | Solomon Islands | Male | Glaucoma | 2005 | 2015 | 0 | -1.01  (-1.03, -0.99) |
| Prevalence | Jamaica | Female | Glaucoma | 2019 | 2021 | 0.882223555 | -0.01  (-0.3, 0.2) |
| Prevalence | Somalia | Both | Glaucoma | 1990 | 2001 | 0 | -0.12  (-0.14, -0.1) |
| Prevalence | Somalia | Both | Glaucoma | 2001 | 2010 | 0 | -0.75  (-0.78, -0.73) |
| Prevalence | Somalia | Both | Glaucoma | 2010 | 2015 | 0.00039992 | 0.81  (0.76, 0.89) |
| Prevalence | Somalia | Both | Glaucoma | 2015 | 2019 | 0.00359928 | 0.25  (0.13, 0.37) |
| Prevalence | Taiwan (Province of China) | Both | Glaucoma | 2014 | 2021 | 0.133973205 | -0.19  (-0.45, 0.06) |
| Prevalence | Somalia | Female | Glaucoma | 1990 | 1994 | 0.0039992 | -0.12  (-0.29, -0.04) |
| Prevalence | Somalia | Female | Glaucoma | 1994 | 2001 | 0.014797041 | 0.05  (0.02, 0.11) |
| Prevalence | Somalia | Female | Glaucoma | 2001 | 2010 | 0.00359928 | -0.62  (-0.65, -0.6) |
| Prevalence | Somalia | Female | Glaucoma | 2010 | 2016 | 0.00479904 | 0.78  (0.75, 0.83) |
| Prevalence | Somalia | Female | Glaucoma | 2016 | 2019 | 0 | 0.31  (0.22, 0.51) |
| Prevalence | Singapore | Female | Glaucoma | 2017 | 2021 | 0.925014997 | -0.01  (-0.16, 0.3) |
| Prevalence | Somalia | Male | Glaucoma | 1990 | 1995 | 0 | 0.18  (0.11, 0.28) |
| Prevalence | Somalia | Male | Glaucoma | 1995 | 2001 | 0 | -0.19  (-0.26, -0.13) |
| Prevalence | Somalia | Male | Glaucoma | 2001 | 2010 | 0 | -0.74  (-0.77, -0.72) |
| Prevalence | Somalia | Male | Glaucoma | 2010 | 2015 | 0.00079984 | 0.89  (0.84, 0.93) |
| Prevalence | Somalia | Male | Glaucoma | 2015 | 2019 | 0.903019396 | 0  (-0.06, 0.1) |
| Prevalence | Argentina | Female | Glaucoma | 2019 | 2021 | 0.981003799 | 0  (-0.15, 0.11) |
| Prevalence | South Africa | Both | Glaucoma | 1990 | 2001 | 0.00039992 | -0.49  (-0.82, -0.3) |
| Prevalence | South Africa | Both | Glaucoma | 2001 | 2005 | 0.00559888 | 1.3  (0.35, 2.32) |
| Prevalence | South Africa | Both | Glaucoma | 2005 | 2011 | 0.00119976 | -3.72  (-4.45, -3.26) |
| Prevalence | South Africa | Both | Glaucoma | 2011 | 2019 | 0.00119976 | -0.77  (-1.09, -0.43) |
| Prevalence | Australia | Both | Glaucoma | 2019 | 2021 | 0.0079984 | -0.18  (-0.45, -0.05) |
| Prevalence | South Africa | Female | Glaucoma | 1990 | 2001 | 0.00119976 | -0.54  (-1.01, -0.33) |
| Prevalence | South Africa | Female | Glaucoma | 2001 | 2005 | 0.027994401 | 1.23  (0.12, 2.45) |
| Prevalence | South Africa | Female | Glaucoma | 2005 | 2011 | 0.00639872 | -3.56  (-4.65, -3.01) |
| Prevalence | South Africa | Female | Glaucoma | 2011 | 2019 | 0.0059988 | -0.74  (-1.1, -0.34) |
| Prevalence | Cameroon | Female | Glaucoma | 2014 | 2021 | 0.916216757 | 0  (-0.1, 0.13) |
| Prevalence | South Africa | Male | Glaucoma | 1990 | 2001 | 0.014797041 | -0.19  (-0.41, -0.04) |
| Prevalence | South Africa | Male | Glaucoma | 2001 | 2005 | 0.00079984 | 1.43  (0.82, 2.16) |
| Prevalence | South Africa | Male | Glaucoma | 2005 | 2011 | 0 | -3.84  (-4.32, -3.51) |
| Prevalence | South Africa | Male | Glaucoma | 2011 | 2019 | 0 | -0.93  (-1.15, -0.68) |
| Prevalence | North Macedonia | Female | Glaucoma | 2018 | 2021 | 0.693061388 | 0.01  (-0.06, 0.08) |
| Prevalence | South Sudan | Both | Glaucoma | 1990 | 1994 | 0 | -1.73  (-2.58, -1.38) |
| Prevalence | South Sudan | Both | Glaucoma | 1994 | 2015 | 0 | -0.89  (-0.93, -0.85) |
| Prevalence | Singapore | Both | Glaucoma | 2017 | 2021 | 0.048790242 | -0.18  (-0.3, 0) |
| Prevalence | South Sudan | Female | Glaucoma | 1990 | 1994 | 0 | -1.92  (-2.08, -1.78) |
| Prevalence | South Sudan | Female | Glaucoma | 1994 | 1998 | 0 | -0.91  (-1.12, -0.77) |
| Prevalence | South Sudan | Female | Glaucoma | 1998 | 2008 | 0 | -0.48  (-0.52, -0.39) |
| Prevalence | South Sudan | Female | Glaucoma | 2008 | 2015 | 0 | -0.75  (-0.88, -0.68) |
| Prevalence | South Sudan | Female | Glaucoma | 2015 | 2019 | 0 | 0.32  (0.15, 0.53) |
| Prevalence | Barbados | Female | Glaucoma | 2019 | 2021 | 0.959008198 | 0.02  (-0.36, 0.19) |
| Prevalence | South Sudan | Male | Glaucoma | 1990 | 1993 | 0 | -1.79  (-2.68, -1.12) |
| Prevalence | South Sudan | Male | Glaucoma | 1993 | 2016 | 0.013597281 | -1.06  (-1.11, -0.9) |
| Prevalence | Romania | Female | Glaucoma | 2018 | 2021 | 0.653869226 | 0.02  (-0.1, 0.2) |
| Prevalence | Spain | Both | Glaucoma | 1990 | 1994 | 0 | -2.35  (-2.7, -2.18) |
| Prevalence | Spain | Both | Glaucoma | 1994 | 1999 | 0 | -1.87  (-1.97, -1.6) |
| Prevalence | Spain | Both | Glaucoma | 1999 | 2006 | 0 | -0.6  (-0.66, -0.48) |
| Prevalence | Spain | Both | Glaucoma | 2006 | 2009 | 0 | -1.21  (-1.31, -0.95) |
| Prevalence | Japan | Both | Glaucoma | 2015 | 2021 | 0.00119976 | -0.16  (-0.2, -0.13) |
| Prevalence | Spain | Female | Glaucoma | 1990 | 1999 | 0 | -2.07  (-2.11, -2.02) |
| Prevalence | Spain | Female | Glaucoma | 1999 | 2005 | 0.00039992 | -0.43  (-0.51, -0.35) |
| Prevalence | Spain | Female | Glaucoma | 2005 | 2010 | 0 | -1.15  (-1.34, -1.03) |
| Prevalence | Spain | Female | Glaucoma | 2010 | 2015 | 0.0019996 | -0.44  (-0.55, -0.26) |
| Prevalence | Spain | Female | Glaucoma | 2015 | 2019 | 0 | -1.36  (-1.6, -1.22) |
| Prevalence | Cook Islands | Female | Glaucoma | 2014 | 2021 | 0.691861628 | 0.03  (-0.31, 0.91) |
| Prevalence | Spain | Male | Glaucoma | 1990 | 1994 | 0 | -2.62  (-2.97, -2.41) |
| Prevalence | Spain | Male | Glaucoma | 1994 | 1999 | 0 | -1.66  (-1.91, -1.41) |
| Prevalence | Spain | Male | Glaucoma | 1999 | 2012 | 0 | -0.83  (-0.87, -0.79) |
| Prevalence | Brunei Darussalam | Female | Glaucoma | 2012 | 2021 | 0.298740252 | 0.03  (-0.04, 0.13) |
| Prevalence | Sri Lanka | Both | Glaucoma | 1990 | 1995 | 0 | -1.04  (-1.2, -0.89) |
| Prevalence | Sri Lanka | Both | Glaucoma | 1995 | 2000 | 0 | 0.4  (0.28, 0.55) |
| Prevalence | Sri Lanka | Both | Glaucoma | 2000 | 2015 | 0 | -1.01  (-1.04, -0.98) |
| Prevalence | Sri Lanka | Both | Glaucoma | 2015 | 2018 | 0 | -2.75  (-2.94, -2.37) |
| Prevalence | Burundi | Both | Glaucoma | 1990 | 2021 | 0 | -0.15  (-0.18, -0.12) |
| Prevalence | Sri Lanka | Female | Glaucoma | 1990 | 1995 | 0 | -0.88  (-1.14, -0.7) |
| Prevalence | Sri Lanka | Female | Glaucoma | 1995 | 2001 | 0.699860028 | -0.02  (-0.17, 0.27) |
| Prevalence | Sri Lanka | Female | Glaucoma | 2001 | 2015 | 0 | -1.02  (-1.06, -0.98) |
| Prevalence | Sri Lanka | Female | Glaucoma | 2015 | 2018 | 0 | -2.41  (-2.61, -1.99) |
| Prevalence | Republic of Moldova | Female | Glaucoma | 2019 | 2021 | 0.618276345 | 0.04  (-0.19, 0.19) |
| Prevalence | Sri Lanka | Male | Glaucoma | 1990 | 1995 | 0 | -1.04  (-1.15, -0.92) |
| Prevalence | Sri Lanka | Male | Glaucoma | 1995 | 2000 | 0 | 0.78  (0.69, 0.87) |
| Prevalence | Sri Lanka | Male | Glaucoma | 2000 | 2015 | 0 | -0.93  (-0.96, -0.9) |
| Prevalence | Sri Lanka | Male | Glaucoma | 2015 | 2019 | 0 | -2.76  (-2.91, -2.63) |
| Prevalence | Ukraine | Female | Glaucoma | 2019 | 2021 | 0.626674665 | 0.04  (-0.17, 0.19) |
| Prevalence | Sudan | Both | Glaucoma | 1990 | 1994 | 0.01079784 | -1.35  (-3.21, -0.46) |
| Prevalence | Sudan | Both | Glaucoma | 1994 | 2001 | 0.015196961 | 0.68  (0.29, 2.09) |
| Prevalence | Sudan | Both | Glaucoma | 2001 | 2019 | 0.00079984 | -1.8  (-1.89, -1.69) |
| Prevalence | Nigeria | Both | Glaucoma | 2015 | 2021 | 0.177564487 | -0.14  (-0.31, 0.08) |
| Prevalence | Sudan | Female | Glaucoma | 1990 | 1995 | 0.029194161 | -0.9  (-2.55, -0.2) |
| Prevalence | Sudan | Female | Glaucoma | 1995 | 2000 | 0.027594481 | 1.21  (0.5, 2.72) |
| Prevalence | Sudan | Female | Glaucoma | 2000 | 2019 | 0.0039992 | -1.61  (-1.71, -1.51) |
| Prevalence | Tonga | Female | Glaucoma | 2015 | 2021 | 0.825034993 | 0.04  (-0.44, 0.95) |
| Prevalence | Sudan | Male | Glaucoma | 1990 | 1994 | 0.00079984 | -1.62  (-3.41, -0.75) |
| Prevalence | Sudan | Male | Glaucoma | 1994 | 2001 | 0.025194961 | 0.47  (0.08, 1.83) |
| Prevalence | Sudan | Male | Glaucoma | 2001 | 2019 | 0.00039992 | -1.93  (-2.02, -1.82) |
| Prevalence | South Sudan | Male | Glaucoma | 2016 | 2021 | 0.775044991 | 0.04  (-0.34, 0.7) |
| Prevalence | Suriname | Both | Glaucoma | 1990 | 1996 | 0 | -0.58  (-0.63, -0.53) |
| Prevalence | Suriname | Both | Glaucoma | 1996 | 2000 | 0 | -1.13  (-1.23, -1.01) |
| Prevalence | Suriname | Both | Glaucoma | 2000 | 2005 | 0 | -1.63  (-1.69, -1.58) |
| Prevalence | Suriname | Both | Glaucoma | 2005 | 2010 | 0 | -0.7  (-0.76, -0.64) |
| Prevalence | Suriname | Both | Glaucoma | 2010 | 2015 | 0 | -1.03  (-1.12, -0.98) |
| Prevalence | Germany | Both | Glaucoma | 2019 | 2021 | 0.129174165 | -0.14  (-0.47, 0.08) |
| Prevalence | Suriname | Female | Glaucoma | 1990 | 1996 | 0 | -0.38  (-0.51, -0.27) |
| Prevalence | Suriname | Female | Glaucoma | 1996 | 2005 | 0 | -1.44  (-1.51, -1.38) |
| Prevalence | Suriname | Female | Glaucoma | 2005 | 2014 | 0 | -0.92  (-0.99, -0.85) |
| Prevalence | Trinidad and Tobago | Female | Glaucoma | 2018 | 2021 | 0.378724255 | 0.04  (-0.07, 0.14) |
| Prevalence | Suriname | Male | Glaucoma | 1990 | 2000 | 0 | -0.79  (-0.88, -0.72) |
| Prevalence | Suriname | Male | Glaucoma | 2000 | 2005 | 0 | -1.67  (-1.83, -0.72) |
| Prevalence | Suriname | Male | Glaucoma | 2005 | 2010 | 0.00959808 | -0.4  (-1.71, -0.24) |
| Prevalence | Suriname | Male | Glaucoma | 2010 | 2015 | 0.00119976 | -1.19  (-1.39, -0.36) |
| Prevalence | Suriname | Male | Glaucoma | 2015 | 2019 | 0 | -0.49  (-1.25, -0.25) |
| Prevalence | Oman | Female | Glaucoma | 2014 | 2021 | 0.021995601 | 0.06  (0.01, 0.11) |
| Prevalence | Sweden | Both | Glaucoma | 1990 | 2000 | 0.034393121 | -0.47  (-1.52, -0.14) |
| Prevalence | Sweden | Both | Glaucoma | 2000 | 2009 | 0.431513697 | 0.1  (-0.99, 1.18) |
| Prevalence | Cameroon | Both | Glaucoma | 2014 | 2021 | 0.293141372 | -0.13  (-0.28, 0.27) |
| Prevalence | Dominica | Female | Glaucoma | 2019 | 2021 | 0.222355529 | 0.06  (-0.07, 0.16) |
| Prevalence | Sweden | Male | Glaucoma | 1990 | 1994 | 0 | -2.32  (-3.72, -1.63) |
| Prevalence | Sweden | Male | Glaucoma | 1994 | 2000 | 0.033193361 | 0.46  (0.06, 1.71) |
| Prevalence | Sweden | Male | Glaucoma | 2000 | 2005 | 0.046790642 | -0.62  (-1.64, -0.11) |
| Prevalence | Sweden | Male | Glaucoma | 2005 | 2010 | 0.044391122 | 1.2  (0.48, 2.28) |
| Prevalence | Armenia | Male | Glaucoma | 2019 | 2021 | 0.297140572 | 0.07  (-0.09, 0.2) |
| Prevalence | Switzerland | Both | Glaucoma | 1990 | 1998 | 0 | -0.9  (-1.04, -0.77) |
| Prevalence | Switzerland | Both | Glaucoma | 1998 | 2004 | 0 | -0.78  (-0.91, -0.63) |
| Prevalence | Switzerland | Both | Glaucoma | 2004 | 2009 | 0 | -0.6  (-0.72, -0.37) |
| Prevalence | Switzerland | Both | Glaucoma | 2009 | 2016 | 0 | -0.31  (-0.35, -0.14) |
| Prevalence | Switzerland | Both | Glaucoma | 2016 | 2019 | 0 | -0.54  (-0.66, -0.43) |
| Prevalence | South Sudan | Both | Glaucoma | 2015 | 2021 | 0.276344731 | -0.13  (-0.34, 0.23) |
| Prevalence | Switzerland | Female | Glaucoma | 1990 | 1999 | 0 | -0.88  (-0.97, -0.83) |
| Prevalence | Switzerland | Female | Glaucoma | 1999 | 2008 | 0 | -0.72  (-0.8, -0.62) |
| Prevalence | Switzerland | Female | Glaucoma | 2008 | 2016 | 0 | -0.37  (-0.72, -0.22) |
| Prevalence | Switzerland | Female | Glaucoma | 2016 | 2019 | 0 | -0.64  (-0.76, -0.35) |
| Prevalence | Tuvalu | Female | Glaucoma | 2014 | 2021 | 0.281543691 | 0.11  (-0.18, 0.81) |
| Prevalence | Switzerland | Male | Glaucoma | 1990 | 2003 | 0 | -0.91  (-0.96, -0.89) |
| Prevalence | Switzerland | Male | Glaucoma | 2003 | 2008 | 0 | -0.67  (-0.92, -0.49) |
| Prevalence | Switzerland | Male | Glaucoma | 2008 | 2019 | 0 | -0.35  (-0.65, -0.32) |
| Prevalence | Mexico | Female | Glaucoma | 2019 | 2021 | 0.016796641 | 0.12  (0.02, 0.23) |
| Prevalence | Syrian Arab Republic | Both | Glaucoma | 1990 | 2000 | 0 | -1.82  (-1.85, -1.8) |
| Prevalence | Syrian Arab Republic | Both | Glaucoma | 2000 | 2009 | 0 | -2.03  (-2.06, -2) |
| Prevalence | Syrian Arab Republic | Both | Glaucoma | 2009 | 2012 | 0 | -0.91  (-1, -0.83) |
| Prevalence | Syrian Arab Republic | Both | Glaucoma | 2012 | 2015 | 0 | -0.53  (-0.68, -0.44) |
| Prevalence | Canada | Both | Glaucoma | 2004 | 2021 | 0 | -0.13  (-0.15, -0.11) |
| Prevalence | Syrian Arab Republic | Female | Glaucoma | 1990 | 1999 | 0 | -1.69  (-1.72, -1.66) |
| Prevalence | Syrian Arab Republic | Female | Glaucoma | 1999 | 2009 | 0 | -1.96  (-1.98, -1.9) |
| Prevalence | Syrian Arab Republic | Female | Glaucoma | 2009 | 2012 | 0 | -0.67  (-1.98, -0.61) |
| Prevalence | Syrian Arab Republic | Female | Glaucoma | 2012 | 2015 | 0 | -0.42  (-0.55, -0.35) |
| Prevalence | Syrian Arab Republic | Female | Glaucoma | 2015 | 2018 | 0 | -1.17  (-1.25, -1.08) |
| Prevalence | Belize | Female | Glaucoma | 2019 | 2021 | 0.038392322 | 0.12  (0.01, 0.22) |
| Prevalence | Syrian Arab Republic | Male | Glaucoma | 1990 | 2005 | 0 | -2.06  (-2.08, -2.04) |
| Prevalence | Syrian Arab Republic | Male | Glaucoma | 2005 | 2009 | 0 | -2.22  (-2.32, -2.07) |
| Prevalence | Syrian Arab Republic | Male | Glaucoma | 2009 | 2012 | 0 | -0.76  (-2.22, -0.66) |
| Prevalence | Syrian Arab Republic | Male | Glaucoma | 2012 | 2015 | 0 | -0.44  (-0.65, -0.36) |
| Prevalence | Lithuania | Male | Glaucoma | 2019 | 2021 | 0.121975605 | 0.14  (-0.08, 0.3) |
| Prevalence | Taiwan (Province of China) | Both | Glaucoma | 1990 | 1996 | 0.00359928 | -0.7  (-0.97, -0.4) |
| Prevalence | Taiwan (Province of China) | Both | Glaucoma | 1996 | 1999 | 0 | -5.07  (-5.41, -4.58) |
| Prevalence | Taiwan (Province of China) | Both | Glaucoma | 1999 | 2011 | 0 | -0.51  (-0.6, -0.42) |
| Prevalence | Taiwan (Province of China) | Both | Glaucoma | 2011 | 2014 | 0 | -5.78  (-6.2, -5.38) |
| Prevalence | Iceland | Both | Glaucoma | 2018 | 2021 | 0.325934813 | -0.13  (-0.4, 0.29) |
| Prevalence | Taiwan (Province of China) | Female | Glaucoma | 1990 | 1996 | 0.00559888 | -0.7  (-1.04, -0.33) |
| Prevalence | Taiwan (Province of China) | Female | Glaucoma | 1996 | 2000 | 0 | -4.12  (-4.64, -3.59) |
| Prevalence | Taiwan (Province of China) | Female | Glaucoma | 2000 | 2011 | 0 | -0.67  (-0.78, -0.55) |
| Prevalence | Taiwan (Province of China) | Female | Glaucoma | 2011 | 2014 | 0 | -6.04  (-6.48, -5.6) |
| Prevalence | San Marino | Female | Glaucoma | 2019 | 2021 | 0.139172166 | 0.14  (-0.09, 0.3) |
| Prevalence | Taiwan (Province of China) | Male | Glaucoma | 1990 | 1996 | 0 | -0.76  (-1.01, -0.48) |
| Prevalence | Taiwan (Province of China) | Male | Glaucoma | 1996 | 1999 | 0 | -5.23  (-5.58, -4.81) |
| Prevalence | Taiwan (Province of China) | Male | Glaucoma | 1999 | 2010 | 0.018396321 | -0.11  (-0.21, -0.02) |
| Prevalence | Taiwan (Province of China) | Male | Glaucoma | 2010 | 2015 | 0 | -4.01  (-4.28, -3.77) |
| Prevalence | Czechia | Female | Glaucoma | 2018 | 2021 | 0.029594081 | 0.14  (0.03, 0.27) |
| Prevalence | Tajikistan | Both | Glaucoma | 1990 | 1993 | 0 | -0.82  (-1.15, -0.63) |
| Prevalence | Tajikistan | Both | Glaucoma | 1993 | 1996 | 0.859028194 | -0.01  (-0.34, 0.47) |
| Prevalence | Tajikistan | Both | Glaucoma | 1996 | 2000 | 0.022395521 | 0.9  (0.79, 1.09) |
| Prevalence | Tajikistan | Both | Glaucoma | 2000 | 2005 | 0 | -0.46  (-0.59, -0.38) |
| Prevalence | Tajikistan | Both | Glaucoma | 2005 | 2015 | 0 | -1.08  (-1.14, -1.04) |
| Prevalence | Poland | Both | Glaucoma | 2017 | 2021 | 0.045990802 | -0.11  (-0.19, -0.01) |
| Prevalence | Tajikistan | Female | Glaucoma | 1990 | 1995 | 0 | -0.54  (-0.69, -0.41) |
| Prevalence | Tajikistan | Female | Glaucoma | 1995 | 2000 | 0.00079984 | 0.71  (0.58, 0.9) |
| Prevalence | Tajikistan | Female | Glaucoma | 2000 | 2005 | 0.00519896 | -0.59  (-0.73, -0.35) |
| Prevalence | Tajikistan | Female | Glaucoma | 2005 | 2013 | 0 | -1.05  (-1.3, -0.98) |
| Prevalence | United States of America | Female | Glaucoma | 2018 | 2021 | 0.123175365 | 0.15  (-0.32, 0.38) |
| Prevalence | Tajikistan | Male | Glaucoma | 1990 | 1995 | 0 | -0.56  (-0.67, -0.46) |
| Prevalence | Tajikistan | Male | Glaucoma | 1995 | 2000 | 0.014397121 | 0.84  (0.74, 0.93) |
| Prevalence | Tajikistan | Male | Glaucoma | 2000 | 2005 | 0.023995201 | -0.67  (-0.79, -0.53) |
| Prevalence | Tajikistan | Male | Glaucoma | 2005 | 2013 | 0 | -1.44  (-1.57, -1.36) |
| Prevalence | Tajikistan | Male | Glaucoma | 2013 | 2018 | 0 | -1.05  (-1.32, -0.88) |
| Prevalence | Bermuda | Female | Glaucoma | 2019 | 2021 | 0.00359928 | 0.16  (0.07, 0.23) |
| Prevalence | Thailand | Both | Glaucoma | 1990 | 1994 | 0 | -1.37  (-1.59, -1.23) |
| Prevalence | Thailand | Both | Glaucoma | 1994 | 2000 | 0 | -0.7  (-0.78, -0.6) |
| Prevalence | Thailand | Both | Glaucoma | 2000 | 2005 | 0 | -2.52  (-2.59, -1.15) |
| Prevalence | Thailand | Both | Glaucoma | 2005 | 2015 | 0 | -0.48  (-2.45, -0.43) |
| Prevalence | Thailand | Both | Glaucoma | 2015 | 2018 | 0 | -1.12  (-1.26, -0.44) |
| Prevalence | Cyprus | Both | Glaucoma | 2019 | 2021 | 0.146770646 | -0.11  (-0.42, 0.04) |
| Prevalence | Thailand | Female | Glaucoma | 1990 | 1994 | 0 | -1.28  (-1.63, -1.07) |
| Prevalence | Thailand | Female | Glaucoma | 1994 | 2000 | 0 | -0.62  (-0.75, -0.41) |
| Prevalence | Thailand | Female | Glaucoma | 2000 | 2005 | 0 | -2  (-2.14, -1.54) |
| Prevalence | Thailand | Female | Glaucoma | 2005 | 2010 | 0.073585283 | 0.17  (-1.9, 0.37) |
| Prevalence | Thailand | Female | Glaucoma | 2010 | 2014 | 0.055588882 | -0.47  (-0.76, 0.02) |
| Prevalence | Slovenia | Female | Glaucoma | 2018 | 2021 | 0.017996401 | 0.16  (0.05, 0.25) |
| Prevalence | Thailand | Male | Glaucoma | 1990 | 1995 | 0 | -1.37  (-1.65, -1.23) |
| Prevalence | Thailand | Male | Glaucoma | 1995 | 2000 | 0 | -0.71  (-0.85, -0.49) |
| Prevalence | Thailand | Male | Glaucoma | 2000 | 2005 | 0 | -3.09  (-3.24, -2.97) |
| Prevalence | Thailand | Male | Glaucoma | 2005 | 2009 | 0 | -1.25  (-1.63, -1.03) |
| Prevalence | Albania | Female | Glaucoma | 2019 | 2021 | 0.044391122 | 0.17  (0.01, 0.28) |
| Prevalence | Timor-Leste | Both | Glaucoma | 1990 | 2005 | 0 | -1.3  (-1.41, -1.17) |
| Prevalence | Timor-Leste | Both | Glaucoma | 2005 | 2010 | 0.00039992 | -3.7  (-4.55, -3.1) |
| Prevalence | Timor-Leste | Both | Glaucoma | 2010 | 2019 | 0.030393921 | -0.53  (-0.7, -0.13) |
| Prevalence | Kiribati | Both | Glaucoma | 2015 | 2021 | 0.4895021 | -0.09  (-0.37, 0.75) |
| Prevalence | Timor-Leste | Female | Glaucoma | 1990 | 2005 | 0 | -1.33  (-1.42, -1.23) |
| Prevalence | Timor-Leste | Female | Glaucoma | 2005 | 2010 | 0 | -3.37  (-3.95, -2.9) |
| Prevalence | Timor-Leste | Female | Glaucoma | 2010 | 2019 | 0.018796241 | -0.4  (-0.54, -0.14) |
| Prevalence | Mexico | Male | Glaucoma | 2019 | 2021 | 0.24555089 | 0.17  (-0.29, 0.38) |
| Prevalence | Timor-Leste | Male | Glaucoma | 1990 | 2005 | 0.00319936 | -1.39  (-1.59, -1.21) |
| Prevalence | Timor-Leste | Male | Glaucoma | 2005 | 2010 | 0.00239952 | -3.59  (-4.64, -1.22) |
| Prevalence | Timor-Leste | Male | Glaucoma | 2010 | 2016 | 0.00119976 | -0.9  (-3.8, -0.64) |
| Prevalence | Timor-Leste | Male | Glaucoma | 2016 | 2019 | 0.309538092 | 0.52  (-0.5, 1.31) |
| Prevalence | Marshall Islands | Female | Glaucoma | 2015 | 2021 | 0.293541292 | 0.18  (-0.24, 1.23) |
| Prevalence | Togo | Both | Glaucoma | 1990 | 1992 | 0 | 2.23  (2.03, 2.42) |
| Prevalence | Togo | Both | Glaucoma | 1992 | 1995 | 0 | 1.15  (1.04, 1.26) |
| Prevalence | Togo | Both | Glaucoma | 1995 | 2000 | 0.039992002 | -0.07  (-0.18, 0) |
| Prevalence | Togo | Both | Glaucoma | 2000 | 2005 | 0 | 0.41  (0.32, 0.58) |
| Prevalence | Togo | Both | Glaucoma | 2005 | 2010 | 0.810637872 | -0.01  (-0.13, 0.11) |
| Prevalence | Venezuela (Bolivarian Republic of) | Both | Glaucoma | 2009 | 2021 | 0.01239752 | -0.08  (-0.13, -0.03) |
| Prevalence | Togo | Female | Glaucoma | 1990 | 1992 | 0 | 2.26  (2.07, 2.43) |
| Prevalence | Togo | Female | Glaucoma | 1992 | 1995 | 0 | 1.19  (1.09, 1.29) |
| Prevalence | Togo | Female | Glaucoma | 1995 | 2000 | 0.560687862 | -0.02  (-0.11, 0.05) |
| Prevalence | Togo | Female | Glaucoma | 2000 | 2005 | 0 | 0.46  (0.38, 0.61) |
| Prevalence | Togo | Female | Glaucoma | 2005 | 2010 | 0.047190562 | 0.13  (0, 0.22) |
| Prevalence | Republic of Korea | Male | Glaucoma | 2019 | 2021 | 0.430713857 | 0.19  (-0.48, 0.5) |
| Prevalence | Togo | Male | Glaucoma | 1990 | 1992 | 0 | 2.18  (1.98, 2.38) |
| Prevalence | Togo | Male | Glaucoma | 1992 | 1995 | 0 | 1.17  (1.05, 1.28) |
| Prevalence | Togo | Male | Glaucoma | 1995 | 2000 | 0.318336333 | 0.03  (-0.08, 0.1) |
| Prevalence | Togo | Male | Glaucoma | 2000 | 2005 | 0 | 0.57  (0.48, 0.7) |
| Prevalence | Togo | Male | Glaucoma | 2005 | 2010 | 0.110777844 | 0.07  (-0.03, 0.15) |
| Prevalence | Lebanon | Female | Glaucoma | 2019 | 2021 | 0.141971606 | 0.2  (-0.06, 0.45) |
| Prevalence | Tokelau | Both | Glaucoma | 1990 | 2007 | 0 | -0.97  (-1, -0.92) |
| Prevalence | Tokelau | Both | Glaucoma | 2007 | 2015 | 0 | -1.43  (-1.84, -1.31) |
| Prevalence | Lithuania | Both | Glaucoma | 2019 | 2021 | 0.340731854 | -0.06  (-0.34, 0.15) |
| Prevalence | Tokelau | Female | Glaucoma | 1990 | 2010 | 0 | -0.78  (-0.83, -0.72) |
| Prevalence | Tokelau | Female | Glaucoma | 2010 | 2014 | 0 | -2.02  (-2.66, -1.46) |
| Prevalence | Sri Lanka | Male | Glaucoma | 2019 | 2021 | 0.276344731 | 0.2  (-0.24, 0.57) |
| Prevalence | Tokelau | Male | Glaucoma | 1990 | 2005 | 0 | -1.15  (-1.16, -1.14) |
| Prevalence | Tokelau | Male | Glaucoma | 2005 | 2010 | 0 | -1.46  (-1.53, -1.39) |
| Prevalence | Tokelau | Male | Glaucoma | 2010 | 2015 | 0 | -1.19  (-1.26, -1.1) |
| Prevalence | Tokelau | Male | Glaucoma | 2015 | 2019 | 0 | -1.52  (-1.62, -1.45) |
| Prevalence | Norway | Female | Glaucoma | 2019 | 2021 | 0.167966407 | 0.2  (-0.14, 0.37) |
| Prevalence | Tonga | Both | Glaucoma | 1990 | 2001 | 0.00559888 | -0.85  (-0.96, -0.75) |
| Prevalence | Tonga | Both | Glaucoma | 2001 | 2004 | 0.00039992 | -2.31  (-2.54, -0.78) |
| Prevalence | Tonga | Both | Glaucoma | 2004 | 2016 | 0 | -1.08  (-2.02, -0.97) |
| Prevalence | Czechia | Both | Glaucoma | 2017 | 2021 | 0.364727055 | -0.05  (-0.21, 0.24) |
| Prevalence | Tonga | Female | Glaucoma | 1990 | 2000 | 0.014397121 | -0.63  (-0.82, -0.31) |
| Prevalence | Tonga | Female | Glaucoma | 2000 | 2015 | 0.00039992 | -1.14  (-1.59, -1.02) |
| Prevalence | Taiwan (Province of China) | Male | Glaucoma | 2015 | 2021 | 0.167566487 | 0.21  (-0.1, 0.49) |
| Prevalence | Tonga | Male | Glaucoma | 1990 | 2000 | 0 | -0.99  (-1.02, -0.95) |
| Prevalence | Tonga | Male | Glaucoma | 2000 | 2005 | 0 | -2.15  (-2.22, -2.08) |
| Prevalence | Tonga | Male | Glaucoma | 2005 | 2015 | 0 | -1.14  (-1.18, -1.09) |
| Prevalence | Bhutan | Male | Glaucoma | 2018 | 2021 | 0.377524495 | 0.22  (-0.38, 1.16) |
| Prevalence | Trinidad and Tobago | Both | Glaucoma | 1990 | 1996 | 0 | -0.93  (-0.98, -0.83) |
| Prevalence | Trinidad and Tobago | Both | Glaucoma | 1996 | 2000 | 0 | -1.11  (-1.48, -1.04) |
| Prevalence | Trinidad and Tobago | Both | Glaucoma | 2000 | 2005 | 0 | -2.12  (-2.17, -2.06) |
| Prevalence | Trinidad and Tobago | Both | Glaucoma | 2005 | 2011 | 0 | -1.16  (-1.23, -1.11) |
| Prevalence | Trinidad and Tobago | Both | Glaucoma | 2011 | 2017 | 0 | -0.88  (-0.95, -0.79) |
| Prevalence | Belarus | Both | Glaucoma | 2018 | 2021 | 0.316736653 | -0.05  (-0.15, 0.05) |
| Prevalence | Trinidad and Tobago | Female | Glaucoma | 1990 | 2000 | 0 | -1.04  (-1.07, -1.02) |
| Prevalence | Trinidad and Tobago | Female | Glaucoma | 2000 | 2005 | 0 | -2.21  (-2.26, -2.16) |
| Prevalence | Trinidad and Tobago | Female | Glaucoma | 2005 | 2009 | 0 | -1.21  (-1.35, -1.13) |
| Prevalence | Trinidad and Tobago | Female | Glaucoma | 2009 | 2018 | 0 | -0.96  (-0.99, -0.92) |
| Prevalence | Croatia | Female | Glaucoma | 2019 | 2021 | 0.00719856 | 0.25  (0.15, 0.37) |
| Prevalence | Trinidad and Tobago | Male | Glaucoma | 1990 | 1996 | 0 | -0.92  (-1, -0.79) |
| Prevalence | Trinidad and Tobago | Male | Glaucoma | 1996 | 2000 | 0 | -1.17  (-1.51, -1.02) |
| Prevalence | Trinidad and Tobago | Male | Glaucoma | 2000 | 2005 | 0 | -2.02  (-2.17, -1.28) |
| Prevalence | Trinidad and Tobago | Male | Glaucoma | 2005 | 2008 | 0 | -1.29  (-1.95, -1.03) |
| Prevalence | Trinidad and Tobago | Male | Glaucoma | 2008 | 2014 | 0 | -1.01  (-1.1, -0.66) |
| Prevalence | Viet Nam | Male | Glaucoma | 2019 | 2021 | 0.132373525 | 0.25  (-0.09, 0.54) |
| Prevalence | Tunisia | Both | Glaucoma | 1990 | 2000 | 0 | -2.06  (-2.09, -2.04) |
| Prevalence | Tunisia | Both | Glaucoma | 2000 | 2005 | 0 | -3.26  (-3.31, -3.21) |
| Prevalence | Tunisia | Both | Glaucoma | 2005 | 2014 | 0 | -1.53  (-1.57, -1.5) |
| Prevalence | Switzerland | Both | Glaucoma | 2019 | 2021 | 0.458708258 | -0.02  (-0.27, 0.15) |
| Prevalence | Tunisia | Female | Glaucoma | 1990 | 1994 | 0 | -2.03  (-2.26, -1.91) |
| Prevalence | Tunisia | Female | Glaucoma | 1994 | 2000 | 0 | -1.8  (-1.86, -1.68) |
| Prevalence | Tunisia | Female | Glaucoma | 2000 | 2005 | 0 | -3.12  (-3.17, -3.05) |
| Prevalence | Tunisia | Female | Glaucoma | 2005 | 2014 | 0 | -1.42  (-1.46, -1.37) |
| Prevalence | Samoa | Female | Glaucoma | 2014 | 2021 | 0.075584883 | 0.25  (-0.06, 0.99) |
| Prevalence | Tunisia | Male | Glaucoma | 1990 | 1998 | 0 | -2.09  (-2.12, -2.05) |
| Prevalence | Tunisia | Male | Glaucoma | 1998 | 2001 | 0 | -2.57  (-2.65, -2.43) |
| Prevalence | Tunisia | Male | Glaucoma | 2001 | 2004 | 0 | -3.48  (-3.58, -3.38) |
| Prevalence | Tunisia | Male | Glaucoma | 2004 | 2007 | 0 | -2.08  (-2.17, -1.95) |
| Prevalence | Tunisia | Male | Glaucoma | 2007 | 2016 | 0 | -1.48  (-1.52, -1.45) |
| Prevalence | United States of America | Male | Glaucoma | 2018 | 2021 | 0.051189762 | 0.26  (-0.01, 0.47) |
| Prevalence | Turkey | Both | Glaucoma | 1990 | 1995 | 0 | -2.05  (-2.18, -1.91) |
| Prevalence | Turkey | Both | Glaucoma | 1995 | 2000 | 0 | 0.55  (0.44, 0.66) |
| Prevalence | Turkey | Both | Glaucoma | 2000 | 2010 | 0 | -2.21  (-2.27, -2.16) |
| Prevalence | Slovenia | Both | Glaucoma | 2018 | 2021 | 0.49470106 | 0.03  (-0.07, 0.18) |
| Prevalence | Turkey | Female | Glaucoma | 1990 | 1995 | 0 | -1.78  (-1.94, -1.63) |
| Prevalence | Turkey | Female | Glaucoma | 1995 | 2000 | 0 | 0.98  (0.85, 1.1) |
| Prevalence | Turkey | Female | Glaucoma | 2000 | 2010 | 0 | -2.13  (-2.21, -2.07) |
| Prevalence | Uruguay | Female | Glaucoma | 2019 | 2021 | 0.047590482 | 0.27  (0, 0.44) |
| Prevalence | Turkey | Male | Glaucoma | 1990 | 1995 | 0 | -2.04  (-2.19, -1.88) |
| Prevalence | Turkey | Male | Glaucoma | 1995 | 2000 | 0.00079984 | 0.37  (0.25, 0.5) |
| Prevalence | Turkey | Male | Glaucoma | 2000 | 2011 | 0 | -2.31  (-2.38, -2.25) |
| Prevalence | Iceland | Male | Glaucoma | 2019 | 2021 | 0.381123775 | 0.3  (-0.38, 0.64) |
| Prevalence | Turkmenistan | Both | Glaucoma | 1990 | 2001 | 0 | -0.37  (-0.39, -0.35) |
| Prevalence | Turkmenistan | Both | Glaucoma | 2001 | 2014 | 0 | -1.03  (-1.05, -1.02) |
| Prevalence | Uruguay | Both | Glaucoma | 2019 | 2021 | 0.264747051 | 0.05  (-0.07, 0.15) |
| Prevalence | Turkmenistan | Female | Glaucoma | 1990 | 2000 | 0.0059988 | -0.25  (-0.31, -0.21) |
| Prevalence | Turkmenistan | Female | Glaucoma | 2000 | 2014 | 0.00079984 | -0.97  (-1.05, -0.93) |
| Prevalence | Slovakia | Female | Glaucoma | 2018 | 2021 | 0 | 0.3  (0.19, 0.39) |
| Prevalence | Turkmenistan | Male | Glaucoma | 1990 | 1994 | 0 | -0.41  (-0.59, -0.31) |
| Prevalence | Turkmenistan | Male | Glaucoma | 1994 | 2001 | 0 | -0.26  (-1.16, -0.19) |
| Prevalence | Turkmenistan | Male | Glaucoma | 2001 | 2018 | 0 | -1.23  (-1.24, -1.22) |
| Prevalence | Zimbabwe | Male | Glaucoma | 2019 | 2021 | 0.073585283 | 0.31  (-0.05, 0.58) |
| Prevalence | Tuvalu | Both | Glaucoma | 1990 | 1996 | 0 | -1.31  (-1.93, -1.09) |
| Prevalence | Tuvalu | Both | Glaucoma | 1996 | 2009 | 0 | -0.81  (-0.87, -0.41) |
| Prevalence | Tuvalu | Both | Glaucoma | 2009 | 2014 | 0 | -1.26  (-1.7, -1.01) |
| Prevalence | Ecuador | Both | Glaucoma | 2018 | 2021 | 0.335932813 | 0.06  (-0.16, 0.44) |
| Prevalence | Tuvalu | Female | Glaucoma | 1990 | 1996 | 0 | -1.35  (-2.54, -0.95) |
| Prevalence | Tuvalu | Female | Glaucoma | 1996 | 2010 | 0.054789042 | -0.62  (-0.71, 0.04) |
| Prevalence | Tuvalu | Female | Glaucoma | 2010 | 2014 | 0.00559888 | -1.73  (-2.48, -1.05) |
| Prevalence | Estonia | Male | Glaucoma | 2018 | 2021 | 0 | 0.32  (0.25, 0.38) |
| Prevalence | Tuvalu | Male | Glaucoma | 1990 | 1994 | 0 | -1.53  (-1.63, -1.47) |
| Prevalence | Tuvalu | Male | Glaucoma | 1994 | 1999 | 0 | -1.34  (-1.41, -1.25) |
| Prevalence | Tuvalu | Male | Glaucoma | 1999 | 2011 | 0 | -1.1  (-1.12, -1.09) |
| Prevalence | Tuvalu | Male | Glaucoma | 2011 | 2015 | 0 | -0.94  (-0.99, -0.87) |
| Prevalence | Tuvalu | Male | Glaucoma | 2015 | 2019 | 0 | -1.39  (-1.43, -1.31) |
| Prevalence | Latvia | Female | Glaucoma | 2019 | 2021 | 0.00959808 | 0.33  (0.08, 0.51) |
| Prevalence | Uganda | Both | Glaucoma | 1990 | 2011 | 0.00239952 | -0.81  (-0.89, -0.72) |
| Prevalence | Uganda | Both | Glaucoma | 2011 | 2015 | 0.00359928 | -3.18  (-4.24, -2.42) |
| Prevalence | Uganda | Both | Glaucoma | 2015 | 2019 | 0.25914817 | -0.62  (-1.3, 0.77) |
| Prevalence | Estonia | Both | Glaucoma | 2018 | 2021 | 0.085182963 | 0.06  (-0.01, 0.15) |
| Prevalence | Uganda | Female | Glaucoma | 1990 | 1994 | 0 | -1.76  (-3.28, -1.02) |
| Prevalence | Uganda | Female | Glaucoma | 1994 | 2011 | 0.045190962 | -0.82  (-0.9, -0.17) |
| Prevalence | Uganda | Female | Glaucoma | 2011 | 2015 | 0.00079984 | -3.24  (-4.3, -2.52) |
| Prevalence | Uganda | Female | Glaucoma | 2015 | 2019 | 0.409118176 | -0.32  (-1, 1.16) |
| Prevalence | Bahamas | Female | Glaucoma | 2019 | 2021 | 0.039992002 | 0.33  (0.02, 0.51) |
| Prevalence | Uganda | Male | Glaucoma | 1990 | 2010 | 0.00079984 | -0.58  (-0.65, -0.51) |
| Prevalence | Uganda | Male | Glaucoma | 2010 | 2015 | 0.00639872 | -2.84  (-3.81, -2.32) |
| Prevalence | Uganda | Male | Glaucoma | 2015 | 2019 | 0.072385523 | -1.05  (-1.66, 0.14) |
| Prevalence | Viet Nam | Female | Glaucoma | 2019 | 2021 | 0.135572885 | 0.34  (-0.28, 0.72) |
| Prevalence | Ukraine | Both | Glaucoma | 1990 | 1994 | 0 | -0.46  (-0.6, -0.38) |
| Prevalence | Ukraine | Both | Glaucoma | 1994 | 2001 | 0 | 0.09  (0.05, 0.13) |
| Prevalence | Ukraine | Both | Glaucoma | 2001 | 2010 | 0 | -1.02  (-1.04, -0.99) |
| Prevalence | Ukraine | Both | Glaucoma | 2010 | 2015 | 0.00439912 | -0.22  (-0.28, -0.15) |
| Prevalence | Ukraine | Both | Glaucoma | 2015 | 2019 | 0 | -0.62  (-0.75, -0.53) |
| Prevalence | Mexico | Both | Glaucoma | 2019 | 2021 | 0.24555089 | 0.1  (-0.14, 0.28) |
| Prevalence | Ukraine | Female | Glaucoma | 1990 | 1994 | 0 | -0.5  (-0.63, -0.41) |
| Prevalence | Ukraine | Female | Glaucoma | 1994 | 2001 | 0.092381524 | -0.03  (-0.06, 0.01) |
| Prevalence | Ukraine | Female | Glaucoma | 2001 | 2009 | 0 | -1.03  (-1.06, -1.01) |
| Prevalence | Ukraine | Female | Glaucoma | 2009 | 2015 | 0 | -0.31  (-0.35, -0.26) |
| Prevalence | Ukraine | Female | Glaucoma | 2015 | 2019 | 0 | -0.62  (-0.75, -0.54) |
| Prevalence | Maldives | Male | Glaucoma | 2019 | 2021 | 0.198760248 | 0.34  (-0.24, 0.78) |
| Prevalence | Ukraine | Male | Glaucoma | 1990 | 1994 | 0.00039992 | -0.34  (-0.65, -0.16) |
| Prevalence | Ukraine | Male | Glaucoma | 1994 | 2001 | 0.00119976 | 0.24  (0.17, 0.37) |
| Prevalence | Ukraine | Male | Glaucoma | 2001 | 2006 | 0.00679864 | -0.95  (-1.04, -0.68) |
| Prevalence | Ukraine | Male | Glaucoma | 2006 | 2009 | 0 | -1.51  (-1.64, -1.24) |
| Prevalence | Ukraine | Male | Glaucoma | 2009 | 2019 | 0 | -0.49  (-0.54, -0.44) |
| Prevalence | Palau | Female | Glaucoma | 2015 | 2021 | 0.074785043 | 0.35  (-0.06, 1.45) |
| Prevalence | United Arab Emirates | Both | Glaucoma | 1990 | 1992 | 0 | -2.45  (-2.61, -2.19) |
| Prevalence | United Arab Emirates | Both | Glaucoma | 1992 | 1995 | 0 | -2.11  (-2.17, -1.3) |
| Prevalence | United Arab Emirates | Both | Glaucoma | 1995 | 2004 | 0 | -1.36  (-1.41, -1.1) |
| Prevalence | United Arab Emirates | Both | Glaucoma | 2004 | 2012 | 0 | -1.08  (-1.15, -1.01) |
| Prevalence | United Arab Emirates | Both | Glaucoma | 2012 | 2018 | 0 | -0.7  (-0.91, -0.64) |
| Prevalence | San Marino | Both | Glaucoma | 2019 | 2021 | 0.137572486 | 0.13  (-0.11, 0.31) |
| Prevalence | United Arab Emirates | Female | Glaucoma | 1990 | 1994 | 0 | -2.25  (-2.34, -2.15) |
| Prevalence | United Arab Emirates | Female | Glaucoma | 1994 | 2012 | 0 | -1.36  (-1.37, -1.34) |
| Prevalence | United Arab Emirates | Female | Glaucoma | 2012 | 2017 | 0 | -1.01  (-1.17, -0.88) |
| Prevalence | Micronesia (Federated States of) | Female | Glaucoma | 2014 | 2021 | 0.018796241 | 0.37  (0.08, 0.93) |
| Prevalence | United Arab Emirates | Male | Glaucoma | 1990 | 1992 | 0 | -2.62  (-2.76, -2.42) |
| Prevalence | United Arab Emirates | Male | Glaucoma | 1992 | 1995 | 0 | -2.2  (-2.26, -1.5) |
| Prevalence | United Arab Emirates | Male | Glaucoma | 1995 | 2011 | 0 | -1.47  (-1.48, -1.46) |
| Prevalence | United Arab Emirates | Male | Glaucoma | 2011 | 2018 | 0 | -1.16  (-1.21, -1.11) |
| Prevalence | Democratic People's Republic of Korea | Male | Glaucoma | 2019 | 2021 | 0.00079984 | 0.37  (0.22, 0.47) |
| Prevalence | United Kingdom | Both | Glaucoma | 1990 | 1996 | 0 | -0.4  (-0.44, -0.35) |
| Prevalence | United Kingdom | Both | Glaucoma | 1996 | 2001 | 0 | -0.73  (-0.78, -0.66) |
| Prevalence | United Kingdom | Both | Glaucoma | 2001 | 2004 | 0 | -1.19  (-1.25, -1.06) |
| Prevalence | United Kingdom | Both | Glaucoma | 2004 | 2015 | 0 | -0.85  (-0.86, -0.83) |
| Prevalence | Norway | Both | Glaucoma | 2019 | 2021 | 0.080783843 | 0.17  (-0.04, 0.3) |
| Prevalence | United Kingdom | Female | Glaucoma | 1990 | 1997 | 0 | -0.46  (-0.51, -0.39) |
| Prevalence | United Kingdom | Female | Glaucoma | 1997 | 2001 | 0 | -0.92  (-1.06, -0.71) |
| Prevalence | United Kingdom | Female | Glaucoma | 2001 | 2005 | 0 | -1.47  (-1.64, -1.33) |
| Prevalence | United Kingdom | Female | Glaucoma | 2005 | 2010 | 0 | -0.96  (-1.05, -0.76) |
| Prevalence | United Kingdom | Female | Glaucoma | 2010 | 2015 | 0 | -1.16  (-1.36, -1.06) |
| Prevalence | Poland | Female | Glaucoma | 2019 | 2021 | 0.0019996 | 0.38  (0.21, 0.51) |
| Prevalence | United Kingdom | Male | Glaucoma | 1990 | 1996 | 0 | -0.43  (-0.45, -0.4) |
| Prevalence | United Kingdom | Male | Glaucoma | 1996 | 2001 | 0 | -0.66  (-0.69, -0.6) |
| Prevalence | United Kingdom | Male | Glaucoma | 2001 | 2008 | 0 | -0.77  (-0.82, -0.75) |
| Prevalence | United Kingdom | Male | Glaucoma | 2008 | 2014 | 0 | -0.67  (-0.74, -0.63) |
| Prevalence | United Kingdom | Male | Glaucoma | 2014 | 2017 | 0 | -0.33  (-0.64, -0.29) |
| Prevalence | American Samoa | Female | Glaucoma | 2015 | 2021 | 0.025594881 | 0.39  (0.05, 1.12) |
| Prevalence | United Republic of Tanzania | Both | Glaucoma | 1990 | 1995 | 0.049990002 | -0.4  (-0.78, 0) |
| Prevalence | United Republic of Tanzania | Both | Glaucoma | 1995 | 2000 | 0.017596481 | -5.74  (-6.02, -4.09) |
| Prevalence | United Republic of Tanzania | Both | Glaucoma | 2000 | 2006 | 0.055588882 | 1.42  (-5.54, 1.79) |
| Prevalence | United Republic of Tanzania | Both | Glaucoma | 2006 | 2015 | 0.090381924 | -0.7  (-1.02, 1.39) |
| Prevalence | United Republic of Tanzania | Both | Glaucoma | 2015 | 2019 | 0.124775045 | 0.83  (-0.82, 1.61) |
| Prevalence | Slovakia | Both | Glaucoma | 2018 | 2021 | 0 | 0.19  (0.11, 0.28) |
| Prevalence | United Republic of Tanzania | Female | Glaucoma | 1990 | 1995 | 0.00279944 | -0.62  (-0.94, -0.29) |
| Prevalence | United Republic of Tanzania | Female | Glaucoma | 1995 | 2000 | 0 | -5.98  (-6.25, -5.7) |
| Prevalence | United Republic of Tanzania | Female | Glaucoma | 2000 | 2005 | 0.00279944 | 1.75  (1.33, 2.12) |
| Prevalence | United Republic of Tanzania | Female | Glaucoma | 2005 | 2015 | 0.00359928 | -0.73  (-0.91, -0.59) |
| Prevalence | United Republic of Tanzania | Female | Glaucoma | 2015 | 2019 | 0.00719856 | 1.44  (0.96, 2.24) |
| Prevalence | Latvia | Male | Glaucoma | 2019 | 2021 | 0.028394321 | 0.4  (0.04, 0.66) |
| Prevalence | United Republic of Tanzania | Male | Glaucoma | 1990 | 1995 | 0.622275545 | -0.11  (-0.74, 0.45) |
| Prevalence | United Republic of Tanzania | Male | Glaucoma | 1995 | 2000 | 0 | -5.59  (-6.05, -5.11) |
| Prevalence | United Republic of Tanzania | Male | Glaucoma | 2000 | 2005 | 0 | 1.78  (1.23, 2.46) |
| Prevalence | Nigeria | Female | Glaucoma | 2015 | 2021 | 0.01159768 | 0.43  (0.28, 0.68) |
| Prevalence | United States Virgin Islands | Both | Glaucoma | 1990 | 1992 | 0 | -1.49  (-1.58, -1.39) |
| Prevalence | United States Virgin Islands | Both | Glaucoma | 1992 | 1995 | 0 | -1.22  (-1.26, -0.9) |
| Prevalence | United States Virgin Islands | Both | Glaucoma | 1995 | 2005 | 0 | -0.79  (-0.81, -0.78) |
| Prevalence | United States Virgin Islands | Both | Glaucoma | 2005 | 2009 | 0 | -1.03  (-1.1, -0.97) |
| Prevalence | United States Virgin Islands | Both | Glaucoma | 2009 | 2015 | 0 | -0.76  (-0.8, -0.73) |
| Prevalence | United States of America | Both | Glaucoma | 2018 | 2021 | 0.074385123 | 0.25  (-0.05, 0.52) |
| Prevalence | United States Virgin Islands | Female | Glaucoma | 1990 | 1994 | 0 | -1.33  (-1.42, -1.28) |
| Prevalence | United States Virgin Islands | Female | Glaucoma | 1994 | 2000 | 0 | -0.96  (-1.01, -0.92) |
| Prevalence | United States Virgin Islands | Female | Glaucoma | 2000 | 2005 | 0 | -0.62  (-0.65, -0.56) |
| Prevalence | United States Virgin Islands | Female | Glaucoma | 2005 | 2010 | 0 | -1.16  (-1.19, -1.13) |
| Prevalence | United States Virgin Islands | Female | Glaucoma | 2010 | 2018 | 0 | -0.7  (-0.72, -0.68) |
| Prevalence | Vanuatu | Female | Glaucoma | 2015 | 2021 | 0.01079784 | 0.43  (0.11, 1.01) |
| Prevalence | United States Virgin Islands | Male | Glaucoma | 1990 | 1994 | 0 | -1.63  (-1.7, -1.58) |
| Prevalence | United States Virgin Islands | Male | Glaucoma | 1994 | 2001 | 0 | -0.77  (-0.8, -0.71) |
| Prevalence | United States Virgin Islands | Male | Glaucoma | 2001 | 2007 | 0 | -0.93  (-1.05, -0.88) |
| Prevalence | United States Virgin Islands | Male | Glaucoma | 2007 | 2015 | 0 | -0.78  (-0.81, -0.73) |
| Prevalence | United States Virgin Islands | Male | Glaucoma | 2015 | 2019 | 0 | -0.34  (-0.42, -0.23) |
| Prevalence | Papua New Guinea | Female | Glaucoma | 2014 | 2021 | 0.021995601 | 0.49  (0.17, 1.1) |
| Prevalence | United States of America | Both | Glaucoma | 1990 | 1995 | 0 | -1.26  (-1.48, -1.12) |
| Prevalence | United States of America | Both | Glaucoma | 1995 | 2010 | 0 | -0.7  (-0.74, -0.67) |
| Prevalence | United States of America | Both | Glaucoma | 2010 | 2013 | 0.084383123 | 0.93  (-0.48, 1.1) |
| Prevalence | United States of America | Both | Glaucoma | 2013 | 2018 | 0 | 1.53  (1.4, 1.8) |
| Prevalence | Viet Nam | Both | Glaucoma | 2019 | 2021 | 0.147970406 | 0.29  (-0.18, 0.63) |
| Prevalence | United States of America | Female | Glaucoma | 1990 | 1997 | 0 | -1.04  (-1.14, -0.97) |
| Prevalence | United States of America | Female | Glaucoma | 1997 | 2005 | 0 | -0.75  (-0.8, -0.54) |
| Prevalence | United States of America | Female | Glaucoma | 2005 | 2010 | 0.014397121 | -0.98  (-1.14, -0.89) |
| Prevalence | United States of America | Female | Glaucoma | 2010 | 2018 | 0 | 1.19  (1.14, 1.26) |
| Prevalence | Hungary | Female | Glaucoma | 2019 | 2021 | 0.017596481 | 0.52  (0.11, 0.81) |
| Prevalence | United States of America | Male | Glaucoma | 1990 | 1994 | 0 | -1.7  (-1.97, -1.5) |
| Prevalence | United States of America | Male | Glaucoma | 1994 | 2010 | 0 | -0.66  (-0.68, -0.63) |
| Prevalence | United States of America | Male | Glaucoma | 2010 | 2015 | 0.00159968 | 0.99  (0.82, 1.07) |
| Prevalence | United States of America | Male | Glaucoma | 2015 | 2018 | 0 | 2.07  (1.76, 2.24) |
| Prevalence | Guam | Female | Glaucoma | 2015 | 2021 | 0.0079984 | 0.52  (0.2, 1.35) |
| Prevalence | Uruguay | Both | Glaucoma | 1990 | 1994 | 0 | -1.17  (-1.23, -1.12) |
| Prevalence | Uruguay | Both | Glaucoma | 1994 | 2000 | 0 | -0.75  (-0.78, -0.72) |
| Prevalence | Uruguay | Both | Glaucoma | 2000 | 2005 | 0.00079984 | -0.16  (-0.2, -0.11) |
| Prevalence | Uruguay | Both | Glaucoma | 2005 | 2010 | 0 | -0.34  (-0.4, -0.28) |
| Prevalence | Uruguay | Both | Glaucoma | 2010 | 2019 | 0 | -0.53  (-0.56, -0.51) |
| Prevalence | Ukraine | Both | Glaucoma | 2019 | 2021 | 0.00079984 | 0.36  (0.16, 0.53) |
| Prevalence | Uruguay | Female | Glaucoma | 1990 | 1993 | 0 | -1.22  (-1.45, -1.06) |
| Prevalence | Uruguay | Female | Glaucoma | 1993 | 1999 | 0.00039992 | -0.91  (-0.96, -0.75) |
| Prevalence | Uruguay | Female | Glaucoma | 1999 | 2007 | 0.00679864 | -0.15  (-0.2, -0.1) |
| Prevalence | Uruguay | Female | Glaucoma | 2007 | 2015 | 0.00239952 | -0.56  (-0.66, -0.21) |
| Prevalence | Uruguay | Female | Glaucoma | 2015 | 2019 | 0 | -0.31  (-0.53, -0.19) |
| Prevalence | Ukraine | Male | Glaucoma | 2019 | 2021 | 0.015596881 | 0.55  (0.13, 0.78) |
| Prevalence | Uruguay | Male | Glaucoma | 1990 | 1994 | 0 | -1.13  (-1.26, -1.06) |
| Prevalence | Uruguay | Male | Glaucoma | 1994 | 2001 | 0 | -0.66  (-0.71, -0.62) |
| Prevalence | Uruguay | Male | Glaucoma | 2001 | 2011 | 0 | -0.27  (-0.29, -0.24) |
| Prevalence | Uruguay | Male | Glaucoma | 2011 | 2015 | 0 | -0.47  (-0.55, -0.36) |
| Prevalence | Uruguay | Male | Glaucoma | 2015 | 2018 | 0 | -0.88  (-0.95, -0.76) |
| Prevalence | Solomon Islands | Female | Glaucoma | 2015 | 2021 | 0.030793841 | 0.56  (0.07, 1.58) |
| Prevalence | Uzbekistan | Both | Glaucoma | 1990 | 1996 | 0 | -0.26  (-0.28, -0.23) |
| Prevalence | Uzbekistan | Both | Glaucoma | 1996 | 2001 | 0 | -0.58  (-0.62, -0.55) |
| Prevalence | Uzbekistan | Both | Glaucoma | 2001 | 2010 | 0 | -0.97  (-0.99, -0.95) |
| Prevalence | Uzbekistan | Both | Glaucoma | 2010 | 2015 | 0 | -1.25  (-1.29, -1.21) |
| Prevalence | Uzbekistan | Both | Glaucoma | 2015 | 2019 | 0 | -0.78  (-0.85, -0.75) |
| Prevalence | Latvia | Both | Glaucoma | 2019 | 2021 | 0.00039992 | 0.4  (0.23, 0.55) |
| Prevalence | Uzbekistan | Female | Glaucoma | 1990 | 1999 | 0.0019996 | -0.31  (-0.37, -0.25) |
| Prevalence | Uzbekistan | Female | Glaucoma | 1999 | 2011 | 0.00079984 | -0.89  (-0.92, -0.83) |
| Prevalence | Uzbekistan | Female | Glaucoma | 2011 | 2014 | 0 | -1.25  (-1.37, -1.01) |
| Prevalence | Belarus | Male | Glaucoma | 2019 | 2021 | 0 | 0.58  (0.44, 0.72) |
| Prevalence | Uzbekistan | Male | Glaucoma | 1990 | 1996 | 0.00079984 | -0.17  (-0.21, -0.12) |
| Prevalence | Uzbekistan | Male | Glaucoma | 1996 | 2001 | 0.00039992 | -0.68  (-0.74, -0.6) |
| Prevalence | Uzbekistan | Male | Glaucoma | 2001 | 2007 | 0 | -1.1  (-1.16, -1.03) |
| Prevalence | Uzbekistan | Male | Glaucoma | 2007 | 2015 | 0 | -1.4  (-1.45, -1.36) |
| Prevalence | Uzbekistan | Male | Glaucoma | 2015 | 2018 | 0 | -1.16  (-1.31, -0.99) |
| Prevalence | Jordan | Female | Glaucoma | 2018 | 2021 | 0 | 0.6  (0.38, 0.8) |
| Prevalence | Vanuatu | Both | Glaucoma | 1990 | 1995 | 0 | -0.66  (-0.82, -0.51) |
| Prevalence | Vanuatu | Both | Glaucoma | 1995 | 2000 | 0 | -1.96  (-2.14, -1.8) |
| Prevalence | Vanuatu | Both | Glaucoma | 2000 | 2006 | 0.023595281 | -0.34  (-0.46, -0.16) |
| Prevalence | Vanuatu | Both | Glaucoma | 2006 | 2015 | 0 | -0.94  (-1.08, -0.87) |
| Prevalence | Niger | Both | Glaucoma | 2016 | 2021 | 0 | 0.72  (0.66, 0.81) |
| Prevalence | Vanuatu | Female | Glaucoma | 1990 | 1996 | 0.00239952 | -0.69  (-0.9, -0.41) |
| Prevalence | Vanuatu | Female | Glaucoma | 1996 | 1999 | 0.00039992 | -3.85  (-4.25, -3.07) |
| Prevalence | Vanuatu | Female | Glaucoma | 1999 | 2007 | 0.105578884 | -0.22  (-0.39, 0.13) |
| Prevalence | Vanuatu | Female | Glaucoma | 2007 | 2015 | 0 | -0.94  (-1.62, -0.75) |
| Prevalence | Kiribati | Female | Glaucoma | 2015 | 2021 | 0.00239952 | 0.7  (0.25, 1.88) |
| Prevalence | Vanuatu | Male | Glaucoma | 1990 | 1995 | 0 | -0.72  (-0.76, -0.67) |
| Prevalence | Vanuatu | Male | Glaucoma | 1995 | 2000 | 0 | -1.28  (-1.32, -1.24) |
| Prevalence | Vanuatu | Male | Glaucoma | 2000 | 2005 | 0 | -0.42  (-0.48, -0.38) |
| Prevalence | Vanuatu | Male | Glaucoma | 2005 | 2010 | 0 | -0.94  (-1.03, -0.88) |
| Prevalence | Vanuatu | Male | Glaucoma | 2010 | 2014 | 0 | -0.77  (-0.85, -0.68) |
| Prevalence | Ecuador | Male | Glaucoma | 2018 | 2021 | 0 | 0.74  (0.42, 1.37) |
| Prevalence | Venezuela (Bolivarian Republic of) | Both | Glaucoma | 1990 | 1994 | 0 | -1.89  (-2.33, -1.67) |
| Prevalence | Venezuela (Bolivarian Republic of) | Both | Glaucoma | 1994 | 1998 | 0 | -0.79  (-1.2, -0.37) |
| Prevalence | Venezuela (Bolivarian Republic of) | Both | Glaucoma | 1998 | 2006 | 0.183563287 | -0.07  (-0.15, 0.09) |
| Prevalence | Venezuela (Bolivarian Republic of) | Both | Glaucoma | 2006 | 2009 | 0.00559888 | -1.52  (-1.68, -1.12) |
| Prevalence | Bhutan | Both | Glaucoma | 2019 | 2021 | 0.195560888 | 0.76  (-0.53, 1.31) |
| Prevalence | Venezuela (Bolivarian Republic of) | Female | Glaucoma | 1990 | 1995 | 0 | -1.54  (-1.63, -1.44) |
| Prevalence | Venezuela (Bolivarian Republic of) | Female | Glaucoma | 1995 | 2006 | 0 | -0.25  (-0.29, -0.2) |
| Prevalence | Venezuela (Bolivarian Republic of) | Female | Glaucoma | 2006 | 2010 | 0 | -0.97  (-1.22, -0.78) |
| Prevalence | Venezuela (Bolivarian Republic of) | Female | Glaucoma | 2010 | 2015 | 0.00119976 | 0.23  (0.11, 0.45) |
| Prevalence | Venezuela (Bolivarian Republic of) | Female | Glaucoma | 2015 | 2019 | 0.00079984 | -0.43  (-0.7, -0.28) |
| Prevalence | Venezuela (Bolivarian Republic of) | Female | Glaucoma | 2019 | 2021 | 0.00079984 | 0.81  (0.37, 1.14) |
| Prevalence | Venezuela (Bolivarian Republic of) | Male | Glaucoma | 1990 | 1994 | 0 | -2.13  (-2.37, -1.96) |
| Prevalence | Venezuela (Bolivarian Republic of) | Male | Glaucoma | 1994 | 1999 | 0 | -0.8  (-1, -0.64) |
| Prevalence | Venezuela (Bolivarian Republic of) | Male | Glaucoma | 1999 | 2005 | 0.00559888 | 0.21  (0.12, 0.33) |
| Prevalence | Venezuela (Bolivarian Republic of) | Male | Glaucoma | 2005 | 2010 | 0 | -1.3  (-1.42, -1.2) |
| Prevalence | Bhutan | Female | Glaucoma | 2019 | 2021 | 0.112777445 | 0.99  (-0.22, 1.63) |
| Prevalence | Viet Nam | Both | Glaucoma | 1990 | 1993 | 0 | 1.81  (1.54, 2.14) |
| Prevalence | Viet Nam | Both | Glaucoma | 1993 | 1996 | 0.335132973 | -0.1  (-0.33, 0.15) |
| Prevalence | Viet Nam | Both | Glaucoma | 1996 | 2005 | 0 | -2.26  (-2.32, -2.2) |
| Prevalence | Viet Nam | Both | Glaucoma | 2005 | 2013 | 0 | -1.2  (-1.27, -1.11) |
| Prevalence | Viet Nam | Both | Glaucoma | 2013 | 2019 | 0 | -1.84  (-2, -1.72) |
| Prevalence | Republic of Korea | Both | Glaucoma | 2019 | 2021 | 0.00239952 | 0.79  (0.32, 1.06) |
| Prevalence | Viet Nam | Female | Glaucoma | 1990 | 1993 | 0 | 2.89  (2.57, 3.28) |
| Prevalence | Viet Nam | Female | Glaucoma | 1993 | 1996 | 0.194761048 | 0.16  (-0.14, 0.44) |
| Prevalence | Viet Nam | Female | Glaucoma | 1996 | 2005 | 0 | -2.5  (-2.57, -2.43) |
| Prevalence | Viet Nam | Female | Glaucoma | 2005 | 2012 | 0 | -1.19  (-1.28, -1.06) |
| Prevalence | Viet Nam | Female | Glaucoma | 2012 | 2019 | 0 | -1.97  (-2.13, -1.87) |
| Prevalence | India | Female | Glaucoma | 2018 | 2021 | 0.00039992 | 1.07  (0.53, 1.88) |
| Prevalence | Viet Nam | Male | Glaucoma | 1990 | 1994 | 0.019196161 | 0.21  (0.07, 0.35) |
| Prevalence | Viet Nam | Male | Glaucoma | 1994 | 1999 | 0 | -1.2  (-1.34, -1.08) |
| Prevalence | Viet Nam | Male | Glaucoma | 1999 | 2005 | 0 | -2.04  (-2.13, -1.96) |
| Prevalence | Viet Nam | Male | Glaucoma | 2005 | 2015 | 0 | -1.17  (-1.21, -1.13) |
| Prevalence | Viet Nam | Male | Glaucoma | 2015 | 2019 | 0 | -1.87  (-2.06, -1.7) |
| Prevalence | Republic of Korea | Female | Glaucoma | 2019 | 2021 | 0 | 1.13  (0.64, 1.44) |
| Prevalence | Yemen | Both | Glaucoma | 1990 | 1993 | 0 | 2.43  (2.22, 2.73) |
| Prevalence | Yemen | Both | Glaucoma | 1993 | 1996 | 0.00039992 | 1.26  (0.92, 1.53) |
| Prevalence | Yemen | Both | Glaucoma | 1996 | 2001 | 0.019196161 | 0.22  (0.07, 0.34) |
| Prevalence | Yemen | Both | Glaucoma | 2001 | 2005 | 0 | -0.77  (-0.97, -0.56) |
| Prevalence | Yemen | Both | Glaucoma | 2005 | 2011 | 0 | -1.49  (-1.67, -1.39) |
| Prevalence | India | Both | Glaucoma | 2018 | 2021 | 0.016396721 | 0.88  (0.17, 2.01) |
| Prevalence | Yemen | Female | Glaucoma | 1990 | 1994 | 0 | 2.34  (1.97, 2.94) |
| Prevalence | Yemen | Female | Glaucoma | 1994 | 2000 | 0.00159968 | 0.89  (0.56, 1.23) |
| Prevalence | Yemen | Female | Glaucoma | 2000 | 2003 | 0.229154169 | -0.28  (-1.28, 0.53) |
| Prevalence | Yemen | Female | Glaucoma | 2003 | 2011 | 0.00639872 | -1.25  (-1.86, -0.38) |
| Prevalence | India | Male | Glaucoma | 2019 | 2021 | 0.00119976 | 1.71  (0.63, 2.46) |
| Prevalence | Yemen | Male | Glaucoma | 1990 | 1994 | 0 | 1.64  (1.45, 1.92) |
| Prevalence | Yemen | Male | Glaucoma | 1994 | 1997 | 0.181163767 | 0.24  (-0.34, 0.83) |
| Prevalence | Yemen | Male | Glaucoma | 1997 | 2004 | 0 | -0.7  (-0.86, -0.61) |
| Prevalence | Yemen | Male | Glaucoma | 2004 | 2012 | 0 | -1.66  (-1.76, -1.57) |
| Prevalence | Niger | Female | Glaucoma | 2019 | 2021 | 0 | 1.74  (1.5, 1.96) |
| Prevalence | Zambia | Both | Glaucoma | 1990 | 1992 | 0 | -0.9  (-1.15, -0.53) |
| Prevalence | Zambia | Both | Glaucoma | 1992 | 1995 | 0.387122575 | -0.42  (-0.53, 0.67) |
| Prevalence | Zambia | Both | Glaucoma | 1995 | 2000 | 0.25714857 | 0.61  (-0.76, 0.74) |
| Prevalence | Zambia | Both | Glaucoma | 2000 | 2006 | 0.00119976 | -0.72  (-1.15, -0.63) |
| Prevalence | Zambia | Both | Glaucoma | 2006 | 2010 | 0 | -1.11  (-1.66, -1) |
| Prevalence | Iran (Islamic Republic of) | Both | Glaucoma | 2019 | 2021 | 0 | 3.03  (2.25, 3.95) |
| Prevalence | Zambia | Female | Glaucoma | 1990 | 1995 | 0.00319936 | -0.57  (-0.82, -0.42) |
| Prevalence | Zambia | Female | Glaucoma | 1995 | 2000 | 0.028394321 | 0.42  (0.24, 0.66) |
| Prevalence | Zambia | Female | Glaucoma | 2000 | 2006 | 0.035192961 | -0.4  (-0.49, -0.21) |
| Prevalence | Zambia | Female | Glaucoma | 2006 | 2010 | 0.00079984 | -0.98  (-1.16, -0.61) |
| Prevalence | Zambia | Female | Glaucoma | 2010 | 2014 | 0 | -1.51  (-1.71, -1.33) |
| Prevalence | Iran (Islamic Republic of) | Female | Glaucoma | 2019 | 2021 | 0 | 2.88  (2.16, 3.7) |
| Prevalence | Zambia | Male | Glaucoma | 1990 | 1995 | 0.00039992 | -0.59  (-0.81, -0.39) |
| Prevalence | Zambia | Male | Glaucoma | 1995 | 2000 | 0.0059988 | 0.93  (0.75, 1.18) |
| Prevalence | Zambia | Male | Glaucoma | 2000 | 2007 | 0.017196561 | -0.58  (-0.68, -0.37) |
| Prevalence | Zambia | Male | Glaucoma | 2007 | 2012 | 0 | -1.06  (-1.39, -0.84) |
| Prevalence | Iran (Islamic Republic of) | Male | Glaucoma | 2019 | 2021 | 0 | 3.22  (2.39, 4.19) |
| Prevalence | Zimbabwe | Both | Glaucoma | 1990 | 1995 | 0 | -0.69  (-0.84, -0.61) |
| Prevalence | Zimbabwe | Both | Glaucoma | 1995 | 2006 | 0.00079984 | -0.21  (-0.23, -0.17) |
| Prevalence | Zimbabwe | Both | Glaucoma | 2006 | 2009 | 0 | -0.66  (-0.74, -0.47) |
| Prevalence | South Africa | Both | Glaucoma | 2019 | 2021 | 0 | 7.77  (5.54, 9.3) |
| Prevalence | Zimbabwe | Female | Glaucoma | 1990 | 1994 | 0 | -0.75  (-0.92, -0.67) |
| Prevalence | Zimbabwe | Female | Glaucoma | 1994 | 2003 | 0.016396721 | -0.14  (-0.17, -0.08) |
| Prevalence | Zimbabwe | Female | Glaucoma | 2003 | 2006 | 0.00039992 | -0.39  (-0.7, -0.19) |
| Prevalence | Zimbabwe | Female | Glaucoma | 2006 | 2009 | 0 | -0.85  (-0.94, -0.38) |
| Prevalence | Zimbabwe | Female | Glaucoma | 2009 | 2014 | 0.027594481 | -0.33  (-0.43, -0.1) |
| Prevalence | South Africa | Male | Glaucoma | 2019 | 2021 | 0 | 6.85  (5.58, 7.92) |
| Prevalence | Zimbabwe | Male | Glaucoma | 1990 | 1995 | 0 | -0.75  (-0.95, -0.63) |
| Prevalence | Zimbabwe | Male | Glaucoma | 1995 | 2006 | 0.035192961 | -0.21  (-0.25, -0.07) |
| Prevalence | Zimbabwe | Male | Glaucoma | 2006 | 2009 | 0.00119976 | -0.51  (-0.63, -0.29) |
| Prevalence | Zimbabwe | Male | Glaucoma | 2009 | 2016 | 0.347130574 | -0.06  (-0.11, 0.18) |
| Prevalence | Zimbabwe | Male | Glaucoma | 2016 | 2019 | 0.00319936 | -0.58  (-0.75, -0.37) |
| Prevalence | South Africa | Female | Glaucoma | 2019 | 2021 | 0 | 8.44  (5.46, 10.26) |
| Abbreviations: DALYs, Disability-Adjusted Life Years; APC, annual percentage change. | | | | | | | |

## Supplementary Table 5

| Supplementary Table 5 Comparison of joinpoint-derived annual percentage changes (APC) for glaucoma prevalence in selected countries between GBD 2021 and GBD 2023, focusing on the pandemic period. | | | | | | |
| --- | --- | --- | --- | --- | --- | --- |
| measure | location | sex | Segment.Start | Segment.End | P.Value | APC_95CI |
| Prevalence | China | Both | 2019 | 2021 | 0 | -13.09  (-15.95, -8.47) |
| Prevalence | Qatar | Both | 2019 | 2021 | 0 | -8.54  (-11.03, -5.82) |
| Prevalence | Uganda | Both | 2019 | 2021 | 0 | -6.45  (-8.16, -4.3) |
| Prevalence | Sudan | Both | 2019 | 2021 | 0 | -5.89  (-7.16, -3.76) |
| Prevalence | Nepal | Both | 2019 | 2021 | 0 | -4.31  (-4.98, -3.51) |
| Prevalence | Fiji | Both | 2019 | 2021 | 0 | -4.17  (-6.07, -2.24) |
| Prevalence | Timor-Leste | Both | 2019 | 2021 | 0 | -4.04  (-5.16, -2.08) |
| Prevalence | Libya | Both | 2019 | 2021 | 0.00039992 | -3.13  (-4.76, -0.61) |
| Prevalence | Afghanistan | Both | 2019 | 2021 | 0 | -2.81  (-3.56, -1.63) |
| Prevalence | Brazil | Both | 2015 | 2021 | 0 | -1.95  (-2.33, -1.54) |
| Prevalence | Afghanistan | Both | 2019 | 2023 | 0 | -4.83  (-5.78, -4.06) |
| Prevalence | Brazil | Both | 2015 | 2023 | 0 | -0.83  (-0.93, -0.75) |
| Prevalence | China | Both | 2019 | 2023 | 0 | -4.78  (-5.9, -4.03) |
| Prevalence | Fiji | Both | 2019 | 2023 | 0 | -1.14  (-1.27, -1) |
| Prevalence | Libya | Both | 2018 | 2023 | 0 | -2.52  (-2.84, -2.19) |
| Prevalence | Nepal | Both | 2019 | 2023 | 0.49470106 | -0.05  (-0.35, 0.12) |
| Prevalence | Qatar | Both | 2020 | 2023 | 0 | -0.8  (-0.91, -0.69) |
| Prevalence | Sudan | Both | 2019 | 2023 | 0 | -5.09  (-5.9, -4.39) |
| Prevalence | Timor-Leste | Both | 2017 | 2023 | 0 | -3.84  (-4.05, -3.52) |
| Prevalence | Uganda | Both | 2019 | 2023 | 0 | -3.45  (-4.06, -3.04) |

## Supplementary Fig.1

Pearson correlation analysis between the SDI and ASR of prevalence (A) and DALYs (B) for glaucoma at the country and territorial levels in 2021
